# Uncovering the social determinants of brain injury rehabilitation

**DOI:** 10.1177/13591053231166263

**Published:** 2023-04-07

**Authors:** Stephen Dunne, Glenn P Williams, Chloe Bradbury, Tamsin Keyes, Alison R Lane, Keming Yang, Amanda Ellison

**Affiliations:** 1 Northumbria University, UK; 2 NIHR Applied Research Collaboration North-East and North Cumbria, UK; 3 University of Sunderland, UK; 4 Headway – The Brain Injury Association, UK; 5 Durham University, UK

**Keywords:** brain injury, health, lockdown, loneliness, social isolation

## Abstract

Social determinants of health (SDH), such as social isolation and loneliness, are often more frequently experienced in brain injury survivors. The paper explores the personal experiences of loneliness among brain injury survivors during lockdown to negate health inequalities and improve rehabilitation for this population in the future. Twenty-four brain injury survivors participated in semi-structured interviews and questionnaires relating to loneliness, resilience and wellbeing. Three themes (the experience of loneliness, loneliness during the pandemic and loneliness after the pandemic) explored survivors’ experiences of loneliness generally post-brain injury, but also chronicle how these feelings developed in lockdown and survivors’ feelings regarding society returning to ‘normal’. Future interventions should focus on reframing survivors’ beliefs regarding societal expectations and minimise the pressure they experience to keep up with their peers physically and emotionally. Additionally, we recommend creating accessible peer support options for all brain injury survivors as an important step for alleviating loneliness.

## Introduction

Loneliness, a determinant of health defined as the unpleasant feeling experienced when our desired social experience does not match our actual social experiences (Cacioppo et al., 2015), is a significant threat to mental and physical health (Hawkley and Cacioppo, 2010). Particular individuals or groups, such as those with a disability, may be more vulnerable to loneliness (Durcan and Bell, 2015). This study explores the experiences of one such group: brain injury survivors during the particularly vulnerable period exemplified by Covid-19 induced lockdowns.

Brain injury is a leading cause of disability worldwide (Maas et al., 2017) and brain injury survivors have a unique profile when compared to other patient groups. Following brain injury, it is not unusual for individuals to be left with impairments of varying severity, including deficits affecting movement, cognition, speech and regulation of emotions for instance (Norlander et al., 2016). These issues impact on an individual’s self-esteem and identity (Salter et al., 2008) which in turn hinder social connections and engagement (Carod-Artal, 2012), with several studies highlighting that brain injury is associated with a decrease in the quantity or quality of friendships, social contacts and meaningful relationships (Dijkers, 2004; Douglas, 2020). The resulting loneliness and/or social isolation is a universal long-term consequence of living with acquired brain injury (Kumar et al., 2020). Even after learning to accept and adapt to any physical impairments, developing and maintaining social contacts and significant relationships is still problematic (Tomberg et al., 2005). Therefore, brain injury has long-lasting ramifications on wellbeing and quality of life (Haley et al., 2011; Hawthorne et al., 2009) often in the form of chronic mental health issues such as anxiety and depression (Fleminger et al., 2003; Proctor and Best, 2019).

Lowe et al. (2021) have furthered understanding of this complex issue, highlighting the importance of therapeutic relationships in mediating reconnection and reintegration following brain injury and a focus on alleviating survivors’ perceptions of feeling left behind to avoid negative mental health outcomes. However, since these findings were published, the UK was operating in some form of lockdown for over 6 months, experiencing three national lockdowns since March 2020. Initially, people were not permitted to leave their homes without a reasonable excuse, but over time these restrictions were relaxed, permitting non-essential shops to re-open and small social gatherings to take place. A second lockdown lasting 1 month began in November 2020 giving way to regional-specific restrictions depending on viral spread. A third national lockdown was called on January 2021. By July 2021, most legal limits on social contact were removed. The lockdowns, social distancing and self-isolation enforced because of the COVID-19 pandemic have resulted in a negative psycho-social impact on the general population (Dawson and Golijani-Moghaddam, 2020) and an exacerbation in feelings of loneliness (Bu et al., 2020). For brain injury survivors the restrictions brought additional challenges as rehabilitation programmes and peer support networking groups were required to undergo significant adaptation: neurorehabilitation services cancelled face-to-face appointments and community projects (Coetzer and Bichard, 2020), clinicians were forced to shield, work from home or were re-deployed (De Silva et al., 2020; Laxe et al., 2020) and there was a switch to video calling and telephone communication where possible.

For a population already at an increased risk of experiencing loneliness, exploring the effect of lockdown measures on brain injury survivors is of paramount importance. Currently there is a lack of in-depth understanding of brain injury survivors’ experiences and mental health when considering the preventative measures imposed due to the COVID-19 pandemic and the pandemic itself and how we can use this knowledge to inform future strategies for brain injury rehabilitation moving forward. We report findings that will further our understanding of the impact of the pandemic on the post-brain injury experience, ramifications for recovery of these patients and lessons that could be learned for approaches to loneliness in this particularly vulnerable subset of patients in non-pandemic conditions.

## Methods

### Methodological approach and research paradigm

This research utilised a mixed methods approach. Qualitative interviews were placed within an interpretivist paradigm, putting emphasis on understanding the subjective experiences of individuals (Kivunja and Kuyini, 2017). Due to our interest in exploring brain injury survivors’ experiences of loneliness in lockdown we utilised a reflexive thematic analysis (Braun and Clarke, 2013, 2021). We have followed the Standards for Reporting Qualitative Research (O’Brien et al., 2014). Quantitative measures were collected to document participants’ resilience, loneliness and wellbeing.

### Researcher characteristics and reflexivity

The multi-disciplinary research team consisted of a mix of academic members and those with working experience of brain injury, with expertise in brain injury rehabilitation, health psychology and research methods.

### Ethical approval

The project was conducted in accordance with the Declaration of Helsinki and received approval from ethics committees at the authors’ respective universities and Headway. The authors received no specific funding for this project.

### Sampling strategy

Third sector organisations such as Headway and The Stroke Association provide information, support and services to brain injury survivors, their families and carers. Study information was circulated through these organisations via their research web pages and among their national support groups. Additionally, project information was distributed via the researchers’ own connections to brain injury groups within the NHS and social media accounts. Eligible participants had to be aged 18 years or above, living in the UK and have had a brain injury in the past.

Prospective participants were sent an information sheet and consent form electronically at least 3 days prior to interview, allowing them time to read the study information and decide whether to participate. When participating, this practice was repeated, with the researcher reading the information sheet and consent form aloud and ensuring that participants had comprehended each element prior to participation.

### Data collection

Interviews were conducted by SD and CB and took place between January and March 2021, prior to the pandemic restrictions being lifted in the UK. As interviews took place over the period of national lockdown, all interviews were conducted online via the Microsoft Teams video conferencing software. Participants had varying technological experience but were assisted in using this platform by the research team as necessary. Interviews, typically lasting approximately 1 hour (mean = 61.85; median = 63.89; standard deviation = 21.29) were audio and video recorded. The researchers monitored participants for signs of fatigue and emotional distress throughout, and breaks were taken whenever required (Carlsson et al., 2007; Dalemans et al., 2009). During the interview, in addition to qualitative questions, quantitative measures were collected to document participants’ resilience, loneliness and wellbeing. Due to previous literature highlighting the loss of key relationships after brain injury, the De Jong Gierveld six-item loneliness scale (De Jong Gierveld and Van Tilburg, 2010) was used to assess survivors’ emotional and social loneliness. Survivors’ resilience was measured using the six-item Brief Resilience Scale (Smith et al., 2008). Finally, survivors completed the 14-item Warwick Edinburgh Mental Well-being Scale (WEMWBS; Tennant et al., 2007) measuring their wellbeing over the past 2-week period. The short versions of scales were specifically selected to minimise fatigue.

### Participants

In total 24 participants (8 females, 16 males; 24–68 years old; *M*_age_ = 48.65, SD_age_ = 11.32) were recruited for the project. One participant was excluded as they did not currently live in the UK. Two participants reported having had more than one brain injury (P20 and P25). Most participants showed mild to severe brain injuries associated with a range of impairments, assessed through the discourse of participant’s symptoms and experiences. Seven participants were medically retired or on long-term sick leave. All participants had access to the internet via a home computer and laptop. Observations of the interviewees and the discussions had during interviews led us to believe they were from diverse socio-economic backgrounds. Full participant demographics can be found in the Supplemental Material.

### Data processing and analysis

After data was transcribed, SD and CB familiarised themselves with the data by watching the recordings, conducting multiple readings of the transcripts and made notes of preliminary ideas. SD independently conducted general coding, highlighting and labelling any relevant passages of text with descriptive code throughout all transcripts. To explore patterns of meaning, similarly coded extracts, along with a description of each code, were placed together in a table. After all transcripts had been coded, SD and CB met to discuss the interpretations of the data, agreeing codes and discarding any not related to the research enquiry. All codes were sorted into categories and subsequently refined into themes. Data and coding within themes were analysed and inter-relationships between themes visualised. The themes were named and defined, and a written summary of the analysis grounded in participant data was constructed.

### Techniques to enhance trustworthiness

To ensure rigour in our qualitative analysis we adhered to the 15-point checklist criteria provided by Braun and Clarke (2006, 2013) which provides guidance on the processes of transcription, coding, analysis and the creation of a written report. Examples of quality control within this study include ensuring transcribed data have the appropriate level of detail, checking recordings for accuracy, checking all data have been considered equally, confirming coded items have been collated and themes checked against the original data set, reviewing whether all data have been interpreted rather than summarised and that all analysis matches the data set and stating all assumptions and approaches to thematic analysis. Additionally, we employed researcher triangulation, with SD and CB having conducted the interviews, not only discussing, and evaluating their impressions of the data but also discussing their experiences of conducting the interview. Finally, throughout the data processing and analysis phases, SD kept a reflexive journal to log their thoughts, motivations, values and assumptions in the research process.

## Results

### Statistical analysis

We used Python (van Rossum, 1995) [Version 3.9.5] and the Python packages PyMC (Salvatier et al., 2016) [Version 4.0.0b2], ArviZ (Kumar et al., 2019) [Version 0.11.4], NumPy (Harris et al., 2020) [Version 1.21.5], pandas (McKinney, 2010) [Version 1.4.1], Aesara (Bastien et al., 2021) [Version 2.3.8, Matplotlib (Hunter, 2007) [Version 3.5.1] and seaborn (Waskom, 2021) [Version 0.11.2] for all data processing, analysis and presentation.

Primary analyses aimed to estimate the effect of resilience (measured using the Brief Resilience Scale; BRS) (median = 3.5, range = 1.83–5, IQR = 1.25) and loneliness (measured using the De Jong Gierveld Scale; DJG) (median = 3, range = 0–6, IQR = 2.25) on mental wellbeing (measured using the Warwick-Edinburgh Mental Well-being Scale; WEMWBS) (median = 46, range = 23–63, IQR = 14.5) using Bayesian regression modelling. Model comparison was performed using Pareto-smoothed importance sampling leave-one-out cross-validation to establish which of several potential models are more likely to make accurate out-of-sample predictions. This showed that the model treating BRS as a sum-coded categorical predictor (low = −1, medium-high = 1) and DJG as a centred continuous predictor performed best^1^ ( $\hat{elpd}$ = –88.69, SE = 4.56, weight = 0.576). For full details of the models, including prior specification, parameterisation, model comparisons, diagnostics and a table of parameter estimates, see https://osf.io/rbtms/.

Overall, DJG had a negative but unreliable effect on mental wellbeing ( $\hat{\beta }$ = –1.37, 95% CI = (–2.98, 0.37)). On average, a one unit increase in DJG was associated with a 2.74 unit decrease in WEMWBS (95% CI = ^~^(5.96, 0.74)). Indeed, as the 95% credible interval spans zero there is not enough evidence to conclude that loneliness had any effect on WEMWBS. However, BRS had a reliable, positive effect on mental wellbeing ( $\hat{\beta }$ = 6.31, 95% CI = (1.85, 2.67)). On average, when going from a low BRS score to a medium-high BRS score there was a 12.62 unit increase in WEMWBS (95% CI = (5.34, 19.94)). We therefore conclude that while loneliness does not reliably affect mental wellbeing among brain injury survivors in lockdown, higher scores of resilience improve mental wellbeing.

### Qualitative analysis

Analysis of the interviews led to the development of three overarching themes to describe brain injury survivors’ experiences of loneliness during lockdown and beyond (See Figure 1 in Supplemental Material).

#### The experience of loneliness

##### Loneliness is being alone

When asked to define loneliness, our interviewees described objective events or states responsible for their feelings rather than describing the subjective state of how they feel when lonely.

*Obviously loneliness means being alone, not having interaction or in my case stimulation. Something missing there, missing life.*

Others emphasised the important role communication plays in alleviating loneliness, with several participants highlighting that conversation and regular social interaction are critical. Without interactions with friends or family, participants highlighted loneliness was an inevitable consequence.

*I think when you* *don’t* *have any way of interacting with family, friends, people in the community and* you’re *locked away in your own wee world. And what you* don’t *want is to be in a place where you have no contact with anybody.*

##### Loneliness is a direct result of brain injury

One recurring theme was the causal relationship between brain injury and loneliness such that loneliness occurred as a direct result of participants’ experiences. Participants described feeling like they are living ‘life in a vacuum’, ‘out of context’ or ‘not feeling relevant’ due to associated issues directly attributable to their brain injury. Either partial or complete loss of cognitive or physical abilities can mean immobility, difficulties in communication with others and stripping back the contents of one’s life (such as employment and social commitments) so that daily life remains manageable, leading to feelings of loneliness.

Additionally, seeking help for loneliness was seen as difficult. Embarrassment, guilt, awkwardness and the fear of being seen as a burden were all prevalent in participants’ responses. One participant eloquently described that the help-seeking process was easier for the person offering help than for the person seeking it, and this imbalance may be the reason why it is harder for brain injury survivors to have those difficult conversations.

*the predominant voice that I hear is people offering to help people. You* *don’t* *hear many people actually say I am a person who needs help. And* it’s *much harder to ask for help. Much, much harder than it is to be the person who offers help.*

#### Loneliness during lockdown

##### A level playing field

Survivors felt the pandemic had raised awareness of what it was like to live with a brain injury for those without a brain injury; reporting the pandemic made them feel ‘more inclusive in the world again’, diminishing feelings of loneliness and social isolation as ‘everyone is in the same boat’.

*in a strange sort of way it made you feel less isolated and lonely because every time you turned on the radio or television everyone was talking about how lonely they were and all that so in a way it made you feel you were kinda part of something a bit more in a strange sort of way, you know.*

Survivors hailed the pandemic as creating an environment of increased understanding, with those who had not sustained a brain injury experiencing what some survivors described as ‘a life in lockdown’. The changes experienced after a brain injury require a process of acceptance and adaptation after the injury. This process of adaptation was described as a positive learning experience, with survivors having already adapted to an adverse set of circumstances and therefore lessening the impact of having to adapt to another life-changing event in the form of the pandemic.

*it was just brilliant for me because everybody was suddenly in, what I say* *‘the same boat’ y’know* *a-and it was almost like I was ahead of the game because it had happened to me because it happened years before that I was eh actually able to deal with it more and it was great*.

##### No different to normal

As survivors had already experienced a process of adaptation post-injury, many survivors pointed out that life was ‘business as usual’ throughout the pandemic. Social habits, freedom and quality of life were generally described as remaining the same as before.

*I think a lot of this is a lot of it is less about lockdown, per se. But for me*, it’s *probably more about. . .It will have been more about our lives being the same. But the difference is to other* *people’s* *lives.*

Rather, survivors commented that the real changes were occurring to those without a brain injury. Participants suggested those without a brain injury were experiencing a period of adaptation similar to that experienced by brain injury survivors’ post-injury. Survivors believed this heightened awareness of what life is like with a brain injury, leading to increased feelings of inclusion.

##### Buying space and time

Although the pandemic may not have had a huge effect on the lives of our participants, there were some notable positives that the pandemic produced. The majority of our interviewees experienced less fatigue, a reduction in societal expectations to achieve and engage and ‘the world was a much quieter place’. This afforded several of the survivors we spoke to an opportunity to reflect.

*having the whole of life disrupted for everybody has given me that breathing space to acknowledge that I* *can’t* *continue in the way that I had done and sad though that might be at least it is also a degree of acceptance. [Lockdown] had the benefit of buying the space and time to actually break from my former lifestyle and give me space then to maybe form a new lifestyle and* *that’s* *very difficult to do when you first have a brain injury because you automatically want to try and go back to as much as what you used to do before. And so* it’s *given me that space to change.*

This period of reflection, as noted in the quote above, was important in providing our respondents an opportunity to adapt on their own terms, without feeling pressured.

##### Fellowship of like-minded people

The positive impact of peer support has been identified throughout the literature, providing opportunities to self-disclose, to be accepted and to ‘talk to other people who have had the same sort of issues’. Survivors spoke of the importance of peer support, specifically throughout the pandemic.

*just the fact that, like, there are other people out there who do have the same thoughts that* *I’ve* *had over the years, and perhaps never sort of discussed myself.*

The opportunity to engage with ‘like-minded people’ who have had ‘the same sort of issues’ cannot be understated, with all participants discussing how important engaging with others with a brain injury has been throughout the pandemic, and their rehabilitation generally.

*psychologically the adjustment you have to go through if you have a brain injury is probably something you can only really understand if* you’ve *had a brain injury.*

##### Technology has been a lifesaver

A key factor discussed across all survivors was technology and its facilitative properties in maintaining social connection and alleviating isolation over the course of the pandemic. Utilising social media and video conferencing platforms enabled all those interviewed to engage with family friends and loved ones during the pandemic.

*the Zoom calls, it made a big difference. And you* didn’t *feel as kinda out on your own because of* what’s *happened to me, and I* *can’t* *do things. But if you involve yourself with your friends, and you do the best you can to try and keep it going and not be so lonely.*

Specific positives highlighted by survivors were the accessibility of the technology, the inclusivity it afforded and how often video conferencing meant that physical disabilities as a result of brain injury could be hidden or diminished.

*[the] shift to online has meant that* *I’ve* *been able to be part of things. The downsides*, *it’s* *very two dimensional. And having more personal conversations are difficult online. So not being able to have those more detailed conversations with friends was difficult, particularly, you know, the combination of mental health, brain injury and belief and other times when you want to have good conversations with people in person*.

Although video conferencing provided a means of staying connected during the pandemic, face to face communication was still the favoured method as more in depth, personal and intimate conversations and topics could be discussed.

*There’s* *so much more communication that goes on in person than it does on a screen.*

#### Loneliness after lockdown

##### Left out and left behind

When asked about the prospect of lockdown ending, participants were less positive. Whilst acknowledging that the pandemic had created a level playing field, participants were sceptical that this would last once restrictions were lifted. Survivors reported feelings of frustration and disappointment regarding this short-lived acceptance of their situations.

*there’s* *been a lot of discussion around the empathy* that’s *been generated through COVID that people have an insight into what it feels like to be socially isolated, to not be able to join in. But I think human nature leaves people to feel excited about the option of that ending. If I had the option for my brain injury to end, I think* I’d *be overjoyed, but I* don’t *so those challenges around social engagement for myself will remain.*

##### Return to normal is not an option

Participants displayed frustration and sometimes anger about lockdown measures lifting and societies perceived return to normality with lockdown measures lifting, highlighting their own inability to ‘return to normal’ after suffering their brain injury and the feelings of loneliness that this generates.

*you go back to normal, but* *I’m* *still in the same boat and* *I’m* *going back to a place where I feel different, I feel alone.*

One participant used the analogy of catching a train:

*when you used to go and catch the train, and the* *train’s* *been delayed, and you all stood there on platform together, you know, all not able to get on the train or feeling grumpy, and stuff, or whatever, and then train comes along, and everyone else can get on it and go off on their life journey. And* *you’re* *the person who has to stay stuck on the platform, you cannot get on that train, and carry on that sort of social engagement. And it, it does feel that the return to normal is not an option for people with a brain injury. And I guess that also then that brings that psychological impact, that is the one that I probably struggle with the most, which is that brain injury* *doesn’t* *get better*.

This analogy describes both the social aspect of being left behind but also touches on the psychological impact that brain injury survivors feel in having to adapt to a ‘new normal’. This adaptation is a constant process for the survivors we spoke to.

### Feeling ‘outside of society’

The invisibility of brain injury is well documented, and this is an aspect that was often repeated by our participants. The majority of our respondents did not display visible signs of an injury, rather they described the cognitive and emotional consequences of their brain injury as invisible to others. Participants found this extremely difficult when engaging with family, friends and those they knew prior to the brain injury, citing a lack of understanding of who they now are contributing to a lack of connection.

*You know, they just* *can’t* *fathom that actually*, *I’m* *not who I was prior the brain injury, and that I* *can’t* *just live a life like they do.*

Brain injury has often been referred to as an invisible disability, a sentiment corroborated among our respondents. Participants suggested that with a more visible disability, expectations would reduce, understanding would be increased and brain injury would generally be treated with more patience and sensitivity. Instead, participants described living with daily pressure and expectations from society due to ‘an undercurrent of we look okay’, with participants expressing anxiety over returning to these feelings post-pandemic. This further generates a barrier to authentic connection between brain injury survivors and society, leaving those with a brain injury ‘feeling outside of society’, ‘vulnerable’ and like ‘it’s every man for themselves’.

## Discussion

Although loneliness and mental wellbeing are inextricably linked, even for the general population (Beutel et al., 2017), it is surprising that for our brain injury survivor’s lockdown loneliness had no further significant effect on their mental health. The qualitative data lends support to the notion that brain injury survivors could already be at a ceiling for loneliness, due to the ‘imposed aloneness’ survivors feel as a direct result of their injury (Diekema, 1992; Yang, 2019) whereby individuals feel powerless or hopeless. Survivors often referred to the word ‘alone’ when defining loneliness, conflating aloneness and loneliness. Being alone is not always a prerequisite for loneliness; some may choose to be alone and others, including some of our participants, may be lonely when in the company of others. When alone, healthy adults can minimise the likely transition from aloneness into loneliness. However, brain injury survivors are conscious of the fact that their abilities to prevent being alone from developing into loneliness have been seriously compromised and that lockdown did not further impact upon this.

The qualitative data support this theory, but this was not reflected in participants loneliness measured by the DJG (*M* = 3.60, SD = 1.13) which would not indicate such a ceiling effect. Whilst measures of loneliness are useful for capturing some ‘universal’ features of loneliness, there have previously been questions arising with how well they relate to the experiences of non-neurotypical populations (Yang et al., 2022). Therefore, our survivors’ phenomenological experiences of loneliness may differ to their quantification of these experiences. It is important that loneliness measures are reliable and valid across different contexts and populations, and the data presented here suggests that current measures of loneliness do not relate to the specific experiences of stroke survivors. Future research may benefit from including a larger data set linking qualitative and quantitative responses in non-neurotypical populations to further differentiate and understand these effects.

An additional possibility to consider is the important role of resilience to the mental health of our respondents. Our quantitative data indicates a positive effect of resilience on psychological wellbeing. This finding is consistent with previous literature demonstrating the relationship between these two factors across a range of situations (Bano and Pervaiz, 2020; Kelifa et al., 2021). Brain injury has an enormous impact on an individual’s life in many different domains, and rehabilitation plays an important role within the process of coming to terms with the disability (Sigurdardottir et al., 2014). It may be the case that brain injury survivors’ adaptation to life changing circumstances post-brain injury has made them more resilient to the changes created by the pandemic, explaining the lack of a consistent, significant effect of loneliness on our survivor’s mental wellbeing. The positive experiences that many of our participants recounted when discussing lockdown were unexpected and contrast with the mental distress, anxiety, depression and stress experienced by healthy adults during this period (Daly et al., 2022; Pierce et al., 2020). Sustaining a brain injury and the subsequent experiences may have buffered survivors to the social isolation and loneliness experienced by the general population during lockdown.

The restricted living experienced by the general population during lockdown was more parsimonious with the brain injury survivor’s lives pre-pandemic. In this way, lockdown generated a ‘level playing field’ that had a curious effect with our survivors qualitatively reporting increased wellbeing, reduced stress and overall better quality of life. Indeed, this provides an insight into the importance of the patient mindset and the role the perceived expectations of others have on mental wellbeing. Brain injury survivors’ perceptions of societal expectations can lead to identity loss and additional stress (Walsh et al., 2015) with survivors feeling both physically and developmentally behind their peers after a brain injury (Lowe et al., 2021). Lockdown removed this pressure to conform and provided survivors with time to reflect and a ‘safe space’ to focus on themselves, absolved of perceived societal expectations.

It is important to focus on bolstering these positive factors among brain injury survivor’s post-pandemic, to boost resilience to loneliness, protect mental health moving forward and negate the health inequalities experienced by this population. Pausing life for everyone, much like it felt occurred during lockdown, is not feasible. However, reconstructing survivors’ identity in society and changing their beliefs around societal expectations of their capabilities will have positive benefits. This is of particular importance post-pandemic because although our respondents felt a greater awareness of their situation by the general public, they were already apprehensive and anxious about being ‘left behind’ again (akin to the post-injury period) as life returned to pre-lockdown norms.

These findings indicate that patients may benefit from adjustment of expectations, through psychotherapy for example. Post brain injury the focus of rehabilitation is often centred on the cognitive and physical symptoms experienced by survivors, with the emotional consequences of brain injury receiving little attention historically. However, psychotherapy such as CBT, has been shown to be effective for improving emotional distress among brain injury survivors (Bradbury et al., 2008). Our findings indicate that utilising this type of psychotherapy to build resilience and address survivors’ perceived societal expectation is an important area to focus within therapeutic practice in this population.

The delivery pathway of any intervention in this group is important to define. Brain injury survivors’ unique profile as individuals living with a multi-morbidity disorder, coupled with the issues identified above places them in a more disadvantaged position as a group of individuals with a higher rate of identified barriers to seeking help, further increasing their likelihood for isolation generally. Survivors in this study tended to believe that instead of seeking help, they should wait for it to be offered. This exacerbated loneliness, as did their tendency to question the genuineness and authenticity of any help received in line with previous findings (Corrigan et al., 2014). Peer support, defined as being composed of individuals who share a similar problem and come together to provide mutual help and support (Adamsen, 2002) provides a space to self-disclose, a unique sense of community, the opportunity to be accepted and the opportunity to share information (Evans, 2011; Ussher et al., 2006). With the associated changes in personal identity and the stigma and help-seeking issues identified by our respondents, the importance of peer support groups to this population is unsurprising. Peer support groups could also serve as a vehicle through which societal expectations are managed; engaging with individuals with similar lived experiences and functioning could help to moderate survivors’ expectations of their capabilities and their identity in society. Granting opportunities for socialising and social network expansion (Castelein et al., 2008), peer support groups provide a ready-made method or coping strategy for dealing with loneliness for many of the participants we interviewed. For those survivors living in rural settings or those with limited access to online technologies, peer support networks are also an excellent method of providing informational and emotional support (Harrison et al., 2017). To maximise the effectiveness and uptake of such groups, they should be made as accessible as possible and actively promoted thereby being viewed as support offered rather than sought.

However, accessibility and the use of technology need to be balanced when thinking about support delivery. Throughout our interviews, participants discussed the importance of video conferencing technology in maintaining social contact throughout the pandemic. Numerous studies highlight the unique difficulties facing individuals with invisible disabilities (Gilworth et al., 2008) but our participants reported how video conferencing had assuaged anxiety and awareness of these deficits. Although participants’ responses highlight the effectiveness of video conferencing, there was still an overriding preference for face-to-face contact where possible. Participants reported that personal conversations can be restrictive when only communicating via online tools, consistent with previous research highlighting the personal connection (Seitz, 2016) and rapport that are only built through face-to-face engagement (Vogl, 2013). Additionally, considering common issues experienced by brain injury survivors, such as headaches, fatigue and cognitive difficulties, many survivors find it challenging to navigate technology or focus on screens for sustained periods of time (Lindén et al., 2010). Therefore, even with the establishment of these adapted support systems to combat loneliness for many survivors, engagement would be difficult for others and loneliness would potentially remain an issue as would an exacerbation of feelings of ineptitude. Due to the range of impairments that survivors can experience and additional factors such as access to technology and geographical location, a combination of face-to-face and technological solutions need to be considered.

The preference for face-to-face social contact is important considering the methodologies employed in the current study. Face-to-face methodologies are often perceived as the gold standard of qualitative research (Novick, 2008). Conducting our interviews via Microsoft Teams removed restrictions often associated with face-to-face interviews such as travel time, expenses and participants distance from the research site. For this study, participants with an internet connection were recruited from across England and Scotland, permitting a much more diverse sample to be achieved than recruiting based on regional proximity to the researchers. However, participants’ views in this regard are an important consideration. Although a necessary and justifiable method for conducting this type of research considering the lack of options for face-to-face research during the pandemic, it should therefore be considered that utilising face-to-face methodologies instead may have produced different conversation points.

Our recruitment strategy for this project was to create a participant pool representative of brain injury generally, rather than one facet of brain injury (e.g. age, gender, specific comorbidity, time since injury, etc). As such, the data in this project provides a holistic representation of the views of brain injury survivors rather than a specific sub-group within this population. However, participants were recruited from previous research associations with the authors, and some were regularly involved with local charities and external support groups. Therefore, the views and circumstances of our participants may differ from those brain injury survivors who, for example, did not belong to any association. This is important to address when considering the positive discussions with respondents on the role of peer support for alleviating loneliness, combatting low mood and improving general wellbeing. Additionally, all participants had internet access and were fluent English speakers, common with 93% (Statista, 2023) and 98% (Office for National Statistics, 2021) of dwellers in the UK respectively. Future research projects should focus on individual facets of brain injury to build an understanding of the specific differences that exist in experiences within these sub-groups. Through a more granular analysis, the differences in survivors’ views and experiences can be addressed through the lens of socioeconomic strata, race and culture.

## Conclusions

This study provides a unique and novel account of the experiences of loneliness for individuals with brain injury during the pandemic. Interestingly, survivors highlighted the break from perceived societal expectations that lockdown afforded, permitting them time and space to focus on themselves. Additionally, our data has reinforced the important role of resilience in survivor wellbeing, with post-brain injury adaptation combatting negative experiences of lockdown. We recommend that psychotherapy, such as CBT, focus on reframing survivors’ beliefs regarding societal expectations, minimising the pressure experienced by survivors to keep up with their peers physically and emotionally and modulate resilience. Furthermore, all participants identified the importance of peer support, whether those respondents had direct access to it or not. We recommend incorporating accessible and promoted peer support into future interventions to help moderate survivors’ expectations of their capabilities and identity in society, reduce loneliness, protect mental health and generally improve wellbeing for brain injury survivors.

## Research Data

sj-csv-2-hpq-10.1177_13591053231166263 – Supplemental material for Uncovering the social determinants of brain injury rehabilitation Click here for additional data file. sj-csv-2-hpq-10.1177_13591053231166263 for Uncovering the social determinants of brain injury rehabilitation by Stephen Dunne, Glenn P Williams, Chloe Bradbury, Tamsin Keyes, Alison R Lane, Keming Yang and Amanda Ellison in Journal of Health Psychology This article is distributed under the terms of the Creative Commons Attribution 4.0 License ( http://www.creativecommons.org/licenses/by/4.0/) which permits any use, reproduction and distribution of the work without further permission provided the original work is attributed as specified on the SAGE and Open Access pages ( https://us.sagepub.com/en-us/nam/open-access-at-sage).

sj-docx-5-hpq-10.1177_13591053231166263 – Supplemental material for Uncovering the social determinants of brain injury rehabilitation Click here for additional data file. Supplemental material, sj-docx-5-hpq-10.1177_13591053231166263 for Uncovering the social determinants of brain injury rehabilitation by Stephen Dunne, Glenn P Williams, Chloe Bradbury, Tamsin Keyes, Alison R Lane, Keming Yang and Amanda Ellison in Journal of Health Psychology

sj-jpg-4-hpq-10.1177_13591053231166263 – Supplemental material for Uncovering the social determinants of brain injury rehabilitation Click here for additional data file. Supplemental material, sj-jpg-4-hpq-10.1177_13591053231166263 for Uncovering the social determinants of brain injury rehabilitation by Stephen Dunne, Glenn P Williams, Chloe Bradbury, Tamsin Keyes, Alison R Lane, Keming Yang and Amanda Ellison in Journal of Health Psychology

sj-pdf-1-hpq-10.1177_13591053231166263 – Supplemental material for Uncovering the social determinants of brain injury rehabilitation Click here for additional data file. sj-pdf-1-hpq-10.1177_13591053231166263 for Uncovering the social determinants of brain injury rehabilitation by Stephen Dunne, Glenn P Williams, Chloe Bradbury, Tamsin Keyes, Alison R Lane, Keming Yang and Amanda Ellison in Journal of Health Psychology This article is distributed under the terms of the Creative Commons Attribution 4.0 License ( http://www.creativecommons.org/licenses/by/4.0/) which permits any use, reproduction and distribution of the work without further permission provided the original work is attributed as specified on the SAGE and Open Access pages ( https://us.sagepub.com/en-us/nam/open-access-at-sage).

sj-pdf-3-hpq-10.1177_13591053231166263 – Supplemental material for Uncovering the social determinants of brain injury rehabilitation Click here for additional data file. sj-pdf-3-hpq-10.1177_13591053231166263 for Uncovering the social determinants of brain injury rehabilitation by Stephen Dunne, Glenn P Williams, Chloe Bradbury, Tamsin Keyes, Alison R Lane, Keming Yang and Amanda Ellison in Journal of Health Psychology This article is distributed under the terms of the Creative Commons Attribution 4.0 License ( http://www.creativecommons.org/licenses/by/4.0/) which permits any use, reproduction and distribution of the work without further permission provided the original work is attributed as specified on the SAGE and Open Access pages ( https://us.sagepub.com/en-us/nam/open-access-at-sage).

## Notes

1. As DJG has a minimal effect on predicting WEMWBS, two alternative model specifications with DJG treated as a monotonic predictor (*Δ_elpd_* = 0.34) or excluded from the model (*Δ_elpd_* = 0.482) performed similarly well. For brevity, here we report the model with the lowest $\hat{elpd}$ and highest weight.
