# Supplementary material for: Uncovering the social determinants of brain injury rehabilitation
Source: J Health Psychol. 2023 Apr 7;28(10):956–69. doi: 10.1177/13591053231166263 (PMC10466963; doi:10.1177/13591053231166263)
Supplement: sj-docx-5-hpq-10.1177_13591053231166263 – Supplemental material for Uncovering the social determinants of brain injury rehabilitation [file sj-docx-5-hpq-10.1177_13591053231166263.docx]

Table 1. Participant demographics

| ID | Gender | Age | Marital Status | Employment | Severity | Cause of injury | Year of injury | Impairment | | | | | | |
| --- | --- | --- | --- | --- | --- | --- | --- | --- | --- | --- | --- | --- | --- | --- |
|  |  |  |  |  |  |  |  | Vision | | Speech | Motor | Memory | Cognitive | Pain |
| 01 | Male | 54 | Married | Medically Retired | Mild | Brain Tumour | 2015 | No | Yes | | No | No | Yes | No |
| 02 | Male | 54 | Married | Medically Retired | Severe | Bike Accident | 2018 | No | Yes | | Yes | No | Yes | No |
| 03 | Female | 37 | Married | Sick Leave | Mild | Sporting Injury | 2016 | Yes | No | | Yes | No | No | Yes |
| 04 | Male | 51 | Single | Unemployed | Moderate | Assault | 1998 | No | No | | No | No | No | No |
| 05 | Male | 52 | Married | Medically Retired | Severe | Stroke | 2013 | Yes | No | | No | Yes | Yes | No |
| 06 | Male | 39 | Married | Medically Retired | Very Severe | Road Accident | 2017 | Yes | Yes | | Yes | No | No | No |
| 07 | Male | 47 | Married | Unemployed | Moderate | Encephalitis | 2020 | No | No | | Yes | Yes | No | No |
| 08 | Male | 53 | In a relationship | Medically Retired | Mild | Brain Haemorrhage | 1992 | No | No | | No | No | Yes | No |
| 09 | Male | 68 | Widowed | Retired | Mild | Stroke | 2016 | Yes | Yes | | Yes | Yes | No | No |
| 10 | Male | 50 | In a relationship | FT Employment | Severe | Fall | 2000 | No | Yes | | Yes | Yes | Yes | No |
| 11 | Male | 46 | Single | Unemployed | Mild | Encephalitis | 2019 | No | Yes | | Yes | No | No | No |
| 12 | Male | 55 | Married | Medically Retired | Severe | Bike Accident | 2017 | No | Yes | | Yes | Yes | Yes | No |
| 13 | Female | 53 | Single | FT Employment | Mild | Road Accident | 2018 | No | No | | No | Yes | Yes | No |
| 14 | Female | 49 | Single | Unemployed | Mild | Brain Tumour | 2004 | No | No | | No | Yes | Yes | No |
| 15^a^ | - | - | - | - | - | - | - | - | - | | - | - | - | - |
| 16 | Female | 56 | Married | PT Employment | Severe | Road Accident | 2009 | Yes | No | | No | Yes | Yes | No |
| 17 | Male | 53 | Single | Volunteer | Mild | Assault | 2001 | No | No | | No | No | Yes | No |
| 18 | Female | 28 | Single | FT Employment | Mild | Fall | 2018 | No | Yes | | No | Yes | Yes | No |
| 19 | Female | 50 | Married | Medically Retired | Mild | Stroke | 2019 | No | No | | Yes | No | No | No |
| 20 | Male | 47 | In a relationship | Unemployed | Moderate | Assault | 1993 | Yes | No | | No | Yes | Yes | No |
| 21 | Female | 24 | Single | Unemployed | Severe | Road Accident | 2012 | Yes | Yes | | Yes | Yes | No | Yes |
| 22 | Male | 68 | Married | Retired | Moderate | Fall | 2016 | No | Yes | | Yes | Yes | Yes | No |
| 23 | Female | 33 | Married | Student | Mild | Fall | 2017 | Yes | No | | Yes | Yes | Yes | No |
| 24 | Male | 39 | In a relationship | Unemployed | Severe | Bike Accident | 2009 | Yes | Yes | | No | No | Yes | No |
| 25 | Male | 64 | Single | Unemployed | Severe | Road Accident | 1978 | Yes | No | | No | Yes | Yes | Yes |

^a.^ Participant 15 was excluded from the study as they did not currently live in the UK.
