# Supplementary figures and images for: Uncovering the social determinants of brain injury rehabilitation
Source: J Health Psychol. 2023 Apr 7;28(10):956–69. doi: 10.1177/13591053231166263 (PMC10466963; doi:10.1177/13591053231166263)

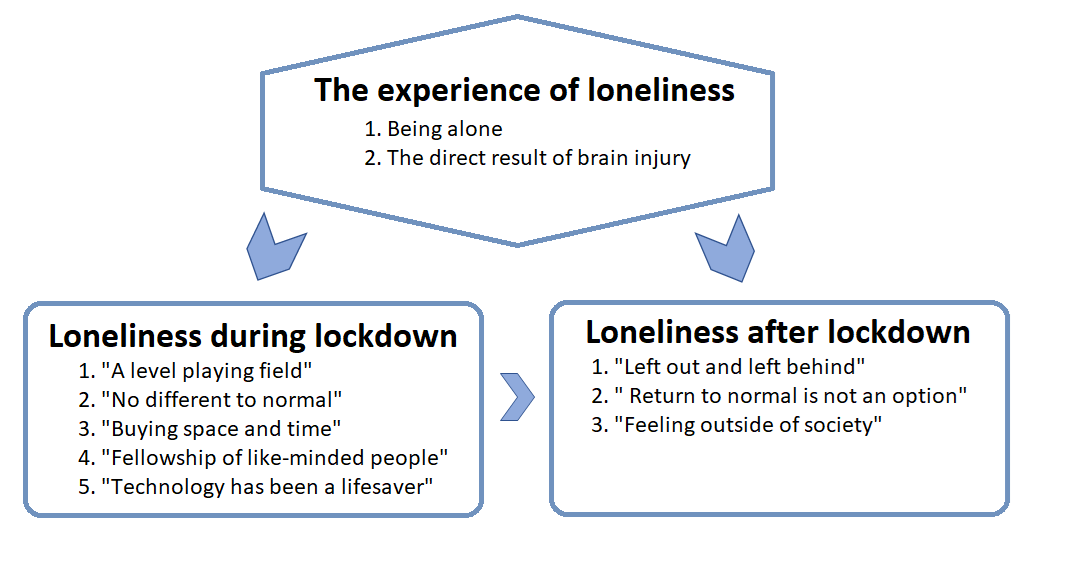

Supplement: sj-jpg-4-hpq-10.1177_13591053231166263 – Supplemental material for Uncovering the social determinants of brain injury rehabilitation [file sj-jpg-4-hpq-10.1177_13591053231166263.jpg]
