# Supplementary material for: Uncovering the social determinants of brain injury rehabilitation
Source: J Health Psychol. 2023 Apr 7;28(10):956–69. doi: 10.1177/13591053231166263 (PMC10466963; doi:10.1177/13591053231166263)
Supplement: sj-pdf-1-hpq-10.1177_13591053231166263 – Supplemental material for Uncovering the social determinants of brain injury rehabilitation [file sj-pdf-1-hpq-10.1177_13591053231166263.pdf]

Compiled Transcripts

Interviewer(s): **INT1:SD**

INT2:CB

Respondent(s): RES (P1)

INT1 **So, you've already started talking about erm, yaknow your particular brain injury, and and, you started talking about the reasons why you do this project in particular, but can you tell me more about, yaknow, your brain injury how it happened, erm, what was affected, these kind of things?**

RES Well I, I, I separated from the first wife 2000 an an ee ee leven and straight away I started to get really bad m m migraines which I thought were down to the stress of, of (unclear, 00:04:55-00.05:00), for four five months they were diagnosed as cluster headaches errm, you froze on me again which is fine

INT1 **Yeah (unclear, 00.05:09)**

RES A a a and then I , after about four, five months of really bad cluster headaches which means my left, my left hand side would get tingly first, I ,I, I'd then go to bed and and spent three of four days in bed with a cluster headaches, which is so intense that I would lay there in the dark with just the radio on to keep sane because (unclear , 00.05.35) , SD

INT1 **Yeah**

RES Uhh, I then, I then uhh m met this lady, REDACTED, I met her in uhh what must have been June 2, 2 uhh, 2 ,2013, we, we met through dancing,

INT1 **Right**

RES I'd gone from playing rug, I'd gone from playing rugby to bloody dancing

INT1 **Yeah (laughs)**

RES (chuckles) yeah I know, madness We uhh met sh uhh we met on the dance floor, she she fell in love with me pretty quick,

INT1 **Yep**

RES obviously (chuckles) uhh, we then started dating and I moved in with her within part of November 2013, and then 2014 the headaches were quite …regularly , every ser, every six or seven weeks erm, must have been November , no must have been August/ July time 2015 I went to me GP who sent me for an MRI scan and then eventually I got told there was a six or seven cm brain tumour there, so uhh. At that point I thought okay fine, so uhh I had the surgery done for, for the third time because, because the first two times it got cancelled, the second times it got cancelled after being all day in and (unclear, 00:06:40)

INT1 **Yep**

RES So uhh yeah, that was quite pain, uh, that was quite bad. Then I had the surgery and then November the 6th 2015, uhh , as as I said via the email google (unclear, 00:06:52) brain injury, you'll see loads of stuff on it because I, I give talks about living life with a brain… injury, uhh I had the surgery on the Friday , 57 stitches, I was discharged on a Sunday cause I

, because I asked them if if I could go home , cause I , cause I needed rest and a week after surgery I was in a pub having a beer!

INT1 **(Laughs)**

INT2 ***(Laughs)***

RES Seriously, seriously (laughs). However, thee thee one time I left (unclear, 00:07:21) in in a wheelchair, my left-hand side was completely numb, my my po, my my my partner then who's now the wife had to feed… feed me, dress me. Uhh I couldn't walk more, more than about 15 meters w without uhh, without n n needing a rest. She had to shower me and I know I was about 6 weeks post op back in the gym, I was I was l l level 1 on a push bike after fifteen seconds my body just went erghh. Went to do some erm weights and my body went erghh, I stood there and I, ya know I'm a big lump, stood there in the REDACTED Lloyd or the Virgin Active there I just burst into tears.

INT1 **yep**

RES …I was having m m meetings with with the doctor she then advised only yoga and Pilates classes, so I started doing them and was totally crap, but then, now over the past couple of years I I'm now back doing the weights twice a week, and I play league tennis so uh

INT1 **Oh amazing!**

RES Uh I had 22 months of neu, neu, neurology rehab at erm, (unclear, 00:08:23) House, which is erm.. which is a neu, neu, neurology department in Norwich. I had erm 23 months I had o, o O team meetings and, and clinical psy psychologists, so so CB, you're doing a FANTASTIC JOB!

INT1 **(Laughs)**

INT2 ***(Laughs)***

RES You really are, seriously, seriously if it hadn't of been for my w w weekly meetings with Dr Dr Allison Woods, I'd have probably topped meself a long time ago because erm …she was great, I then attended Headway for about 3 or 4 years, just to get use and now I understand how how, yaknow, how my brain works and and , yaknow how to manage my injury. But because I'm , yaknow I I'm yaknow I'm biggish lump in in decent shape people go 'You got a brain injury!?' and I'm like yeah, because I look normal.

INT1 **Yeah**

RES So uhh, uhh yaknow, so so, so that's why I give talks about living life with a brain injury because yaknow I'm loud, bubbly most of the time b b b yaknow, but uhh, b b but people don't see me on a bad day with a brain which is that tired yaknow, yaknow, for for me to get out of bed that day is enough.

INT1 **Yeah**

RES yaknow, it's a complex thing

INT1 **Yeah, definitely, yeah, erm there's a lot you've mentioned in there that I will come back around to, cause erm, I think it's really interesting to talk about. Erm, in terms of, cause, because you were talking about your rehabilitations specifically there,**

RES Yeah

INT1 Is there anything that you found was really helpful in your rehabilitation, or anything you found was more of a hinderance, or, or a problem?

RES OHH…, iiit's when your uhh (unclear, 00:10:01) because another thing as well was after being in the Headway for about ..3 months, I I sat there on a Monday morning doing cognitive stuff there and I couldn't, add up 2 + 2 and I just cried because I thought God, yaknow b but then theres yaknow, the brain ermm, rehab, rehab is about developing a close professional re re relationship with your medical experts, erm, and I'm the type of person who who if you tell me straight that's fine, but the way,b but that takes time because, yaknow, when you first have a meeting with a health professional, it takes time just to delve deep inside yourself b because you are exploring stuff, yaknow, stuff which has been hidden, hidden for donkeys years. But its about just yaknow, if you like…a brutal c conversation and also then taking on board what the experts say, put, putting it into every day stuff, and then ma managing it and it's the most out of.. lived in process which takes time and it takes honesty and a lot of people, they can't do it because a lot of people , this is where, as a former rugby player and rugby coach it it's ma management, and some players need an arm and a shoulder and some players need a kick up the arse. Mo, most

INT1 (Chuckles)

INT 2 (Chuckles)

RES (Laughs) it's true though isn't it

INT1 Yeah

RES most people just want, yaknow, want a middle ground but yaknow, ha ha having seen having seen my mum, yaknow have two brain surgeries and her life was basically..housebound.. all she done, all she done was watch tele, smoke, I thought no, I want a decent quality of life,yaknow. Working with professionals, asking awkward questions and and just going so deep inside yourself, that only then then can you sort of manage it but that takes… years.

INT2 Yep

INT1 yeah, that was a really good answer

RES You've, you've frozen on me again (laughs)

INT1 Are we?

RES Yeah! Yeah (unclear, 00:12:05)

INT1 Oh that's so frus, s s sorry about that I am, on my screen I am moving, my internet is, is absolutely, has been absolutely terrible these past couple of days so hopefully it will kick back in shortly.

RES No that's fine, yeah y y y , I mean but, I mean and that's why yaknow I, I'm really pleased to have this chat b y yaknow, because I do bits and pieces with Headway, I've done research some research stuff before and however bouts you put it onto yaknow (unclear, 00:12:32), you don't get the erm proper.. emotion

INT1 **Yep**

RES Of a, yaknow of of a person who you're talking to, people skills and things like that

INT1 **Definitely, yep, no it's obviously really good to actually be able to speak to you about this type of thing**

RES thank you,and you too

INT1 **In terms of, one thing I want to delve in a little bit more deeper into I think is this idea of , well , a better understanding of the support network around you. So, erm, you, you spoke a bit about erm, is it your current partner?**

RES Wife, yeah, yeah

INT1 **Current wife, sorry so**

RES Current wife yeah, yeah we got married yeah

INT1 **Yeah, oh perfect congratulations, erm so can you, can you tell me about, if you think about over the course of the pandemic specifically over the past year,**

RES Yeah

INT1 **Can you tell me about the the kind of people who have helped you, or in your life who's been around you, erm or who you've even seen.**

RES Right, over the past year, erm.. I've, yeah, there's obviously t there's obviously the wife who works from home couple days a week, that's been different. Erm, m my parents are both dead, her parents, my outlaws, are both alive, he's 82 (laughs), she's 77, they're both really fit an and, yaknow when the lockdown last march time, my wife said to them right what's instore, they said no, you can still come round as and when. So I go and see them, probably at at least once a week, if not twice a week because….well yaknow whatever (unclear 00:14:03) they're my support bubble, I'm classed as disabled, they're classed as old and vulnerable, or what ever they're. But, we're all grownups, we're yaknow a a ,yaknow a a and I said (unclear, 00:14:14). So I see them regularly, 2 close friends , male friends my age from the tennis club

INT1 **YEP**

RES Yaknow, we've all kept in tough at least once a fortnight, zoom, yaknow yaknow , zoom catch up normally after with a few beers and and also weekly chats and whatsapp messages so, erm because, because the latest word is it, is it social isolation, does that ring a bell?

INT2 ***Yep***

INT1 **Yeah absolutely, yeah**

RES Well well well people people with a brain injury (unclear, 00:14:52). I mean, we've had this all the time and its still still ongoing, but erm me me and my 2 b both male friends who I play tennis with, we actually made an effort just to guys talk and mens feelings, so , we got through it (laughs) we got through it.

INT1 **Were you having those types of conversations before the pandemic or is that something that has only happened over the past year?**

RES Well…we'll have, well, we'll have sort of general feelings but but no where near, no where near as deep because men don't talk about our feelings we're all tattooed, ya know, chest (unclear 00:15:30), so ,but, it was so it was so it was so bloody nice to to hear that everyone was feeling feeling feeling the same way.

INT1 Yep

RES So uhh, yeah, yeah so, yeah.

INT1 So , erm CB **did you have anything you wanted to add onto that?**

INT2 I just wanted to ask, you mentioned that you'd been really quite sociable through the pandemic, is that different to before the pandemic? Has it dropped at all or increased or is it just changed in the fact its more online now?

RES (Sighs), well when… well it's been, I mean I mean it's difficult really because I I'm really pleased now at the fact that g y y yaknow, gyms are open again because I I I'm back there couple times a week, I'm back there playing tennis. Before, before the erm, the erm pan pandemic though, I I was also a patient research ambassador for the n erm n Norfolk community healthcare trust, which means I get invited along to meetings, join in bits and pieces there, that over the past year has, has, has has been done through zoom. Now zoom for some people is great, b because if you're working it's great, you can have your put, you put on a decent top and then stand around with s s shorts on, you can work from home you y don't have to travel blah blah blah that's great, but for me, errm erm erm the erm stuff there didn't work be because my my my concentration span would just wander off into air, I got nothing from it so I now, I stopped them so uhh, yeah, uhh nother thing as well is I I play the guitar badly, I have uhh since uhh since the uhh (unclear, 00:17:21)last may time , before that for about six months I'd I'd I'd have a guitar lesson once a fortnight since last, since last m March is now once a week,errm, when we're allowed to do… it in the flesh we can, apart from that it's all done under zoom, which obviously, with the delays in wifi things can get things can get held out. So uhh ,but it's been bloody hard, yaknow, really hard.

INT1 So w what it feels like is that you and you can correct me if I'm wrong here tell me if I'm wrong, but it feels like you were quite a sociable individual prior to the pandemic, you had these certain activities that you would do but actually, what you've managed to do over the past year is actually adapt and switch them to maybe online?

RES …I've adapted because I've had to but then ,I, yaknow, I feel very resentful. Because y yaknow, after what, after what I've gone through with seeing Mum, if you like be be incontinent through her brain surgery, I was determined not to do that and touch wood I wasn't and then having worked e extremely hard through headway and and and and yaknow, and the new neuology erm department there. I just wanted to join for simple things in life, which is going to the gym, do the weights play tennis three, four times a week and the freedom took about t took about two hours to go out to to the a pub and have a few beers and take the wife out. Does that make sense?

INT1 Absolutely

RES Yeah, I mean I mean the everyday things has been, yaknow, has been taken away from me and for you guys and holiday. So

INT1 Yeah

INT2 ***can I just ask you, you've talked quite a lot about sport and erm, your tennis and going to the gym. How have you managed through the pandemic when you haven't had access to that?***

RES that that CB is a fantastic question. L l l last year, when when the first lockdown, uhh now bear bear bear in mind the government said three weeks to flatten the curve. I thought, yaknow, everyone thought I thought great three, three weeks off there, fine, I'll just chill out did some pieces there. Then after about a month of that the old weight piles on it's like, I got to do something I've got some erm dumbbells back home. Yaknow, yaknow which is okay. I I had a pushbike so so I started cycling, the wife starts.. so both our bikes there there were about 18 years old so last may we actually (unclear, 00:21:01)but… when most people most people weren’t working, we were actually saving a fair bit of money every month so what we done was last may we went out bought and bought bought a brand new bike each, and and then cycle most days there so, so I actually lost weight, uhh (unclear, 00:20:21) there, then obviously Christmas Christmas time there, December Christmas time fine. January, February March is cold where you can't be arsed (laughs), you turn, you turn on the news it’s all doom and gloom is all contradiction now, uhh so I've struggled (laughs) because erm, because you know, I’ve lost motivation, but now I'm thanking the gym back playing tennis and I know the extra stone, stone which I’ve put on will, will come off because because for me, a light brow sport doesn't worth well, living, if that makes sense. I'm a former b erm b boxer, played for fun fun rugby until I was I was 47. So uh as you know, without doing sport, oh dear.

INT1 **And this was something so one thing I wanted to touch on,uhh that you said previously was obviously your.. if you think not..don't think about the pandemic now, but we just think about the brain**

RES yeah

INT1 **in the brain injury itself**

RES yeah

INT1 **So when you, you said you said obviously used to play rugby**

RES yeah

INT1 **erm, and obviously then you, uhh, you obviously couldn’t play that anymore because of the brain injury.**

RES Well well, I I I could do anything I did, I I could do anything. I I went to a couple of erm I I told the wife b b because my my son plays as well cause I I I coached his team as well so he plays, a and that must have been about 2016/2017 I went along to a couple of pr preseason preseason coaching s sessions, which were following contact that I told her it was, you know, touch rugby, it’s only my gobby son, there's a sliver that are slim, and she made mad she went mad. So I thought ohh I cannot do that anymore. So were, (laughs) so yeah.

INT1 **it seems like you've managed to uhh, uh adapt in terms of like the some of the just literally some of the activities you've already mentioned in this conversation, things like playing tennis now in a league and dancing and going to the gym. So these are all, so obviously there's a lot of, there's a lot to be said about, erm, you and your enjoyment of sports sporting activities.**

RES M m m most definitely because because through, through, through sport it's a sense of purpose yaknow a a and the friendships which you get a are there for a long long time plus, plus the the REDACTED Lloyd where where I train is literally round the corner, I can walk there in in in seven to eight… oh, yeah. You know, I've got to do sport. I've got to do sport, you know, yaknow

INT1 Perfect.

RES Thank you.

INT1 Erm, CB **did you have anything to add for move on to the next section?**

INT2 No, I think that we've got everything there.

INT1 Okay, cool.

INT2 Thank you.

INT1 **So, just I'm going to change tact a little bit now…erm** REDACTED**, I'm just going to ask you more about mainly sort of things around social isolation and loneliness if that's okay, the first thing, the first thing I kind of want to know, is how you would define the term loneliness?**

RES …Oh how how would I, how would I define it tonigh, well I well I would say a few things. One, one, there there's a massive difference between being alone and been lonely. If you're alone, then it's your your choice to be alone. B but I think the term loneliness means means a person is forced to be alone and forced to not have contact for any any other member of life, uhh loneliness, yeah, it can be a killer.

INT1 Umhm, is it, is it something to you with you? It is something that you felt previously?

RES No, no, because I'm I'm I'm quite confident in my own skin. A a and I'd like my own company, if that makes sense, uhh because I,because I'm you know, I'm at peace with myself and that's what, and that's why when, yaknow when the wife works from home, Tuesdays and Thursdays she's there, uhh kitchen and I have been less than quiet, well I'm quite a quite a big lump (interviewers chuckle) there, there is music playing so so she's there on laptop quarter past nine in the morning. I come waddling through, stark bollock naked to make a cup of tea. She's like, ahh for fack sake, I'm like, you don't get this. And the other, she's like, I'm at work, I'm like, REDACTED, we've had this the past year. Your works along, we're still at home, do you want a brew, she's like, yeah, b but does that make sense there? So? Yeah. Y yaknow, prior to the original question, loneliness. Oh, oh yeah, I mean, I mean, I mean, to me,uhh Prince Philip's funeral other weekend we know it was a national tragedy, blah, blah, blah. He was ninety nine years old he is , but for me the thing which, which, which I can't fathom, is we all saw, we all saw the Queen there in his service by herself. That's just wrong, that to me is is loneliness because there she is, she's there in the church service for what, 45 minutes playing the game, when, when, yaknow no mention of b Buckingham palace, there says she's got her her maid of honour, her grandkids round her, and the fact that no one had the balls to, sit to sit beside her, because they because they knew again, (unclear,00:26:19), you're fine. To me. It's just wrong.

INT1 In terms of your feelings of loneliness Mike

RES yeah

INT1 **so over the past year, have those feelings changed at all? Or have you? Do you feel that you haven't felt lonely at all? Over the past year?**

RES I, I,no, I personally I personally I would say it's been more.. more frustration, rather than loneliness because we've all got TVs ,WIFI, telephone, yaknow, oh, I've got WiFi telephone calls there online, you can go on there. But you know, that for me. It'sss more a signal of frustration rather than loneliness. We've got a dog so so so so so, you know, so so so the bloody dog now goes out more, more more than what we did.

INT1 **(laughs) Yeah, erm,in terms of, in terms of the dog,erm.. do you find, erm, that helpful in alleviating like, you know, any feelings of, erm I dunno a lot of people often link pets with, you know, erm potentially alleviating any feelings of I mean, I I absolutely love my partner's dog.**

RES Yeah

INT1 **You know, absolutely fantastic.**

RES Yeah

INT1 **Do you find,do you find that having a pet has a therapeutic**

RES Definitely

INT1 **Yeah?**

RES Yeah, definitely. Yeah, yeah. Yeah. I mean, when I say I got married in the first time 1991 within (unclear, 00:27:530, 1992, we we we got a bulldog, which I always wanted and, and, and since then, I've always had dogs. Apart from when I had a flat by myself for a couple years. It was very (unclear,00:28:05), but even then, though, the people downstairs had a German shepherd and I got to know it and they and they and they g gave me a key and and me and this German Shepherd would go out for long walks. And he'd come to mine for s sleep overs (laughs)

INT1 **(laughs) Amazing (laughs)**

RES Yeah, I love dogs.

INT1 **Yeah, yeah. Over, sorry, go on** CB

INT2 ***It was just like, I just wanted some clarification. Was your wife working in an office? Is she just working from home because of the pandemic?***

RES she uhh ,like REDACTED's a a a legal sec secretary. She.. w what she do what she do, the firm… I think I think the firm shut down first. So so so so REDACTED was working, working from home for about the first three or four weeks then(unclear,00:29:03) about going back to to to the workplace. So obviously went there.

INT2 ***Did you like her work in at home, do you find that it's still company with her being there? Or would you prefer her to be away in the office?***

RES If I'm honest, if I'm honest, it'd be better if she went went to to the office full time but then, uhh, her her and her work colleagues who ha both do they both do the same job, REDACTED

lives all, REDACTED probably about four or five miles away from the office, her work colleague, another legal secretary lives about, lived about 13 miles, now 13 miles away. Now the powers at bay said basically said to REDACTED (unclear, 00:29:57) ,you got to come out, c c come back come back to the office full time. She said, why is that, she said, and she got told because you live nearer then Ali, and I'm like well what about Ali then, she said well Ali lives 13 miles away, a and straight away I went well, that's not fair because to be told if anything that you live nearer, isn't fair. So, so so person has said, so you know, so so the compromise was the fact that REDACTED would go to work Monday, Wednesday, Fridays, ali would go there Tuesdays, Thursdays.Uhh, and it has been bloody difficult because, uhh because, you know, when the weather's crap, I've got to be quiet. There's only so much uhh uhh dog walking, I can go. But but now the erm gyms open now. It's all good, or it's better. Does that make sense?

INT1 Yeah, absolutely. Erm, in terms of your social life and your your social relationships, that the people that are around you. Erm, has that, have there been any changes in those relationships over the past year? And that can be, that doesn't have to just be negative, that could be positive as well?

RES Has there been any changes? No er erm, I, you know, both, you know, both Me, me, me, me and the outlaws now we get on. I mean, I mean, we, well, what's the difference between in laws and outlaws? (laughs) Outlaws are wanted? I mean, I mean, me. I mean, me me and the outlaws get on pretty well, anyway. But more so now though. We've gone in pairs, in pairs such as same as same as the teammates, same as, tennis, tennis, tennis group (unclear, 00:31:47- 00:31:54)we've got even better now yeah, does that make sense?

INT1 Yeah. So w would you say that those relationships have been strengthened over the past year?

RES That's the one yeah, that's the one. Yeah, yeah. Yeah. Yeah. I think as well, I think as well erm erm, everyone, everyone, you know, has gone through this. I mean, the fact that a a if you're home with you know, with young kids to be stuck with them to do the home-schooling is a yaknow, must be a bloody nightmare. CB! (laughs)

INT1 She's well there

INT2 yeah, we'd best not talk about it! (laughs) I'm still distressed (laughs).

RES (Laughs) Back at school now though, are they? Yeah, so erm, yeah, no no erm I'ts all madness!

INT1 Yeah. Erm, one thing to ah obviously, you, you based on what you said, there actually isn't ..a massive amount of you experiencing loneliness,

RES no,

INT1 But in terms of your definition of loneliness, and how you understand loneliness,

RES yeah,

INT1 Do you think people in your life understand,erm, you know, what loneliness means to you? Or or how you would define loneliness, or would actually be able to, I guess, would be able to understand if you were lonely?

RES I don't, s s sorry, can you? Can you?

INT1 Yeah!

RES Sorry.

INT1 So essentially, I'm asking, sort of, do you think people in your life or do you think people, the people around you, the people closest to you, so for example, your wife or or your tennis friends or anyone like that, do you think they would be able to,erm, understand, for example, if you were lonely?

RES I… don't…know. I know, because I personally, I don't get, I don't get lonely, I guess, because of my erm, cognitive fatigue I get..erm I get down there, but I don't get yaknow, I don't I don't get lonely,so erm I can't really, I'm sorry, my mum ,my idea of loneliness is sssomeone like we've all seen.. news. Erm,someone stuck in a care home, who can't see their friends and family and carers or people stuck at home who are too scared to go out to me, that's loneliness. And it made me and it just, you know, because there's Christmas time last year, I'm not seeing planning this year, protect the blah, blah, blah, blah. But okay, fine. I get that. But what if granny wants to see you? 'Well, we'll see her next year', you got a 52 fucking weeks between this Christmas and next year. And you know, and a lot can happen in that time. I I, yaknow only want to get the most from it each and every day. Does that make sense?

INT1 Yeah. If, what it feels like to me, is that and you can again correct me if I'm wrong, if I'm putting words in your mouth, but it feels like because of your experiences with your mother you've actually made an active decision to live life if that makes sense. Rather than

RES there's a drive, there's a drive there, I , I had the brain surgery (unclear:00:35:05) on the Sunday we had a peaceful uhh uh Sunday night. I I I I lay there on the sofa, the, the Monday I I I made the wife drive me, drive me to the local library, cause erm, I'm a massive book work, and to go there just to prove to other people, I'm fine. And then, and then, and then that was on the Monday then I had an appointment at (unclear, 00:35:32) the following Thursday or, or six days after surgery to chat with a doctor there. The Friday I walked, I walked to the REDACTED Lloyd which is just around the corner there, there's erm, they got a bar there, had a coffee there erm erm and one one of the rugby coaches is a doctor and and he's called REDACTED and he's Welsh, now doctor, erm erm, every year at the rugby club will go away for two or three days, bring all the kids there, had a few beers and stuff like that, and and Dr Martin, welsh, he said to me (welsh impression) 'Oh, Mikey boy, how's the first beer?', I'm like (unclear, :00:35:15) Oh, that that isn't like you! I sat there and thought, well, it's a week after brain ssurgery, I do like a beer, and I and I have to walk past a a JD wetherspoons which is down the road to go home, so I thought fuck it (laughs). So they all went had a beer, I got a photograph which (unclear, 00:36:41) I'll show ya , went in there, had a beer and then erm, the the daily, one one of one of the best things about my brain injury is how whole affects me, I'm a cheap date. So I was mainly there (unclear,00:37:00) after surgery. My my (unclear, 00:37:02) , two beers there, got home, had a few more beers, and and REDACTED came home and went bloody mad!(laughs)

INT1 (laughs) I'm not surprised!

RES I said to her, doctors orders! (laughs) yeah, doctors orders!

INT1 (Laughs)Very good. Yeah. erm, so there's not a massive, there there, I've just got a couple of more questions to ask,

RES Course, yeah,

INT1 and then we can move on to doing the erm, the nitty gritty of the measures. But if we think about lockdown specifically now, erm what would you say has been the most helpful thing or has most helped you in getting through this lockdown period, so that you know I'm talking about the past year really not just this third lockdown, but the the past year.

RES It's very much, it's very much case of I can't control what things are done, yaknow, what ,what yaknow what things are done or happened to me, the the only thing I can control is how I react to other things, because the whole thing about yaknow the term lockdown, we've all been in in lockdown, but there's still people coming into country every day, and, erm so what I've done is, you know, avoid erm social media, you know, in bits and pieces there, avoided news because it by it, you know, it's just about, you know, understand (unclear, 00:38:30) and and I will tell you is understand how I'm feeling, and then then man , man managing it as best I can. And that comes through just, you know, that patient lived in experience honesty, and yaknow, yaknow, so you know, and and, and, and sometimes just saying, fuck it, back to bed I go, and that's it for for a few hours. Because,erm, the way the way I see things is is to get through, yaknow, yaknow, it's better now because I can do things. But whatever, whatever it takes to get through that day providing, there's no drink, drugs, or violence, people, I mean, people, I mean, people, people have just done what they can to get through the that day the day. Does that make sense?

INT1 Yeah. Yeah, completely understand. In terms of, in terms of you personally, what have you found most difficult then? Your your mindset is obviously really good in terms of like, I really respect your outlook, in terms of over the past year though, like there must have been difficult periods,

RES of course, of course

INT1 what, what have you found most difficult about you know, when you're thinking about this lockdown over the past year?

RES Oh God there's so much.. it's like the most typical thing for me has been to sheer income in can the system see of the rules and guidelines because when none, none of it makes sense. Now, you, you guys are all obviously highly qualified (unclear, 00:40:05),now now for me sport, you know sport is great..erm, I can now play tennis outdoors, now I'm a bit too big and bit too old f f for s s s singles, so I play doubles in a league now, I play with my wife on Monday nights, and Wednesday, REDACTED, my one or my best mate who's, he's very high up in one of the local NHS trusts. I've had a jab, REDACTED had a small prick again, boom boom, REDACTED had both his jabs. The other player REDACTED, NHS, has had her her jab as well. Now, but we can't play, we can't play indoors over a 20 metre cold air, but I can be two, two metres away from a total stranger, puffing, puffing, breathing and breathing, breathing out of their mouths. And then the fact that we're locked down, but there's still been 12,000 people a day coming, coming into the erm, UK and the fact that this mask wearing stuff, you know, you know, and then last year, you're gonna have three points and a substantial bill. So okay, so I can go to the pub with a wife, she can drink loads of, you know, soft drinks,yaknow and coffee. If I have, yaknow, three pies, I gotta have a substantial meal with, (laughs) the, you know, the COVID COVID is clever, yaknow. Fair point? Yeah, I mean, and it just, I mean, I mean, you know, it's been the government's policies, who voted for Boris, you know, so. And the fact that the mainstream media are just, you know, the, the lies

and propaganda. I mean, I mean, I, you know, I I I said a year ago, this is all about control and compliance, and nothing..the capital, the government has implemented has changed. Cash now. Cash now is basically done away apart from takeaways, most places you pay on card.

INT1 Umm

RES Think about think online stuff there. We've all spent loads and loads and loads of money on Amazon there, so so so so so that their, that their profits have increased, they aren't paying any more tax, the local, independent businessman, they're just on their last aren't they!

INT1 Yeah.

RES You know, you know, and the bigger picture, none of it makes sense. None of it makes sense. Uhh uhh uhh because to me, you guys have probably heard this, the great (00:42:57)declaration came out last year, all these well-known societies basically saying protect the the the vulnerable, let the rest of of of all the rest of us, make up make up our minds as, as mature s s sensible grownups. Well, w uhh that wasn't even discussed.

INT1 umm

RES frustrating (laughs).

INT1 Yeah, yeah. Obviously, it has been hasn't it. I think, I think my my final question,

RES yeah

INT1 and I think I know, I know the answer to this one, but I'm going to ask anyway.

RES (laughs)

INT1 How do you feel about how did you feel about life after lockdown? So what your feelings about you know, things opening back up and actually returning to normal life for you personally.

RES I don't want much from life all,.. all I want is freedom to go to the gym, to go to the pub, to the pub gym, and tennis. And uhh, and normally h holidays abroad. Erm, I don't trust the government anymore, uhh. For me, it's very much a case of take, take each day as it comes now, now, CB will understand this because of my brain, my brain, my brain injury, I I suffer massively with cognitive fatigue that now, now today, one o'clock I've got my weekly zoom guitar (unclear, 00;44:26) this afternoon, I'll walk the dog then that's it, then tonight me, me , REDACTED, REDACTED, and my son are playing outdoor tennis. We wanted (unclear, 00:44:35)which we shouldn't do. I will sneak along a couple of days here because the REDACTED Lloyd isn't in.. indoors so that's daft there. So that means that that means tonight I'll come home say half past eight, have a shower with the wife, tomorrow I'll be so, I'll be so bloody tired I won't get out of bed until 12 o'clock by then, by then that's another day. So, I am hopeful but, I just think ermm this is more about control compliance. The jab, the jab uhh was the, uh um um um the big thing there had the jab there, most people have had a jab. Now Boris, you're saying second jabs. Okay, fine. Now you're saying, take take take two, two pills. All of those in time there, who's who's making the money then the pharmaceutical companies, most of us, or, or the average person there is on their last. Look at students, I mean, God, frightening. You know, it's been mismanaged. So I'm, you know, I I I'm not gonna say I'm hopeful. But, you know, yaknow I'm still frustrated, b but I proceed, you know, just take each day as it comes now, which is trying to kind of s sad but also it it gets me through

the day. Yaknow, you know, everyone, you know, everyone's just pissed off…and careful of it haven't we. Yaknow you guys can say that to say that because you're we're professionals (laughs) You're, you're health professionals!

INT2 ***(laughs)***

INT1 **(Laughs) yeah**

RES But, but, but if we look, if we look at the policies at Christmas, and look at the facts, none of it makes any sense. None of it makes sense.

INT2 ***Can I just check? And I'm pretty sure I know the answer to this as many of SDs questions. And you don't seem to be concerned at all about catching the virus.***

RES No

INT2 ***No fears about going back into society, has this always been the case? Have you, since we first heard about it sort of last January, February time. Have you never had a concern about actually catching it would be important.***

RES No, no, the, the the the the only things guaranteed in life are death, taxes, and the uhh f furniture sales from the DBS.

INT1 **(laughs)**

Int2 ***(laughs)***

RES No, no we're all gonna live we're all gonna die. I wanted as a yaknow, and this is probably due to my like, my Buddhism sort of way of thinking there. I think they're all going live and die, I I mean, look at the face. No, no, the way the way I see things is those of us who have carried on as best we can over the past year, ie. Gyms, tennis going to the shop coming, coming coming to the p pub, the chances are at some point, we've, you know, we've caught it, I had to trust my my immune system. And then when I get ill, when I die, that's it! I didn't come back, re reincarnation. But we walk and we live. We you know, we will get older we will get sick, we will die! Fact of life you can't change it. You can't hide it can ya!

INT1 **One thing one thing not touched on there, I guess is the the religion have you always been quite religious in terms of**

RES Oh oh no, I I I am not r I'm not r r uh r religious but my my my if you like thought process has has been towards Buddhism and and and has been for donkey's years.

INT1 **Right okay, cool**

RES donkeys , so I'm not you know, I'm not I'm not I'm not a churchgoer. I still, yaknow like a beer, like my chicken, like my food but to me is that is that is that being in touch with yourself and and and your own moral compass but no, I mean, am I scared of catching the virus? No. If I catch the virus or I get ill I'll go to bed or go to hospital. The day I die, then I die and then everyone who's pissed me off I would then come back and haunt them.

INT2 ***(laughs)***

INT1 **(laughs) Very good.**

INT2 ***I love your Outlook. Sorry. I just got one last question if that's all right.***

RES Of course! Yeah

INT2 Y You seem really self-aware of yourself and you've really got a fantastic mindset. Have you always had that, or do you think the stroke has changed that a little bit and you've become more, erm, determined if you like, to make sure you enjoy your life?

RES One, I I I I I I never never had a stroke, it was a brain tumour. Uhh I..as a teenager, I was bullied back at school q quite badly then started working, you know really shy, shy and nervous and then I was was 19 years old I started boxing and and and and through the years of that, yaknow, suddenly, yaknow brough brough brought out my confidence and that's why boxing is a brutal sport. I mean, I can't watch it now because of the fact that the box is trying to you know hurt somebody, any sport is good. The rugby my favourite, football when the ball, all sport is good because it changes you. So uhh, sorry c can we I forgot the question again? (laughs) Sorry, my brain goes off don't it.

INT2 That's all right. It was it was just to say, have you always had this sort of really positive outlook of really knowing yourself? You're very sort of self aware?

RES No, of course, no, no, no, no, oh christ no no no no no , that, that, that, that that comes about, uhhh, that that ..I guess, I guess, since about 25 years old. That's always been there. But but , but I know myself more so more so now with with, yaknow, with with , you know, with a brain injury, because I, you know, uhh, I can't push through, I can't push through my brain injury. Now my game, the only thing I can do is is manage it. So, uhh yaknow and that's why, yaknow, I set myself, I mentally have a plan of things to do, yaknow, each day now it's because I'm so uhh c c cognitive fatigue. I can't uhh I'll just say to myself , fuck it. I can't do is not important I'll do it later and at first yaknow, the first few years of that you know, I I tend to beat myself up but now.. it can wait…

INT1 Excellent, sorry, sorry,

RES Hello? (laughs)

INT1 that was me. I was I was having a really quick check at your umm, your survey data,

RES Yeah

INT1 sorry about , I was having a quick check your survey data just to see uhh what demographic detail we had because I, what I've what I've remembered, unfortunately Mike, you're the first participant where we've had for this study to have a chat to I completely forgot to ask you a whole host of questions at the beginning. So So

RES (unclear, talks over)

INT1 (laughs) yeah, I know, yeah, I will be doing that. And so effect, so that's the that's the interview component of this, this done, erm so uhh unless you've got any more questions, CB, **you're good. Okay, perfect. And so yeah, really appreciate you sitting through erm that with us. The next thing, next thing is left is just 123456 measures and just ask you some demographic data that I already I think I have the the answers to anyway.**

Compiled Transcripts

Interviewer(s): **INT1**

INT2

Respondent(s): RES (P2)

INT1 …and erm your employment status?

RES Eh yeah, so I'm uh going through a process, at the minute of becoming retired. In that post-injury I've not actually been able to work… and yeah (tearing up) just in me saying that it's a- it's an emotional time, it's just in the fact that y'know coming to terms with not being able to work is um is gonna be hard for anybody, but the it's al- it's three years now since my accident so it's I-I have come to terms with the fact that y'know I'm not gonna be able to work so just um getting getting retirement is that it's I'm still on (smiling) there's a few things that that I'm still on a company books basically uh and I'll also then I'll- (smiling) you might ask anyway, but I actually started the company so it-it was uh it was uh if you like me exiting from it, yeah. Which in all, al-all sounds good but hence why I might even be more emotional th-than it wasn't just a normal job y'know… but anyway eh so yeah I'm retired.

INT1 Yeah totally… okay (chuckle). Erm would you be happy to tell us a bit about your brain injury?

RES Course I would, yeah. So um I… was always eh a keen cyclist and I um (00:05:02 unclear "I don't ??? saying I'll go back") so I'm originally actually from Liverpool and eh I've been in Aberdeen now th-thirty-two years or something I came in as an engineer, but why why I'm going back to Liverpool (smiling) is I genuinely used to race quite competitively and I used to- and I'm not making this up, I used to beat a guy called REDACTED (smiling) that you might've heard of, yeah and obviously we taught different paths, but my point is that it shows that actually it's m-my competitiveness an- has always been there so um wha- n-n-never I never erm went professional, but I always stayed on my bike and- So what happened in May 2018 is that I was doing an event called 'The Tour of the Highlands' eh which is a-a sportif and so it's not officially a race it's just a probably a dozen people- no sorry a thousand people going and um (00:06:05 unclear) go riding with friends and I'll (chuckle) say th-this just so that you guys understand is that the the competitiveness in me was so much that I used to still be able to beat guys in their twenties and y'know (laughing) and so I-I couldn't help myself s-so this um and I used to invent the th-the sprints to go for and it's important because eh I was just coming- it was the end of the thir- it was a three day event and basically again from when I grew up if you come to a speed sign that changes, so you come into a village that's like a from one restricted to a 30 that's a sprint. So, the first one to that. That's obviously

(smiling) anybody that knows me and any actually anybody that's raced knows th-that's the rule t- of cycling. A-a I should ask are either of you cyclists by the way?

INT1 No, sorry (chuckles).

RES Oh okay okay that's okay, but so… eh we were just coming into Fort William which was the last eh… i-it was em Fort William just Glencoe. So it was another a few miles away to the finish after the third day in the last afternoon and I was just- it was basically the last time for a sprint, (smiling) which I'd knew coming into Fort William and unfortunately which never generally happened is I I'd l- and I don't remember any of this cause I went into a coma, but I apparently just hit a slight undulation in the road, I was sprinting for no reason other than a few guys that were coming with me were into it and unbelievably I came off my bike which it'd be fine if I'd just hit the tarmac but actually it was just somebody had a rockery at the side of their garden and that's what my helmet then went into which penetrated obviously my brain and gave me a bleed an-and so then the the good things about it um in terms of surviving is that um I was five-four hundred meters away from Fort William Ambulance eh an-and eh Fire Station. So there was somebody on the scene fairly quickly and because of the part of the world that it happened in, they were used to people falling off mountains y'know it's obviously next to Ben Nevis and things and so they then- an-and I'm obviously told all this and it's in my records. I was then flown straight to Glasgow to… head injury. I can't, i-is it called Queen Margret I thinkit's eh something like that in Glasgow, so that's where they flew me and what I would say is that… I'm definitely only here because the ambulance guys, who I've actually had an opportunity to meet. The policeman that was first on the scene- I wanted to go and meet them and also um the ambulance guys and I'm definitely only here because they knew what they were doing, yeah, with a head injury and um and then eh yeah to be honest with you. Well, as I say I was in a coma, so, I-I will quote is that I was three on the Glasgow Coma Score, so effectively if you know anything i-it's I shouldn't really be here y'know, but they knew what to do and then by the time they got me into Glasgow I'd gone up to I think nine or something so y'know I was heading in the in the right direction, but also I an-and I'll try not get emotional, but the policeman had actually been told to get in touch with my wife to basically just say that (crying) I hadn't made it um… sorry, I just um so anyway thank god w-that it happened in the in the place that it did, and then I was there, which I don't remember, but I was there apparently for two weeks. But then they normally try and get you back home, which was Aberdeen and in… Aberdeen I'll probably give- tell me to shut up by the way if I'm going on too much, but in in Aberdeen again thank goodness they have a neuro ward which is called I think it's '10205' or something I used to know the number and actually I knew where it was and then again unbelievably as part of the NHS there's uh there's a care home outside Aberdeen which is part of it, which is called- I can't remember the name of it, but I was there… so I was in Aberdeen for… so Glasgow for a couple of weeks, Aberdeen for three weeks and then this care home outside Aberdeen for a month, something like that and then what I did know because I cottoned onto it is that- and there's so many classic things that happened to me in terms of brain injury eh in that for example I thought that they were trapping me, I didn't need to be there, I was fine. Y'know you will of, I'm sure both of you- that y'know I was fine well y'know I could why wouldn't they let me home? And the other thing is I needed to get back to work, yeah, and I'd say it was probably… eas-easily took six months, my wife even

might even say and I might agree two years before I realised that actually I wasn't right y'know and I wasn't 'normal' whatever that means y'know em, an-and that actually then t- S-so anyway the point is a freak accident em eh yeah, so, yeah, it's important again so you guys know so that um I actually landed at this rock- you got I was sprinting, so I was doing about 50mph, the point being is that I was flat out yeah and um and absolutely going for it, but the point is if I had've just been normally cycling I wouldn't of probably had the injury, that this rock penetrated through the helmet and again I'm only here because I was wearing that helmet, so it was the uh the right temporal lobe is that the one? And then the ricochet eh onto the left eh um an-and all I've seen is the, is it MRI? The scan that you go, is t- I'd say it was the size of about a fifty pence piece missing off my brain on that side and on that side its about a normal 10p (laughing) that sort of size. Y'know but the other thing is just yeah them, I'll say them as in the NHS, thank god, but y'know them showing that just bringing to terms that this bit is missing and- the other thing that it doesn't become clear now is that I couldn't actually speak, I had to relearn words and um I could draw as in diagrams, but I couldn't actually use words because whatever part of my brain had gone is where obviously I kept all of the ability to speak, I've had to relearn if you like um and- Yeah, so then then brilliantly in eh Aberdeen or I think in Scotland there is something called- they do a brain injury rehabilitation course that happens twice a year, obviously I don't think it happens with the COVID, but so in tw- early 2019 I did a six week course for brain injury in a- in a out eh for the NHS and that was just… I was so lucky t-to get that in terms of it was thirteen of us on the course em and it wasn't that we all went on about- we all knew we'd had a brain injury and it wasn't that we'd like to introduce why we'd had it, but it was all sorts from the fact that, well there's a few good friends that I still have because of the brain injury and all ages that I'd say as well there was uh I was towards the hi- the older end, but thers eh a friend of mine that's a professor of eh in Aberdeen and he was into I forget the name of the words, but basically plants and he was on an exhibition somewhere and there was one something a disease that got into his body and they knew that it existed and they were really careful but it was only months later that they realised it got actually into his brain so this um- but my point being is that there was a range some and there was a young guy as well that'd probably in his early twenties, but just shared experience, and you're a psychologist and a clinical psychologist which is just brilliant because suddenly you're in a world where which is the significant thing is that people understood because in on on ward 205 they definitely there were people there that understood, but you're in this big hospital where that actually there was everything going on and I'm sure I'm not the only one to say this, but I was then in this freak world where I looked- I-I had broke my right shoulder so that was the only thing and I've actually lost hearing in my right ear, but other than that physically I looked exactly the same and yeah so I was in this freak world where I'd say I-I um I would've been guilty of it is almost like we would just judge the world based on what we see physically we didn't understand the mentally. Y'know I think we have got better I mean as society and understanding mentally and I get to say not least with what's happening in the pandemic its uh its people are gonna realise that actually mental health is just y'know so enormous, but I-I-I've been call myself guilty is that I'm pretty sure if I'd ever met anybody in work or uh personally that had been complaining about something that would've been mental health without saying that I would've just said "pull yourself together and you'll be fine" y'know and then suddenly I was in this world where y'know actually yeah the only people that understood me was were just y'know all specialist that had actually understood brain eh brain injury as well eh em because yeah eh and yeah so I was then um probably the easily the greatest loneliness would've been the fact that (tearing up)… as I say I get

emotional but I'm fine is that nobody understood- I didn't even understand what was bloody going on and that everything that you were able to do then you couldn't and not least in that realising that, well I'm way better now in that I have relearnt to speak, but y'know I still know that it's not the same vocabulary that I used to have and so it was the fact that nobody, I can say that even my dear wife had to learn y'know that actually I'm not the same y'know and and so the idea of- and fatigue, again I know you'll know this, fatigue was easily or is still easily the biggest thing but I just manage it because again the brain re- injury rehabilitation course I know that and I genuinely do things like and my wife can even say is that like say like for example even before this call I knew I was just needed to sit here for an hour and just do nothing an-and I'm not and I don't in any way feel lonely about doing that I just know that's what I've got to do in order to get the energy in order to speak… after y'know so the thing that if you go back to pre-pandemic I just had to manage my time which meant that if we were on the rare occasion we used to go out on the evenings I typically I got into the fact that I'd be in bed by nine o'clcok, whereas y'know I go back, the funny thing is as well people remind me about age in the fact that actually I talk about post-injury of things I can't do and they people good friends of mine say "REDACTED, but none of us could do that you just (unclear 00:19:21)" I just unfortunately whatever it was just used to push myself y'know and you go back to the fact i-in-in time with work you might I occasionally went abroad and y'know you might stay up the night and you'd still have a great days work and there's no way even now I kinda definitely need to be honest with you ten hours probably um uh y'know and that just um- as I say just managed my energy. So, the the point of all of that is that yeah, nobody understood, the only people that understood were as I say psychology or neuropsychologist y'know in that they had probably already seen this and Im not- I'm generalising but y'know I knew that all the people that had been on the brain injury rehabilitation course that we did for six- so it was six weeks, I dindt tell say but it was just one day a week of the six weeks um and uh yeah no and the whole thing of that was that as well was suddenly you were meeting people with shared stories and actually somebody would say and y'know go around the table and go yeah- "that's what happens to me" do y'know and then you suddenly just found this conection with people because again it was the fact that you- it's a hidden disability y'know and um just in that and I obviously early on had to deal with- now I think people are aware, but I had to deal with "why are you doing that?" or "why y'know come on out, you'll be fine" do y'know and um and the the other thing that happened not that I was- yeah so I definitely miss cycling massively I mean I I used to probably uh well th-the other mad thing is that that happened in Aberdeen and um I'm thinking everywhere is cycling was definitely the new golf y'know it used to be that people organised business golf outings, whereas cycling was easily the biggest thing in the- workwise with uh- so I was we were an engineering consultancy an-uh and I did most of our work for oil companies or energy companies as they're called now, but y'know th-they would organise BP would organise the big event that you would get invited to and I-I I was well we'll say I was quite well known round Aberdeen for cycling wise, as well as work. But, it was um so it was an important eh part that suddenly that was gone and uh and then the fact you couldn't go out in the evenings um and I'll jump forward, what I would say and I don't mean to sound nasty it's just the fact a year ago when this pandemic started kicking in, it was actually brilliant for me in the fact that everybody else suddenly their life changed and they got that actually things slowed down I will add that I've had an offer now by the way I would like to at least go and meet somebody for a coffee but uh but yeah so the the point being is that every bodies suddenly life changed and suddenly people started understanding actually not just (unclear 00:22:50) y'know life can change and s-so that I-I'd

say up until to be honest until Christmas… and as I say I don't mean to be sound evil but it was just brilliant for me because everybody was suddenly in, what I say "the same boat" y'know a-and it was almost like I was ahead of the game because it had happened to me because it happened years before that I was eh actually able to deal with it more and it was great and I was uh meeting a- friends for coffee or lunch em when y'know it changes obviously when you could and it was interesting in that I-I'd learnt- Iknow it doesn't sound like this now- but I'd learnt this new skill it seemed and I just used to listen and then I'd say a word and it would encourage them to carry on, so the good thing is and I enjoyed meeting always enjoy meeting friends but I the thing that- its just an offload for them to be honest really which is good but that's- I wasn't ever aware that I would do that but eh I suddenly become more aware of eh yeah I was more conscious of emotion and eh mental behaviours really.

INT2 Did you find that the people you were meeting were- obviously these are your friends that know… that know you and know what you've been through, did you find that they may be understood more?

RES Absolutely, that's exactly what it comes down to is that suddenly they just like kind of got an incline of what might of happened to me y'know. Or if you'd like what I've been through and I don't mean to bring it back to me, but y'know a few of them did say "REDACTED, Jesus man now I know what you were talking about 18 months ago or a year ago" so yes, that was good in that suddenly its almost like I became more inclusive in the world again. Now, that's a big thing to say but just the fact that everybody suddenly y'know doing… we used to do team meetings eh we used to do online anyway because you'd speak to people offshore or you'd speak to people in Houston, so in our industry it was it was normal if you like y'know if I'd ever suggested to a friend or even my sister down in Liverpool there's now way she would've did a video call, but suddenly every bodies into eh doing a video call and that the other brilliant thing as well is that it didn't matter where you were. People were obsessed with the fact that if you so if I didn't go out for a night then em y'know there were obviously concerned but obviously obsessed that you weren't there and then suddenly everybody realised well actually w-we can sit here me and my wife for a night and catch-up with friends and y'know and I'm not saying we'll do that forever but suddenly its everybody understands that you can do it, so eh so yeah the pandemics actually helped.

INT1 Yeah, I can totally understand where you're coming from. Did you find that you were em offering support and advice to your friends that were realising what you'd been going through?

RES A-Absolutely absolutely and I wasn't I wasn't eh I'll say doing it so obvious way, but y'know I didn't say "oh yeah that's what I-" but I just listened, and I thought that exactly what I (chuckles) an-and all I all I would then say is that I probably might have been quoted a- quoting a neuropsychologist (smiling) I- fortunately in my in my rehabilitation is I probably was about eh let me think… was at least four of them that I'm

actually still in contact with just not- I mean when I say in contact with that one of them works at the university in Aberdeen and as I said prior to the pandemic they're all they all seem to work either in the NHS or in the university, I don't quite know how to- can I go between the two? I think is what is what happens but eh-

INT2 ***Yeah, so what the-***

INT1 **(Nodding) quite possibly-**

INT1 ***Yeah, sorry CB I'll let you answer sorry.***

(mumbling)

INT1 **Carry on.**

RES So, there's eh why I'm saying that is that I've known four of them during through various times in my rehabilitation and all I say is that I was probably always quite fast learner but I pick things up from them that just made total sense that then became in my head that actually when I heard somebody saying certain things and I just sort of quote something back to them and I can't give you and immediate example now, but y'know you'd get thes- somebody told me that like like for example eh the other one that's coming is "just cause you think its fact, y'know just cause you think its something is fact" that is just so relevant now, but y'know eh it was that during my early days of rehabilitation is that yeah y'know it was so just so important to realise okay, just cause something can- like what's the- why am I thinking that? I done all of that hence why, you both know I'm sure yourselves that why couldn't eh didn't have much spare energy is cause I was probably doing too much bloody processing myself y'know and before I even before I went to meet somebody for a coffee I would have to think through what they were gonna ask me and what I needed to answer them and the similar element of that but I think I'm a bit faster at it now (chuckle).

INT1 **Yeah, it sounds like you've had quite a positive rehabilitation in phrase, would you agree with that?**

RES Totally, yeah-

INT1 **W-what do you think with the best- obviously I presume that that the six weeks rehabilitation eh course that you did. Would you say that was the… the best thing for you? Or were there things as well that contributed?**

RES Absolutely, em the the so being in hospital I think its important as well is that I never I've never been in hospital. So the point is I was one of these humans that actually or adults that they just weren't nice places and I have been into them to some bodies eh given birth or eh I've been into them with having an operation or something, but y'know there's not that I'm totally adverse to them it's just that I was never in them. But suddenly I was in this place and eh not only was I in this place but eh there's various wards for- I just remember the fact that I knew I needed to be working so uh and I forget that one of the eh neuropsychologists told me this is that I was obsessed with apparently trying to help the nurses and doctors so I used to get- I tried to follow them and help them (chuckles) I didn't realise that obviously I couldn't bloody do it but it's just eh y'know I needed to be needed to be working again if you like. Um and eh yeah so then I was in this out- we call it outreach place it was c- it's called REDACTED actually in- and its… I know I was in there as part of the NHS but it's actually run by a private business I don't quite know how they work but um but it was just people with head injuries and that was brilliant in that all of the staff knew exactly how people behaved and what they did. Actually, it was also quite disturbing because there was a range of head injuries in there and I do remember some of the people would just be screaming to y'know and screaming out as in they didn't want to be there and I this is the difference between them and me is that actually I-I knew I didn't need to be there at all. I didn't need to be there, but actually I thought okay now just play along with it because actually apparently what I used to do is I couldn't- I can't even think of the name- Gestapo y'know Gestapo so I'd came up with the fact that they were keeping me prison and actually the only way you get out of that prison, I've never been in is that you've got to serve your timeso I just serve my time and they they actually cottoned on and again I seen one of the neuropsychologists a few times after, she knew that exactly, which is just learnt again that I just had to give them the right answer, wasn't- and they even set me up to check that actually that I thought I'd just given the answer that they wanted to hear, it wasn't what I thought y'know and that and actually what I then had learned is that actually they were just checking that Louise my wife could actually cope with it too to be honest, which em yeah so so so then from getting discharged what after just two and a half months from the NHS to then, effectively I suppose being on your own, in the fact that y'know it's just Louise and Lousie m-my wife she doe- fortunately she only works part time but but she was out y'know em and I knew that… Well again coming back to back to the this rehabilitation so there was definitely dark times before eh I went on the rehabilitation started in March 2019 so y'know if you imagine a gap from late summer after being discharged to then. And what I actually was good during that time I did I think every, I'm gonna say every fortnight it might've been every month I don't I see eh and come on you're a neuropsychologist, I dunno it might be clinical sorry I just know that in eh and I did see a neuropsychologists a number of times which was eh really great help cause she taught me something that I've I say I still absolutely use, which is eh I just remember is ACE, Achievement, Connection Enjoyment and actually the eh but the the important thing then is I started to realise how I was pre-accident and that actually I do love people and I love eh being with people so I deliberately pushed myself out to make sure that I met people for eh a coffee or lunch y'know em but yeah so so so so I pushed myself but actually I prefer not to. The reality is that if you go back to if I go back to that time and I don't say this lightly is that if I could've just been in a cave and stayed there then that would've been I say fine, that's fine y'know and that's what I would've preferred because actually getting out going out people would ask me questions which was like y'know I dunno

this was mad but they'd say "how are you" it's like y'know and I had obviously I'd learnt responses to everything then actually I realised so much of conversation is just it's just trivia do y'know and I obviously had learnt the trivia bit em and and the other brilliant thing again that this eh REDACTED she was called, neuropsychologist she eh she said to me- taught me about actually deflecting things yeah and actually its brilliant in that people would ask me questions y'know I'd answer it really shallow, but actually they're now saying the same thing back they were then these thoughs distracted from the fact that actually I dindt really need to think y'know and it wasn't- you could've then tested me on what did they say, I didn't really listen. It just didn't go in y'know. Em so yeah so then going in the rehabilitation course it just reinforcing the fact- yeah there was two eh two doctors who, neuropsychologists or clinical psychologists and there was three students actually on that's just graduating or just graduated and and so five of them versus the thirteen of us it was really good mix y'know and you were- you could just uh yeah so I really looked forward to that to be honest and I go up to the point that as we all did, after that finished what next? Y'know and yeah w-we brilliantly there is them through headway there is eh a local branch in Aberdeen that's called it's called REDACTED, is eh yeah and eh so um so yeah through through that then made me realise that there are people out there that y'know have unfortunately had a brain injury for whatever reason and actually that (chuckling) they're fine y'know its nice they, we've got something in common and what I would also say is that I've actually eh had a friend- got a friend years ago, I sort of eh probably went on our ways ten years before my injury but um he had he actually had a speak stroke and I do remember and why why I'm saying is not to just bring him into it is actually he never got the help that I got because he he never realised he was any different he never realised he wasn't gonna work so actually what I also then realised is as you guys will of it was so bloody important that my wife was everything to making sure that I got to go into REDACTED that y'know staying in the hospital is that I don't- and in the good days although I wasn't at the time she definitely made sure that I focused on rehabilitation because yeah she wasn't- she didn't get it, didn't understand it and em I wouldn't of probably been well I certainly wouldn't have been able to eh do this now do y'know I wouldn't have been even necessarily been speaking in the way that I was just because um yeah she she pushed me but I I got that it was actually eh everything that well its now I get in life is that if there's a point and again I've seen it with the pandemic if there's a point where I think I really don't want to do that I really cannot to that I actually do do it because afterwards I'm so pleased that I've done it (chuckling) that's consistent in everything that I do is I kind of think y'know eh geez I really can't (unclear 00:38:38) but actually every time I've done something that I've pushed I'm really pleased that I have.

INT1 **Aw, that's good. It sounds like your wife is very supportive of you as well an-and helped you along the way. Em can you tell me a little bit more about the people that you've seen during the pandemic? Sort of since we've been in lockdown em have you seen many people? Have yoU managed to stay all connected?**

RES Eh yeah so I've uk stayed connected uh through through using zoomto be honest yeah and and again it would've been that a load of friends who… my sister jokes in the fact that I-I was always a geek and techy anyway an um what I haven't- I'm an engineer so eh and so y'know I was probably always slightly off the scale in terms of what normal was and those of my friends who were in engineers or techys y'know we were- we don't have an issue with so

catching up with the zoom, on zoom or teams or what other um but then it was as I said the brilliant thing is that suddenly I could just catch up with my family and my wifes from REDACTED actually so originally eh although she came here as a student shes actually eh a she's PHD ne- ah I forget the bloody name she's doing this um… not just eating things nutrition is what I'm trying to say, its nutrition yeah. She eh used to be in REDACTED eh she she let eh she was let go from there about five years ago actually em but em why I'm saying that is that shes from Dublin but or originally from Dublin and her family is still obviously over there and her sister and brother and so again just having a zoom with them is that y'know eh with the pandemic I mean obviously she has spend some time over there but it wasn't as easy as it used to be and so um again in tying with the fact that actually she post my injury I couldn't gp over for a weekend it's just too much so we used to go for a week and then that had to I had to tie in with Louise's work and when could we stay and things is always very difficult em whereas eh yeah just catching up with people people on zoom its eh its suddenly- well everybody became aware of it y'know and eh and that's why in that-anybody and well even my father in law actually died in January eh is eh ninety-three COVID had the final word eh well obviously we were over there after the small funeral but why I'm saying that is that for the last year he was quite happy to chat to you on eh whatever the app that they use, I call it zoom and he was a ninety-three year-old, he got it he got that you y'know you could chat to him which is just brilliant you know so suddenly as I've said-suddenly it becomes all inclusive really I think.

INT1 **Did you prefer the fact that you can stay at home and chat and everybody else is happy to do that as opposed to having to socialise out and about where you might get more tired and more lose more energy?**

RES Yeah It think I would use the word that I if that's all I could do as I definitely had enough of it and it is great now that I can I can meet friends for a coffee and in the local park, it sounds (laughing) but just y'know in the fact that obviously you can't go in a coffee house em just sitting outside on a bench is eh it is still great seeing people and I-I I'm gonna say this in whatever the sixth sense is you do get something else from just meeting people. But if if it was one or the other then actually I would pick this (gestures to zoom call), but given that actually I can do both then I love doing both because y'know if it was just seeing people it would just be a continuous stress just thinking okay I need to do, I need to go out or I need to- and especially everybody working in the day they generally wanted to see you at night, so that was the that was the issue but if em no yeah ca- I say just seeing people in the eh in the evenings yeah if it yeah if it becomes one this week y'know I'd be into that but equally catching up with people once or twice a week on zoom is bloody good.

INT1 **Is that about sort of how often you are seeing people at the minute? Is it a couple of times, three times a week or…**

RES Yeah I'd say probably a bit more but yeah um so actually actually I'd say this week just as an example is that yeah it's probably 50/50 so I've seen three people outside where I've met

them for a coffee and actually I've probably done about uh three earlier this week eh catch-ups with people. And the good thing eh the guy I met yesterday is actually we hadn't seen each other since well sorry we've zoomed each other but we actually hadn't gone for a lunch or coffee since last summer- August or something he reminded me but the point is that we've kept in touch y'know and actually then just meeting him for a coffee it was bloody good y'know-

INT1 Yeah, it's lovely isn't it.

RES An-and then he was he was then eh I'll say revealing to me or just chatting to me about the fact of how brilliant it is to see everybody in the office yeah and I mean he's really concerned about the young guys wh-who actually y'know started work and then the pandemic came and they they've done loads of work and y'know they're on the computers the whole time but actually he says they they just don't quite know how to behave in a- and its obviously the worlds gonna have.

INT1 Those social connections t- how to behave in public.

RES Absolutely absolutely.

INT1 Before the pandemic were you seeing more or less people? Were you- has the pandemic sort of prevented you from getting out and about more?

RES I would say so yeah prior to the pandemic I was really concious of seeing way less people than what I had been used to in terms of post-injury eh but the reality is I was probably was seeing I'm gonna say three people a week, different, over the week. So that was okay, but post the pandemic you could double that the same people with zoom. Yeah and and the other thing as well is that eh I'm sure both of you are aware is that we should I should say condition into, but eh recovering from a brain injury is not it's not quick effectively youre re-learning a lot y'know so the idea of two years is definitely y'know I'd say the early days, three is kind of like what y'know where I am now is sort of- yeah I'm way better than I thought I was in a year. The other thing then is that everybody- I'm saying everybody is that normal people just kind of assume, again if somebodies got a broken leg y'know how long it takes to fix and you you assume y'know and and the usual thing was that when you cause again just from zoom it's obviously that it's nice to catch up and chat with people but y'know you don't see certain things whereas when I saw people out- outside they'd say "well youre walking very slow" or y'know and it's just in that y'know other things that they they suddenly realised in the fact that y'know I-I and it was the case that I did have to balance what worked for me, so I had to re-learn to- I say re-learn to walk but it was the fact that I was really concious that the fact that you couldn't just walk fortunately I don't even think about it now so it's it's- I''ve re-learnt it (chuckling).

INT1 Oth-other than the speech and your balance. What other sort of daily activities were affected when you first had your brain injury?

RES Em… so yeah just just actually y'know not being able to take part in conversations to be honest y'know and just and um what- and actually zoom is really amplified this is that we all know people and I might be one of those that actually never shut up and they're just constantly speaking and on zoom its eh its quite funny with my family, with my sister eh my wifes family sister especially is actually two hours is she actually fine but suddenly I think you become more aware of it when you're on the computer.

INT1 So would you say…

iNT2 Sorry I was wondering-

iNT1 Go on REDACTED**.**

INT2 One thing I just wanted to check with you REDACTEDathan em obviously we originally built this for an hour are you okay I'm just in terms of your fatigue or anything like that-

RES No no I'm fine honestly-

INT2 Okay okay, if you wanna stop or anything just let us know.

RES I'll be absolutely fine, definitely saved enough energy REDACTED.

INT2 Perfect I just wanted to check just in case.

RES Yeah, no it's good of you to.

INT1 So I was just gonna say do you feel like the pandemic has actually been quite a positive em experience for yourself?

RES Yeah, definitely definitely.

INT1 What about the term loneliness, how would you define that?

RES Erm s-so eh eh probably a good f- question. I I'd say then loneliness eh yeah nobody understands you and especially then post-injury because to be honest with you I didn't really understand myself and I can say that now, but you didn't know that at the time do y'know? And eh so yeah a-a-a-as I've already said the only people that I could really speak to was the neuropsychologist or the clinical psychologist and whatever they had learnt they had learnt that you could- eh had this ability- sorry that's my eh the front door sort of going, my wife's getting it. But you em I just well I-I—I I'd be stronger in what is it in our education actually that had stopped people and it might've changed now is to actually just understand other people as opposed to- y'know I was in a world where I could do maths and physics and y'know em actually I was bloody useless at writing but that's okay I got- but y'know it was kinda like em just the, you just nobody understands you is what I would say y'know just- when I say nobody that's to say with the exception of people who've clearly been trained to to to understand but anyway em yeah so that's what I'd say.

INT1 Would you say that your em thoughts about loneliness have changed either because of your brain injury?

RES Oh definitely definitely yeah I would eh… I've actually probably I would've been one of the many people I'm now accusing in terms of I was oblivious to it y'know I-I would've sort of thought that y'know the classic idea "just pull yourself together" or "you'll be fine" yeah and um yes so now I suddenly well yeah I suddenly people somebody might say something and you kind of eh (chuckles) I kind of can't give you an example but I would say something back to them and say "well actually REDACTED that's a great thing yeah, I'll think about that y'know" and again what I've learnt so yeah it's got to be that eh.. the whole pandemic is the fact that there's people that are lonely aren't there.

INT1 Yeah, would you consider yourself lonely currently?

RES Em.. no actually. I don't now. I definitely don't now I'd say ironically (chuckles) eh as I've said anyway is that actually coming into the pandemic actually suddenly I became more included yeah from this world that actually I was totally alien from and nobody understood me to actually now okay I can say to you guys I don't say to people "see that's what I went through."

INT1 (Laughing) What you want to say, you've become the expert and you can eh educate all of us. Em can I just ask how how are you keeping yourself busy? What sorts of things are you doing through the day to to use up the time or your energy?

RES Yeah so the em main thing that I still try and do is I catch up with people and it might be that eh I only catch up with eh well I say it could be somebody every day and it might just be for eh a coffee and then the other thing that I've started doing not that either of you will be thinking is that I've actually got a smart train now on my bike in the garage so whilst I've not been on a bike outside post-accident I now actually I think eh I call her I can't remember her name but as I say she (unclear 00:54:08) she's the head brain surgeon in Aberdeen, but the head consultant em and she said to me she's a lovely person… she said to me "REDACTED if I ever hear you getting back on a bike I'm coming to break it" and I get it but equally I'm now on the bike that I eh crashed on and actually the ironic thing is I took all the injury the bike was still fine. But I'm actually now it's called the smart trainer but it's just an introduction back to the bike again, it's looking at a screen- I know I used to they've obviously been around but I used to take the Michael out of people who trained in a garage it was sad y'know I loved being out on the bike but suddenly now I'm I might only do it again, I might only do it at least 3 days a week and I might be away at it for an hour but the most I've done so far is two and a half hours, just to determine I used to be on my bike for six hours, five six hours y'know I would certainly be well the joke always used to be if you did less than 100 miles that wasn't really cycling. Y'know if you did sort of 120 miles and by you had to do a mile climb (unclear 00:55:38) cause that's called a unit of cycle its obviously all made up in my world but um yeah so then being on back on the bike which I've only done actually for the last month em… and by the way just in case you're- I'm probably fifteen kilos heavier than I was prior to my accident but again I could say that now and people will say yeah well I've put on weight during lockdown and (laughing) y'know its just so eh- and then the other thing that I do which actually I mainly did is I cooked and eh I-I-I I always have and eh I liked and actually this weekend in Scotland you can have people in your back garden now so we're having friends around and I'll cook something, I could say BBQ but it'll probably be a bit fancier.

INT1 Fantastic, lovely, I bet your wife appreciates all the eh the extra cooking that you've been doing.

RES Well the reality as I say is eh I probably always did the the cooking anyway. My my wife can cook but she's just, I just enjoy it that's one of my tasks and then in saying that then I go to the supermarket say once a week, I do get some things delivered but actually I deliberately go to the super market and it was all actually about pushing myself because there is post-injury there's no way for the first year could I have gone into a shop or I could of because there's been another thing as well another important thing which eh the pandemic has reinforced which is eh and I'm not making this up I couldn't deal with money and what I meant is the notes when y'know you used to pay with things with cash is that it was honestly so bloody complicated in terms of what it was and whereas now I just obviously do everything with the phone and and a good thing is I had started to do that, I've certainly one it before the pandemic but its just absolutely normal now if you were sometimes you went round to the corner shop and then they didn't you obviously ad to pay cash whereas now it's perfectly normal so yeah yeah that's just another trivial thing but just just one of the many things you don't realise just going into that shop was so bloody complicating so many

people in line where do you have to go so I did push myself. My point being is that I do still go during the pandemic I do still go into the supermarket obviously I say very careful but actually it's just it's a bit of an outing might sound sad it's just that's my duty if you like- duty's not the word one of my achievements in the week is to make sure to go to the supermarket.

INT1 Yeah… bit of a routine.

RES Yeah yeah routines so so and and and why I'm also adding that is in that Jesus man it takes energy from you and in that I wouldn't do that was in that planning my day y'know and an I'm planning my day and again I can say to you guys if you said to most people they would think you were taking the piss and actually all I can do is really have a zoom call meet somebody for a coffee and I wouldn't- and then go for a spin on my bike for an hour and that's my day.

INT1 Yeah, you sound like you've had quite a positive time throughout the lockdown and the pandemic, is there anything in particular that stands out that's helped you get through?

RES Em… other than repeating myself just the fact that somebody's life changed and they became conscious of it, the single thing that always helps is understanding, isn't it? So, everybody else is understanding without really being hammered home, you just suddenly get used to it.

INT1 Yeah definitely and has there been anything particularly difficult?

RES Em… yah so then this I'm gonna say this… a nice problem to have or a modern world problem is the fact that going- we used to go to Dublin probably- well Louise would go there probably six times a year and used to get on a plane in Aberdeen and go for a weekend. Whereas, just because of COVID just getting there, we went at Christmas and the reality is when we're next back we'll go in the summer. Whats really nice is when we go we go for at least a month whereas y'know its planning, previously we used to just be popping over there not being able to pop back to Louise's family is y'know but as I said we do catch up with them on zoom, which is as I said um yeah that that bit just not being able to regularly travel as you used to and I just mean to (unclear 01:01:27).

INT1 Yeah no I get that and then in terms of when we start to get back into society and lockdowns lifted and em how are you feeling about life getting back to… pre-pandemic, I won't say normal but how it was before?

RES Em… the the first thing that I have is concern because I'm actually now concerned as to whether I'm I feel way better over a year than I did before because actually of the pandemic and everybody slowing down and so I definitely have a a quite a- obviously I'll deal with it but quite a concern in that when people, when we get back to "normal" in that the expectations will be back on me in terms of eh going out and Friday Saturday for dinner the (chuckles) the other thing that's subtle but is honestly so brilliant is that em when restaurants were open in Aberdeen there was a period where they had to close by 10 and then they had to close by 8. That was brilliant because it actually meant we'd go out at 6 whereas pre prior to my accident we would meet somebody with might going out to a bar at 7 and the table at 8 and then you might get home at midnight or something whereas y'know I just don't have the energy and my point in me saying that is in that em I don't know what it'll I don't know how well energy wise but I know I'll have to manage it whereas if in a way this might sound mad but I'm sure you get it is that I know I can stay up till midnight because I have in the house but equally there's no pressure y'know. Whereas I definitely- we live in the city, but the idea of making sure I had enough energy that I could just walk home or get a taxi was constantly on my on my thought y'know and so so yeah, concern but I know I'll be able to manage it.

INT1 Brilliant em just going back to some things that you mentioned earlier, if that's alright, mainly about eh when you first had your brain injury and your friends couldn't understand why you couldn't go out, or why you weren't socialising as much. Have you lost any of those friends? Have they sort of drifted off over time because you've perhaps em denied or said no to social events? Or do you think you've remained the same?

RES No, I would I would generally say that I've remained the same but what probably to be more specific is that actually I've probably gained more friends. Some from the head injury rehabilitation group which I wouldn't want to not be, but also it seemed like I became which I got it was sensible I was suddenly meeting more people that were retired yeah and I I'd always been I might've worked with them like a group of guys that I had lunch with eh yesterday on zoom is eh we all worked together 25 years ago or 20 that sort of time but and we've always stayed in touch but suddenly I'm more connected with them because they've actually, they're all a few years older than me they've all formally retired and gone through the process of making the decision okay I'm gonna retire em whereas if you like it was forced upon me but it's just suddenly I then connect with people who are retired y'know and you can actually- as I say I wouldn't group myself as a retiree as I said I'm getting there in terms of being able to accept y'know what I'd learnt is that if I'd bumped into anybody in town and they'd said to me pre-pandemic they say "so you back at work yet REDACTED" I'd say "oh well I just do a bit of a bit of work for them I do-" I don't but I just I couldn't say actually I'm not y'know because then it would just drive something off em yeah so when I connected more with people that have actually as I say naturally went into retirement and actually then some of the actually some of the younger guys… I've I still see I still do the occasional zoom call with but their their life has just been turmoil in terms of the fact that they've not been able to go into work they've had to look after their young famiy so so I I I I get there is some people that I wouldn't see as much now but it's because they're not doing their regular things. There was guys that I used to eh see in the day and that was because they were coming into town and where I'd just go wander and see them whereas yeah now

they've uh… yeah they've if you like had to just get on dealing with their lives which is fine so so there's a mix.

INT2 ***One of the things eh you've mentioned a couple of times this idea of uh uh a weight of expectation em on you know and even in what you just said there around the idea of em some potential shame or embarrassment about retiring at this point in time.***

RES Yeah.

INT2 ***Do you feel that that has been lessened because of the pandemic? Or do you feel that is it is it is it a worry now that things are opening up and you'll be potentially having those conversations again? Or do you think there is a a more awareness now with people being furloughed or-***

RES I think that that yeah definitely it's eh the awareness of it now post of into the pandemic is easily gonna make it easier for me in the fact that y'know when I say to people actually I'm retired now, it won't be "Jesus" at the shock of it but they'll probably almost be because actually (unclear 01:08:14) but for some reason there might be guys that decided to retire at 55 yeah. By the way I always had a joke and I sort of meant it as I say backfired on me, I always used to say to people when they were saying "oh I'm thinking about retiring" I'd say "you work and then you die" (laughing) and I kind of said it as a joke because I never thought for a minute I would ever do this thing called retire do y'know and obviously it was forced upon me but suddenly as eh- there's a guy who's now, he's a very good friend of mine, who's become becoming sixty this year and he was toying with the idea, actually the pandemics just the fact he definitely he's retiring that's it y'know and there was eh another friend of mine (unclear 01:08:08) just around the corner who's he's fifty-eight and he he was one of these guys who would joke about the fact he'd love to retire when he's fifty-five but he never had enough money y'know and and so he always carried on but actually em yeah he he he's got to the point now yeah he's just gonna next year or sorry later this year he's just gonna call it a day so suddenly it's just forcing into people the fact that a-and actually what the sense of eh a couple of them is the fact that and I so get this, what was really good about work was the number of people that you were involved with y'know and the range of people you're involved with. It's eh quite quite eh I obviously had perhaps quite a unique industry but y'know there was people from all over the world and it's just eh… all ages y'know so em being if like one of your key drivers for work well at least for me anyways was it was just being with people and having a laugh y'know, whereas yeah, so this this has now brought home to everybody well yeah you you can't see everybody and what is it you want which- it's brought on another thing that I will just mention is that and I do mean it is that y'know I know eh we all know you need money to survive but actually it's so down it's so down the list in terms of y'know and again this pandemic has brought it home in that actually guys I know especially in Aberdeen that were probably earning a fortune and saving a fortune but what do they want it for do y'know it's like actually there's more to life y'know and eh so yeah there's um anyway yeah…

INT1 Yeah, no I totally agree with you and I think the pandemics brought out two types of people, the ones that realise family is important and being at home and spending more time and want to take a step back from work to… do that more. Or the others that think god I love work and I am missing it and get me back there right now (laughing). So I think there's and I agree with some of your colleagues that are nearing em retirement age, that they've enjoyed a lot of people have enjoyed being furloughed and being off and want more of that. Eh you mentioned earlier about headway, have you been involved with them or is it just sort of the support groups and things are you socialising with them much at all?

RES J-just with the local support group this uh one that's called BIG em which eh yeah so, I think why it's not called headway in Abedeen, I mean it's definitely affiliated with it, but I think Brain Injury Grampion started before headway. I-I can't remember there's eh a history I know I should know but I think the guy that started it his brother em was in the forces and there's (unclear 01:12:15) up the road had eh had an injury or something and he realised once he left the forces that to be honest with you it was probably a time in the forces where we didn't understand peoples mental state y'know, just let them go and he realised that his brother was missing something massively. So, it started BIG and then it became affiliated with Head Way.

INT1 Brilliant. Eh REDACTED **do you have any questions before I move onto the scales?**

INT2 No nothing more from me, thank you.

Interviewer(s): ***INT1***
INT2
Respondent(s): RES (P3)

INT2 **And erm, can I just ask about your brain injury**

Res Yeah

INT2 **Can you tell me a little bit about how it's come about.**

Res Erm so September 2016, erm, I was playing sport, erm collided with another player erm at the back of my head. And then like three days later, became very drowsy, dizzy erm and started getting really bad headaches. Erm and went to a&e and all that sort of stuff. Erm. Yeah, I went home because I couldn't stay awake from work. Erm and the next morning, I basically couldn't walk in a straight line and felt really bad. I thought I've got to go to a&e. Erm and they sort of at the time diagnosed me basically with concussion and told me to take it easy. Erm. Over the next three months, I erm felt pretty, well felt terrible, most of the time, erm this erm nausea, I had pretty bad nausea. As in when I was walking around anywhere or when I was erm standing looking at things that were moving, erm light sensitivity, noise sensitivity, erm fatigue, crippling headaches, migraines. I ended up with like three migraines in two months or something. erm and err ended up seeing someone privately who very quickly diagnosed me with some damage to erm my brain, some lost functions, erm occipital nerve damage and vestibular erm hypofunction err and various other things. And at that point, I stopped working, I started going into rehab and taking medication to try to relieve the headaches and the migraines. Erm and so I started the vestibular rehabilitation therapy erm at that time, which I did for, I mean 18 months. Erm but we wanted to have a baby. And we were like, we can't wait forever. Erm and I was doing quite well. And so then we were trying for baby, I had to come off of my medication to have the baby, to try and have the baby because its not supposed to be taken at the same time. Erm, and so yeah, pregnancy and shortly after having birth was pretty bad. And then I mean at nine months, then we went into lockdown. So when the baby was nine months old, then we went into lockdown. Erm and so we've been in lockdown ever since then. Erm I'm sure we'll talk we'll talk more about those, the effects of those on me and erm all of those some pieces. Erm as it happens, I'm actually, erm obviously, confidential, so that's fine, but I'm actually pregnant again now. But I've had, I have been we've been trying for nine months, and I had to come off of my medication again, erm which does mean that I may be slightly more fatigued and erm suffering headaches every day and all that sort of stuff. As previously really, so is an ongoing, you know, get off my medication. I think I said that. Erm, so it's like an ongoing still, my brain injury still affects me on a daily basis, really. Erm even though I got to the point where I had probably done quite a good recovery erm. But I still wasn't able to,

let's say go for a run or erm I could walk for about an hour at that point without symptoms. Erm and then the pregnancy is all (laughs) like, messed everything up again. Erm.

INT2 **Oh but, congratulations.**

Res Nice. Yeah. (unclear, 04:06, laugh)

INT2 **I bet I can imagine I can. Yeah, they did. It's tough being pregnant and, and in lockdown. I'm sure it's not much fun.**

Res I think with the brain injury makes it doubly worse somehow.

INT2 **Yes, definitely. But yeah, absolutely shattered, are you?**

Res Er Yeah

INT2 **Yeah (laughs) What about any types of rehabilitation have you had, obviously you'vementioned medication. Have you had anything else at all?**

Res well just this vestibular rehabilitation therapy, vrt, which is erm daily exercises to recalibrate your and help your brain relearn balance and eye movements, because those were the things for me that were not working.

INT2 **And have you found that helpful?**

Res It did definitely improve my symptoms erm over the period that I did it. But erm the thing with that is, erm you're pushing your brain every time to learn the new things, or to to work harder again, where you've lost functions that your brain would have done naturally, erm automatically before, so you do end up getting a lot more tired and a lot more it it does bring on your symptoms. That's kind of like the whole point, you're supposed to do it till just before your symptoms come on erm three times a day, every day. bAnd erm so it does make things worse. But over the, over the long term, you do see an improvement. And I take, so I take medication, I take amitriptyline, erm which is like an anti erm migraine and sort of anti sensitivity kind of medication is the way it's put to me. Erm, I take 10 milligrams a day, normally, but while I'm pregnant, I have to reduce that or sort of come off it. But (unclear, 06:06)

INT2 **No problem and how are you finding, without, without that medication, how you finding life at the minute,**

Res Erm I have symptoms every day. Where, where, where previously, we had got to the point where maybe I would have symptoms, like 60 or 70% of the time. Currently, I have symptoms, since I've been on my lower dose of medications, I have symptoms, like every day at some point.

INT2 **Okay. And is there anything else that you've found that you can do to manage that at all? Or is it just a case of getting on with it at the minute,**

Res Erm sleep is really the only thing that erm repairs, my energy level and my symptoms? So when my baby is asleep, I try and have a snooze. Erm or if I've got other things on I don't, I have to sit down whatever but erm and then I just try and get through it. Just try to get through the day, basically.

INT2 **Yeah,** REDACTED **is there anything else you want to ask about brain injury before I move on to lockdown.**

INT1 ***I erm, I phased out a bit there as in my, my teams went a bit screwy. Erm so I think I was frozen. And I think you asked already, but maybe I'm missed it. How have you been coping? On the reduced medication?***

Res Yeah, it erm it means that I have symptoms, really every day. I also felt withdrawal symptoms. When I came off of the medication, you have to kind of come off of it quite slowly. And the withdrawal symptoms are like dizziness, headaches, fatigue, they're all like the symptoms any way you would feel. I feel every day. Erm so yeah, now, since it's when I reduced my medication, which was err a year ago. Erm I have symptoms every day. 100% of the, 100% of days, but not all day. If that makes sense.

INT1 ***Yeah. Yeah. Yeah.***

INT2 **Yeah, yeah. So erm if we can just talk a little bit about lockdown. Erm can you tell me about the people that you've been in regular contact with or that have supported you whilst we've been in lockdown?**

Res	Yeah, I think the biggest change for me was that my husband stopped going into work and he was at home. Erm, err, so actually, err I have felt that I have had more contact in lockdown. Erm. Which I know it's maybe difficult to understand, but err I didn't, I don't really I can't go and meet with a group of people because I'm noise and light and movement sensitive. Erm so going to a busy pub isn't great for me. Erm so I tended not to have done very much of that anyway, and I had just had a baby. So, erm I had been spent the last, the previous nine months you know, recovering from having had a baby and you're like at home a lot with the baby. Erm and whilst you know, home by yourselves, most of the time with the baby and you do go out for walks and things but I was very exhausted and really not able to do most other social activities that I would have done erm, before having the baby like social activity (mumbled, 09:29). Since my accident, social activities are like a low anyway. So maybe I would do one or two things a month. And that would be the case of going for lunch somewhere or something or meeting up with a friend for dinner that it would literally be once or twice a month. Erm and then you have a baby and then you don't really do anything. Erm you maybe see people for quick like half an hour walk and then you're just too tired. You want to go to bed anyway and then lockdown happened. So actually for me, I had my husband now at home then with me every day erm and providing me with support. Erm yeah, for me even using a computer for a long period of time or doing kind of visual calls erm is also tiring because it's my eyes and my erm. You know, it’s the use of my eyes and my head. And so using, I don't use a computer really anymore because it's exacerbate my symptoms. So we did zoom calls with people erm. You know, with friends, everyone started doing zoom calls. I was like, I've been in lockdown for like, four years. And now you're all doing zoom calls, it’s just great. Erm, err yeah, I think maybe I know that other, Well, you’ll know better than I will, because you'll be talking to lots of different people. But erm there's like two camps where people have not been able to have their, go on their rehabilitation and not have their appointments and things like that. And I know that that's been very difficult for people. But there's also quite a lot of people who were like wired when I was in the same boat as we are now. And a lot more things have become more accessible. Everyone's deciding to do zoom calls all together, where I wouldn't have met up with all of my friends. No one had wanted to come meet me before, you know erm, that sounds, that sounds a bit. I think this is something that brain injury sufferers, or I have particularly felt, it is something that's generally felt, I think that you know, a lot often you lose a lot of friends, people don't have time to come and see you, people don't have time, and I can't travel and I can't drive and I can't do lots of things. I can't go to the pub, all the things that everyone wants to go and do. And someone in their mid 30s should be doing. Erm but now everyone else is in the same boat. Erm. So it was kind of quite nice.

***INT1	Has lock down been, lock down been a positive experience, then potentially,***

Res	It has, I would say, for me, it was a positive experience. Yeah, I felt no pressure to go and do things that were going to make me feel poorly. Erm No anxiety about having to get somewhere and how am I going to get there and what happens if I don't feel very well. And erm often, migraine starts. And, And I had my husband with me every day. Erm given that I don't work now, erm and people don't really come and see me. (Laughs) Because they've got their own lives.

**INT2	Yeah. Is your husband working from home, or has he, is he furloughed?**

Res No, He's working at home. So he's working every day anyway. But I mean, you know, even having him for five minutes or something? Erm

INT2 Yeah

Res He's more contact than I would have had previously.

INT2 Yeah. And are you seeing anybody else? Erm like if you've got like a support bubble or anything, like that, on a regular basis,

Res Not on a regular basis, but my family live fairly close. So erm I have seen my mum and my dad erm, you know, maybe once a week or once every other week, something like that. Erm. Obviously, all doing social distancing. And when it was all permitted, but not, not as, not as a erm support bubble. (Just as in, 13:28) whatever, we were allowed to do it at the time that the rules permitted.

INT2 Okay. And do you think because of erm zoom calls and things, are you having more social interactions now than you was before the pandemic?

Res Yeah.

INT2 Yeah. Okay. And do you? Would you like that to continue? Do you think that will be beneficial for you?

Res (I would like, Unclear 13:51) Yeah, it would be a way for me to engage with more people.

INT2 And are you part of any, erm like, brain injury support groups or anything like that either before or now?

Res Yeah. So I do, I did go to erm a once a month headway, erm support group. Erm. Just locally, and then obviously, it started going online. Erm. For me online was a little bit trickier. I think the other thing is that people with brain injuries have a, such a variety of issues. And mine is really mechanical, and it makes me feel very tired and fatigued. Erm. But it's not to do with my frontal lobe. It's not to do with my personality. It's not to do with my inhibition or disinhibition. Erm, and erm so, in those zoom calls, particularly, there's a lot of brain injury

sufferers, it's quite, it's quite difficult. (Laughs) Everyone talks over each other and erm it's not, really you have to find someone who's got a similar, similar issues to you. It really depends on what the issue is as to how people will have felt like in lockdown and erm mine being mechanical means that I have certain difficulties that erm not everyone else has and I don't, there are other difficulties that I, that are fine for me, you know, so erm err I went to the zoom call quite a bit, but obviously I have my toddler as well to going on zoom call in the middle of day with my toddler running around is not so useful. I have to like watch him and erm, yeah.

INT2 (Muffled)

Res Yeah, I didn't always go to them, I don't always need erm. You know, I don't always need someone to help me fill in a form or something that the headway support provides. Erm. It's rather just as umm a community, I actually felt like I didn't really need it, when my husband has been, in lock down, with my husband being at home.

INT2 Before locked down, when you could physically go to the support group, did you find that that was the benefit?

Res It was, erm I think, for me, the bigger benefit was feeling that I was helping other people. Erm giving me some purpose. Because I had to stop working, I had to stop doing lots of things that I might have done previously. And I sort of started lacking real kind of purpose in life. Erm, I know like getting well was like a purpose. But that's like, not really. For me, I worked in the city, I was like, doing full on job. Erm and so I felt like I could, I was quite often more like a helper in the groups than I was necessarily a patient, people would come and they'd be like, Oh, are you you know, oh are you, how are you involved with headway? And I'd be like, Oh, I'm a brain injury survivor. And they would go would what, you know, you look well, if anything (unclear, little human 16:52) is wrong with you.

INT1 That's ah, it's a real turn around there cause erm obviously, the brain injury considers to be a hidden disability, and then even yours, taking it even further to brain injury survivors having a even further hidden disability.

Res Yeah. Well, yeah, I guess so. People, people don't know. Yeah, people wouldn’t know cause they didn’t see. Unless you’re with me a lot of the time, then you wouldn't know there's something wrong with me. Like, I can see just looking at my picture that my eyes are err heavy, and smaller. But you might not know that because you don't know me. And erm, you wouldn't know what was causing it. You might just think I'd had a bad night's sleep. Erm, but this is every day.

INT2 **Erm and I just want to check as well. Obviously, you mentioned that before lockdown. You probably go out once or twice a month for social activity. Pre brain injury. Was that a, was that a big difference?**

Res Oh, yeah.

INT2 **You'd go out a lot more.**

Res Oh yeah, I'd be doing like five nights a week. Erm. Doing either some social event with friends or doing at work social event or doing a social event with work and then go into a social event with friends. Erm, playing sport three times a week. Training at the gym twice a week. Erm it was literally like, a full-on life all the time.

INT2 **Yeah. So very, very busy in comparison to now.**

Res Huge difference socially and erm physically than after my brain injury. And now, yeah, yeah. The difference between pre brain injury post brain injury.

INT2 **What about the term loneliness? What does erm? How do you define that?**

Res Erm (pause until 18:57). I guess, not having any one. Not having anyone to turn to or not having anyone. Erm, yeah, not having anyone to turn to. I wouldn't say, fortunately for me, I've always, even through my brain injury I've had my husband and my family close by. So, I don't think I've ever felt lonely. In that respect, I felt socially isolated.

INT2 **Would you say that was the same before the pandemic when your husband still went out to work? Do you still say that you? You didn't feel lonely?**

Res I think I felt more lonely. I think I did feel more lonely because he was working long hours. And really, I probably didn't have him to turn to because he had the other commitment of being at work. I couldn't turn to him and say, I'm really struggling today. Can you come home?

INT2 **Would you say that your feelings of loneliness have changed over the last 12 months? Do you feel? I know you mentioned that you, you feel better now? Do you think your thoughts about what loneliness is has changed because you've now got your husband at home all the time, or because of the pandemic?**

Res I don't think, I don’t think my idea of what loneliness is has changed, particularly.

INT2 Its stayed pretty much the same two things.

Res Yeah. Yeah.

INT2 Erm and what about REDACTED**? Has there been anything that you say that has helped you?**

Res Sorry, say that one more time

INT1 You’re breaking up a bit REDACTED***, I think. I dunno what, (background speech) You're Charlotte, can you see is*** REDACTED ***screen stopped?***

Res Yes.

INT1 Am I still moving?

Res Yes.

INT2 Can you still hear me, I think I’ve frozen?

INT1 Yeah, I think you froze out for a minute there. REDACTED***, I think you're back in.***

INT2 Oh, am I still there?

INT1 Can you still hear us?

INT2 Oh?

INT1 ***(laughs)***

Res Okay. I can still hear you, REDACTED.

INT1 ***Okay, yeah, that's perfect.***

INT2 **I'll come out and come back in.**

INT1 ***Yeah, that's fine. I'll just mute. So I think what REDACTED was asking there was erm just, erm yeah,***

Res (muffled)

INT1 ***Yeah, just in terms. Yeah, I think it was just in terms of lockdown. One. One thing I'll ask slightly differently, which I was quite interested by. Erm is this idea of erm loneliness. Generally, but if you obviously, you said the moment you don't really feel you know, how you would define loneliness, You don't really feel that way? If you did have that feeling of loneliness? Would you feel able to tell someone or talk to them about it?***

Res No, because I would feel probably that I had no one that I could turn to.

INT1 ***Even when your sort of support network that you had now or that you have now, for example, with your, your husband, or is that something that you wouldn't potentially erm talk to him about?***

Res Um, I think I would. I would talk to him about it, but I don't think I would feel it. Erm (pause). Because I have, yeah, in my, in the in the sort of definition of it that I've given it.

INT1 ***Yeah. Yeah.***

Res You have no one to turn to. It's sort of like erm

INT1 ***it's more of a physical aloneness,***

Res Therefore indicates? Yeah, like I'm saying, yeah. And a deep feeling that you're by yourself.

INT1 Yeah.

Res Where I don't have a deep feeling that I'm by myself.

INT1 mm.

Res Because I think that there's always some help for me available if I would need it.

INT1 Yeah.

Res Erm and I well, that feeling of that you're totally by yourself. And you have no one else you can turn to. Erm and loneliness. I would. I would equate with being with sadness also. Erm. I'm not sad at the moment.

INT1 Mm-hm

Res I don't think

INT1 (Laughs)

Res I have suffered from depression over the period of since my brain injury. Erm But I think that's more to do with er loss of purpose and grief.

INT1 Yeah.

Res Of my life than to do with loneliness.

INT1 Yeah. Okay. I think just before, so REDACTED your back now. I think just before you erm froze out, I think you were asking about lockdown specifically. Erm but it was hard to, it was hard to figure out what you're saying I think but I think it was in relation to erm if we

think about the lockdown over the past year, what has been potentially the most helpful things? Erm I think you've already touched on it. But I think it'd be good to know I think what you think would be most or what has helped you most through lockdown over the past year.

Res What's helped me over lockdown is being prepared for not having a social life. Erm, in terms of if I were facing lockdown, and I didn't have a brain injury, I would have found it harder. But I had a brain injury. And so I was very used to what lockdown felt, feels like erm so the sense of what's helped me, that would be what I would say would help me if I hadn't had a brain injury in terms of the fact that I have had a brain injury and what's helped me, I think it's probably just having my husband at home erm. Specifically in terms of lockdown. I think the other thing is maybe being,erm being somewhere where we where you can go for walks and things like that err outside, taking joy from nature and being outside. Erm I mean, for example, like I live in West London, and err there have been less planes going over. So that's to positive cuz erm this noise, theres pollution, all that sort of stuff, but yeah, for me particularly it's like less noise. There have been less cars around, lovely because I don't like movement, seeing cars go past sets off my symptoms, generally less people around when you go for a walk. Great. Erm (chuckles) and ermthe other thing maybe it's just that everyone knowing that everyone else is in the same boat. Like, no FOMO

INT2 Yeah, I can understand that. Can you hear me again? Am I my back?

Res Yeah, your back.

INT2 Brilliant. Yeah, totally understand that. are you managing to, Did you say you would get out for a walk quite regularly?

Res Yeah, go for a walk once a day. Yeah.

INT2 Did you find that helps?

Res Yeah. And that was something that I had to start doing more often my brain injury anyway, that is all I can do is go for a walk. I can't do any other physical exercise or sport. Erm. So, it that, I think is something that most brain injury sufferers talk about being something good being in nature calming, erm it tends to not stimulate your issues. Erm so for me, it is tiring because it's, you know, you're walking and you're using a balance. And that is something that is tiring for me anyway, so I can do what I want every day now. I mean, I have been walking, I think I was like doing like 80 kilometres a week in lockdown. I mean, like a lot of walking. Erm.

INT2 Impressive. (laughs)

Res I know, probably, probably more than most people anyway. I don't know.

INT2 Definitely. It sounds like locked down has been relatively positive for most aspects. Has there been anything that you have found particularly difficult?

Res Yeah, I think the thing I've found the most difficult is the people who I have seen, like complaining about it. Erm. I found that really difficult to stomach. Erm. It shows to me a total.

INT2 In terms of sort of, ah sorry.

Res (muffled speech, 27:20)

INT2 In terms of social interactions, were you meaning?

Res Erm. I mean, erm people's mindsets. I found it really frustrating that people have the mindset that I can't do this. I have to break the rules. Erm I can't do it. Erm. You know, this is this is awful, blah, blah, blah. Erm largely because. You know, people saying what about my mental health? Yeah, of course, it's an issue. But no one said that to me. When I err went through my periods of depression after having my brain injury, they were on the sympathetic to me. No one called on me to check I was okay. Erm Yeah, just those things really, that I found particularly difficult. Erm I think we might have lost REDACTED again. Erm.

INT2 I'm breaking up a bit. Can you hear me or not? (disrupted connection).

INT1 I'll take it from a REDACTED just because you're a little (disrupted connection)

INT2 Are you sure?

INT1 Yeah, no problem at all.

INT2 No worries, thank you.

Res Erm And also people when every lockdown ended. Erm People suddenly wanted to go out and see everyone and it made no difference to me when locked down ended, it's still, I'm still in the same position that I was in lockdown. I still can't really go to the pub, I still can't really meet with a group of people. Erm. So frustrating for me that suddenly everyone who wanted to go for a walk, which is what I can do, suddenly now wants to go to the pub. And no one says you want to still go for a walk with me. Erm. When I say to them, actually I don't think I can do the pub or whatever. They say okay, well, maybe next time, rather than saying, Do you want to come for a walk instead? What would be easier for you? Yeah. But that I think is a problem that most brain injuries sufferers have is that people don't understand what the issues are. And everyone's got their own lives to leave, lead and do what they want to do. Erm. We just get left behind.

INT2 So do you feel with obviously things now, like you said, like moving out into this post apocalyptic lifestyle that soon we'll be living erm, once locked down lifts legitimately and how do you feel about that like heading back, like this idea of you know, reintegration into into society. Is it something that you're more anxious about now? Or is it something that you just feel like things will go back to normal.

Res Erm, I'm not anxious about it, because I'm just probably like, I mean, in terms of like COVID risk. Erm I had read research and white papers. And as it was coming out on erm the effect of COVID, on erm your nerves, the, you know, the the brain's nervous system and things. And I knew that there had been some issues associated with that, you know, the basic thing of loss of taste and smell is to do with your, your erm, your brain and your nerve system within that. So I was more anxious that if I were to get ill, that it might affect me more badly than other people, or it might take me longer to recover. or in any case erm, you know, you've gotta look after a toddler as well. And it would be really 'bloo', t's hard work anyway. (laughs). So be really, bloody hard work. And so that was, I think I was of a heightened concern, maybe then other people of my age and demographic, and because I have my brain injury. But I'm not more worried about that now, particularly in terms of going out socialising and that type of stuff, because I don't really do that anyway.

INT2 Yep.

Res And that's not going to change for me, what will change for me is that I will realise again, that people don't really care (laughs) about me.

INT2 So one thing that

Res if you see what I mean?

INT2 ***Yeah, completely, I completely understand what you're saying. One thing that you have spoken about is how positive it has been having your husband at home? Is that something that obviously with things now opening up a bit more? I'm not sure what your husband does. But is he, is there a potential for him to be going back into work more often now?***

Res Yeah, there is. And I'm not looking forward to that. That is for sure. Erm I don't think that he'll do full hours back at work, err probably to two or three days, you know, back in the office. Erm but I'm definitely not looking forward to that at all. And going back to the idea that I'm being pregnant again, poorly again, and dealing with the toddler again, all that sort of stuff. Erm no, yeah, I'm not looking forward to that. So that is that maybe makes me more anxious, this idea that he's going to be going back to work and, you know, and appearing at nine o'clock at night or gone that. So I'm alone all day again. Erm but we'll see. Yes, I might have my, I'll have my hand is totally full anyway for a while. So I don't, cant think about it for a while.

INT1 ***No that's good. Erm I think that's everything erm that I have to ask, I don't know, REDACTED do you have anything, any additional questions?***

INT2 **No, I think we've pretty much covered everything. Erm that I can think of as well. So yeah, I'm good.**

Res Can I just say one more thing, that I think a lot of this is a lot of it is less about lockdown, per se and I but I know it has affected a lot of brain injury suffers a lot. But for me, it's probably more about an, and for. For others. It will have been more about our lives being the same. But the difference is to other people's lives. And how that's been erm err accepted or not accepted? And do you see what I mean?

INT1 ***This is something that that we've heard. I mean, we haven't done a huge amount of interviews so far. But this is a recurring thing that is coming out at the moment of these interviews, is this idea that the change is for the general population? Erm not for brain injury survivors, per se.***

Res Yeah. And erm, and I think that speaks more to how brain injury sufferers or survivors feel post their brain injury and err less to do with lockdown, rather to do with the brain injury.

INT1 ***Yeah. I mean, does it just feel like based on the conversations that we've had, that? Erm. Yeah, you know there's this, there's been this raised awareness, I guess, around how, like, headway are running this, this campaign really around, you know, life in lockdown, which is***

Res Exactly

INT1 ***which is, you know, that is the experience that, that people have experienced over these lockdown periods. But generally, that's what brain injury is full time.***

Res Yep 100%

INT1 ***So that's the interesting thing. I think that's that's the interesting thing that we're trying to tap into here is this understanding of, well, is that the case across the board for brain injury survivors, how is it How does it manifest? What types of feelings? is this related to loneliness? Is this a legitimate theory or, you know, just trying to explore this idea a bit, a bit more I think and it is really interesting. I think as an outsider, you know, someone who hasn't had a brain injury and doesn't understand, erm I can't say understand those experiences. Erm to hear, you know, what kind of, effectively the kind of things you've been saying around, you know how people treat brain injury survivors if, if it's advertently, or inadvertently,***

Res Mm-hm, I mean, for example, my mother in law said to us when lockdown ended and we didn't suddenly go out loads, she said, I think you two are just becoming really anti social (chuckles). And I was like, Okay, number one thing you shouldn't say to someone who's got brain injury, because it clearly is really a misunder, massive misunderstanding of what brain injuries are all about. Erm and then she said, erm, Well, when I said, you know, it's obviously tricky for me to go to events that, you know, gatherings with you all, you know, it gets rather raucous. And they're difficult for me anyway, at the best of time. So given its lockdown, or whatever, or limited social interaction, I think we'll, you know, we'll just sort of, you know, keep our distance and come to the ones that we can come to kind of thing. Erm and erm she sort of said, well, would it be better for you if we just didn't invite you?

INT1 ***Wow.***

Res Yeah, then you wouldn't feel like you have to say no. erm and my husband afterwards is like, well that's kind of what the Disability Act is all about, you know, not excluding people who have issues, but that is what our life is like all the time. Erm and it's more like an anger about that. Really, I guess people who have brain injuries feel that, and I'm kind of like, have no sympathy for people who are being erm mardy about the lockdown.

INT1 ***Yeah, I can completely understand that. I can, I can absolutely see that and***

Res Just suck it up. I've sucked it up for years. Erm get on with it. You'll be back to normal life soon. And I'll still be in the same situation I was.

INT1 ***Yeah, yeah. This uh, yeah, it's an interesting. Yeah.***

Res (chuckle) I thought that might be different. Maybe from what you had heard from a lot of brain injury survivors

INT1 ***No, no, no, no.***

Res For a lot of people, like the same.

INT1 ***I think you'd be surprised. Yeah. But like, we, like I said, we haven't had many interviews. And but this is the prevailing thing is that, you know, welcome to welcome to world of we, that brain injury survivors have lived in ever since their brain injury.***

Res Yeah, yeah

INT1 ***And yeah, so yeah, this this, this is definitely the prevailing attitude is, and haven't heard, I haven't had a different attitude. so far. Let's put it that way. So.***

Res Right ok, it,s so interesting. I feel like I'm fairly eloquent in describing things.

INT1 ***Yeah you are.***

Res And you know, as I said, like, my temporal lobe and all that other stuff hasn't been affected at all. And I'm, you know, highly educated and professionally educated and everything, I can really quite easily see what the issues are. And erm err that's why I want to be part of these studies and things. Erm. Although my brain injury isn't as bad as a lot of many other people.

And mine is mostly mechanical. Erm, but that gives me a different kind erm of perception and view of things that err cerebrally is still all there.

INT1 ***Yeah. Nice, honestly, it's been err. It's fascinating to hear, it's just, it's really, it's really interesting, from our perspective to be able to, I mean, because brain injury is different on the whole Anyway, do you know what I mean, so it's, it's going to be a case of, we're trying to speak to as many people as possible with a whole range of issues and erm sites and locations of brain injury as well. But as we're doing that, these are the same things that are coming out. So that's the fascinating thing about it, you know, just because, you know, you potentially don't exhibit maybe as much erm in terms of a physical disability erm. It doesn't mean that you're not feeling the same thing as a as a brain injury survivor. So it's interesting,***

Res Almost all brain injury survivors will suffer fatigue, erm and things like that, erm which affect you on a daily basis. Erm and then along with other things, erm other symptoms, so we'll all be having the same challenges. Erm that lockdown largely took away.

INT1 ***Yeah.***

Res Or made a, made a level playing field.

INT1 ***Yeah.***

Res For everyone else in the country.

INT1 ***Yeah. Well, it's nice. It's honestly this is really good to talk about***

Interviewer(s) **INT1 – CB**

INT2 – SD

Respondent: RES (P4)

INT1 **So if we are ok can we start with just finding out a little bit more about your brain injury I know you sent an email erm to SD sort of** composer **detailing what had happened, can you tell us a little more about how that has affected your life?**

RES yeah.. erm… ok what happened was I was, well I call myself a composer, right and what happened was I had performed at a … club and erm I was assaulted erm one my partners ex beat me up and erm… knocked me out and when I came round some point later, one of his drug dealers was sitting on top of me and had shoved a hand full of some kind of pills in me mouth and that knocked me out for a couple of days. When I came round erm the bloke who owned the building where the studio were is where is panicking he is in my face there (saying) "breathe Sam breathe breathe breathe" and erm well I didn't, I didn't know what was going on then. I was like "who are you where am" I sort of thing and I made it back to my partners house err somehow well her house was quite easy to find it was on the end of the terrace next to the canal so it was easy to find her house and erm made it in there. That was over a week later and she was furious with me coz I've been away for a week and this was in erm 19th July 1998 when I was assaulted and erm we'd had a, we'd bought a (unclear 04.14) she was furious with me for being away without telling her what I was doing erm rather than sort of getting me any help she, she was just you know just angry with me she thought I'd had been out enjoying myself and erm I didn't know what was going on and… erm yeah… I thought I'm done I erm I sort of as well I'd been erm traveller they used to call us like hippie travellers, I used to live in a thing called a bender erm which is like a …erm you know do you know what I mean,…. Yeah? I'd lived in a bender for 11 years,

INT1 **Yeah like a bus type of thing**

RES But I'd been staying with her at that time erm ….well no it's made out of, you make a frame out of hazel poles that's sort of that shape you bend hazel sticks over like that together and, and make it is shaped like a basket like upside down basket erm traditional travellers homes erm then you cover it in a tarREDACTEDin erm which is waterproof and erm I used to make me own wood stoves and I'd lived in one of those for 11 years people on those sites live in buses and lorries as well yeah and to be like that the peace convoy and and now yeah and erm at one point I had a pony as well as a pony for nearly three years and a little cart erm…But when when Nikki had Martha our daughter I'd been staying in her house sort of looking after them and you know sort of being a dad and erm anyway she thought I'd been out enjoying myself and didn't do anything to help. But erm I was doing things like I'd walk into the wrong house and go into town and get completely lost in places that I knew like the back of my hand and people walk up and start chatting to me and I didn't know who they were I forgotten everything completely. And I didn't know the words for things, you know. dysphasia really bad dysphasia you know, I'd sort of not just people's names, you know, I wouldn't know the word for anything, and err yeah,

and I have just signed up to do a degree as well within the first two years in Burnley and the last one in Preston is it Preston erm yeah which I had to postpone erm.... And yeah, stopped I had to stop living in the bender. Because I couldn't cope with living like that anymore. Because like you have you have to be really organised with like that people think people used to think that we were all out to lunch and and really disorganised. But you have to be really organised to live like that properly. Otherwise, you can't can't cope, and err why some people are really out their brains on booze and stuff like that. But you know, to do it properly, you've got to be really organised. And er, yeah I used to do things like, before that I used to go to council meetings and, and participate as a member of the public and sort of make suggestions and things like that. And, you know, I was into permaculture. Yeah. And I was trying to do something positive. Yeah, I'd started erm… erm the recycling around here, which is called curbside. My proposal made that agenda 21 and erm… other stuff, you know, yeah. There was there was sort of little band of us around here that were sort of not quite anarchists but, sort of you know (laughs), trying to change things a bit. And err yeah. anyway, it all stopped. You know, I didn't know what hit me. And erm I had to start living in the house. And so yeah, I was, I was walking into the wrong house and people go, "who the fuck are you what are you doing in my house?" And I was like, "Oh, shit, sorry." Yeah, I think any help from anywhere at all? Yeah, but I didn't. I didn't I wasn't asking for it either. You know, I didn't know what hit me. And erm as well, I was a single parent. I had two children. This was 98. The youngest well the oldest was born in 92 when the youngest was born in 93. And then, there was another one born in 98, as well, but I wasn't a single parent. So I don't know how we all coped. It was like I don't know how I did it. Erm I don't know how they did it either but we did somehow. Yeah, and I couldn't read or anything. I couldn't do anything at all. We were surviving somehow and yeah

INT1 **And have you been to the hospital**

RES no no

INT1 **And at that point, had you sought any any medical erm advice at this point,**

RES people would walk up to me in the street and chat, start chatting to us people I've known for years, and I just wouldn't recognise ......any what? No

INT1 **Have you been to the hospital? (unclear)**

RES No then that like the what happened that I got started getting medical help when erm we were booked to go on a holiday, we'd booked a erm rented a cottage….. And we got a bad connection. It's really garbled what you're saying.

INT2 ***I think there's***

INT1 **I'm not saying anything**

INT2 ***There's a delay. So I think one thing you have to be mindful of Sam, I think is that***

RES what did you ask?

INT2 ***I think I think there's a delay. So I think whenever we speak it, there's been a pause. So I think that***

RES There Yeah, so I think because about 10 seconds.

INT2 ***Exactly. So I think one thing we'll try to be mindful of is we'll let you speak as much as possible. And then we'll, we'll come in, but there maybe it may feel like there's an awkward tumbleweed pause moment in between that. But I promise we are probably asking a question. It's just taking a while to get to you. You still there, Sam I'm really sorry. I've done it it again.***

RES Yeah, perfect. medical help came, we'd booked erm a few weeks after the July 98. We'd booked to go on holiday. In Robin Hood's Bay we'd booked a little erm a little cottage and we stood on Hebden bridge railway station. And the next thing I knew was I was in Calderdale hospital. And they're going "how long have you had epilepsy for?" And I was saying, "I haven't got epilepsy". And they were going "Yes, you have mate". And err yeah, so then erm So then I started to getting treatment for it in they tried me on loads of different drugs. I've been on loads of different tablets, and none of them really work. I take topiramate the moment, but I still have seizures. I probably would have more if I didn't take them. I still, I still have seizures now. You know? And I'm not going to stop taking. But yeah, it's Yeah, I've just asked to be transferred to a different hospital as well, because they don't, they don't give me any support. And they're supposed to give me four points of contact a year, but I only get one. And when, when I spoke I was well I've been when I found headway the brain injuries charity in 2019, that's when I realised what had happened to me in 1998. Because they do really good information sets that like they do little booklets and little leaflets and they sent me the full the whole lot. And I read all of that. And I

realise then what happened to me in in 1998, which was, well I'd stopped breathing. And the hospital should have told me that in 1998 they should have realised that the penny should drop with them straightaway. Because I told what had happened. So really, they've been negligent with me. And when I tried to talk to them about it, and they all clammed up (chuckle) and acted really furtive and you know, they're worried which they shouldn't be they should, they should open up and you know, and act professional. Now wasn't wasn't going to sue them, but I think that's what they're worried about.

INT1 **Yeah, I can see (unclear 14.29**

RES I still have pretty bad memory problems now? you know

INT1 **And if you had any rehabilitation at all, since presumed since 98, you didn't because you've only just discovered and What about since anything at all,**

RES Nothing, nothing at all.

INT1 **Okay, and in terms of your, your life before erm you were attacked, you, were you working then and at your work and sort of in the community and things like that. What about now? What kinds of things are you doing now?**

RES Now I do erm well before erm the plague hit us I was doing loads of erm like a conservation type volunteering. I was going out with err councils countryside department volunteering. I was going out with a erm group called CROWS community rights of way service, which is fixing footpaths. I was going out with erm something called slow the flow which is putting in flood defences, to stop Hebden Bridge flooding. I used to go out with erm at one point with erm... up at erm....what do they call it? Hardcastle Crags like they do, they're pretty err. They didn't treat me very well over there. And as well, they couldn't cope with the fact that I had epilepsy one of the best groups I've been out with is TCV erm used to be called British cars for conservation volunteers, but that may get into Leeds, getting to Kirkstall everyday. Well, for all three days a week, but that they're brilliant. TCV really good. And as well, you know, the train, you, and give you qualifications out, as well as you go out and do in like loads of really varied volunteering, and as well then publish these brilliant booklets, which err I'll show you one these right there, sort of A level quality booklets that teach you how to do things like hedge or tree planting or fencing or footpaths or dry stone walling or all sorts of. They're really good. And yeah, I like going out with TCV. But that yeah, that meant getting Leeds. The other good thing about TCV is it's in the same building as Permaculture Association, which I'm a member of. And you know, I could just pop in and have a chat with Andy

about, you know, whatever is interested in that week. Yeah, I did, like, what else I used to volunteer at home with Late Well, I used to cycle a lot, but the epilepsy stopped to that. And one of the places that I cycled was Hollingworth Lake, and it was a mess. And I popped into the office and said "'scuse us if you give us a pair of gloves, I'll pick up litter while I'm here". They ended up giving me a job as a volunteer Ranger and I worked there for about two years. I loved working there was flippin brilliant, but then one day, I had a seizure while I was cycling and fractured my collarbone. And they found out that it was illegal for me to work there in the cycle support worker. (wouldn't) Let me go back, which was crap. Yeah. And the more I find out about epilepsy, the more I find that what I can and can't do.

INT1 **Yeah,**

RES then as well as volunteered for the Yorkshire Wildlife Trust. erm They've got they've got virgin woodland. Erm two bits of virgin woodland left in England. One of them's just here at (unclear 19.59) which is a fantastic little bit of woodland. Yeah, there's it's not been touched since the Ice Age. But the place I only been there once ever. The other place that I used to go was erm, erm the one in Grassington I've been there seven or eight nine times maybe but that I've got somewhere to stay there. I don't I don't know why when this often but for me, mum lives about two miles away. So I'd go and stay with my mum and then go and volunteer the next day up at Grossword and Grassington which was a bit daft and then I only I didn't do that much with Yorkshire Wildlife Trust. But yeah. It was all when I was cycling all of that. And err

INT1 **Yeah, you sound like you really enjoy outdoors in nature and being free. How has (lockdown unclear 19.55) it impacted you sort of in being restricted a bit more.**

RES (sigh) It's just like being in jail in it a little bit. Yeah. erm right. Well erm, me and I've got one friend in the village. And we formed what what did they call it

INT1 **A bubble.**

RES That was a bubble. But then one day he was taking the mickey out of me, right? So he's taking the piss really badly out of me one day, and I said, "look, stop it or go go home." And he's not been back since. So it was like, Okay, then. What? What was the matter there? erm Yeah, yeah.

INT1 **Anybody else that you've had supported you? Who have you had erm sort of seen regularly? Or who were you living with?**

RES No, no, nothing. I'm supposed to get four points of contact of epilepsy team a year. And a it's it's a bit less than one. Yeah, they used to get two off me Sapphire nurse. And two off the consultant. Yeah. But now when the old Sapphire nurse retired, the the new one came along, and she used to make the two and then cancel them. And err I'd only met her once in the first two years. Erm and then and then radio four what was it? call you in yours having a phone in about the NHS, I phoned in anonymously and said that, and she must have been listening and recognised my voice. And erm and the situation because they didn't say where I was or who I was? Yeah, she must have just recognised what I was talking about rang me up the next day and says "not your Sapphire nurse anymore. You just see the consultant in the future". Bam, slammed the phone down. All right, okay. Erm and the erm consultant makes me one appointment a year, instead of two and they always ring me up about a week before and postpone it. So, I actually get less than one appointment a year. But you know, is really, I think it's because they're flippin overworked. Yeah. So I'm torn between thinking, well, it's not really their fault. But (laugh) it's me who has to pay for it. You know,

INT1 **Yeah**

RES it's by not getting as well. I'm getting brilliant support off headway the charity, peer support. That's flippin brilliant. And erm Healthy Minds. Again, that's peer support off charity. What's the other one? erm... Sure there's another one. Well, I mean, I'm, I'm part of epilepsy action. We don't have err support. But you can phone them up and talk about stuff. Have

INT1 **and were you having a peer support before....**

RES It's not peer support. But there's, I'm sure there's another session next week.

INT1 **Were you having peer support before lockdown.**

RES Well, I was, I was I was having peer support before erm.... Yes, but it takes on more value, don't it. When? Yeah.

INT1 **And what about erm any other support networks sort of friends or family on as well?**

RES As well we're doing it on Zoom.

INT1 Yeah.

RES What? When I had my brain injury, all my friends just vanished in 1998. And I used to have loads of friends and used to smoke pot. When I lived in my bender. I used to be a dope smoker, didn't used to drink booze or take other drugs, apart from coffee. And people would come and we'd all we'd smoke pot together err didn't used to smoke tobacco either. Yeah. And when had the brain injury, all my friends just vanished completely. erm And I thought oh, well they weren't my friends then obviously. erm... And that.... yeah... that was it created isolation, that for like years. You know, I was just sort of sitting there thinking, Oh, I thought they were me friends and where have they all gone. And erm I was, like, even the ones that have made music with erm I've got a roomful of musical instruments here. And they used to all be in, in the room with other people's musical instruments as well. And we used to do things together. Erm.. And they just all vanished. And I couldn't think well, why, you know, what, what? What? Well, there must be a reason. And err so, yeah, no I have changed. But sort of what's the like, the inside of me's? Yeah, I've got brain injury, but I'm still the same me inside. You know, it's err... And I sort of don't sort of live the same or do some of the same things. But I'm still me, you know, it's well, what where've they all gone? So okay, to let go of all that. And erm… yeah…. and I still don't quite understand that. As well, I used to, I used to help a lot of me friends erm they knew they could call on me if they needed me. And why it wasn't two way. Which I never got quite to the bottom of erm So what happened? No, they weren't me friends. Really? They were acquaintances? And that there's a couple who still are there?

INT1 Yeah,

RES just a couple, not dozens (laugh)

INT1 Yeah. Find it. It's quite common with with brain injuries, that sort of friendships change and evolve. And it's quite common to lose quite a few friends for some reason. It's, and I can imagine it must be very hard when you're sort of erm trying to find yourself again, and then not have support. But you said that the peer support from headway in the charities that's been really good for you…..Are you still there, Sam.

RES Yeah, but it was 21 years, until I found headway.

INT1 **Yeah, it's a long time. Now. How did you manage in that time?**

RES Yes. I'm still here I said yes. It was 21 years until I found headway

INT1 **Yeah,**

RES yeah. Yeah. I'm not sure how to manage but I mean, yeah, I'm still here. But it's....

INT1 **hard?**

RES ……….You know, there was a lot, there was a lot of a lot of blank in there. And, you know, a lot of sort of sitting there wondering, flip or hit me, you know, there was a lot of it where it was no, it was probably like 18 months old again in there and rebuilding like that, I think. And, as well, right back at the beginning erm. In 98, I'd signed up to do a degree and they said look, take a year out and come back. erm come back next year. We didn't know what happened then. I did. And the degree was music and the creative arts and performance technology. And the creative work I was doing was getting erm a lot of it was getting the highest marks out of everybody in Burnley and Preston and my written work was getting the lowest my music was getting really high marks though it was making people sort of think differently and I did a what one of the briefs was make a we had to make a piece of music using erm… an existing piece of music and everybody else made a pop tune and then I did like some, you know erm something a bit different using err .. erm... it was like you know, some Misty Anne might of done. You know if I ripped up a phonebook and played tune using a wineglass and err, you know, it was one of the themes out of Handel's Messiah and said well I ripped up a copy of Handel's Messiah and in then you could hear, matches, matches in a roaring Blaze, you know, and it was called ripping up Handels Messiah and burning it and there was a couple of little bits in there. Everybody has just made the sort of, you know, fairly average, opportune, you know, and nothing stood out except mine, you know, and even if I say myself, and that's what they thought as well, you know, but but all of my stuff really stood out. And err yeah, and then we had one of the other things we had to do was make a CD, a CD ROM and everybody else made the CD ROM. Like, like that. Mine was five foot square made out of cardboard and things sown on and err you know, like a museum ex exhibit (laughs) from for a book I made the video to put on there and pulled all the tape out of the thing and sowed it on.

INT1 **What about your partner? Are you still with her?**

RES No, she she's, like I said, she thought I'd done it all to myself. She went off and had sex with somebody else. And so it just sort of left her it to it. And yeah, I went in started renting. my own house. And while we mean we were..(unclear 32.01). When Martha was born, our daughter I'd sort of been staying with her to give her support. But erm yeah, she was er..... Yeah, she wasn't err. It wasn't two way. So I just left her to it.

INT1 And who were you living with at the moment to live by yourself?

RES Yeah, it wasn't a relationship. It was it was a dependency. Yeah.

INT1 So if we look at lockdown, can you tell me a bit about erm sort of what you've been doing through the days how you've been filling your time?

RES Yep. I've… erm well, right after we get up, in lockdown, I've started waking up really early in the morning, like I wake up about 3am in and usually erm.... when the alarm set for 520. Yeah, that's when I usually that's when the alarms always been set for for years. And erm I started waking up at 3am, which is a bit odd, I found an explanation for that. And erm... all the volunteering is stopped. But erm some of the volunteers are still going out in sort of twos and threes. But of the themes aren't going out yet, so I have not been doing any volunteering. erm Some days, I'll go out, I will go out for a stroll, or erm when erm I've been doing erm... nature in mind is one of the err group, group things that we've done. And so we sort of go out and sort of observe things and go and tell them what we've been doing. Do it every day, what else? Would get up have my breakfast, sort of have a family breakfast before six o'clock. Take my tablets have to be pretty organised about taking the tablets. Because if if you do do them all at different times, it's err you know they advise you to take them all at the same time. Every day, they're Topiramate. Erm, part of the observing is, I've got a decent pair of binoculars and I'll sit here and look out the window with them. Which you know, it's it's not much but you know, I've got a decent view out the window. And I've got err loads of musical instruments in here and fiddle with some of them. I've got a decent electric guitar, I can just plug into I've got little box, I can plug headphones into. I've got a nice preamp. I've got I've just bought a keyboard, which I've got to the bottom of yet. err And I don't claim to be proficient musician. But it's the composing I can do. Erm… What else? When I'm bored, I do. erm what's it called? Sudoku. And erm what else reading, I used to read when I was younger, but when I had the brain injury, I couldn't I can't sort of. I can't read properly anymore. But textbooks I can manage, but I can, I can't manage novels erm that, that went with the brain injury. Erm and I think that's to do with memory. When I go in for a stroll, but again, if I, if I go somewhere pretty wild. And if I have, if I have a fit in the middle of nowhere, I'm in trouble. So, I don't so I use in the old days, I'd go. I'd go for miles and miles here. But don't dare do that anymore. You

know, I'm in trouble with if I ever fit in the middle of nowhere. erm excuse me. I'm just gonna go for go to loo for a pee.

INT1 **Yeah, no worries. … Hiya. Welcome back. Erm Is the vast majority of social engagements that you're doing at the minute is all online, are you seeing anybody? Erm face to face since you you broke up with you, bubble person? Have you replaced them?**

RES No, everything's online at the moment. So on zoom.

INT1 **Yeah And have you got any other support that you see in on a regular basis?**

RES No, no, I've never had any at all.

INT1 **And then if we can just talk a little bit about erm loneliness. How would you define the term loneliness?**

RES Erm… What is lack of lack of any other any other person to, to share sort of thoughts and feelings with or communicate with. as well, now that I could phone people up, but as well, it's all a lack of lack of love. The lonely of it is sort of when you think Oh, you know what I could phone them and sommat stops you from, from doing it as well?

INT2 ***Is this a feeling that you felt Sam yourself?***

RES Yeah, yeah, I know the feeling Yeah. But as well, like I lived in this in that bender thing for 11 years. And err a lot of that that was just me sort of in the middle of nowhere. And I liked it as well, some of it er, I couldn't cope with doing that now. Err But I did like it but as well I liked it when there were people around. erm It depend who. The best when I had the bus, you know, I loved that but, I ended up in North Pennines, I'm set up in Mennhead, Cumbria and around there as well. erm we ended up we ended up in the west coast of Scotland just to buy some Land and got gazumped. Erm I think that somebody sort of didn't want travellers living on there. I didn't have a horse then we had a larbian(?) caravan and my partner lived in the caravan and I lived in a bender, and err that's. We had Annaise the oldest had been born 92 Feb 92. And Maive was born in obon in erm May 93. Erm and yeah, it only rains a lot up there, so you've got to like rain. But we did, we'd love to have that. And we came back down, came down to the Pennines. And

INT2 ***just to sorry, Sam, just to circle back to one thing.***

RES Couple years later,

INT2 ***just to circle back to one thing that you must. Sorry, Sam, I know that we've got this delay. Apologies. Just to circle back to one thing that you had said. When you were talking about loneliness in that the first instance, you said about calling people, but something stops you from doing that? Can you can you describe a bit more about what it is that what you would describe as like it stops you from potentially reaching out to people? Or what is is that a feeling? Or is that a thought process? Or? Or what is it that?***

RES I'm not sure? It's it's not knowing what to say and not knowing what you want? I think and that's about... I don't know,

INT2 ***Out of interest? If you were for example, if you did feel lonely? Would you potentially seek help for that? Or would it be something that you would try and get through on your own?***

RES If I did feel lonely? I probably wouldn't know what to do to be honest, to be honest, I would Yeah, I wouldn't I'd erm....I mean, I mean, like nowadays, I would probably I would probably I'd probably go and look but in those days I wouldn't know what to do.

INT2 ***Just in relation to obviously you we're talking about your daughters as well. Do you still see them regularly?***

RES I'm still yeah, I'm still in touch with them. Yeah, but one of them the middle one Maive, she's got depression and she lives in Tommerdon. Ermm She can like she doesn't always answer the phone because of that. Annie is the oldest one. She lives in Leeds and erm she's, yeah, she's working in Leeds and erm so got you got a ring her at the right time. And erm she can be sometimes sometimes she's really grumpy with me. But their mum died a couple years ago. I think, was it a couple years ago? Erm. So, I think they're both still upset about that. And Martha, the youngest, we get on really well. Erm..She's down. She's down in Brighton. And she, I think she just finished her degree….Yeah. erm… But when I was, yeah, we all when there was a single parent. Well, we were all yeah, we're like, really close family. You know. And err it kinda. I mean, not, I don't know what to compare it to now. I was trying to be

	a better dad than mine. Than mine had been in some ways. And errr..... yeah..... because while I had my stepfather who was quite violent, he used to, he was an ex army captain, and he used to have quite a punch. Yeah. He was quite a bastard. (laughs) And he was a bit of a crook as well. He used to do erm private tuition. And he didn't pay tax on it. And we errr, we have to keep quiet about it. And as well, he would grope some of his erm. Some of the girls he was teaching I don't know how much further he went. Erm… year.. So but he’s dead now. So I've got trying to get forget all that
**INT1**	**And then, we mentioned about sort of loneliness. And you as you was talking, erm do you think you've experienced loneliness over during the lockdown? Or have you changed, your feelings changed? Do you think? during lockdown?**
RES	Erm... Well, yeah, but not sure if it's any more than than normal, you know?..... During other times, like because I'm going to these peer support groups on zoom. So you know I've got have got contact. So if, and if I want contacts I can phone people up
INT2	***One thing that you did touch on earlier was the peer support. And you did one thing I wanted to ask you a bit more about was erm you said that, obviously, the peer support from places like Headway and Healthy Minds has still been going on erm during lockdown, but you said that it means more. So do you think that there's a difference between the peer support you're experiencing previously and like during lockdown instead is locked down the difference effectively? Like do you find that the peer support is more beneficial during lockdown? Or is it just as beneficial?***
RES	.... erm...What what one thing about especially about the headway thing is it means I don't have to do all the travelling.
INT2	***does that actually easier in to some extent to actually get that support erm via obviously online methods rather than having to travel for it.***
RES	yeah.. But I'd rather I'd rather see humans than pictures
INT2	***So it's sort of risk reward sort of the balance***
RES	I'd rather do I'd rather do all the think I would rather do it all. And yeah, yeah means I've got to pay for the train or me, I've got a free bus pass. I still when I go to

Bradford I still use the train. Which you got paid for. but erm Yeah, it's I think I'm pretty sure it's got more, it's great. It's gotten a…. that's quite hard question really, because it's, it's still it's the same. It's the same peer support group. You know, it's, it's, we don't, we don't do any, and we don't do anything different. It's just that with, you know, there's there isn't any other social thing thing happening thing. So that's all there is really And err that's what gives it the increased value is what I'm getting.... you know... Otherwise, what else would there be? Um, what else would I have options to do? Because I don't go into the pubs or anything like that or... you know... In the old days, when they used to make music, …. there was there was er, some of the DJs that they're always connected with. They were connected with various clubs. I didn't go to them and unless I was performing, you know, I didn't go out for the for the fun. Really, I've never been sort of, like going out type. And err never been err going to the pub or going to the club type. And erm living on travellers sites, they're, they're quite, they're like families. And when you get ones the, that you don't like living on you just move and go to go and find another one. Or you just go and set your own one up, you know, and if people turn up that you don't like, you just move and the I liked living like that. You can't do it. Sort of it's difficult to do with a condition like this. And I'd stopped living like that anyway, before. Erm... Well, no, no, I say it stopped living like that before I stopped living like that when I was assaulted. And but I was I had I had a Bender in the field that I'd been given permission to live in. And I had the studio in a house about a quarter of a mile away when I was assaulted. And sort of I was I had.. two of my daughters were coming every weekend. And err yeah, and that's when it all fell apart. And then then I started renting a house. And err two daughters are coming alternate for seven days. And then to their mums for seven days. And the youngest is coming every weekend and already in the holidays are staying for the same seven days as the other two were there which err I don't know how we did it. When I look back but we did somehow

INT1 **Talking about lockdown specifically what do you think**

RES I've lost the thread of what I was waffling about

INT1 **(laughs) It's alright and talking just about lockdown.**

RES Go on

INT1 **What do you think has helped you? what's helped you these last sort of 14 months is it we're at now.**

RES Coffee.....

INT1 **anything else (laughs)**

RES erm … well.... erm…. (sigh) I've err been into macrobiotics, sort of since, since the 90s. I sort of yeah erm. I don't I don't do it sort of religiously, but I've erm I'm aware. And erm what else? Erm If things are important, I have to write them down and make lists or, you know, put me in the diary if just gone Yeah, that's, that's one of the things that's got me through erm....erm...well I get…. well music, music gets me through this this arrived yesterday.... its a groovy one 1920's and 30s.

INT2 ***What about the flip side?***

RES Yeah. But it could be brass next sort of anything, you know.

INT2 ***So what about the flip side of the argument then? What about what's been most difficult during lockdown during the pandemic***

RES Erm...erm….. to be honest it… for me personally just, just the waiting and the dragging on of it you know, it's it hasn't changed my life that much. you know the it's all the volunteering stopped that you know, that's been a drag but er, had already stopped because I'd erm damaged my knee cartilage, while having fit. And so I had already stopped volunteering. And now when I was ready to go back, because it had healed it's all it's all stopped because of the erm because of the plague. And erm sort of erm because I'm not sort of really part of this community, up here, which I have been part of the community in the last village, like I was part of the team that when I lived next door to a little wooden hut, that was a library in the last village, and when closed the library, I was part of the team that turned it into a community centre, and ran the community centre. And erm so it would have affected me much more in the last village. And here it's not really affected me that that much. erm You know, I'm just .....you know, I've got to wear a mask when I go out. And, err okay, well, you know, that's not very pleasant it steams my glasses up and Okay. And I've got to be really organised about buying my food and storing it up. And what else erm.... but, but again, so living in a bender for 11 years will do that. But I'd sort of fade faded out of doing that live in the house for years. But we just got back into it again. erm What else.... that when I moved into this house, the neighbour downstairs was erm his wife was dying, and he was abusing and I'm have been trained to support families for charity. So, the first thing it was offering support, but he just was he is really aggressive to me. And then he carried on being aggressive and abusive to me after she died. And I've had to phone the police about him about 300 times in six years err but in this lockdown he's I've not had to phone, I've only had to phone the police out three times. So yeah, he's calmed down. So that's good. erm...

INT1 **When you spoke about**

RES although he keeps playing really loud music, and if that's what keeps him quiet? Well, you know, I've got a studio in here we can compete.

INT1 **And you spoke about sort of being in the community and setting up the community centre things in your last village. If that's something that you would like to do in this village, would you like to be more involved? Is that a plan?**

RES There's a community butcher shop here. And I went and offered to, to be one of the on the team. and they're really funny with me, they were really weird with me. And started, I just, you know, backed off and didn't didn't go back and left them and stopped going in there as well. As a result of that. I don't know if it was because the neighbour downstairs had said something or if if they would just being weird with me. erm I can't I'm not sure. Erm.. But yeah, just I didn't even go in there for me newspaper anymore. Em…But yeah, I would do if I offered so they had the opportunity, and they didn't want it.

INT1 **How are you feeling about sort of post lockdown and getting back to normality and being out and about and things?**

RES Oh, it'll be a relief (sigh). But you know, what, what is normality? Is that does that come in inverted commas?

INT1 **It certainly does. (laughs) What What is it you're most looking forward to doing?**

RES Getting back to some of volunteering, I think. I'd, Yeah. Going back to the mending footpaths and putting the dams back in to Hardcastle Crags. yeah... Not having to wear a mask at the supermarket anymore.

INT1 **Yeah. And are you concerned at all about contracting the virus or your health?**

RES We learned at junior school, you can't catch bugs if you do this no.

INT1 **If it worked back then, bet they haven't tried it this time (laughs). SD is there any other questions that you'd like to ask before we move on to the scale,**

SCALES

RES (1.13.37) Brilliant. Sounds good. Yeah. The first few years it was I was just sitting in the house, not knowing what hit me. Everybody had vanished. My mum was a support, but you know, she's in the she's in the 70s. And, and she lives miles away. I was a single parent. So my, my kids were getting probably the worst of it. Erm of course At school all day. And then when they came home, they were just often playing on the computer. So I mean, yeah, they did. Yeah, they just needed me to cook and do the washing Really? And err yeah, it was just Yeah, I was just sitting there not knowing what hit me. Yeah, the whole everything just gone out the window. Yeah. And it wasn't until 20 years later that I realised what he was that had happened and the doctor should have known the consultant and (unclear 1.14.38)

INT2 ***from your story, particularly I find it, it's maddening that there wasn't a level of erm medical intervention. You know, when this particularly happened, it's just it's crazy to think that all the signs were there and there wasn't a push towards you know, some type of At least consult with a consultant with an understanding of brain injury or something in relation to rehabilitate or focus rehabilitation for that particular issue. Erm Yeah.***

RES As well, now, when they go and talk to them about it, they might they I think they realise, and they're going, oh, oh, yeah. We're gonna get sued, and they clam up. And that's not what I'm after. Really, what I'm after now is, is I want my records to say the right thing. You know, and so, as a result of that, have have changed to the one in where's it called? Seacroft. Class, that means we've got to travel all the way to flipping Leeds ever time.

INT2 ***Not Great.***

INT1 **Well, I hope you get a better of service from them, and you start to get some, some answers from some moving forward a little bit fingers crossed.**

INT2 ***But no, thanks. Honestly. Thanks so much for having a chat with us. It's been it's been really good for me.***

RES *You're welcome*

INT2 ***And yeah, I mean, I'll keep you posted in terms of the results as well generally, and do that for all participants. So once we've, once we've got a fair amount of data, we'll do some analysis. And then hopefully, we'll get it published. But obviously, everything will remain anonymous from from your end, so that you'd have to***

	worry about being named. And but one thing you can do is just keep you abreast of the results. So see what happens and I can be back in contact once we've got something
RES	interesting
INT2	***Well, thanks so much for spending some time with us today, Sam. Thanks very much. Yeah, have a good rest of day. Bye bye.***

CONFIDENTIAL

Interviewer(s): ***INT1***

Respondent(s): RES (P5)

INT1 Okay, so you said obviously, you had your…Did you have… when you had the car crash was that? Did you have the stroke and the brain injury at the same time? Or was that, are they different events?

Res I had a car crash, which…there was a sort of cut on… the artery got blocked. So, I had the stroke sort of 12 hours later or whatever. And that led to, I don't know which order it happens, but that led to the brain injury as well.

INT1 And…erm… but do you remember anything about your rehabilitation afterwards?

Res vaguely, yeah, I mean, I know I was in hospital for three months in total. And I had a lot of Warfarin. And I just, it's just a slow plod basically. As I say there was a period of sort of a day before the crash and two and a half, three weeks later, which I don't remember at all. I wrote, I've written a book called polar strokes, I went to North Pole beforehand. The first bit is about going to the North Pole and how I got there. And then the second bit is about the stroke and how it works. I decided to write, I've got a section, which is basically the period of time that I had, I had without memory. And it's comes out round about a page of A4. And it's total rubbish. Which make sense because, so it makes sense, because I'm here, I'm walking along the road and things like that. But the bloke I saw he was in a 1950s coat, literally open top van so no glass at all. And he was had no hair probably sort of up here, down here. But he certainly didn't exist. But that's it. That's my entire memory.

INT1 So how does that make you feel thinking about that now looking back?

Res It's sort of a separate issue. It's my past, and that's what I've got. And that's how it is. And I think there's no, there's no point worrying about it. I just say, Well, okay, that's, that's what it is. And we'll let that be. Bu everything's… everything I do has been changed. On the whole, I suppose it's quite dramatic, but I just accept that's what Ive got now

INT1 this was, this was what I was going to ask you next was in relation to how much of your daily life do you feel has been affected from the stroke and brain injury?

Res Yeah, for a start knowing you were coming on today. I couldn’t do anything beforehand. So which is why I…I accept that you have to leave it later. That's fine. That's why I wanted it to be 10 o'clock. I also know that actually, I'll be very short afterwards. Because in fact, I'll probably end up sleeping depending on how tired I am, whereas that would not have happened before, you know its just another interview if slightly different from my work ones, but it's the same principle. So fine. I think that's the big thing is the amount of awakeness time I've got to think about you know what I mean whereas before I would do my bit and then get on to the next bit and so on.

I was a very very keen walker, but now I've got a stick and that sort of helps me along but there’s only so much I can do. Beforehand. I used to guarantee if I were walking with REDACTED my wife, after five or so minutes, she was just, can you please slow down. Whereas now anything it’s the other way around. And I think that's, that's the real thing is that everything is that little bit. Everything's just that down a few grades. And of course, because everything's down a few grades it has an impact. I don’t know if you know the covid stuff and you hear this sort of people may lose their taste people may lose smell. I smell, people may have problems breathing. All of these things. She used to say to me well can you taste as well as you can. Well I can taste as well I can since the stroke but not as beforehand. And all the things in the COVID things these could be wrong with you I already have.

INT1 Do you see a lot of similarities and between the effects of COVID to some extent, and the effects of your brain injury.

Res Yeah, I mean, it's I became aware of this, actually, with the North Pole, because I was taught all sorts of things about cold awareness. And as it happens, I've got … I have a certain degree of coldness with me. In my shoulder particularly for some reason, but things are as taught, there are three places where you are naturally warmest, your armpits in your crotch. And, and I remember being told this when I had the stroke and I though why do I know this. And it suddenly dawned on me that actually I had been taught what happens when you’re cold, rather than what happens with an injury and there’s cold but fundamentally they’re the same thing. But it did make me realise there's a lot of things as you say, that you tend to treat them as just something else. But actually, if you look at it properly, you think Yeah, okay, that sort of makes sense. Theres a slightly different reason why you’re taught it but fundamentally it’s the same reason

INT1 Youve spoken a bit as well about your support network to some extent. So REDACTED, your wife. Over the course of if we just think specifically, less so around the stroke and the brain injury now, but more so around sort of the pandemic? Can you tell me about the people who you've like interacted with over the past year? Is it just REDACTED or are they’re friends as well?

Res REDACTED is far and away the most important. Theres a woman called [REDACTED] from REDACTED. She's very good. But it's very much a plus. We sort of extended it beyond the professional amount, but it's still like professional. They're sort of, you know, she still is an employee of a trust, charity that does support me. And they've been very good to me, and her in particular, then our church has also been reasonably good. Now in Scotland, we've been a little bit more free to go to church than you have in England. And that's, for us, that's sort of one hour or so each week, where we can get you there and meet people and saying hello to people, and getting to know them. Were limited to, I think it's 48, just short of 50 because of the size of the church. But so fundamentally, they tend to be the same people or at least most of the same which is actually quite nice but that's it really.

INT1 So that's been sort of the extent of the social interaction you've had really over the past year?

Res Pretty much. I mean, there's been a little bit from chest heart and stroke, which sort of I used to go to meetings of theirs when we're allowed them. But they've been sort of very intermittent phone calls, but not really. Our doctor I've sort of I've not seen face to face, but I've had a few things to do with him. But again, that's all. Whereas before, we used to be a little bit more, more friendly with each other. That was just the COVID things very much. This is this is what I've got and how you deal with it, rather than anything else. But yeah, I'd say that's pretty much it. Yeah

INT1 Thinking of an average week for you. What are the types of activities that you would do in an average week sort of outside of the pandemic?

Res To be honest with you the pandemic hasn't made a huge amount of different to me, because I was always not stuck in but I was always I tended to be pretty much inside quite a lot, because you've only got so much alertness and awakeness. Now yesterday, for example, we walked down to the high street. And when we were last were there, we decided to do a couple of extra things. And it absolutely killed me. And coming back, REDACTED, fortunately she, she's a nurse. And she knows when I'm not right. Whereas before we were sort of just walking down the path and things like that she was very much here, guiding me and helping those sorts of things. So I wouldn't do that much extra, what has been, what has been more annoying, is that I used to have a reasonable amount of erm…you know I used to meet with chest heart and stroke once a week but it wasn't great it was just a meeting, and it was something else extra to deal with. I used to meet with Headway a couple of times a month and it meant that I got to see them and meet them. And they might have the odd thing away and such… conference away and stuff, which is fine, because it was headway. And so all the rooms, everything so from that view point it was good but in terms of what do I do in an average day, it's not really a huge amount of difference. What there is, is whereas before, where I went for a walk during the pandemic, I tended to go down to the own a bit, because he knew where to sit down and stuff but it very much became stay local, things like that, to

change it to be stay local. But fundamentally, I went for a walk and it was the same rather than looking and seeing what was in the shops I'd look and see what was outdoors which was actually probably better

INT1 And in relation to you obviously spoke about the headway activities and going potentially going away and things of that. Have they ever been any sort of online versions of the support that they've been offering that you've been attending?

Res I probably see more. Because it's now on the computer and I see…whereas before... I mean, I don't know if you know this area at all but Im in REDACTED and the meetings with Headway used to be [REDACTED], [REDACTED] or [REDACTED] and they would have three meetings a week. Every… the nearest one of those is about sort of 20 miles away. I don't drive. So I can, but that scares me now. Too much concentration. And, and to be honest, REDACTED could take me but it's not really fair on REDACTED to take out the entire morning or the afternoon or whatever. And so I tended not to do that many of those, whereas the computer one well its around REDACTED and that's fine. In many ways that's actually worked out better. And they've been, as I say, they have been very good to the point that actually I joined the Board of being a Trustee. I don't really know how much I'm going to do for that yet. Then they know, you know, they know the score, and they know. So they've made the decision and it's up to them to live with it.

INT1 That's pretty good. That sounds really promising. And so obviously, one of the one of the things I'm really interested in, in this project is sort of exploring the idea of loneliness as well. And what I like to ask people is how do you define the term loneliness? What does that mean to you

Res I think, is the…It's just the ability to have…or not being lonely is the ability to actually ring up and speak to someone or just pop round. I mean, it doesn't really bother me that I can no longer go to someone's door and say, How are you sort of thing? I never used to really do that anyway. But it's one of those things that once you know you can't do it it's a lot different from whether or not you actually did it or not. I actually think that my, my alertness level and my awakeness level. And of course, the fact I'm not really fully aware of what's happened to me even now means that I'm relatively... I personally am probably not too bad at being lonely. But one thing personally really affected is my wife, because she's sort of stuck with me. And she doesn't have that she doesn't have the benefit of not being able to remember it and not being able to be aware of it and I think from that viewpoint I am definitely concerned about it

INT1 Have you had that conversation with her? Have you spoken to her about? You know, about that type of loneliness? Or how that may affect what you just said to me there***.***

Res Yeah, I mean, to some extent, it comes up every so often. But it is it's a lot of the problems historically with the brain injury, actually are more of a problem for her than they are for me because brain injury is such that I sort of become, well, sort of blasé I suppose to the impact of them. Just so you know I had the brain injury and I've got exemption from council tax, and the doctor was meant to put on a sort of problems this is likely to cause and I cant remember exactly what it says but it does finish with 'and has childish dreams' which I guess means is childish full stop…and I think she's probably right with that. There are things that I do remember doing that when I was sort of 12, 13, 14 or so? I don't remember doing it when I was 35. Yeah. So it probably is right. It does mean that I sort of put more pressure on REDACTED than it does on me really

INT1 Just in relation to if you …the way that you define loneliness, this idea of, you know, kind of being, I guess, outwardly social, or sociable to some extent, and that not being not... I guess you said you weren't like that before, but it's having the opportunity taken away from you to be like that, is one of the issues.

Res It's a very difficult one. I think. Yeah, I've always, I've always been a fairly sort of independent person. And, yeah. I never had a huge army of friends. But I had very good friends and and, you know, when you're able to just sort of pick them up, and I might only see them once every month or so. But it was knowing that they were on the end of a phone, come across and see them. And it's taken a level of sort of alertness and awareness that it's just taken that level. Now, I cant see them.

INT1 Do you think that if you did feel lonely, or you did have some feelings of loneliness that you would be able to speak to someone about them?

Res Yeah, I mean, I mean, I know that I could speak to [REDACTED] at Headway. And she would be pretty. She would. She's one of these people who was more … became more of a personal phone call than a Headway phoencall. With chest heart and stroke I know one of the people there I could speak to although I'm not entirely sure how good he'd be but I can speak to him, And again, I could speak to the church. So I mean, there's a variety of people I could speak to. There isn't, outside of REDACTED, someone who really fundamentally knows me and knows what's happened to me….they know what's happened to me but they don't really know the impact of it all

INT1 If we think about … I know you said the lockdown didn't really have, you know, as a massive impact on you because things I guess the real change was the brain injuries. The stroke and the brain injury. If we were thinking about lockdown, what would you say has been the most helpful thing that you've experienced over the past year? What's helped you through this? It can be the answer can be nothing as well, if nothing is really changed.

Res Well I mean, there's no doubt REDACTED has helped hugely. What REDACTED has done is made me aware of the fact that… it probably took me a bit longer to realise that we were in lockdown and couldn't do things than it should have done. She really got it through to me that you can do than and cant do that and that viewpoint was great. And the Headway people, what they've done is given a sort of, yeah…they've acted as an additional group of friends, which is nice. And one of the things that's happened, when I went to the North pole, since then Ive sort of talked to a few people about it as a general, personal adventure audience and what Headway have done, is they've actually said, I spoke to them first the local Headway, and then a bit later on, she said, why don't we just, do you mind if we put out to all the people at Headway? And so I've done about five now, people, various people, because they're all headway people. They all understand what I'm going through as well. So they're sensible about the behaviour towards it. And so from that viewpoint, that's been quite nice, because, the North Pole, what it does is gives me something to dwell on if you like, that sort of, I've got the old and the new REDACTED really. And it's something which is like I've done it, doesn't matter that I can't do it now. I've done it. That's been quite nice. It's difficult to identify things that made a difference either way.

INT1 That's completely fair as well. And is there anything that's been really difficult over the past year?

Res Not really. I mean, there are things that…it used to be that I used to be able to go for a walk and it didn't matter how long...I knew that if I went for a walk, I'd probably have to sit down somewhere… whereas before and that's stopped now to be fair everything's come back to some extent but I never really got back into it. I don't really know of anything. I suppose what's happened is we've set our sights a lot more local and we stick within this certain bit now whereas before I didn't think about it whereas now the moment I'm going more than a quarter of a mile from home lets think about this and what I have to do

INT1 So there's more of this sort of planning element around your activities.

Res Yeah, I mean, planning is a big issue for me as it is and to the point that I tended to just let REDACTED do it whereas what's happened now is that I wont…whereas since the lockdown its a little bit more serious, and, you don't really want to have people coming up and saying hello. You want to keep away from them. So yeah. Yeah, it's probably taking that extra level of thought that's needed which means that I don't bother and I just stick locally now.

INT1 And, and obviously, well, fingers crossed, we'll see what happens. But we're moving more towards this post-apocalyptic lockdown-less society, obviously with things opening up again over the next couple of months now. How do you feel about that? How do you feel about this idea of society, you know opening up again, potentially getting back out there.

Res I think it's a good idea that it's opening up, but I don't think I'm going to take an active role in it. I mean its…we've now got the situation where pretty much all of the shops are open in Berwick. Not entirely but pretty much and whilst I sort of have a look at what's in the window when I go past, I wouldn’t dream of going in unless I specifically want something. And I think that's going to be the way I’ll be for a long time. If I want something I'm going to go in and get it because we have to, otherwise no.

Interviewer(s) **INT1**

INT2

Respondent: RES (P6)

INT1 Can you tell us a bit more just sort of around your brain injury, how it happened?

RES Yeah. I was due to go walking erm the day happened and had my nephew in the car with me. And we head to REDACTED. And we're due to walk up, erm to REDACTED, and literally Joe was ill. So we decided to go back and I had a black out or whatever. And we jumped it. So went into a field, erm jumped a, dry stone, well jumped over, after then who went into (unclear - four rolls?) And I stopped, erm next to the river swell. So Joe got out, manage to get out the car with some cuts and some like, and luckily, we had this guy's stay staying at the hotel, the bungalow just next to us. And we erm well they managed to get me out, undo my seatbelt. And then I had two helicopters arrive. So the REDACTED also arrived. And they err using the REDACTED and they transferred me to REDACTED and then I was in a coma for a while 8 weeks then hours in hospital for eleven months. So

INT1 and so when you extremely harrowing experiences, so when you came around?

RES Yeah.

INT1 Do you remember anything of what had happened? Or is this just purely recollected from other people?

RES Yeah, it's from other people. Erm on the day, my nephew, erm that people helped me and then nurses and doctors in the hospital and my family erm didn't have, I started to remember a little bit in in hospital and probably in April time. So about four months went and I couldn't remember Yeah,

INT1 and what what are the sort of main issues that you've experienced since your brain injury?

RES Erm.. Well, On my right hand side it's weakness. So my right hand side I used to kick football I used to write my my letters and notes on my right hand side, erm i used to not have glasses on but due to the due to the head injury, erm my right hand side was completely fucked, sorry.

INT1 No, no no, speak as freely as you want

RES Yeah, it was it was completely gone to the, I left the hospital using a wheelchair. Couldn't really walk. So yeah, um, I use the wheelchair and came home. And for about a month or so, I was staying downstairs, then we got a stairlift put in. And we have that for about year and a half and we only we took out last year and did charity walk erm and took 81 days to walk the height of Scafell Pike erm using my steps, stairs. And yeah. took about 81 days, raised a lot of money for great north air ambulance. And erm we yeah, did that. Also, erm problem well, not a problem erm (mumble) A thing I've got is I've got aphasia. I don't know if you know, aphasia. What that is…

INT1 Yes, yes speech and language production.

RES So got an issue with that as well. So, yeah.

INT1 Do you find that these issues have affected obviously massive portions of your your life? Like considering your life previously? Or?

RES Erm yeah. So I used to play football and things like that when I was younger, and I sat just the other day and watched my wife kick some footballs to my son and daughter I was thinking to do that I'll probably won't be able to again. Erm Things like that erm I know, well, my wife has said to me about if we go out to have some drinks and things with friends, I'm a lot more quieter than I used to be. erm Yeah, really.

INT1 Do you find you can still enjoy those situations?

RES Yeah, I can Yeah. I can do. It's just, like just, it's, I tend to sit there and listen to people more, you know, don't get involved with their speaking things. I'll still say my, my words, but I'll take it. I'll listen more.

INT1 Yeah, If we circle back to um your brain injury, specifically thinking around the rehabilitation that you went through? Em can you just talk to me a little bit about that? So, did you have a lot of rehabilitation? Like, obviously, with with the aphasia as well, I guess there was some speech and language elements to that.

RES Yeah, I was, I was seen for the eleven months I was in the hospital. And probably for about eights month I was seeing a Physio daily, trying to move and things like that. And then also also seeing erm speech and language on weekly basis. And, erm and yeah, I was seeing

them. And to be honest, I think it's helped me a lot. I'm also now seeing erm so I'm in touch with headway. Don't know if you know headway. But yeah, headway. So I'm in touch with Teeside and Hartlepool as well. Darlington I was seeing them for I went to see him once or twice in, when I first came out of hospital, but to be honest it wasn't for me at the time, because I have to focus on my rehab. Erm Yeah, and I'm also using another group called Say Aphasia, and I-Cafe as well. So it's good and erm I've started to my previous work, I used to work for REDACTED and I was going down to Bourneville and leading some erm meetings and things. So obviously that's gone. (unclear 07.58) well, with I-cafe and headway I start to do some quizzes and things just to get involved with it? little bit more? So yeah, come on a little bit more.

INT1 Do you find those groups helpful?

RES Yes. Yeah I do. I have been doing since about October time last year. And yeah, they're they're good. So with the weekly call, don't think I've missed one at all. Yeah, I found really helpful.

INT1 What kind of things do you like about them?

RES Erm the group itself is only about four or five people. And just gives us a chance to talk to other people who have had the same sort of issues specially with speech and language. To be honest most of the people in in the call are erm stroke people that have had strokes. And erm I've just said just sent well I'm bout sent a letter to erm the doctors, nurses that have helped me and headway and I-cafe and so on to say, is there anything up here that's available? I've heard this a group called Netta, I think it's called and they're based in Newcastle. And to be honest, Newcastle's a little bit too far for me to go at the moment. So is there a group in Teeside Or? Did obviously headway is good, but it's something else there who's available? We've got erm, (10.03 catch up) area of Darlington, Richmond, Catterick oh see at Catterick there the the Army's base there?

INT1 Yeah,

RES There’s Durham, there’s even Sunderland, and then probably south of the Tyne. So, but doesn't seem to be anything available, for people that’s got aphasia and things.

INT1 Yeah, I can't think of any off the top of my head either actually.

RES It's just so there's something there that hopefully if there's something available for other people, and to be honest the groups of like Headway Darlington, when I speak to one spoke

to them and when I went to group were talk about arts and Scrabble and flowers and to be honest And for some people, that's fine you know, I won't say anything bad to be honest for the younger type people as happened to me. Is there anything out there probably not at the moment? So, I thinking about setting up my own group

INT1 **Sounds like a good shout Yeah, yeah.Erm in terms of obviously, you spoken quite positively about the rehabilitation that is available aside from obviously the lack of potential groups or things in your particular area or particular catchment area, for aphasia. Is there anything that has been particularly hard during rehab or anything that was particularly difficult or? erm.. Yeah, I guess that you found particularly hard in relation to rehabilitation?**

RES Erm To be honest no, I didn't, because I've done my video on YouTube, with what happened that day, and I'm thinking about that and it's called Pete Cody story, and it's on YouTube now. But the biggest thing about the video is PMA, I don't know if you know it but its positive mental attitude and that's you know. Every day I think about that and now you need to stay positive. You know, there's no, you know shits happened. And you just get on and do it there's no point thinking about what if this happens or this happened, and you know, it's happened, it's happened, nothing else you can do about moving, just move forward. There's probably people out there that's in a worse place than us really.

INT1 **Yeah. That's good, good mentality to take, I think. In relation to, so you spoken about your support network, and specifically your family, but also, obviously, you know, headway and the doctors that you've been in touch with, you've mentioned them as well, in terms of being quite supportive. Over the past year, so if you think about it in terms of 14 months or so, think about in terms of the pandemic, who were the people that have helped you most or who the people that you've seen over the course of this past sort of 12 to 14 months.**

RES We has a convo, just had a conversation with my wet wife about it actually and one of the biggest things about the pandemic is home-schooling actually, and dealing with home-schooling, because I've got two young children, who are just at school age So Abi's now nine, ten sorry and Harry's six and doing home-schooling it's not just brought something out for them, it's helped me as well. So to be honest, the home-schooling things I used to focus on setting up the work things and erm maths my son and English with both them and things like that

INT1 **that's really good, really positive. I know a lot of people have really struggled with the home-schooling aspects of it. So it's good that you've, you've got something out of it. Is there anyone else that you've seen over the course of that this period of time, so any friends or anything**

RES Erm I have stayed in touch with people To be honest when we we first had locked down number one, and we used to do a quiz on Zoom quite a lot erm it was weekly we did that with friends, and then family. And I've stepped up. I've been in touch for my family a lot since it all happened. And you know we've we've kept in touch to be honest through zoom and other things I wouldn't say so I had this situation that I'm by myself at all. Yeah. My family at home, my family, my big it's like because I'm after a big family. So we've got I've got three sisters and a brother. Who's got erm married they've got children. We've got 11, well, my mum, dad, have got 11 grandchildren. So there's a lot of people I think there's the, I think there's 29 of us, so been touched quite a lot. Since since all happened.

INT1 Do you feel it's brought you closer.

RES Yeah, probably. Yeah. And to be honest I think cuz I'm at home quite well, I've been home a lot. People have been focusing on me, it's like my sister is hitting 50 tomorrow actually. So then we we set it and we've aim to do a spa day, and a hamper and to be honest I've been involved with that, well I've ever led it. So I've been involved with it and erm would do some bits for my fortieth and again, I've, i've done it myself,

INT1 So it feels like it's given you an actual, more of an opportunity to be involved in things that maybe you would have been overlooked or not been able to be involved in?

RES Yeah, yeah.

INT1 Cool. Erm, So think about it. If you think about now sorry, in terms of the activities you've been able to do, erm..how have the activities. So obviously, you know, you spoken about some of the activities that erm for example, playing football would be something that you enjoy doing pre brain injury. Erm has your level of daily activity been affected by the pandemic itself?

RES Yeah, I'd say so, erm like you say I was big into football, erm mountain biking things like that. And so I've just sold my bike actually, we, we sold it for about an couple of thousand. It was, it was a decent bike. And I used to take it don't know if you know Hamsterley Forest near Durham. So yeah, when I used to use that. But to be honest, we've just bought a new trike a mountain bike trike. So hopefully I'll be able to get used to using it. It's a bit it's a big change to using a bike because the width of the bike, bike wheels, and it's a lot bigger. So I've already come off it once. And err hit my arm. But yeah, we'll be good to get up there and do a little bit more, if if I can. Yeah.

INT1 As the as the pandemic affected, anything you've been able to do, as well. So over the past sort of 12 months.

RES Yeah, because I'll probably go to Hamsterley with my friends. But obviously we can't do that at the moment well we couldn't. And erm yeah, I'll be going with my, my young, sorry my older nephews as well. But again, that's not been available to do so as it's come backs to erm a little bit more. Yeah.

INT1 Just before I move on to the next section. REDACTED, do you have any questions?

INT2 just wondered about the lockdown. What is it? Or is there anything that you found particularly difficult? It seems like you've had quite a positive experience sort of, especially with home-schooling well done on that one. And, and you've had more socialising because you've been able to be a bit more organised and more involved. Is there anything you found particularly hard?

RES Erm to be honest no. Again, all, just because of the accident I have It's just gotta stay positive. So here I've been at home but, I've been at home with my kids and things I wouldn't probably have been able to do with working because I'm still in touch with some of the guys who I used to work with. They've been at work 24/7. Well, since all happen, they've been working so probably wouldn't have been able to do that. And yeah, I things that at home, I am at home a lot , we've developed the house, and we've done a lot

INT1 Sounds like you've been grafting? (laugh)

RES Aye (laugh)

INT1 So this is a bit of an abstract question, I guess. But in relation to loneliness, if I was to say the word loneliness to you, how would you define it? How do you define the term loneliness?

RES Erm.... erm.... I don't know actually ...... it's…. I don't know

INT1 What does that word mean to you?

RES Yeah. Well, obviously people go through loneliness and. erm some people will be getting erm be having a lot of loneliness with the pandemic. Erm. Have I seen it? No, I've not seen it myself. Because of all my family and friends around erm what people probably have, have seen it, especially not being able to get out and about and doing things.

INT1 **Is it something that you would have say, so, I'm going to ask this, and then I will, I will say what I mean in relation to it. But have your feelings of loneliness changed over the past year or, you know, prior to the pandemic, and then post pandemic. And what I mean by that is, in terms of it doesn't have to be increasing. As you've said, most people probably have, it could be the other way around. Whereby obviously, you feel less lonely, because you've now like, from what you've said, before, you've had these, you know, you've actually been quite closer to your family, and you've been able to see your kids more spending more time with them because they're at home, etc. So that's kind of what I mean, I have, have those feelings of loneliness or, or have you even felt lonely prior to the pandemic? I know, it's hard to look back and think about it retrospectively. But have those feelings changed over the course of the past 12 to 14 months?**

RES Erm No, I don't think so actually, because we've always been a quite tight err group or people, family and friends. So, we've, we've stayed in touch and during the pandemic and beforehand as well, always been in touch, again I told you might just be because they the whole positive mental attitude I've always been a positive guy. And I've always if there's anything wrong or, people turn turn to me. Yeah.

INT1 **And if it's if it was the other way around, so if it was something that you felt, do you feel you would be comfortable talking to them about it talking to friends talking to family?**

RES Yeah, yeah.

INT1 **Yeah. Excellent. And the final thing I want to ask about and then I'll I'll see if REDACTED fancies talking about anything as well. So obviously we're in a position now where the apocalypse is slowly ending and we are allowed back outside to some extent. How do you feel about life post lockdown, you know with society reopening.**

RES Again, we just just need to get erm to build my strength up again. It's it's not just been the pandemic but like this morning We went for a walk with my wife. And we only went down road and probably about, I used to be able to in 5-10 minutes. it took me about half an hour to.. you know things like that all will take time. And erm having the brain injury myself, I think I've been to a pub what two or three time since I came out, what three, three years ago so what what happens. I just again all you know what's what's happened's happened you can't change it you just got to move forward I think

INT1 **It feels like your mentality you're really strong positive mentality is something that pushes you forward regardless of what's going on around you if that makes sense.**

RES Yeah, well that's it because we can't change what's happened you know, there's no point thinking about it too much. What's happened happened, just get on and do something else and different.

INT1 That's really, really insightful really, really good to hear. REDACTED, do you have any additional questions?

INT2 Yeah, just one I just wanted to check. Do you feel like the pandemic set you back in terms of building up your, your physical strength? You feel like it's been on hold a little bit? Or has it not really made much difference?

RES Erm. No, well, to be honest I was due to finish the physio well I finished physio, the weekly physio erm, the Monday and obviously went down to lockdown on Friday. So that's all ended. I was using a hydro pool and from South Tees hospital to James Cook hospital every other week, and obviously that's now ended due to lockdown things. So, and that help me with my strength and things. But you know hopefully that's going to start again in the next couple weeks.

INT2 Thank you.

INT1 That's great.

Interviewer(s): ***INT1***
INT2

Respondent(s): RES (P7)

INT1 And then if can start and just tell me a little bit about your your brain injury please um

Res it's not so much what I remember but it's what my wife told me. It started around Christmas. What year are we now? 21? 20? yeah Christmas 19 going into 2020…she noticed I was…because of the jobs I've done I've predominantly worked outside. So if it's called everyone else's walking around with big thick padded jackets on and I'm in T shirt and shorts I just don't feel the cold. I was coming in and I was like, oh, I'm really cold. Can we put the heating on? Which she thought was very strange for me. It was not that I was a little bit chilly. I was shivering. And then it branched into memory loss. What did I just have for me dinner. I've never had a very good memory the best times for the not innocuous things but it was getting worse. And then on May the 14th I'd been outside laying a patio, so thats how well I'd been obviously other than the cold, I'd come in, she'd made a nice cup of coffee and as I went backwards for coffee my mouth started foaming. I'm not the smallest of people shall we say, my wife is 9 stone ringing wet and she managed to support my weight as I had a massive epileptic fit in the kitchen. We've got a galley kitchen so prior to other than the cold and the memory loss no major signs. I was taken to North Tees. Kept in over night and sent home with paracetamol suffering from stress which anyone who knows me I've inherited my response to stress from my Dad. It comes down to two questions; can I do anything. If the answer's no, then no, don't worry about it. I've always been the same, very stress free.

My condition carried on through the next week. And she's on the phone crying and sobbing, can someone please help me and it came to the attention of Neurology at James Cook. Dr. [REDACTED]. She's the consultant Im under. It came to her attention. And she said, Well, can you bring him in on Friday? She brought us in on Friday morning. And she said well, can you bring him back at tea time and we'll admit him. Obviously unknowing what was going on but she knew it was something neurological.

I agreed with it on the morning and then she basically had to cattle prod me to get in the car on the tea time to go in at which point once I got in there, I was obviously not with it at all. But she was sobbing at home because I was sending her nasty texts saying you've just abandoned me, you've kicked me out but deep down she knew it wasn't me. She knew there was something wrong. I was kept in for five weeks. By the end of the first week, they kind of sussed out that it was encephalitis. What it was, I don't have epilepsy. But the encephalitis had triggered an epileptic fit/event whichever you want to call it.

Post that while I was in there, I had five courses of, what do they call it, blood, cleansing where they change the white cells in your blood, pump it all out and put new ones back in.

Doctor [REDACTED] had phoned REDACTED and said I wasn't happy because they wouldn't tell me how much all this was costing for me. Each treatment was 2800 pounds when I found out I went off it. Theres nowt wrong with us. Obviously in your head, you've just got a bad case of flu.

It has been pointed out by various members of the family that I'm very similar to how it was beforehand, but I'm still not the same person. But from my perspective, I'm no different to how I was. it's not off putting but it's strange that they say that and for me I don't see any difference whatsoever. Apart from one legs five centimetres thicker than the other at the minute which I'm still receiving.. Im going for a scam tomorrow to see exactly what it is.

INT1 And has it affected much of your your daily life? So can you tell me a little bit about what's been affected

Res The main one is feeling useless. I mean I've worked all my life. At most from leaving school, I had six weeks off in 1993, other than that you're lucky if Ive had a week off in between jobs. I have always worked, Ive always been…not so much a physical job but using my hands and up until the latter which is the health and safety officer, but youre still out on the park. They've eased off now but I used to have terrible balance issues. To even put me …a pair of slip-on shoes I had to grip the wall. I couldn't put it on unless I sat down otherwise I'd fall down. My confidence has been knocked even though I try my best to…I'm always a glass half full sort of person. You always look at the positives and when the positive at the moment is I've got time to sit down as I'm repeatedly told sit down and shut up to do courses that when I get back because I refuse to admit that I can't go back top work, when I get back to work I'll have a broader perspective to look at different types of work if I cant get back into construction.

My wifes noticed and when she highlighted to me I have started looking out for it. it's very similar to the (unclear) some mornings it's really bad if you think of, what they call it Blazing Saddles, the thing with Gene Wilder with the gun, you know, look at that hand, but I can shoot with that hand. it's never both hands. But if I come in for a cuddle she can feel my body shaking where I cant.

My temperaments changed, I seem to be on a shorter fuse… things..It's fits and starts. it's not…you cant say well, the same thing that annoyed me today will annoy me tomorrow. But with the two boys if they start fighting all of a sudden I'll snap and my wifes got to interject before… it never gets physical. Don't get me wrong it's all verbal. I mean case in point the other night the little'un made his mouth go in the bath and she had to interject. But then its well what are you doing, hes mouthing off and then I have a go at her and then she will leave it while I calm down and mention it the next day nice and calmly but it's all the things you don't notice until someone points out, you think aye im doing smashing. And I'm ready to go back to work and then something happens you're like well, actually, No, I'm not. Even though I want to get back to work because that's all I've known. It just feels wrong to be physically

most of the time I'm fine. And it just feels wrong to be sitting on my backside looking into a computer doing nothing

Don't get me wrong, I help out around the house as I can and where REDACTED will let me but the likes of doing the Hoovering and or the washing up or the drying of the dishes or depending on how I am on that day she lets me, without bother me she lets me do the dinner or..and im quite happy but it's days where I've really got the shakes or I'm feeling a bit groggy or…I feel bad that I cant help out where previously I was working long hours or working away. I wasn't in the position to help or do anything because obviously you're not there, where now I'm there 24/7 but not available available to do anything.

INT1 Did you have or did you need any rehabilitation at all when you left the hospital?

Res Not really not that I remember. I mean I'm down to…well I was. I was down to 28 tablets a day. So I rattle well, I shake well, I'm just waiting for the rolls to come out now. A lot of it was conversations with the wife and the staff at the hospital are fantastic. They're there without being needy at your beck and call. You know we've got an email address a direct line. She says even if I'm busy, if the email flags up ill phone you right back. Not to the consultant because we know she's run off her feet, but it's her secretary and she can get hold of her immediately if need be. Whereas for rehabilitation, as you would think of it in that sort of way, yeah, I don't think it was required.

INT1 And then if we can talk a little bit sort of about the pandemic in the last 12 to 14 months, can you tell me a bit about the kind of people that you've been in regular contact with if you had supporting you? Any visitors, things like that, and a social side of things

Res Supporting us, without a doubt, with my wife, I'd have been knackered. there's no ifs. Buts, maybes. We've had my mother-in-law, I call her Mum, my parents have passed away so she's the only mom I've got. She moved in with us, because she's 80 on the 10th. So rather than kind of wonder, well, I can't come over because I'm breach of COVID, etc., she moved in with us for the pandemic, which was great at the time. Thinking about it backwards while I was in hospital REDACTED had someone with her and then she's part of our bubble. The brother-in-law will come in the garden and talk through the windows sort of thing. But friends…it surprised me. Considering that how many people knew what I was going through, few and far between of the big group of friends or thought I had had even sent a text. So I was like, Alright, well, we know where we stand. So next time I'm out, "oh how are you doing are you getting us a pint"…No. And that's pretty much as polite as I can put it.

But then there's some who thought were merely acquaintances even though you got on well with them. They've been round and, 'here's my phone number, anytime you need us you

need anything or you need any shopping or anything whatsoever, pick the phone up. It's twisted a few things on its head and cleared a few things up, shall we say

But with being in the construction industry to put it bluntly, you acquire certain products, just in case. So the nice selection of FFP2 masks. So while everybody else has got these cloth masks on, if I was going to just walk on the shop and get a carton of milk, the mask went on as soon as you come out the door. I mean, the saying, you've got to shield but sitting in the house is not good for anyone's mental health. So my wife… the shop was literally the end of our street. It's 600 yards tops. So she was quite content to let me nip to the shop for the milk or something like that. But like I say that FFP2 went on the moment I went out the door. But as for social circles that’s about it.

INT1 Did it surprise you that the change in your friendships?

Res Yes. One of them. I always thought we like brothers. We've known each other since secondary school we've been on holiday together. There was three of us knocked about together. And one before all this sort of went his own way. Whatever. But me and the other lad we were like thick as thieves, we were brothers. And apparently when I went into hospital, the first time he brought some clothes through for us. But that was July last year and Ive had no texts, no phone calls. Not even the message on Facebook or anything or a text to REDACTED asking how he’s doing. My cousin in Scotland, he’s five or six years younger than me. I'm the youngest son of the youngest son so he’s my second cousin, but we're more or less the same age.

He apologized and said I don't know what to say. I'm sorry, I can't talk to him, but his wife was in regular touch. And then once I come out, and I was more or less myself and when I rang him, he was almost in tears on the phone when I' was talking to him and he said 'm really sorry. I just didn't know what to say. Hello, it's still me. I'm still daft as a brush. And then both laughed and it was He's fine. We get regular texts now, like have you seen this video? It's just like before.

INT1 Would you say your level of sociaREDACTEDtion has changed because of the pandemic? Or do you think because there was so much sort of overlap with your injury anyway it wouldn’t have made a difference.

Res I don't think it's as much of a pandemic because I think we would…we would have still text or had a sneaky one as people have done, you know what I mean? But I think with what I had and I wasn't I mean, a lot of times I wasn’t compos mentis, REDACTED threatened to take my phone off me because I was just putting all sorts of rubbish on Facebook. I have no idea what I had written or what have you. But even so it's … it doesn't bother me I’m here, whatever. Get on with it. But at the time Okay, well, you know, Where is everyone? Where are all these

friends that I used to… any chance you can give us a lift here, yeah give us a lift with this, well you would do they're all your friends and now, nothing

INT1 And what about in terms of sort of keeping yourself busy? This past sort of few months, as you mentioned, going to…a walk to the shop, have you been doing anything else? Or is it mainly been the housework and something within the house is there anything else that you've been doing to keep yourself busy?

Res Where previously, I would have thought nothing more about getting the ladders out. You know, there's this and there's that. Again, it comes down to perception. In my mind there's nothing wrong with this. Apart from ooh I get the shivers. Youre forgetting that there's still issues with your brain and anything can happen. And at any point as look at the fainting the other week that came out of the blue. I'll do as much as REDACTED will let me or she thinks is safe. Originally, when we started doing bits and bobs in the garden he didn't want me using power tools. And I went why? But previously, I wouldn't have argued where now but why there's nothing wrong with us.

It's… it's very strange to look back and think well that's not me. And a lot of times we sit in the kitchen and over a cup of coffee or something and I say I don't mean to cause an argument or anything but what you said yesterday, what did you say yesterday? I don't recall. And it's frustrating from discussions we've had that I can't remember it. But she can vividly obviously because and you think… Well, did I really say that. That doesn't sound like me at all.

But I've done a few talking to the lady from the works and pensions this morning. Just to keep my mind occupied, I've done two online diplomas. Seven certificates. As well as bit and bobs required around the house. I mean, nothing major, nothing structural, putting like a new bannister up or something like that.

But that's about it. I did a woodturning course come up, which is something I've never done. And again, it's aimed at mental health. It's woodturning, [REDACTED] run by a gentleman called [REDACTED]. And on the first day he made like a little trinket box where the lid comes off, but it looks like a barrel, most things are circular. And as we varnishing I said can we engrave a letter on there, like, so put a nice K on it. And so the first thing I made was for REDACTED, a little trinket box where you lift the lid off. But then we made various other things, one of which was a massive clapper. We got a certain amount of luggage, mom's birthday coming up, her 8oth birthday. Can we get this engraved? He says if you want to do the engraving, {unclear} clips where you're hanging on the wall. So it's something I've made, which means more than going out and buying whatever. For it's personal that we've made it and she can use it as a sweety thing or put fruit on it. It's functional as well. I mean, I'll be honest. I cant cut a straight line to save my life. It doesn't matter how big you make the line, never have been able to. But to see some of the products I've made. I cant believe I've made that.

We did an interview for Radio Tees. And he said would you mind coming in? Yeah, no problem at all. You know if I can help promote, again, you know what you've done for me. It boosted my confidence. And there was a lad from the Collieries and he was deaf.

They did everything with another lad and they had an interpreter each, sign language (unclear) but like the wife says you know when we will have a little trinkets things and she can do the pyrography on it. Just whether you make money or its just something you can do to occupy your mind. It's something brilliant

INT1 Yeah definitely that's everybody's Christmas presents sorted for a while isn't it

Res Shhh they may be listening

INT1 Can you tell me a little bit more about sort of your social life before your injury? Were you quite social? Were you out quite a lot?

Res Beforehand, obviously, I was the health and safety manager at the Biomass down at Teesside, the new building down their on their steelworks.

A lot of hours, but…'do you fancy going out for a pint?'…aye! So I'm just going out for a few beers. It was one of those, whether it's as the kids grow older, or the position that I was in, always driving or working one day out of the weekend. It was never to the amount of drink you used to have when you were younger. But I'd still go out for a couple of drinks. Since the… lockdown and this none whatsoever. We've never been anywhere. It's not worth it. I mean, I can't drink anyway, the amount of tablets I'm on. But then REDACTED can't drink because given the fact that it is classed as an epileptic fit, I've got to wait for a month to get the licence back anyway. But then if there's always because there's only her that can drive if something happens to the kids, we're knackered, we can't drive. If something happens to her mum, again, we're in the same situation. No one can get over or go to the doctor's or anything. So we've just had the nieces round the house, and they've come home for the weekend. Ive just put my mask on and then at least they can interact with everybody else. But that's about the extent. We had a couple of visitors. I had a mate who used to be a DJ in a nightclub, we've known him… God. Well, (inaudible), put it that way. He called in the other week and brought us a pasty in, we just stood in the kitchen and had a bit of a catch up. But that's about it really.

INT1 How do you feel about your activities that you're doing at the moment?

Res I'm thoroughly enjoying the courses…erm…some of them, it's a case of just refreshing what I already know. But my wife sits and laughs because you're lying in bed and she's like 'what you just learnt?' and I'm like why and the fact you're going 'ohhhhh'. But I mean I've got a broad interest field of science fiction and ancient history. And she bought us the REDACTED Fry trilogy. Heroes, mythos…And I can't remember the third one, sorry. But reading that, obviously ancient Greek myths, there's bits that link up with Christianity and she's like 'I wish you'd put that book down'.

But I've always had a better grasp of the English language, shall we say for err…what's the word for English language? You know, when you translate the text into questions. The word escapes me but REDACTED is doing a care course, you know aimed at working in a care home. Not that she wants to do that. But she's got an interest in the ethos behind it. So I'm doing a lot of comprehension, that's the one. So I'm doing a lot of the comprehension to translate it from, because you can't copy the answers verbatim, so I'm copying that and putting it in like our sort of language. Obviously being from Hartlepool, I'm making sure to put the H's in, not 'artlepool. But it's that sort of thing, but I'm enjoying it. I mean one of the courses I'm doing this latest one, the train the trainer, it's all videos. There's no PowerPoints that you've got to go through. So I said well, I managed to get all of the videos. And I said, Well, I think I'll go through yours first. We'll get yours done. And then we'll submit yours. And then I'll go back to my videos because there's no time limit on mine. Whereas hers is time bound.

INT1 And have you enjoyed helping her as well, do you think, as much as doing it yourself?

Res Exactly. When you look back, and you say, See all the little things she does, even down to organising your tablets, I tried it once, and…don't take this out of context she screamed at, as you know, like, for Gods sake! Just leave them alone. But I've put them in the right way. No, you'll kill yourself if you took all them the wrong way, the way you've put them in. Right. Okay, point taken. Well, I mean, you look at the likes of (unclear) the last week. Yeah, the 10th. Obviously, she's got a bouquet of flowers and all the presents and everything. And then there's a knock at the door. And I'm sitting there with a big cheesy grin on my face. And you know the papers shaking. And it was Inter Flora, nice wicker basket with the moss in. And it's a plant, but it looks like flowers. And it's a big thank you for all you do for us. Love your boys. It wasn't just from me, it was from the other two as well. But it's just nice to show the better I get. Or the more cognitive I get, the more you can see all the little things she's been doing for us. And you know, I didn't realise that. But then you read comments on LinkedIn, and Facebook, you know, yeah, but it's, most women do all that. But we think we're at work. And we go, I don't want that. Yeah. So once a week, you run the hoover over and you think that's it. No, there's preparing bits and doing the shopping and making sure there's enough of this. And there's enough of that. And you know, I didn't realise that

INT1 Can just repeat all of that to my husband for me please!

Res Yes!

INT1 It could be your next project, please to train up all the husbands, if you don't mind

Res I said i was a good trainer. I didn't say I could work miracles!

(Laughter)

INT1 So just to change stance a little bit. How would you define the term loneliness?

Res Been there done that…loneliness is it's not so much the no one with you. But it's the thought that there's no one way no one looking for you or inside your head, you are by yourself. And when you got a head like mine, it's not a good place. And that’s at the best of times. When your theme tune is thundercats, you can sort of gather where I'm going with that. Loneliness is it's not a physical thing. It's a mental thing. You can be alone by yourself, been there, done that got the T shirts, sat at hotel rooms, but I know if I pick up the phone until, I can talk to my wife, I could’ve talked to my friends, I can talk to colleagues. Loneliness is where in your head you have got nobody and it, it could turn into desperation.

INT1 Do you think you've felt like that recently at all?

Res When I was in hospital, I think I went through stages of it. From what REDACTED said, because if I said I was ringing at nine o'clock and she didn't answer the phone at nine o'clock Id then phone the house phone and then the mobile. But then in the other way, if I was saying I going to phone at 9 o clock Id go sit and watch a film or something and phone at half 11. Well, the kids are still sat up waiting. But obviously, in my head at the time there there's nothing wrong with that. It's…it comes down to that perception. But again, the loneliness is definitely a mental thing. It's not a physical thing. I've worked abroad and I’ve worked all over where people will speak foreign languages and you've been alone in your hotel room but during the day you've got someone to talk to. I used to call my Dad up, I'm just having a couple of pints with my mate, what are you having, and we'd catch up. Maybe not every night, you know every other night or two or three days, but there was always someone to talk to

INT1 You spoke about sort of your changing social friends and quite a few have dropped off. How does that make you feel?

Res At the time I was…angry is maybe a bit too strong. Now it's just like well, let's Christmas cards to buy. I've always been a positive person which is, the consultant couldn't get, she thought I was… at first, there was something wrong until my wife explained 'he's a nutter', in the nicest possible terms. But, now it's like well, most friendships dwindle. Or it's just the suddenness of it. It's quite strange. But I don't dwell on it, it's just shows that those are important are the ones who have been in touch. People who live in Lincoln, have made a point that they've called up to see their Mum and they've made a point to come round and see you whereas people who live in the same town can be bothered to pick up the phone

INT1 And would you say that you've got quite a lot of support, besides your wife around you? Do you feel that if you were having a bit of a bad day, or you were feeling a little bit down, you'd have somebody to reach out to? Or would it just be your wife

Res Theres my wife, the kids both know I'm not right in the head anyway…I've got to stop using terms like that, even the consultants said it but again it come back to my sense of humour. If I was to feel, depressed, or anything like that, I know for a fact if we sent a message to my consultant secretary, there would be assistance available at any point. As for the assistance, we were promised, carers coming in and all this, barring an extra barrister and some rails, for getting off the toilet. And we've still got the ones waiting to go on the shower, obviously, COVID's put a big lump of that on the back burner, I mean they've come out and done stuff out the back garden so at least I can get through and if I do stumble, there's rails there. That's about the extent of the support. To be honest, I don't think it would have made that much of a difference. From what, from what I've got off, the love and everything else and the patience of me wife, she needs a medal bigger than I can give her and that's at the best of times. I don't think anybody else would have been able to do anything better.

INT2 If you obviously you said there about, if you felt depressed, or if you felt sad, to some extent you would feel okay talking to, or you would talk to obviously medical professionals potentially or you know, talk to other people. Would you do the same if you if you felt lonely? If you had feelings of loneliness? Do you feel you could talk to people about being lonely?

Res Oh, yeah. With being in the construction industry, as a scaffolder, I have about the embarrassment level of a dead rat. I'm quite open to discuss anything and everything. And people are like, oh god I wish he would shut up. There's no need to go into that detail. It's never ever bothered me to discuss feelings or anything.

INT2 That's it's interesting that you said embarrassment, because by meeting next question from that is do you think then, do you think some people would be embarrassed? Or do you think there is an element of embarrassment around talking about things like loneliness or about feelings?

Res Well, even amongst, shall we say people of a normal, normal disposition, when we've been out and about and they're embarrassed to tell about feelings, but how can anyone help you if you don't open up? With me? I think I've gotten to a point where I just don't care what anybody thinks, you either like me or you don't. To the point where Christmas 1992 I got restrained by two door stuff. Because I decided to do a striptease at a DJ stand just for the crack of it. I was in a good mood, it was Christmas and let's all enjoy it. And I was there, I got me shirt and my tie and my waistcoat off but as I attempted to get my trousers off realised I couldn't move because my feet weren't touching the floor. Please don't or we'd have to put you out. Okay then I'll put my clothes back on. But, um, I don't get embarrassed.

But like you said, I think within that there is a lot of people. I mean, my Dad, although he's a couple of generations older than me if you look at the way life's gone, he was always embarrassed. He could never… he felt…even towards the end he passed away with cancer. He was always a bit wary about saying I love you or me saying I love you. He knew I did and I knew he did, but to actually say it... He said it once. And he said it was drink fuelled. He said, Please don't ask me to repeat that. He said it was too hard to say. But that's just a generational thing. He knew what it is and vice versa. But I could understand certain people, how we could drive them to do something ill thought of, or ill thought out what I should say where they feel lonely or depressed and they just can't open up to someone. Where I would just put a cartoon on and it would sort me right out but that's just me. I've always been like that. Just comes back to the strange sense of humour.

INT1 Great. And do you think because you're so open, your boys will be the same?

Res Id like to think so. They are pretty much. It's been hard for them to see dad the way he is in one minute, I'll be happy, then the other minute be shouting at them. And then it's over and done with and then I'm trying to get a joke with them. And it's had to be pointed out you can't just switch like that, they won't understand fully at their age. But trying your best to cater for all needs. We're getting there. And they are quite open, we've sat down and had family meetings and you're…there's something bothering you, you know what were like we won't judge you just tell us what's bothering you and they've opened up. But we've said repeatedly you know if you've got any problems, we can't help you unless you tell us, and they have gone down that route and 'can I have a word with you without mum listening' or 'can I have a word without dad listening'. Either. You know that's…and I'll tell REDACTED and REDACTED will tell me. Don't say anything to your Dad or don't say anything to your Mum. But you know were going to tell each other anyway? And then it's like, well, Ive told you and don't tell anybody. Shhh!

You know, we like our hugs, whatever happens, whatever the situations been, however they've been. It's always night night, love you, see in the morning. We emphasise the fact that, night night, love you and its always kiss and cuddles before we go to bed. Even going to school before they, before they get… it used to be at the gates. But [REDACTED] has been that bit older and he's a little bit embarrassed about kissing mum and dad. So it's as we get

out the car. It's *kiss* see you tonight. Whereas [REDACTED] like me, he doesn't care. He's got no filters whatsoever.

INT1 Brilliant. Might be a bit of a difficult question for you to answer because there's so much overlap between your injury and lockdown. What would you say has helped you get through the lockdown and sort of not being able to see as many people as you'd want to or have that freedom because we've been quarantined or isolating?

Res I think without a doubt my previous mental disposition The fact that I'm daft as rags. I can't take anything serious. I mean even when we went to see the consultant is a prime example. This will have been six weeks after coming out of James Cook, for an appointment, and as we walked in there's a perfect epitome of a Norwegian God for want of a better word, you know, he looked about 12 but he was a student doctor, student nurse, whichever, he's in the blue scrubs, the full mask, bright piercing blue eyes, the sheer blond hair and I walked in, figured out straight away what he's on and I'm like sorry no. And she says this is such and such do you mind if we, and I said no, I'm not having anybody in. And he got up and you can see was a bit downhearted and he got off the bed and he literally got to there as he walked past and I was like, unless you go to Starbucks and get us a double mocha. Don't forget the cookie. And he looked at me and the consultant just said I should have known better, I shouldn't have warned him. But that's me. That's the only way you can learn by being in the room. If like I said in the beginning you know if what I've got and what we're going through can help the next person or the 10th person down the line. the only way they're going to get their help is through people like me saying jog on, look at my notes, look at the pictures look what happened. Interested in being famous or whatever, you want to take the time and cut my face out when you're showing the images of what was done, do it, I couldn't care less. It's the information that's important. But I think that mindset, always looking on the positive is, it's helped me greatly through life before all this. The times when you've, you felt a little bit lonely, and ill phone my day and he's been out sitting there and you're like I'll put a film on, or I'll go get a pizza or I'll go for a walk down the river side, it's that…everything happens for a reason, you know, there's always something you may not know what it is now. Somewhere down the line you'll go ah That's why you did that. That's why this has happened.

INT1 Just sort of in relation to that you've, you've talked about how much you want to help and try and support other people that are going through something similar. Have you reached out to any support groups, or anybody that has gone through a similar injury to yourself, and for support, or to discuss how they've managed things or anything like that?

Res Not long after id come out, one of the support systems was Andy's man club. Initially, because they didn't just you know, to see how you were and react and everything, you don't do a local one. So I was logged in one in Huddersfield. And from my perspective, they were suffering way more than me. There's people with some serious issues that would have benefited from it. Yes, I enjoyed the conversation and but it wasn't me. I don't believe I was that bad. I don't mean to sound negative at anybody else. But I don't think I was suffering as

serious as they needed it. So for me, I was taking somebody else's spot who could need it. But other than that in the back of my head It's still like a flu, Ive just got to ride the storm and recover. The one I've got, I've got this… It's not just encephalitis I have to be awkward. I've got LGI one, which is a very rare strain. So much that I'm number six in six year, who've got it in the UK or in our region, something like that. Hence, do you mind if we share your information? Jog on…Looking at the statistics? Normally, it's a lifetime. It's a long time. There's no time frame where you can say take these tablets for a year and that's you. But there have been some remarkable recoveries. Again, this is all information from the consultant, 93-year-old gentleman from down south. He made a full recovery in a year at 93. There was a lady with other medical issues, she made a full recovery in six years Another patient mid-50s. He made a recovery within two year but then had a massive relapse and still went on to recover. So with a bit of luck, it could be two or three years and I could be back to some sense of normality. If you can figure out what that is for me.

INT1 Definitely. And so back to lockdown. Again, this might be difficult for you to answer. Is there anything that lockdown has brought about that it's been particularly difficult for you.

Res Wearing a mask! Not so much that you get cold sores because you're sweating and all that…not that I don't clean my mask cos I can see you looking. But when you're walking about you're only seeing that much of people. And me and my wife were only on about this the other night. I was convinced I knew them all. Thats such and such and the person is looking at you like take a step back. I'm sorry, I don't know you. But I know you, I know you, you were in hospital with me. And it's because you're not getting the full picture it's quite strange because you look at people's eyes now. It's… I mean, that was… you're convinced you know them, I mean 100% and, again, that's triggered another thing. My wife used to say she'd not so much argue but I was convinced the sky is red or that sort of thing. If she said it was blue I'd be convinced that it was red. I mean, I would put a year's wages that that sky is red. It's very, very disconcerting when you, you're 100% sure that it's…it's like the lottery tonight. I'm 100% sure I'm going to win until I go to the shop tonight and the lass just goes no. Well you sold us it so it's your fault. But that's about it. Theres been nothing that's really triggered anything.

INT1 What about your thoughts about sort of lockdown easing and society getting back to everyday busyness, whatever the new normal is going to be? How do you feel about that, sort of life moving on again?

Res For me, it won't affect me anyway, I will still be going over the FFP2. Because what they said was in the hospital, my immune system was that strong, they've had to basically turn it off to get the medication in. And so the minute they started giving us any it was just kicking it out. So I've hardly got an immune system at all. So I've got to where the mask. To be on the safe side, I'd rather put a P2 on and look like a doctor who's escaped, than walk around with a doctor who sign on. Its for my protection as much as my families. Obviously, if something else happens, it's more strain on my wife and mum and the kids so it's up to me as well as then to make sure that I put the mask on, I wear it correctly, Ive got a beard but it's under the chin

line so my mask fits properly, them bits I do remember from doing my training and the things like that. Eating fruit, eating healthy, trying to cut down on chocolate. All that sort of stuff. So I don't think the reason lockdown will change or the way that certain parties are going on, we will have another one by September. Whether it's as severe as what we started with, or it's a partial lockdown, I would suspect we'll have another one by September.

INT1 Yeah, there's every chance, I'm hoping not.

Res Well once I've been to Costa Del Whitby, they can lock it down!

INT1 What about the social side of things? Do you think you'll be more sociable? And the friends that you still have got will you make more of an effort to go and see them than we currently are? Or could that not be a top priority for you?

Res No. Until I've been told not to shield by the consultant. My sisters busting to go for a cup of coffee. She's the eldest of the three of us. My brothers seven year older than me, my sister's 10 year older but her husband's got COPD as well. So the last thing they need is for me to pass anything on or vice versa. So until I've been told I'm alright to go out for a cup of coffee or something like that, the minute I can I'll drop a message and we'll go out have a cup of coffee. I mean she's been in touch. And if you need anything, and not that we've got much, but it's there if you need it and…that's what families for isn't it? But other than that not really. It'd be nice to think me and my wife could go out for a meal somewhere just for a change. But at the end of the day, we're all family. We don't care, what it is is what it is, if we all go out to the local, sort of whatever they're called, and we have a meal and we all go for a meal. It's the point that we're all together and no one's left to feel alone. That's what counts. Friends will be friends, whether you see them today or you see them in six months or …There's a mate of mine who live in the top of Welsh Wales, just near the border. And when we don't speak, maybe six, seven months, and then maybe I'll phone him or he'll phone me and it's like I saw him yesterday. Hes a bit ill at the moment. But I dropped him a text message and he responds when he responds, there's no 'you haven't responded'. It's just he responds when he feels like it. Because he's in a little bit of a dark place as well as the physical health. He's not as positive as me, shall we say that? I think that's the best way to describe it.

INT1 Yeah, definitely. It's nice that you you're there for him and that he knows that you're there if he wants to reach out to you. And REDACTED, do you have any more questions?

INT2 Just one based on what you've just said. Do you think your positivity is the, I guess the key thing? Or the key difference between you and your friend in terms of like your outlook on life? How you feel about things?

Res Yeah. Maybe some of the people that were in the hospital with us, I mean, they were in and out with different things. You could see the difference between, there was a lad next to me, who was, we were very similar in nature. You know, everything is a joke. We treat life as a joke. Although you…Although you take it seriously that you only have one life. But you don't take things seriously if you know what I mean. And then there's people coming in 'oh woe is me and it's all this…'. Youre in hospital mate. Youre surrounded by nurses, by staff who actually care. What are you whinging about? Someone's coming in, their feeding you, they're catering for your needs, looking after your health, they're talking to you, if there's any issues, they're going to bring someone in to talk to you. You've got nothing to worry about, there's people living on the streets, you're in a hospital in a warm bed, and you're complaining.

I don't get it. And the other lad was the same and then there was another lad that was severe epilepsy as well as other things. And he was a riot of laughter despite being severely ill, both mentally and physically. He was both of us put together in one person. I mean, you talk about positivity, you know, he was made out of it despite, he could manage to walk in the bathroom some days and other days he couldn't, he'd have to be taken in a wheelchair. But you couldn't put him down for anything. He was just …the minute he woke up, like me he was like wheeey, I'm awake, I'm alive, smashing, anything else we'll worry about it later. I definitely think positivity is one of the biggest things that keeps us going. Like what I said about work, I'm 100% positive I will return to work, whatever that work may be and until, don't know don't care. Ill worry about that later on. But until I'm told otherwise, unfortunately I cant work so, smashing, I'm sat on my backside, what am I gonna do. I'm gonna sit in the corner and cry or make useful time. I'll do online courses. And then at least when I am allowed to go back to work I've got that broader spectrum to look for work instead of thinking I'm tied because I'm a scaffolder, I only know scaffolding. No, I don't. I know more about construction. I know more about health and safety. I know more about food products. I know more about working outside and everything else. How to deal with people, how to talk to people, which I've always done. As you can probably tell it's, it's easy to get me talking, it's a nightmare to get me to shut up.

INT1 Brilliant. I think positivity can be very powerful, can't it. And it's amazing what it can do for you for the soul.

Res Not at 6 o'clock in the morning when I wake up and I start talking and the wife thinks just go and make me a cuppa

INT1 Yeah, I might agree with your wife on that one. I think that might be a little bit too much even for me.

Interviewer(s): **INT1**

INT2

Respondent(s): RES (P8)

RES My accident, my head injury my accident was while I was on duty in the Royal Air Force. That's why I've done a a a testimony of about 10 pages of. um And it's called life changing injuries. Very often on the news you hear people going on about in life changing injuries, well they are. You wake up in hospital with a foot missing three weeks after they've been digging in the back of your head to sort out the bruised brain while they're waiting for a helicopter to take you back to camp. Right. And then you start meeting up with guys who have come back from Afghanistan with more than one leg missing. And you think I'm actually just scratched because these guys have been blown up in the air and lost limbs and I been lying in my bath looking at my one foot instead of two feet. And the Airforce kept me on for two more years because they didn't know what to do with me. I went back to my old job because they didn't know what to do with me. They sent me down to a place called Headley Court, which is a joint services hospital now in Surrey, or Kent or somewhere down south. Um To assess my epilepsy um schedule, I suppose you'd call it. I didn't have any seizures, so he sent me back to work. Um. And I've had two seizures since my head injury um and the biggest problem there is losing your driving licence. And when at the time you're a motorcyclist and a passionate motorcyclists, and they take your licence off you once you've had your motorbike converted, so you change gear with your thumb rather than your foot. It's annoying. Um. And then when you discover that you've got anger management problems because of your head injury, it just it adds to it. Okay. Um... We are very lucky in Norfolk, we have not had in a rural area, we have not had problems with COVID that many of the cities have had. We have been very, there has been adjustments to what we can do. We can't go out already go go through my list. Right? The things that have changed, changed during lockdown. Church was closed. I'm a Christian. The swimming pool we used was closed. No real social, no real loss of social life. But then there wasn't a great deal of social life beforehand. My partner was unable to travel to Coventry to visit her family through the Easter break sort of thing. So I was fortunate enough to have a companion in the house with me throughout lockdown, company. Um I was unable to obviously because my my licence was missing. I'd started using the buses everywhere anyway. And then that's the fact I'd lost my licence all of a sudden wasn't such a big issue because I was staying at home anyway. Um . And then finally, because I have they discovered I have a high iron count in my blood. I was having to go up to the Norfolk and Norwich hospital every week to give blood to the in the Hema Talat haematology department. Um And that got postponed at Christmas as well. However, come lockdown, I would have said, well, let's not bother with this because it's not a life threatening condition, situation. And the nursing team that I used to see for my venesection would probably be better used elsewhere in the hospital, because they're all qualified and very capable nurses. Um the supermarket stayed open. My partner was able to go shopping, she's older than me, she was able to get in as the OAP the vulnerable people

side of things, I think they call it she has her own vehicle. So there was always food in the fridge, there was a roof over my head. There was somebody to talk to. Sometimes that got very difficult and a very, very difficult um stress and strain on our relationship.,, Um. But the COVID lockdown has not probably been as difficult for me as for people in other places in the country. Even now we're hearing about schools being closed down till September, in Bolton or somewhere. Um..My parents live locally. In REDACTED. I wasn't able to get over and visit them. But that's okay. Because I've lived the other side of the world and many, many years living in Scotland. So, you know that they're there on the end of the telephone. They're both in their 80s. And they're doing very well. They were some of the first people to have their vaccinations. So they're safe in that respect. It's been an adjustment.

INT1 **Do you find that it's had negative aspects to lock down for you? So you, obviously you, your partner was at home, but that also caused additional pressures on your relationship. Other than that, you've got on with it. Do you think? Have you found it difficult or have you found it quite easy.**

RES Um, it was a shame not being able to carry on using my car. Because I've been a motorist and a motorcyclist since I was 16-17 years old, right the way through my time in the Airforce, so it was a shame not to be able to get out and about in the car. Um... (sigh) it's not as though I've got the stresses and strains of employment. You see, you hear some horror stories from people that were losing jobs and things. But because I'm medically discharged from the military, I get a veterans I get full veterans pension. I also get an RAF pension for serving nine years. This money every month, tallies up to something like what hospital or school cleaner would earn. Okay, so I've not even been um denied my the the threat of losing my job, and the income and all these sorts of thing. Very, very fortunate.... um. negative aspects um nothing that… (sigh)…. I've been through some shit in the past. And COVID is being dealt with very, very well. In this country, when you watch the news. I'm a bit of a geek for news and politics and things. We are doing very, very well. I'm very, very proud to be British. I didn't serve overseas. I served in Scotland. I didn't serve overseas, but we have something that we should almost be passing on now to places like India. It is horrific over there. Bodies stacked high. I had friends in the army who said when they came back from Eastern Europe when the communist wall dropped. And they you know, warehouses and things body stacked high there, children. It's not nice. I have been.. i've muddled through it. Yeah, we've had some sometimes the worst thing of the day has been the weather. Right. I live in REDACTED. REDACTED is in a dip. Mobile phones don't work here because there's no signal. Hoorah Yes, I'm retired. I'm early retirement. I'm not if I go back to work, I lose all my benefits. Right. But head injury I've got a book full of engineering certificates. That my head injury stops me going to work in case I have a fit in a hangar, on an aeroplane, in a workshop, on the production line. So I'm knackered in that respect.... um.. (sigh) It's been an adjustment he's having to adjust. There's been stresses and strains on our relationship between myself and the partner. And most of the time we've been able to diagnose it down to problems with me because of my head injury. Headway has been the first brotherhood I have met since leaving the military, my accident my head injury was 1992 I finished with the REDACTED in 1994. That's a long time to be going around on your own.

INT1 Can you tell me a little bit more about your your head injury and how it affected your daily life?

RES Um, I was rock climbing. I was a member of the the REDACTED. And I was rock climbing and we were on a face near um where would you know, Sterling, perhaps? Um a training Day at Wednesday, we normally go out Friday off evenings and come back Sunday night. We've done the weekend. Picking up people who get lost and hurt and things on the Scottish mountains coordinating um with another team up um REDACTED, and several search and rescue helicopters buzzing about picking people up and things. um. The people go up Ben Nevis in flip flops with an A to Z of Fort William. It's on a track called the tourist route. Hmm, yeah, Snowden probably has the same I don't know. But Ben Nevis is a beauty a beautiful mountain. The Scottish hills are beautiful. We rock climbing on a Wednesday, and I was leading the route up, putting protection in leading the route up. And the next thing I knew... um probably three weeks later in hospital, the rock faces broken with me on it. myself with the protection I'd put in at all slid down avalanched about 35-40 feet. my helmet must have moved on to my face because I bashed the back of my skull and loose rocks like rubble landed on my feet, smashing my left foot and ankle.... um. The guys did what they could, we were there in a Landrover, we had they had what equipment they could go to first aid and everything. The first aiding we have done is has been excellent standard. They then gave up and said no, we need we need to get him into hospital. So they called Camp who then arrange the helicopter. And I was winched and taken into the when the pilot was told about my head injury. He said Southern General, because um the Southern General is the best neuro ward in Scotland. And I don't know how many minutes flight but it's a lot quicker in a helicopter than an ambulance. I was landed there, um my parents must have been phoned by somebody and notified REDACTED sent a car and a driver to get my parents to bring them up to Glasgow. Um my parents then had the debate about um what we're going to do with his leg, he's going to be left with a stump a mangled foot, he'll be in crutches in a wheelchair, all sorts of things, or we can cut his leg off under his knee. And we fit him over sensation of time a prosthetic leg. So my father elected for the amputation. I'm an only child. I was 24 years old. I just come through a marriage and a divorce. Um you don't need these things do you?.... um.. Many going on.

INT2 ***No I was gonna say feel free to tell us anything that you want. This isn't a erm a situation where we're going to stop you in terms of err being as open as you want to be. Emm so don't feel like you have to stick to the script of lockdown or anything like that***

RES is the one I'm trying to sort of perhaps say is that, life has been such a Higgledy Piggledy of things. I've been a student several times since, I've had several jobs since several reasonably good jobs but, but that life has been Higgledy Piggledy before COVID started.

INT2 Yeah, sounds like you've been through. You've had you've had a lot of life experiences.

RES Yes. Yeah. So COVID the negative side of things. Yes, there has been, I would say to emphasise the real negative side ... has been issues perhaps between myself and my partner.

INT2 Okay. I want to come on to that in a little bit. wondering, obviously, we've spoken a little bit about your brain injury and how it happened. And you spoke yourself about, you know, the, the anger management aspects of it. So there was obviously a personality change. Or, you know, there was some aspects of the brain injury in terms of personality.

RES Yeah.

INT2 What were there any other issues that you experienced because of the brain injury? Were there any sort of, you know, the, the the compendium of issues that people experienced with a brain injury? What kind of things did you experience?

RES The the epilepsy being accused, err not accused, or convicted, perhaps, of having epilepsy. When you're an aircraft engineer, and it stops your career is, like I said, I was it was two more years before the RAF discharged me. They sent me down to Headley court. Joint service, joint it is now anyway, joint services Hospital in Kent, erm to assess my epilepsy, and I was there for 8, 10 weeks, and I didn't have a seizure. So they sent me back to Scotland and I went back to work with the aeroplanes. Um the… I then went into education I went into Edinburgh University did the first year there as a on an engineering course. um And from there I had to pack that up because my mathematics is not I can talk to you until you were bored with me about a gas gas um turbine theory. You asked me about calculus ill tell you he is a Greek god. Okay. The…. several other education including motorcycling, I qualified as a motorcycle mechanic. I then went on to do automotive engineering studies, um in Edinburgh. This was while I was living in Edinburgh, and worked had several jobs in Edinburgh in the motor trade,,, Um But the more application forms I was filling out, usually on page three, tell us about your disabilities and what medication you're on. There was a discrimination, I felt very much actually the RAF was almost sweeping me under like, it's not a problem now he's gone. Um One of my headway, workshops is paid for by Safa from the RF Benevolent Fund. The military is still there looking after me. Now that's very reassuring and very complimentary, almost, isn't it? Um I'm also I'm early retirement, I make model aeroplanes, I make models. I have a garden, I'm not really interested in the garden, but I will get green fingers one day um.... (sigh)... just taking over an existing my faith, I became a Christian after my accident Um. And my faith has slipped, perhaps my faith has slipped a little bit as a negative. While we've been here and not been able to get to fellowship with people from church, I've not been living in this house many years. So we've not really got to know the people in the church very well, this particular church. Um... But my survival is down to a greater being. Um, and I can only now try and get on and behave myself and continue um as well, as well as I can. And put the dark past behind me... um…. COVID has been almost from here entertainment.... Because Norfolk has had so little um... I mean, we only have three hospitals with A&E departments. Two of those have helipads. So Norfolk is

not a great, but the hospitals are working very, very well. But everybody's agreeing now that the NHS are wonder beings, you know, that's that's fine..... um....My experiences in COVID I'm reading your your line along the top of the message. I've had a one go one.

INT2 I was gonna say we can come back to that if you want.

RES Yeah.

INT2 And we can we can touch on that in a little bit, rather than getting directly into that bit as well. Em obviously spoke about your Is it your wife? Is it? Is it? Do you live with your wife?

RES No she's now (laughs) this is another little funny story. She's actually still my partner legally. But we both have the same family name. So when I book a table at a restaurant, it's Mr. or Mrs. REDACTED. Because she's Mrs. REDACTED, you see, so she was married for many months. She's a grandma married for many, many years. um And we just get on as husband and wife. It could easily be assumed as husband and wife going out sort of thing. Um.. We don't have any there's no animosity between me in any of her family. She's got three grandsons. One is doing very, very well at in Cheltenham as an apprentice and the other two are little boys one of whom is right little rascal and needs um some talking to but um I get on with the rest of the family. Some of the family were over and visited in the other day, and I make a point I've always been believed in in families and things. When you're in the airforce come Christmas time, the guys you've been working beside, you see them next to their wives and with their children. And you think this family is what the armed forces is all about. It's not single people. It's its families. And that's what we're working for. And that's what we're defending..... There's more air cadets now than there is people in the Royal Air Force. The Air Force is small. Yeah. And I would imagine the Royal Navy and the army are going to shrink same as well. Because it's money, isn't it? Yeah. We're not worth anything. It's money.

INT1 Yeah, If I can just get back in time to after your, your head injury, the hospital or do you have any rehabilitation at all? Is there anything that you need it for your head injury, not for your foot....

RES Not..No, I don't think so. Interesting question. I don't think so. No. Certainly, I was using the Dundee limb fitting centre was back home because um your your residual limb is still shrinking from the swelling. And you have to have several different legs made over a period of um two, three years. And your backwards and forwards there all the time? Sort of tune ups and adjustments and things? um... (sigh) I don't think there was.... There wasn't certainly people, a lot of people over the 8 years or so um. after my accident, didn't, a lot of people recommending and suggesting headway. When I was shrugging off? No, no, no, I don't need headway I'm alright. But I'd lived out and ended up living out in Canada for four years and came back from Canada. And I thought right that one was head let's go and investigate

headway. I was actually introduced to headway by somebody when I was on a head injuries um unit near Colchester. And they said, Oh, headways, you know, and I had a look. And that's all this is quite good. And I followed it up in the Norwich department. And it's I've realised now is that there's a fellowship of brotherhood there of people. It's not obviously just head head injuries, and very, very few head injuries. A lot of it is strokes and brain tumours. But you're with the same um background of people. You know, and that's fine.

INT1 **And do you find… Sorry go on**

RES That's it. That's also.

INT1 **And have you found that since you've connected with headway, have you found sort of that support of other people that have had um either head injuries or stokes and things? Do you find that that's helped to be able to talk to them and share sort of what you're going through?**

RES I think so. Yes. Yes. It's… um... it's very useful to the activities and things where um, if you feel you want to stop, you can stop. I mean, the zoom meetings we've had for the cogwheels I mean, some weeks there's been nine or 10 perhaps people attending, sometimes there's just been three or four of us. But you come away from it, feeling really good. Really had a really good laugh. You know, what I do miss is things like going down the pub with my mates for few beers on the Friday. But that's when you're in industry. And you, you thrive on that you're not it's not the alcohol you're after, it's the Brotherhood.... um. And the zoom meetings, the zoom meetings have been adequate. Right? They are not like being at the centre. You know, I just started a woodwork workshop at the centre. um. And that's really good because I'm, I'm a technician. I'll put aeroplanes up in the sky, you know um? And that's, it's good. It's good fun to be with two or three other people who are doing the same sort of things we would, here they have a cookery and an art, different art paintings and crafts and things. It's quite a good centre the Norwich Centre. Um You know, but the the regular rules and regulations obviously, they're going up and down dramatically (laughs). So it was it was deemed that no, you had to have both vaccinations and there'll be a limited number of people available Each day, um the timetable is still full, Monday mornings and afternoons is now full for workshops um. And so it is run by several three or four senior OT's and one or two other people who are sort of branch managers who were clients themselves um and it's very well.... But you don't, but they don't have to you don't have to climb inside each other's head to know there's a bit missing, perhaps, you know, um it's... losing a limb was inconvenient. Losing a limb, I had to get my motorbike and modified and it was a bit of a problem thing. And I couldn't get up as many hills as we used to do. The head injury from a technical person's point of view is the real disability. That has been the real weight on my shoulders. No, you can't do this. You can't do that. In case you have a seizure in case you have a seizure. Well in 28 years two seizures and proves the tablets work. You know, and DVLA when they say 12 months my fit was in October. I wasn't driving again until two

January's later. Because they work walkthrough toffee in their the headquarters. Um That's the way the way the world is isn't it?

INT1 **Em When you was speaking about headway and been able to get face to face and things like like the woodwork courses and things like that, if we think back to before lockdown, was that something you were doing regularly anyway, were you quite involved in with different courses and things that would help what headway or or otherwise?**

RES No, not really, I was doing the cogwheels course because a lot of it had to do with funding. And the first workshop every week, I was paying for myself. Um And then my OT who um I suppose is sort of like a mentor. She said all this communication um group would be quite good for you meeting people, we're not actually doing a great deal when you when you meet them. Um.. And we I said Well, how do we get on with that? And she said, Well, what if you give me the names and everything for the people at safa will that make an application and see if we can get funding for that? From the RAF Benevolent Fund, which happened because I'm doing two er a week now. Um So... so that's a very good thing that limited me to what was going on. Um But then if there's not many staff, and there's a fair turnover every week of people, you can't be taking the denying other people's place sometimes, you know, a lot of the things that wouldn't be interested in me the craft and the cookery and things wouldn't be interesting. Um It's the more... um... technical um... and err not academic things but of that academic nature. That will be invested interest in me, to do to go up as I said, the city that side of the city is 25 minutes drive away. So um it was err every it's just it's it's a start for what I'm going to be doing with Headway I think. In the years to come I think I'll be doing more and more headway as we get on. You've frozen up on me now

INT1 **Oh,**

RES yeah.

INT1 **Am I back?**

RES No, you've probably heard me. There you go...

INT1 **I can hear you**

RES Yeah.

INT1 **Brilliant. Brilliant. So headway seems like it's been a really positive sort of experience for you. And it sounds like going forward is going to open up extra avenues and things. Were you were happy with the amount of activities and socialising before we went into lockdown to get a good social life?**

RES No, I didn't. It was now that's not fair. Really. Um….. I've slipped further and further away is the sort of social lifestyle I used to have um. Possibly because I'm not a student anymore. And I'm not employed anymore. So I haven't got workmates and I haven't got student mates to go out for beer with have a laugh with whatever, go camping, walk in with whatever students and people do now, you know That I've sort of gone past perhaps, you know (sigh) it's difficult.

INT1 **How do you feel about that? How do you feel about the fact that you've lost friends? mainly due to circumstances?**

RES Well, the the military, everyone will tell you is a revolving door. People go to sea together they come home they promise they'll stay in touch they walk away from each other. And that's it. um.... sigh....The....men who I still have most affection for um were older than me, higher ranks than me, ever so ever so slightly senior to me um. They I'm sure by now will be the out of the military and um hopefully they got themselves into employment that was helping them continue with their families... um... You can only wonder what is going on. um...The...going to I used to go to sort of go to reunions Mountain Rescue reunions particularly um .... And after 40 minutes sat there, you've sat down and spoken about everything and caught up with everything from everybody from the previous year. And it's well, what shall we talk, what should we do now? And reunions get to be a bit. Some people love them the time and effort Some people put in to organising reunions every year. Fantastic. But it's not for me.... that's the way it is... you know em...

INT1 **And how do you think you would define the term loneliness?**

RES Um… cor You see, … you probably see, I'm sat next to things like that (Holds up a book), right?

INT2 ***Just remember that this is your definition. No, this is what that means to you rather than?***

RES Yeah. um Loneliness is .... you don't see um... I never see people now. I have a companion in the house with me. And if I want to go talk to somebody and, and hang out and have a cup of tea and a piece of cake or somebody with somebody my parents are 20 minutes down the road um ... I haven't got the physical strength to be a junior technician anymore. I've got a limb missing.... I'm also liability to an employer in case I have a seizure and hurt myself or...

um ... ultimately, they said if you're working on an aircraft and you had a seizure and you fell off the wing, you land on a concrete floor or somebody else and hurt them as well. So the liability of the head injury has um sort of grated me up a little bit. Hasn't it.... um.. loneliness. Yeah, um…. I enjoy. I did enjoy group activities, campings, Mountain Rescue certainly, there was 15-20 of us on the team, um most weekends we all went out together as a group. Um we don't do that now. Um I only worked with half a dozen other guys on my particularly in my department if you like um,...I mean, I read a read an awful lot. Now that I didn't do before um, I try and pick and choose what television to watch because most of it is utter rubbish um....

INT1 **Are those things sort of the reading and the television are those things that have increased since we've been in lockdown, or have they increased gradually over the years anyway?**

RES They've increased gradually over the years, the reading, I think has increased gradually over the years anyway. um In fact, during lockdown, there was one or two in my books that I've actually reread again um but err....

INT1 **Do you think your em perceptions of loneliness have changed over lockdown? Obviously you mentioned about the fact you've got the companion at the moment and your parents are close by? Do you think you'd have thought the same way before lockdown?**

RES ..... No, I don't think so. Um..... there's a long line of bus stops in Norwich in the middle of Norwich outside Debenhams, and two doorways that always have cardboard boxes and sleeping bags. And you watch the people there. Or you think of the people there. And that's hard work, whether they, what their story is, it's, it's so sad. It's a sad state. In Canada, where you get used to get all your cash points behind the sliding door. So the cash point doesn't get snowed up, iced up, you'll get people sleeping behind there. But they're always wearing a $200 - $300 ski jacket, you know, so they're not actually that bad, although they're making out that they're homeless um.... That's loneliness. But being.... through lockdown is possibly taught loneliness. Brought loneliness home, if you like to a lot of people, but they won't actually admit it. You know?

INT1 **Do you think em it has for yourself? Do you feel that it's brought lockdown's brought loneliness for you?**

RES (Sigh) ..... Possibly, but at the same time, it's given me.... um reading time, thinking time, doing time. I've made a lot of plastic aeroplanes, model aeroplanes since the.... I'm not out in my car anymore, listening to music. Um, but then I'm not spending money every time you go out in the car, you spend money on petrol don't ya. But I've been saving money to put towards things like doing bits and pieces in the house. You know, we've just had a new bathroom last year, and then bits and pieces in the kitchen. The kitchen is going to be next.

That is all need to be ripped out at some stage and replaced. So if we keep the money back for that, rather than it just frittering it away on petrol.... um... You have to juggle things you have to juggle your time, don't you?

INT1 Do you feel like you've adjusted quite well to juggling your time do you feel like when you look back now, the fact that you've been had all this free time that you've been able to do perhaps some of the things that you couldn't have done before?

RES Um I don't know. Some of the things I tell you I am looking forward to the centre reopening properly. The headway Centre in Norwich reopening I'm looking forward to that

INT1 I bet, Yeah

RES To get back in proper contact with people in that respect. This has been um a obviously not a holiday, but a pause in things a time to reflect and sort for people to sort themselves out perhaps. Um. It's a difficult one, there's this this has been an opportunity for an awful lot of people to adjust and prepare to start achieving things.

INT2 Do you think if you did feel lonely, or you did feel a certain way in relation to isolation or these types of things, do you feel like you could talk to someone about it?

RES …….. (Sigh) No, I don't think so.

INT2 Do you mind if I ask why?

RES Because nobody knows me... um. In the past, when I've done things like run the Samaritans, you have to start day one of my climbing accident. A Head Injury a leg missing 3, 6, 9 weeks in hospital, buggering about backwards and forwards to hospital to get a new leg to fit. All you're doing is going over you pulling the black clouds back over your head from the past. um... What I would need is for your work mate who could say "come on, go for a beer or cup of tea it's cup of tea now init and they come over and we'll go for a cup of tea"..... um... Yeah, I would need a work mate even in in student days, it was difficult sometimes because there weren't that many of the students old enough to who were interested in the course who I was getting to know um... that you could share things with. They will busy playing on Facebook, you know? um... No it will be a very difficult um… there's one or two things I'm up to at the moment that are still Yeah. No, just at the moment. No, I'm trying to make arrangements where there will be somebody who I could talk to. Okay. um But I see a dip in my well being self being side of things um..... You know, once they get the pool open again, and we start swimming again. And we've had a couple of visits back to church. But we've

we've changed the moving church to our local town rather than the previous one. It's sort of getting to know the people there a little bit more. Yeah, having to be more independent about things stronger, perhaps about things. Perhaps it's making me stronger.

INT1 **Following on from Stevens questions you mentioned about other people not being able to talk about if they were feeling lonely, do you feel you're the friend that you're you're looking for to share with? Could you open up and start a conversation with somebody else that you felt they might be feeling a bit down or a bit lonely? Or do you still sort of think it's not something that you'd be comfortable sharing and talking about**

RES No, That I think in the past somewhere, vaguely I remember, I've tried in the past, and you spiral further downwards....You get to talk to somebody like a minister at a church, and they will help bring you up. Somebody who's on the way down themselves is already on this spiral downwards and you end up no...

INT1 **and then, is there anything that you can think of that has helped you over locked down, has kept you kept going kept you motivated?**

RES Bank balance (laugh)

INT1 **(laugh)**

RES the time and opportunity the reading time, but they're sort of reflecting, thinking time um...What are the opportunities now to when we get out the door? What are the opportunities to now to sort of go and do? um...Like I said I'm not green fingered at all, I've got a reasonable little garden at the back of the house that could have some attention... um... It would if I wish I could wake up with a passion for gardening erm... but.... Positive from lockdown.... um...

INT1 **Or anything that you think helped pass the time or get through each day.**

RES I've enjoyed doing, doing my modelmaking I've enjoyed that a lot. I've now set up a workshop of sorts, in our shed garden shed a union or work my work used to be here. But it was pack it all away, get the laptop out for zoom meetings? Well, I've got my work, modelling down in the shed, I keep on keep on going modelling in the shed, you know this in my modelling shirt sort of thing, you know, um... at err the....no preparation, our thing is that is this is the easiest answer.

INT1 **Yeah? And then the flip side, is there anything specific that's been exceptionally har**d?

RES um....Again, sadly, no. Because I've been up and down in in and out hospitals and things all the time. For some years before that. You know, um... just errr being empathy, empathetic towards my partner. She's very capable home sort of management type person, and helping her ensuring her that she's, you know, coping all right with with things as far as the grocery shopping and things go um...We've being been…. Yeah, we've been very fortunate if it was getting her to see her family back at Easter time. Um I mean, twice before that she'd taken my car, the nicer the two cars, to see her son and daughter and families and things in Coventry. But they sort of say when are you can't be travelling between areas. And that was a bit of annoyance. But err…..

INT1 **I think I already know the answer. But I'll just check. How are you feeling about after lockdown, sort of getting back to your normal daily activities**

RES Doing things perhaps sort of in the right order. So it's time management side of things, or we've got all this free time. Now let's make sure that we do get on and do things in but so it's done in a logical order and a nice, neat and tidy way.

INT1 **Are you looking forward to sort of going back to face to face and putting the laptop away? No more zoom calls? Or are you a little bit some of it's been quite handy.**

RES It's been it's been useful to keep or not been involved long with Norwich headway. It's been useful to keep um… in contact with him for the the the threads the strands, I was beginning to build with the meetings and the workshops and things there. The Zoom has been useful for that. But it will be nice to get back to the face to face. I mean, I don't mind driving in and out of Norwich I mean that's easy enough. It's not a difficult city um..... but err No, I don't think sorry. What was the question? Again? I'm going off track. I think

INT1 **it's all right. It's just how you're feeling about sort of post lockdown. How are you feeling about getting back out?**

RES I'm quite quite excited as it's a bit nervous but trying to get things in, in a in an order of err job list of things. Um So I can use my time management sensibly and, and successfully. I know even more money for things like do bits and pieces in the garden to do nice things in the garden um is... just to be. What I don't like is um failure and I don't like mistakes, which cause stress and frustration. And then I can get angry with things and angry with people for no apparent reason, and be really, really nasty. And I hate that side of myself, if they could have amputated that piece out of the back of my brain. Which you can't do. I've had psychiatry sit down and say, we don't know anything about the brain. um... So I've got this

my partner says is it's like a light switch. You're like Jekyll and Hyde, sometimes, I hate myself for that. But that's another story. We're not going to discuss that now. That's okay. No.... it's to move forward, the whole country, the whole world is going to benefit from this. This this trauma, this this difficulty, and the loss people have suffered and the problems with their jobs and things is incredible. But at the moment, you go out and you walk around on the streets and it's like an almost an atmosphere of VE- day. You've got I've got we've got to use this wisely. For the future, I think.

INT1 **Yeah, definitely. Steven, do you have any questions at all?**

CONFIDENTIAL

Interviewer(s): ***INT1***
INT2

Respondent(s): RES (P9)

INT1 And then can I ask, would you mind telling us a little bit about your brain injury?

Res Well, it was Friday night and we were out down at the local public house, it was my Mums 92nd birthday. So I excused myself and went to the toilet. When I came back, Ive got 2 cousins and they were nurses and they said 'there's something wrong with REDACTED'. They spotted it right away. My brother just came (unclear) and walked me out the door, put in the car, carried me upstairs, put me to bed, phoned an ambulance and before I knew it that was me on my road to the Victoria Infirmary. So, they ended up taking me to hospital…(unclear)…and that was me. I was a bit confused about what had happened (unclear). No great pains, no collapsing. I think it was the ambulance men who were by the side of the bed who said 'alright REDACTED Just calm down'. I said 'aye Im okay, I feel okay.' But obviously you're not…(unclear). So when they took me down to the ambulance they put me on the bed (unclear). So we went to the new Victoria, then we went to the old Victoria. I said look pal, you're guaranteed to not get a tip. And then they took me down by Queens's park and then along [REDACTED] and we headed up to the mansion house. So that was me admitted.

(coughing)…excuse me

The ambulance men carried me and some porters then took over. I said, right lads watch that driver as he will take you the long way. He didn't realise (unclear). So he's not getting any extra. So basically that was me admitted into the Mansion House. I'm not quite sure but I think I was there for basically 6 months. But obviously if you listen to the ambulance drivers (unclear)

INT1 So while you were in the hospital for the six months did you undergo some rehabilitation? Did you notice sort of a big difference to the amount of activities that you could do?

Res My walking wasn't very good. My balance…my eye sights affected now, paralysis down my left side. Basically, I was just lying in a bed. I saw my family and my ex-boss came up to see me which was really nice

INT1 Did you have any sort of medical help to help with your walking and your balance again or anything like that whilst you were either in the hospital or when you came home?

Res Occupational therapists, (unclear) maybe walked round to Asda or something. Just so there's a wee bit of support. Occupational therapists gave me the information for Headway Glasgow. Basically, I never looked back since. What a place. Absolutely tremendous. I mean I've developed skills I never thought I had. You know like I go to the writing groups. I go to the art group the discussion group (unclear). The thing about Headway is everybody's same. I mean there's no one (unclear) one or the other. When I first went to it, it was (REDACTED) who was in charge. We were based in the [REDACTED] at [REDACTED]. On St REDACTEDs day the year before a big steeple of the church collapsed and fell on the church. So I can say whoever looked at the church went, smart idea, I'll make offices and rooms, so they made different rooms and (unclear) so that they can rent them out. So that was Headway that moved into them and after that I never really looked back. The first day I was there it was down the stairs in a very big room with a stage in. There was a lot of people and a lot noise and they were playing pool and dominos, just having chats, but everything just seemed to (unclear) but unfortunately I met a friend there and just started to talk to this boy, a lovely guy, just started to talk to him (unclear) but it soon goes away don't worry about it. So I looked at my watch and thought I better go out and get (unclear). So I walked out, upstairs, seen [REDACTED] sitting in a car, I walked up the stairs and down the stairs. I said I'm just going to treat Headway, just embrace it, the way it's meant. And from that day forward, ever since Ive just wholeheartedly done my best. Went to this group. Went to that group. I went to the art group and I couldn't even draw (unclear) never mind draw a picture. Like I said I just embraced Headway. They're not gonna ask me to do something that's going to be harmful. And surprise, surprise, the tutor calls me Salvador Dali of Glasgow. So you didn't realise I was that famous did you?

INT1 Brilliant. So you obviously enjoyed it. Can I just check how long ago was it that you had your brain injury?

Res About six and a half years, six and a half years

INT1 And have you found that headway has been really beneficial during lockdown as well? Have you managed to still keep in touch through sort of video calls and things like this or other means?

Res Yeah. We have zoom meetings. But as I say, there's things that have been happening in Headway Glasgow just now. Tutors leaving and different things. It's not in turmoil but its stalling. Weve got a meeting this week. I have found (unclear) As my mother used to say to everybody, treat everybody as they like treat you. So (unclear) I've just tried my best. Some people say I'm very trying. But at least I'm trying

INT1 Definitely. Sounds like your Mum had some very good advice. What about anybody else that supported you during lockdown? Have you still been able to keep up with any other people that you've seen on a regular basis?

Res Yeah. Ive got my friend [REDACTED]. Were in contact most days or nights. Unfortunately, other friends and that have passed away.

INT1 And have you noticed a big difference in your social connections whilst we've been in lockdown? Has there been a drop in the amount of people you've been able to see?

Res Let's see. I would say my family at Headway because you make friends (unclear). And I'll tell you how best to see all the other good

INT1 And how have you found lockdown? Have you found it Okay, or have you found it quite difficult?

Res No, I know I was I was okay with it. See, my pals are very protective. And they let me go out on the veranda and I just sit out there. Everybody 'how are you doing pal, blah blah blah'. (unclear)

INT1 And have you been…erm…What kind of things you've been doing to keep yourself busy whilst we've been in lockdown?

Res As I say I'm very (unclear). At Headway we got a new tutor for the writing. Hes only a young boy…an exceptional boy. (unclear) He normally sends us prompts, like a picture, and it's amazing. Youre maybe talking say 7-10 people, all different and all write the same story. And [REDACTED) has this knack, really beautiful ideas. So I don't know if you know this but 2 weeks ago was National Nurses Day, so we're on Zoom and (REDACTED} says (unclear) and he says 'right, we've heard each others writing so is there anything there that jumps out at you?). So he goes round the group and says what about yourself. No. (unclear) Ive brought a short video. Named seasons of the mind. [REDACTED] put a sentence in, so [REDACTED] takes the sentence using the intro, moves it down, (unclear). But some of them have came out, from complete strangers. Just from a prompt, a wee photograph. You couldn't believe the content. Absolutely beautiful. (unclear) I was saying to the nurses, I go to hospital quite regular, more or less every week. (unclear) A blood (unclear) in iron. So I had to go to (unclear). And they do my bloods. A pint here, a couple of pints there (unclear). I surprise myself with everything I've tried. I just embraced Headway in the way that its meant and I just went on and on. (unclear) Funny enough (unclear).

INT1 Was that something that you had done previously? Or was this something brand new?

Res The writing is new

INT1 Are you enjoying it?

Res Oh yeah.

INT1 And what about anything besides headway that you've been doing at all? Anything else besides the things you've been doing with headway? Have you been doing anything else at all?

Res Not really, I've got [REDACTED] who's a Support Manager for Headway UK. So he involved a good few of us (unclear) The church is not really suitable for wheelchairs and such. It's just like a rabbits warren. Even now I get lost in it. Apart from just sitting out on the veranda and any neighbours that go past just saying hello and that

INT1 And if we think about sort of the term loneliness, how would you define it?

Res Yeah, I don't know. Is lonely not the one in Taggart? Maybe I'm getting mixed up

INT1 I don't watch Taggart I'm afraid. You're showing your age there REDACTED. I'm far too young to know what Taggart is.

Res I think you’re having me on

INT1 So your feelings about the word loneliness?

Res See because of my arm and because of my writing, a New York policeman said to me when I was over. What you see is what you get. Me and my friend (unclear) Say I couldn’t accept it (unclear) But loneliness, I don’t really suffer much

INT1 Would you say you've always felt like that. It's never been an issue for you?

Res Yeah

INT1 Then if we think about lockdown, is there anything that you would say has helped you through?

Res Zoom and Headway. My friends on the phone

INT1 Were you using technology like zoom before we went into lockdown or is that something that you've just used?

Res No. Because of lockdown. You should see my idea of technology. You’ll say you’re too young. But Dr. REDACTED might know. We used to use a telephone and it was press button there, press button B. that’s my total grasp of technology

INT1 Ah you see you're too modern for me because my telephone used to have the dial used to have to spin all the way round. You must have been posh up there in Glasgow

Res No I used to use public telephones that used to be on the street you’d put your forms in. So if you get through to a number you called you’d press button A. But if you wanted your money back you’d press button B. It was always button B for me.

INT1 And again thinking about lockdown Is there anything that you found particularly difficult

Res Just going out. (unclear) I can’t remember the last time I’ve seen my son. But I think it’s the distance (unclear).

INT1 Before the lockdown were you seeing your son quite regularly?

Res Oh yeah

INT1 Does he live local did you say, sorry?

Res Yeah. [REDACTED]

INT1 And how are you feeling about sort of after the lockdown and everything starts opening back up again and you can get out. How are you feeling about that?

Res As I say ill just (unclear). You know? I’d want my peace. I’ll still play by the rules. So I think I'll just keep my distance. Wear my mask and just try my best

INT1 Is it something you're looking forward to, to be able to get out and back to Headway’s face to face group meetings and seeing more people and your son, is that? Is it something you're ready for?

Res Yeah.

INT1 REDACTED, do you have any more questions before the scales?

INT2 Yes. Just in relation to loneliness, obviously, you said that it wasn't something that you felt really at all, [REDACTED]. If you did feel lonely? Do you think you'd be able to talk to people about that? Would you be able to seek help for that? Would you, would you feel confident enough to have a conversation with people?

Res Yeah

INT2 Do you feel you've got enough people in your life to be able to have that conversation?

Res Yeah

INT2 The only other thing was, you're obviously at the start of this as well you're very open about your attitude, you're quite jokey. You've got quite a… seems like you got quite a good mentality, quite positive attitude for life. Has that always been the case? Have you always had that mentality?

Res Yeah. Well as I say earlier, (unclear) if I come away from Headway and Ive made somebody laugh, that's made my day.

INT2 It's a good mentality to have

Res Well as I say Ive just embraced Headway. Ive taken up what's available. That's why I'm here today. I don't know Dr. REDACTED, do you know [REDACTED].

INT2 No I don't know

Res Hes in the REDACTED. He was up in Glasgow in the centre, (unclear). He does books (unclear) and he spoke to us about different thinks. His book is called the Happy Mind. (unclear) The Happy Brain (unclear). It was a wee bit too much for me, so I just bought it and he signed it for me. But I was under the impression that it was going to tell me about brain injury but it's more a dictation about his friends and things that….(unclear) to digress again (unclear) to come up and speak to them about different things. And I went once to Strathclyde University. We had found out that Strathclyde was actually doing a course on ABI's so I went up to speak to students and really a great bunch of kids. (unclear) The class were like 'who's this'. So I went up. [REDACTED], I think she's a principal, so she was sitting there and I just walked over to her, shook her hands and said, it's a pleasure to meet you. She said I hope you don't mind, and I gave her a painting I did. She said its lovely. It was just a wee thank you for inviting me up to talk to the students. And the students (unclear) I says to them, right you're all studying ABIs, tell me something. When you look at me what do you see? And a big fella stood up and said we see you have problems with your walk and you've got a walking stick. Glasses, eyesight. Theres obviously nothing wrong with your hand. I says how do you think that. He said well you just gave a beautiful painting away. I said are you getting jealous, do you want one. And he says to me oh id love one, put it up in the flat, his residence. I said well is there anything you particularly like. He said yes, I don't know if you know about it but there's a couple of them in the Isle of Skye. Musical people. Instruments. (unclear) they come in off the sea and just park up their boats, get out and over the years they've cut things out so there's like seats. And they just sit in there and "da da da". And then they start playing. I keep saying

to the boy, how do you get the grand piano in. He says, we done it in keys. I said who tuned it. He said the tuner. Oh is that the fish? So Ive sort of clicked with this young student. He’s given as good as I’m giving him. But as I say we just seemed to click. And there was a young girl there. And she seemed to be…she was hanging on every word I was saying. Digest it as if I’m some sort of intelligent guy (unclear). You’ve got one thing that’s holding you back. And she said what’s that? And I said your ideas just dip into your head but you talk yourself out of your ideas. (unclear) What you may think is a solid idea, share it with your friends or your roommates or whatever because what I’ve found out is there are people who look at things differently from you so you’ve made yourself (unclear) and then talked yourself out of it. That could be the thing that discovered (unclear) to kickstart the neurons, bounce through the brain to kickstart because (unclear) So I assist the students. I says I can see a few of your ears are starting to bleed. Is that me going on too much? I was reading [REDACTED] see I’d just like to (unclear) I would like to write a wee poem with the class. She said that would be lovely REDACTED, if you could manage. Well I have the sort of thing in my mind just now (unclear) This might be able to help the students. It went like “why, oh why, did I get my ABI. Was it because of my lifestyle or was it because my wife had died. No putting pressure on my brain where I had the bleed” Well once I got home I got it typed up and sent it to [REDACTED] and she sent me back an email saying “the wee poem was lovely. Ive put it on the students website” or something. So I’m assuming it’s a website that the students have access to. (unclear) I’ll make sure to (unclear). So she’s sent about 6 e-things from students. Just thanking me for being honest and coming out to see them. But as I say, that’s just me

INT1 I imagine they were really grateful to hear your experiences as well. It makes everything sort of fit into place and you understand things so much more when you can put it to context. So I bet they were really appreciative of you. REDACTED is there anything else before I move on to the scale?

INT2 I think the only other thing I wanted to ask was, you may have already said this. I don't know if I missed it at the beginning. There was no loss of consciousness associated with your brain injury was there you weren't in a coma at all?

Res No, nothing

INT2 Okay. That's everything from me.

Interviewer(s): **INT1**

Respondent(s): RES (P10)

INT1 **And can you just tell me about your brain injury and what happened?**

RES Okay, sure.... It was discovered in December 2000s

INT1 **Yeah.**

RES And I was getting last climb in before new year with three of my climbing friends went up to Ross and there was a good good bit of a climb the conditions were really good on the way back down the snows it started snowing, snowed over ice so the car slipped and fell into a tree bashed into a tree near Loch Lomond and (unclear 0.46) I can't remember a thing at all which is brilliant, seemingly what happened was I went to southern general No I went to ?? first of all and then say went down to erm... did south general said and then went down to Ayrshire central and then went on to (unclear 1.15) in Edinburgh and then went to day care at Ayreshire Central again so I feel like it was the third place wasn't Ayreshire Central it was crosshouse (unclear 1.32) hospitals so basically what happened was I didn't know if I was induced in a coma or I whether I actually went into a coma myself

INT1 **Right**

RES but I was in for six weeks odd and

INT1 **was that, was that sorry was that six weeks in a coma?**

RES Yeah yeah yeah. And I was my right pelvis, left side joint and right side was fractured in six places four set places two(?) upteen ribs broken and... often (unclear 2.17- 2.22) and... so when eventually came to wasn't like a (unclear 2.28) it's very hard to come to because i didn't know what was reality and what wasn't reality because I was dreaming and not dreaming because everything (unclear 2.40) routine was completely knocked off

INT1 **right.**

RES Yeah. And then obviously I couldn't walk so I I kept getting pulled back into bed kept trying to get up when I went down to Crosshouse they eventually took my (bed? unclear 3.02) away because I kept trying to get up. So I was the only person that camping in the hospital so I done that and then (unclear 3.12) easily for two months overall it was premier health care It was about six months and then went to day care for a year two years of that that's at cross err Ayreshire central

INT1 Yeah. What other so you spoke about erm obviously your your motivate like the your motor skills your you lost the ability to walk which you you've unclear 3.47) was there anything else that was effect? Cuz obviously the brain injury can affect a whole host of different aspects?

RES Yeah. It was very much my speeches away to? also I knocked off a couple of front teeth, as well.

INT1 Right.

RES So I had a wee bracing, which is a wee brace, it was a huge big bracing, which very slurry, I thought it was due to that. But seeing, seeing the reactions feedback from people when I talked. I was getting that immediate reaction back seeing like they didn't understand what I was saying . So I find that really difficult to come by. But the one thing I remember being in hospital it was like, doing it for role as watching television being a patient. And that that's it that that helped a lot. Yeah. Just getting through it all Yeah. And so the thing was, we're going to (unclear 4.55) then started learning about obviously I couldn't wait to learn more about physiology developments?

INT1 Yeah.

RES And things like OT, basically well, I kind of had and like a stroke, which I left handed anyway so it was very difficult to write with. Pause speaking, it was dysarthria dysarthria is it called

INT1 yeah,

RES (unclear 5.28) general. And so as far as communication was going, it's like me speaking and not being able to write and stuff. and also my minds going quicker than what I could actually physically do. Plus, my memory is, is failing.

INT1 Yeah.

RES So as far as good for anythings my mind was jumping up. Because my brain was slower. It could lost. So what, when senses that I see for different subjects (unclear 6.04) it was really hard going?

INT1 Yeah. With erm. Obviously, you moved around quite a few places.

RES Yeah.

INT1 Did you? And I assume you had sort of different aspects of rehabilitation in those different places. Can you tell me about I guess, obviously, now, that's, that's had an impact. And because of the kind of career and self employment route you've gone down? What What would you say were the, you know, the things that helped you in your rehabilitation? And what would you say are the things that maybe hindered you or you found hard in your rehabilitation?

RES I found hard was learning speaking, I said to you last week, about I can remember going to the speech therapist. And it was getting really, really like exercise you expect of a wee, wee child do to. And I've struggled it then so I had to build them, build them, recorrect what I was saying? That took a long time, because obviously with different formats, like yeah, and your way thinking can be different. (unclear 7.14) different too. Apply the same action but said differently. That was difficult to go by and the best thing I loved. I really enjoyed doing physical activity.

INT1 Yeah,

RES The do the thing physical therapist (unclear 07.40) and stuff I love that. This (unclear 7.46) is something I knew before. And listen, I can I can make a result with. And those with psychology I knew (unclear 7.58) before that point. Do you know what NLP at all?

INT1 Neuro Linguistic processing?

RES Yeah. Yeah, yeah.

INT1 Yeah. I'm familiar with it. Yeah.

RES Oh sure. I used it a lot when I was training. as a as a trainer, I teach to train, trainers, to help them organise their classes better. So I favoured that too, that helped.

INT1 Did you use that that because obviously, those NLP processes are about the I guess the power of words to some extent. So did you use that for yourself in order to motivate yourself in rehabilitation?

RES Absolutely. It's using err skills and tools to do that.

INT1 Yeah,

RES that helped me greatly it show me messaged to help control being annoyed and frustrated. It is a pattern to interrupt.

INT1 Yeah.

RES That helped immensely (unclear 8.57) between that and thats willing to help, and that's what I'm doing the groups I want to show my pattern interrupts too

INT1 Yeah, just out of interest, the groups that you sort of work with now, are they also individuals who've had a brain injury or these erm?

RES Yes, yes. I'm sorry I didn’t explain that very well, It's like it was actually with Headway in Glasgow.

INT1 Right.

RES It made me stop and want to do full Scotland with it two?

INT1 Interesting, I think you must find that extremely rewarding as well in terms of being able to help people who've gone through a similar experience the …

RES Absolutely, yeah. I find that very, very rewarding

INT1 **It must be very rewarding, as it must be rewarding for them as well. Being able to see obviously, what they could potentially achieve or what they can, where they can get to based on your rehabilitation.**

RES Yeah, it's because it's like someone's walked to talk. And basically it's like same thing I done, (unclear 10.05) teaching is when you screen someone and get a wow. A positive. That's what I go for. That's why I do what I do. Seeing that experience that wow factor from people

INT1 **yeah,**

RES even when don't see it i you just know also as well, After my wee car bump, is in my senses, I can pick up far more, more now than I could before,

INT1 **In what way?**

RES I can pick up on people, I get more information from people I've spoken, thats it I can learn more, I've learned more from NLP and stuff, also psychology and stuff by what folks see and how they see it with a consensus of special (unclear 11.02) therapists, as well as I can I can sense where someones pains come from, some reason I think I've acquire the skill of doing that

INT1 **It's more a.., is kind of a better understanding of human behaviour, I guess, to some extent.**

RES Yes, that's definitely part of the NLP but it's more sort of I can just see it in. I can pick up a sense from someone who is coming from your forearm the pain. Yeah. Well, that's (unclear 11.39) I can tell by feeling whats in my hand.

INT1 **Right, interesting. (cough) excuse me sorry. Em.. So I just said, obviously, that you spoke a bit about your brain injury in relation. One thing I want to talk about a bit more is the lockdown and the pandemic more generally. Erm so over the past sort of 14 months, 12 to 14 months or so effectively.. errr. Ever since we've been in erm sorry, one second. Okay, yeah. Yeah. Can you still see me? Can you see it?**

RES So just trying to I can hear you well? I'll just leave it alone.

INT1 **Erm so yeah, so yeah, so long as you can still hear me that's absolutely fine. Yeah. And you're not missing anything, I promise (laugh). So in relation to the sort of pandemic in the**

past 12 to 14 months or so. Can you tell me about the people that you you've seen you've interacted when you've interacted with sorry, how has how has this time been different for you? In terms of you know, your your support network or your social network?

RES listen, to be quite honest. It was the same

INT1 Yeah.

RES See, same with lock down it was same old to me. And there's limitations of what I could do. But I still felt isolation within the community beforehand and even now it's just more so, I was constantly ignored by people

INT1 constantly ignored by people sorry did you say?

RES Constantly ignored, constantly knew that. what is like. what I was experiencing so last thing I want to do is help life experiences all the time (laughing) I didn't see that but erm inside that is what I was experiencing.

INT1 Yeah

RES And just spoke will give them melodramatic "Oh, this is all terrible (unclear 13.54)" Well, welcome to my world (laugh)

INT1 Yeah, yeah. Have you seen anyone like it? Have you? What's your what's your your social network like generally prior to the pandemic then is it is it quite similar? Or like So? So for example, obviously you got your your partner who I assume you spend quite a lot of time with. Is there anyone else that you interact with socially or sorry, prior to the pandemic was anyone you interact with socially

RES Of course of course. See before my wee car bump it was I had a very big social life

INT1 Yeah,

RES (Unclear 14.34) wish all on get on well with people stuff and then basically what happened was there was a reduction there was (unclear 14.42) general friends, there was true friends. So there was a reduction big time reduction still go out and about but it was reduced so

spend a lot of time by myself, rather than have to explain the differences or slow down etc is better to just other times go by myself. For example I could get up the hills, I used to go way up the hills, drive up north, sleep in the car (unclear 15.12) cos I was slower at doing it and climbing all in one day can be very tiring. So I'll go up there and sleep over something it depends on the person, where we are going, it's totally feasible then them. But I gained a lot of friends like see the massive swimming. I mean, I'm also a massive swimmer. That's where I was this morning at six o'clock this morning.

INT1 Wow fair play

RES I was up at five to six to get to the pool. So did over a mile, so good. But because of that I've gained a good network of friends I wouldn't (unclear 15.58). A couple friends I stayed close with, and one of the people I used to go uphill's with because he's retired too, he's got more time. So he enjoys the company too.

INT1 Yeah.

RES it's a hand in hand situation.

INT1 And have you been able to, so obviously, there's there's obviously that divide between your friendship groups prior to the brain injury. Then you've had your friendship groups post brain injury where you've spoke about this divide between real, you know, true friends and friends who then potentially fell by the wayside. And yeah, less interaction with because of changes in circumstances over this past week. Yeah, yeah. Yeah, completely get that over this past sort of 14 month 12 to 14 month period and the pandemic, then how has you like any, have you been able to see or not see as such? Because obviously, we haven't been able to meet up with many people. But have you managed to keep in touch with these friends that you. Because obviously, you used to do quite a lot of exercise with these people as well. I see your work now that things are opening up again, you're able to do that a bit more? Have you still been able to interact with this group of friends over the past 12 to 14 months?

RES Seeing in compassion to prior lockdown. It's a, I actually have more of a structured week?

INT1 Yeah.

RES And I was seeing more people (unclear 17.34) that sounds like a lot. But like, I could do regular visits once, maybe twice a week with someone. I also have time with partner with her working as a nurse she she erratic shifts and stuff.

INT1 Yeah I bet, yeah

RES So it's like that's that it was a big help. We used to go a couple times a week, walk up the hills for example or even, go with my sons and just been walk the dog and thing. And then moves like really good, Because with lockdown people more family orientated where's before that was getting a bit missed.

INT1 Yeah.

RES So it's more prioritising the new bubble. Family bubble. Yes. So

INT1 Has it strengthens, so in that respect, then do you feel is strengthened your relationship with your partner?

RES Yes, I agree with that yes. I will say that also. Personally, I'd say Yes. In general, say sometimes have so much time with someone that become the default. (Unclear 18.50)

INT1 I understand. Yeah, yeah. And you obviously, you obviously do a lot of activities actually generally then like in terms of your you know, your you're not not content with just a single job. You effectively do two jobs as well in terms of the the self employment also. And you also do it's sounds like you're quite a fit individual, you do quite a lot of exercise activities with hiking and things like that post brain injury. How has your level of activity changed, I guess, since the pandemic

RES During, that's a good question that I think the one ethos I had, that's because I was very, very busy beforehand, and I've always done lots of different types of work. And she seems employed I've have got one two, three, maybe four different types of work within it. I always keep busy. as well ethos always had was keeping busy, active and going out all the time cos there's nothing worse than spending time with yourself totally deflates things. So I had to keep busy as well. Plus also, one thing erm you should always do was to help improve my running example, I used to go running a lot, I used to go daily. I was about 30 miles week, 40 miles a week.

INT1 Christ yeah, good distances then

RES it was a good, good while, and there's a 10 mile run on Saturday, whatever, 12 miles went to half marathon. But then it's like, basically what it is, is keeping busy. It's the things by do things, your mind still (unclear 20.51) little time, Very good for your mind, but also productivity. And instead of all your eggs in one basket is much bigger now so.

INT1 So in terms of So You said that? And is that idea of spending time with yourself? You find quite deflating, like, is that something you've experienced more I assume? Well. So what I'm trying to do here is tease apart the brain, what post brain injury and I guess during lockdown is two separate time periods, if that makes sense. So do you find spending time with yourself has been problematic during the past 12-14 months. And has it been more problematic than prior to lock down? If that makes sense?

RES I understand what your saying. I see the difference being is like pre locked down and post lockdown and stuff. actually lockdown because of limitations of what you could do. I did feel difficult being myself. At times doing things. Because there wasn't that many things to do?

INT1 Yeah.

RES It's like one thing I've done, I've done umpteen other courses. like cooking. Yeah.

INT1 So you're someone who actually who constantly needs that stimulation then?

RES Absolutely. Yeah. Yeah. Yeah. That's a good, it's a good synopsis yeah.

INT1 Okay, that's interesting, in terms of Okay, so one thing, obviously, that this project is kind of geared towards, and what we just spoke about, that leads me quite nicely into is trying to explore the concept and the idea of loneliness. And so one thing that we've been asking people is, is how would you define the term loneliness? What does that term mean to you?

RES Sure. That's a good, I've got a bit of a contradiction there, hope you don't mind, it's up to you Because it's like, I'm happy with more company. Yes. I like to keep busy, I really like to keep myself stimulated with things, keep going. Whether it's running. The one thing about running for example is that it helps have (unclear 23.10) the world my mind (laughs) thing even is cycling and same thing it's different aspects, also, swimming I keep swimming four times a week there. And also I swim train, three times a week, three times, four times a week there. So it's like is also thinking of what i'm doing for that, keeps me going. I thought, that's a good point. Because this is like (unclear 23.52) thing of of having to keep busy, and stuff

INT1 I don’t want you to overthink it. That's the thing. So obviously, that there is that quite solo sports as well. So things like cycling and running, swimming.

RES Yeah your right yeah

INT1 Things you can obviously do on your own, like I do the same thing. So for me runing running. I can run with other people when I train with a running partner sometimes. Yeah, that's quite nice, because you can go for a run and you can have a chat and yeah, it's actually quite a nice experience. The run becomes secondary, I guess, to having a conversation. Whereas it's a very different experience when I run on my own because then I'm in my own head and I'm like you say, figuring things out all the sports that you've just described there. I can understand because they are quite solo ventures. So yeah, the thing is like the running cycling and the swimming are obviously releases the essentially releases for you.

RES Yeah, that was releases yep I would definitely comment releases. I think the second to complement thinking, just think I'm improving, how to better, how to better it Yeah,

INT1 That's interesting. So just going back to the term loneliness then so how would you define the term loneliness? What does the term loneliness mean?

RES Well, obviously loneliness mean being alone, not having interaction or my case stimulation. Something missing there, missing life.

INT1 Interesting. So, so in relation to so that meaning to you, do you. So you said that not having that stimulation in relation to you. So do you, is that something? Is that a feeling that you've experienced? Would you say that you've experienced loneliness particularly over the last year for example.

RES Umm, I would, (sigh) there's nothing comes to mind directly to say I feel like lonely because I always keep myself busy. Again, it's because I go to certain talent of keeping busy but that doesn't happen, but of course, in one sense you get and one sense you take so with having such a prime aspect of keeping busy that takes away things. So It can be, I get the effects indirectly.

INT1 Interesting. Did you do you feel that like the because Because obviously, right, you said you're you're quite busy, and you do these quite, quite exercising produce activities as

well? Do you find that? Would, would it be? Would I be correct in saying that these are potential coping strategies for alleviating or stopping those stopping those things of loneliness? potentially?

RES Absolutely. Yeah. Definitely? Bingo. Yeah. I think particularly as (unclear 27.04) keeping going, is one aspect of it. I wouldn't say it Initially, I wouldn't see it’s feeling the loneliness, but it is important part of it

INT1 Interesting yeah, it's it's great. Erm One question around that if it's, for example, if you did feel lonely? Do you think that you would be able to talk to people who you have currently in your life about that experience? About You know, you were feeling this way?

RES Um exception to the rule over this? Because it's like, once upon a time I wrote a book about myself. And it is, what, what what a friend would see me here and that's helped me a lot different difficulties is spoken, understanding to that. rather than me tell me more aspects. An (unclear23.27) another way,

INT1 Right Ok

RES So that helped pass the information of what I was experiencing of dealing for example, with loss in my life Yeah. I had a major major loss in my life like, I lost my work, then I lost my wife, I had two kids they went away, lost my house, lost my car. Everything was back to beginning again. So... It was said, How did I cope with that

INT1 yeah.

RES So simple coping has become like a way of being with me. If I'm gonna deal with emotions from it, just feel ok that sad deal with it.

INT1 You've obviously got quite a lot of resilience.

RES Yeah, yeah. I would say so yeah

INT1 In relation to Okay, so just think about lockdown now, specifically, what would you say has been the most helpful thing that's got you through the past? I know. But to be fair, you've already said this is taking into account obviously, you really said that. Nothing really massively changed you in this period of time. But if we think about lockdown,

specifically the past 12 to 14 months or so, what would you say has been the best thing about that period of time or what's helped you the most through that period of time?

RES Well, the one thing I see is not going to work. (unclear 29.50) my identity I felt a big loss at that because the big question mark about what's going to happen? Am I going to lose my job? I lost as well, first of all, the first one, then see what my (unclear 30.06) employed instead of being self employed, I was re-employed in the company that's still into process, but what helped me go through it all was erm doing courses, give me structure to go back also give a targets to go for, had to pace myself, pace myself give me a focus to go for. So that helped me big time. came through lockdown. Yes.

INT1 What did you assume the inverse of that then what was the most difficult thing? I assume? Probably what you've already explained there in terms of is there potentially something else in relation to that, you know, the lockdown period? What was it that you found most difficult? Was it that loss of identity because of the job insecurity aspects or, or is there something else as well?

RES I would say probably starting loss of identify, It was something I felt I'm really really (unclear 31.13) to get up there and get this back in my life, then been lost. It's like being kicked when you fall down. And that sort of, that was a big thing for me was loss of identity and not knowing how to do it, I tried different aspects of getting to into other aspects of work that wasn't successful at all

INT1 Yeah The final thing that I wanted to ask because obviously, we're now moving out of this, this sort of post apocalyptic life that we've been living for the past 14 months or so. And societies starting to start well starting to move again, really, with the opening of you know, shops and pubs and etc. How do you feel about life post lockdown? How do you feel about re entering society again or getting back to civil society getting back to some level of normality?

RES That's good question. What I feel is like we haven't been (unclear 32.25) do you understand and then you go back to normal, but I’m still in the same boat and I'm going back to a place where I feel different I feel alone. Because today, even describing folk that I find difficult, because even with the fine folk, (unclear 32.49) it's like I'll give you example on the train today, early morning Thursday mornings dead early and cognitively it's very hard to get in to go fast. Too fast to process information was supposed to drill, supposed to do be doing and you can tell before obviously early morning they alert when I ask what's this, whats this, I'll go back home keep asking with this. I see him go okay. And it's very much like a like some times like when It's an effort for them or gives the impression talking to a child. So that (unclear 33.37), that's one of the reasons why I don't make big deal about head injuries to folk I rarely say it.

INT1 Yeah.

RES And I was quite good before it was (unclear 33.50) is when I was quite prominent and before the accident I had foreign friends. That was my cousin who got in touch with a girl a German girl who worked at IBM (unclear 34.03) and through her I got a little hope (unclear 34.10) foreign friends. So that was really good. We all had a connection was detailed (because they felt a bit out of it and so did I? 34.27) it's was team work

INT1 Yeah,

RES and it was good.

Interviewer(s): ***INT1***
INT2

Respondent(s): RES (P11)

INT1

And If I can get you to tell me a little bit about your brain injury if that's okay.

RES

Yeah. I contracted viral encephalitis in September 2019. Well, you know, it just started like a flu like illness. I was, I was in London, I was in REDACTED their service area. I was in a Wetherpoons having my dinner with some colleagues before going to the hotel next door, I was staying at that hotel with work, like for the best part of two weeks. And, you know, I started to get flu symptoms, fever, high fever and really feeling poorly, feeling odd you know. Just like you know, combine that like a flu, you know, lemisps and hot lemon and just cough remedies from the service station and I thought, you know, give it a day or two, probably three days and I was in the hotel. Anyway, I got a phone call from the work saying I had some work scheduled for the Friday, Saturday, and Sunday. You know, be prepared for that work. By that point I was fevering so I kind of said Right, okay. I got 45 minutes later a call back and said now I'm feeling poorly, I'm feeling unwell. I think it would be wise to ask someone else to do the job. And you know, give us a few days to rest and get over this bug. That wasn't good enough. So, I ended up I got the sack. And so, they said to me, Well, you've got the rooms booked until Monday. So, you've gotten to Monday to either get better or go home. So, I got left home to Glasgow, my parents' house, stayed in bed for seven days and went to a GP surgery. At that point I started getting these shakes and tremors. The Monday, I was still getting the shakes in fairness. I had been to out of hours NHS over the weekend. I'd also been to Casualty on the Sunday. Presented on the Monday shaking in front of the practice nurse, in front of the GP. And on the Friday the practice nurse had prescribed me penicillin when it's marked on my documents that I'm allergic to penicillin. I was issued other meds on the Saturday, you know over the weekend these tremors and shakes are getting worse and longer. Non controllable, you know, and like blinding headache. I was I was very surprised when I went to Out of Hours with my father after experiencing these severe headaches that they sent me home you know, I was very surprised that the severity of these headaches was…I thought I should really go to the hospital you know, I thought there was something seriously wrong but later turned out to be viral encephalitis. Initially they thought I might have had meningitis or sepsis, but it was really intense pain in my head. It was like a pane of glass freezing up you know, pulling over your head and just… tense

INT1

And so, what about sort of life after that, then were you offered any? Did you need any rehabilitation? Or were you in hospital for a while or…

RES

After that I was, I was seven days in the hospital. I wanted out, I think I was going to 8 days in the hospital, but I wanted out on the seventh, because it was my son's birthday on the day I got discharged, the day before I think, I got out the hospital. And I just kind of, I get freaked out, there was an old guy. There was four of us in the room. And there was an old guy who's dying. And the guy opposite me was given a cancer diagnosis like, you know, he was given cancer, cancer of the penis, you know, he was like, I heard him being told, oh my god, I felt so bad for the guy that it was, he was going to die. And the way he was going to die, he was just, you know, I was freaked out. This old boys giving his death throws and I just wanted out that day. But I never got any rehab. The only rehab I found was via the job centre, the local football club, Celtic Football Club, the foundation were doing a training course for 10 weeks, like to improve fitness and, and employability. So, I went along, and it was metafit training they were doing and it was quite intense. For myself, I was uncoordinated. My limb movements were delayed, my right-hand side was you know, weaker than it used to be. My dominant side was like disabled. You know retarded kind of thing, but I found that it kind of stimulated me, helped my recovery. My coordination started to improve. Sadly, the, the pandemic, meant that we only get eight weeks into the course instead of 10. So, like, all that all that encouragement just stopped suddenly and then I had I just moved into this flat as well. I had previously been living with my parents. And you know, I had been working before as a coach driver. So, I was on tour, I was busy, different hotel every night. The Hilton at London Heathrow Airport and Saturday night I'd be out in like Cambridge or something on the Sunday. And then up to Edinburgh and Glasgow the highlands over Ireland, you know, I was busy with Chinese people. Mainly Japanese and Chinese market. I don’t know if i suspect maybe COVID, that looking back I actually think I had COVID back in, or a form of that that led onto this flu that led onto encephalitis. The doctor don’t really know how I got it. The only people I was mixing with were passengers or hotel staff and you know it was kind of a bubble I was in. And like, I was dating a girl at the time who's a pharmacist. She worked in the hospital in the pharmacy. And so, I don't know if there's been some bug I picked up.

INT2

Yeah, it's quite possible. And other your weakness to your right-hand side has it left you with any other impairments at all?

RES

Yeah, well, I had a lot of trouble sleeping. Like it's improving recently. Anytime I would be going to bed id be having blackout blinds, rooms totally dark. But when I shut my eyes I’m in a bright room you know. I have great difficulty sleeping. Lately my sleeps improved because I've been a having the odd joint. So that has actually improved my… I'll be honest, I gave up smoking weed for about two years. Since this happened to me, I was tee total, I had stopped drinking alcohol. Stopped smoking weed. Only in the last sort of month, six weeks, started smoking weed. And my sleep is so much better. Ive actually stopped taking my antidepressants, I feel better. Sleep better. And that's a good thing.

INT2

Was that something you chose to do yourself taking? Getting rid of the antidepressants? And you think…?

RES

Yeah, I was keen... I found antidepressants were making me really sleepy and lethargic, like a zombie. It wasn't enabling me to do anything. It was making me drowsy during the day. But it wasn't enabling me to sleep at night. You know, so that was a negative. When I stopped antidepressants, I found I had more energy for the day. Plus, I'm sleeping at night. So, it’s a win-win situation

INT1

just to check with the depression diagnosis is that since the encephalitis or was that prior?

RES

Well, I did have it prior, but I was stable for a long, long time. After encephalitis I was really depressed because I had lost my driving licence. And, and, you know, my income and all that. The loss of independence, you know, was a big depression thing for me. Coming up to next month, one of my friends committed suicide, you know, he was in a similar position to me, he was a, he was given a possible cancer diagnosis. And he was a coach driver. He was actually a doctor of toxicology, He used to poison fish. You know, that's how he got his degree. And he hung himself when he was faced with being made unemployed as a result of the pandemic. The team I was working with all got paid off before the furlough scheme was announced. So, I don't have a job to go back to. If I get my driving licence restored. I don't have a job to return to. The company is no longer trading. And because it’s a commercial licence, like I can apply for my car licence, and then I would need to undergo a medical to, you know, gain my coach licence, my bus licence again. I think I can do it but I don’t know. I think my fatigue would be an issue, that would, I think that’s what would hold me back. I think I could drive but I don’t know I would need to try it.

INT2

Yeah. Just while we stick with the pandemic can you tell me about people that have supported you that you've seen on a regular basis that you've been in contact with?

RES

Yeah, well, I've been involved with local headway group, Headway Glasgow. They've been great. They have a meeting five days a week. You know, for between an hour and two hours every single week. And that was that was great for a bit of social interaction. A lot was virtual via zoom. I found it great because it meant I wasn’t sitting…I wasn’t meeting anyone, but I was talking to people virtually and it was all people with like head injuries and brain injuries. So that was, I found it. That was rehabilitation in itself. My talking kind of improved a bit. Yeah, I found it real good. That’s about all, I think. I've spoken to the members of Headway Glasgow more than I have my family.

INT2

What about before the pandemic, obviously you started the metafit. What kinds of other social engagements were you doing?

RES

That was it really, I was I was, well, I was back. I was obviously doing before the pandemic, I was always shocked when I was driving the coaches. I would be in Heathrow Airport on a Saturday morning with a departing group of Chinese tourists. And then in the afternoon, I would be at Heathrow airport arrivals to collect another group. Then take them up to a hotel in London. Give them a bit of rest, cuty tour of London the next day. Out to Stonehenge, you know, just start a UK tour for either a week or two weeks duration. That kept me really busy. I would tend to do that, you know, every week, every two weeks was the same. You know, the same route. Just different people, different tour guides. Sometimes the same tour guide.

INT2

Did you find that kept you very busy? You didn't have a lot of time for the things

RES

It did keep me quite busy. I would get some time off when I got to the hotels. But when I came up to Scotland when I came to either Edinburgh or Glasgow, that's when I got my time off. Another driver would come up and meet me and they would do the Scottish part of the tour and I could come home. At the time I had recently split up with my fiancé. I had been engaged to be married but we split up. So, I was on tour and I started dating this pharmacy technician at the hospital, I was dating her for a while and screwed it up. I was with her the day I was meant to get married, and I said…ah I don't know.

INT2

Did you mention you've got a son as well? Do you get to see him much?

RES

Yeah, I see my son regularly. He's doing his 4th year exams at school just now.

INT2

Obviously, before the pandemic, you were very busy with your job and probably took up most of your time. How have you been filling your time whilst we've been in lockdown?

RES

I don’t know. Ive been carving a groove in this armchair. I've really done nothing. I’m planning on doing a West Highland Way walk next month but apart from that I’m hardly out of the house, you know, don't really see anyone besides my neighbours and the regular dog walkers. It’s really, really quiet round here. I think this is what it feels like to be retired like a pensioner you know?

INT2

Do you like it? Or do you prefer to be busy?

RES

It’s too quiet. I miss my independence. I miss being able to drive. I miss being able to just jump in the car and go anywhere I want. You know, it's cold out there trying to get public transport in Scotland. It's a wet day, it’s raining…

INT2

It's not just Scotland. North of England is just as bad at the moment. Can't remember the last time we saw the sunshine.

RES

Where are you’s from is it Northumberland?

INT2

Yeah, well, I'm Newcastle, Chester-le-Street

RES

Yeah. I mean, I used to be I think last time I was in Newcastle I brought a coach load of Chinese people to to a pub. Trying to remember the name it. They all stayed at Jurys down by the Quayside. Last time I was there it was fun. Newcastle is good. Got a lot of fond memories of Newcastle.

INT2

Yeah, yeah. It's a lot of people would say that. It's one of those places. I think people come for a good time.

RES

Chester-le-Street yeah?

INT 2

Yeah. That's me. So, there would have been a little bit of time, after you were in hospital before the lockdown happened. How were you finding that time readjusting?

RES

I found that difficult because I was back. I had nowhere to stay really. Whenever myself and my ex-fiancé split up I don't really have anywhere to stay. I was I was kind of hot bunking you know, I was using my parents addresses, as you know, somewhere to stay. But I was always away on tour, I had been working all over Europe. So, I was all over Spain, I was all over France, and all UK and Ireland with work. So, I was really really busy. I didn't have to socialise. Which I found out whenever I went to hospital. The only person that visited was my father. Like my friends and all that, you know, I speak to them on Facebook, but I wouldn't see them in person. That's still true to this day, it's very very…very few people around. If I see them, it's just pure random luck

INT2

And how does that make you feel?

RES

It does make me realise just that we are all quite vulnerable and alone and its every man for themself.

INT2

What about the term loneliness? How would you describe that?

RES

It's, I don't know. You learn to be at one with yourself. Ive found Ive become a bit more zen

INT2

Do you think that you experience loneliness? Do you feel Do you feel lonely at times?

RES

Yeah, yeah. Yeah, I do you feel lonely at times

INT2

And this is something that you feel you could talk about with somebody that perhaps your dad or…

RES

Oh yeah, I mean, I've been trying to do something about that you know. Ive been on the old tinder a bit more. I'm not as lonely as I used to be, put it that way (laughter)

INT2

Oh good (laughter). Do you think either lockdown or your illness, do you think that's made you change how you thought of loneliness? Or how you what you thought loneliness was?

RES

Well, I don't know, I always kind of function well alone, even you know, in relationships I've always been quite independent and, and, and I've had a lot of time alone through work placements, you know, that would take me away from home, Ive always been quite happy with that. Loneliness has always been there but I've I've been distracted by other, you know, other engagements at the time.

INT2

Yeah, I can, I can get that. And what about things that have helped you through lockdown? Is there anything in particular that stands out?

RES

I don't know. Internet TV. And, you know, access to the information on the internet. It's amazing just how you can immerse yourself in the internet these days, you know, without leaving your home you can like, order your food, order your groceries and you can just immerse yourself in films and movies and it's great, it really is. Fantastic

INT2

And is there anything that's been particularly difficult for you to think?

RES

For me, again, meeting people and sort of just having self confidence in yourself. I find that difficult. But I don't know, Ive been reading a few of these guru books, and that's kind of changed my attitude and changed my mindset as well.

INT2

The lack of self-confidence, is that because you're interacting with new people that you've never met before, sort of through headway?

RES

Well, I’m not really meeting new people through headway. Theres not a lot of active members. It was strange to meet them at first. You know, that was I was challenge. I think when lockdown happened, they just started to put these Zoom meetings together. And I had great difficulty trying to access Zoom through my phone. And so, after a wee while, Headway actually offered me a tablet, you know, which helped me get online. And that improved my isolation because could then take part in zoom meetings on a daily basis which helped a lot for me. That was a lifelines for me

INT1

Obviously with Headway you use Zoom for the video calls. Did you use Zoom to stay in touch with anyone else?

RES

Well, since then we've had some family Zoom quiz nights and that's involved family from America, Phoenix and Arizona and Ireland and Scotland. And that's a regular…like we were doing them once a week, we were doing them over the Christmas and New Year time we started to do them. I've not done them lately I must admit. I had COVID which, so I was meant to do the quiz one night and had to pull out so not done it since

INT2

In the respect, though, that you've been able to reach out to sort of family in America and things. Do you think that’s brought you closer that perhaps wouldn't have happened had we not been in this situation?

RES

Oh, yeah, it's good. I mean, especially talking to my cousin in America it’s great. He got married a few years ago and him and his wife they just had the first daughter about two months ago, three months ago. So, to experience that you know, via zoom and video calls was excellent. You know. That was something we never had before. These video calls are brilliant

INT2

Do you think that's something you'll continue to do after lockdown?

RES

Yeah, definitely. We'll definitely keep in touch more. It's, I think they've not as much time recently because obviously the babies demanding being a new-born. There won't be much sleep getting down in that house. But yes, it's good, I'll definitely be using it.

INT2

And how are you feeling about sort of life after lockdown and society opening back up again?

RES

It's interesting. I'm interested to see what's out there. Go and explore. And just, I'm nervous at the same time, I'm kind of…I'm limited by public transport, my options around here. You know, I have to think do I have money in my pocket to travel and stuff? That for me during lockdown has been difficult you know, you know, budgeting has been hard. And motivation, motivation as well. But definitely isolations…it would be good to get, I think, motivation improves when you've got someone to, you know, encourage you from a friendship point of view you know. I need to work on that

INT2

Yeah. Do you think when the headway group started to open up face to face again? Or is it still just virtual for the time being?

RES

They've were starting to, they've proposed to meet up in Glasgow, the walking group was going ahead, and then well, Glasgow is still on Level two restrictions, or level 4 restrictions I think it is. But they're hoping to bring that down next week. So, I don't know. I was going to Glasgow, being a rebel. Ive had my virus and the vaccine so I was just going to go if I want to

INT2

Yes, so you'll…you're not sort of concerned. You'll be out and about as soon as soon as you can?

RES

It's just a virus you know, we've got Hadrian's Wall, the romans were scared of the Scots, were made of tough stuff

INT2

REDACTED, do you have any more questions before I move on to the scales?

INT 1

No no, nothing more for me.

Interviewer(s): ***INT1***
INT2

Respondent(s): RES (P12)

INT2

Then, if you don't mind, would you be able to tell us a little bit about your brain injury?

RES

About my brain injury?

INT2

Yes, please.

RES

Right. It was in August 2017. I was mountain biking in the lakes with my wife, where for some reason I came off my bike and collided with a minibus coming the other way. So, the air ambulance was called obviously, I was put in an induced coma, flown to Preston. Suffice to say, I've got 8 weeks where I've got post traumatic amnesia. So, I can't remember a thing for eight weeks after the accident. I'm just going by what people have told me. I was in a coma for 10 days. I was at Preston for five weeks, until I was able to be transferred to James Cook Hospital in Middlesbrough. In total I was in hospital for three months. The injuries I had was a bleed on the brain, a bleed around the brain, a fractured skull, fractured eye socket, and other minor injuries.

INT1

Sorry, REDACTED, just before we keep going. The connections a bit strange. Is it? Is it just me or is anyone else's camera flipping in and out?

INT 2

My cameras okay

INT1

Okay, I'm just going to quickly leave the interview and come back. I'm just going to reset my internet because I think my internet is a bit dodgy So okay, I'll leave and come back and join in very quickly. Just for like the next five minutes or so

INT2

I didn’t get that. Oh, he's gone. I think he said to carry on. Did you get that?

RES

Yeah, yeah.

INT2

So, can I just check it to you were you were in a coma for 10 days but in the hospital did you say for eight weeks?

RES

No, I was over was flown to Preston in intensive care, I was there for 10 days in a coma and there for five weeks until I've transferred to James Cook and I was in hospital for a total of three months.

INT2

And what kind of rehabilitation did you undergo at that time?

RES

Well, when I was in hospital?

RES

Yeah, or after?

RES

Well, that’s hard to explain really. I mean James Cook were excellent. I can't remember what… you see my short-term memory is bad. I think they were called… I can't remember the name of the department they were from, but they would like walk around the car park, have talks with them and just explaining stuff. It was therapy but I can’t remember what it was called.

INT1

That’s okay. Did you find it helped?

RES

Oh a great deal? Yeah. I started going to headway. Mainly because the headway nurse at James cook had a big impact and big involvement and told me about it. So, we went and then I’m now one of the trustees of Headway Hartlepool.

INT2

Fantastic. So how much of your daily life was affected at Brain Injury?

RES

Well, it was a lot because... sorry Im just playing with the dog. My executive functions, I think that’s what you call it. So, I find it hard to follow instructions and like follow menus or recipes and stuff. And fatigue is a big thing as well, I get really tired really easily, which takes a lot of getting used to, but you soon learn that you listen to what your body's telling you, if you're tired have a sit down. Have a rest.

INT2

I forgot to mention sorry. Obviously, if you get a bit tired during the interview, just let us know and we can take a break or we can come back to it if you need to be so we'll just go at your pace so just let us know

RES

I get easily confused as well.

INT2

No worries that fine. So, did you have, the rehabilitation that you had afterwards…did you do any…Did you say you had your balance, or anything affected after the injury?

RES

Yeah, I've got right side weakness as well. So, my right side isn’t as strong as it was. I did get physio when I was in hospital I enjoyed. But it's just learning the limits of what your body's telling you. Just take your time and slowly. It's not like a big step. It's small steps. Ive learned that as well. I mean, being with Headway we used to meet once or twice a month. But when we do meet later on in the afternoon, the chairman or whatever, the secretary will actually go back to James Coo, and we

would go on the ward that I was on to sit down have a little chat to patients who are in the ward and tell them and their families what happened to me and that there is light at the end of the tunnel sort of thing so I enjoyed that.

INT2

That must be really rewarding sharing your story

RES

Yeah. At the start I was really wary of going back. Because there had been a few months, six months or so since I'd been on the ward. And I was wary that none of the staff would recognise me or remember who I was, but they all were like “Hello!” and it put me at ease. And it was nice talking to families and telling them this is what happened, and this is what you do and take small steps. So, the sort of passing on what I've gone through to help them and I found that really helpful

INT2

Yeah, did you find that when you were in the hospital, did you find talking to other people with brain injury and getting that support and hearing how they've recovered that helped you?

RES

Yeah, there wasn't really many people because the patients in the ward with me were at different stages. So, some were okay and some were really quite but bad

INT2

If we can just think about the last 12 to 14 months whilst we've been in lockdown. Can you tell me a little bit about sort of the people that have helped you and how your social engagements have been how that's been different to before lockdown?

RES

Yeah. Mainly I’ve been in the house the majority of the time. I didn’t want to go and mix with people. I was like that before Covid. But I got a lot of support from my wife and my family. So just talk things out. Do things together, keep busy and occupied. And any worries I had I would tell them, especially my wife. I would say how I was feeling, and she would really help me.

INT2

Brilliant and before lockdown happened so sort of that space in between your brain injury and lockdown happening, were you having a lot of engagement with anybody else sort of friends or sort of a support network outside of the family.

RES

Yeah, there's one main friend I had, who Ive still got actually, I used to work with him. Because, when, I'm trying to explain so you understand…when I was working, I was I was actually a detective. So, I was going from being full on, 24/7, going to work not knowing when you'd finish because all the jobs that would come in. I used to work in Washington, Sunderland, Newcastle, anywhere. I lived at least half an hour from where I used to work so a lot of the people, I worked with don't live near me. So, there's nobody really over this way but this one friend is the closest and we kept in touch and go out and meet up and walk the dogs and have a good chat and get things off our chests and have a good chat. I really enjoyed that but then lockdown hit and that was knocked on the head for a while. If that makes any sense

INT2

Definitely yeah. So, would you say that other than that the friend that used to work with, your social engagements have pretty much stayed the same through lockdown, it was mainly sort of your wife and your family that wasn't a big difference?

RES

I've managed to volunteer. It's just a local small like Country Park. So, I volunteer there once per week only for a few hours. And we go and go around picking litter up, repair fencing and stuff like that. So, it's just I really enjoy it because it's nice to get out and get some fresh air and do something worthwhile. And then lockdown hit so it was knocked on the head but it's starting up again. So, I really enjoy that.

INT2

Oh, excellent. And I'm guessing when you worked as a detective you were extremely busy. Pretty much probably near enough the full day. How have you managed since you've retired? Did you retire at the time of the brain injury or had you already retired?

RES

I got the brain injury the August was due to retire in October anyway but actually retired when I was in James Cook so that wasn't the plan. But my wife had manged to contact my last place of work, the boss, and all my sift came through for a surprise party on the ward. I saw one guy walk past and "what's he doing here?" and then I went down to the meeting room and all my shift were there having a little party, it was excellent

INT2

That sounds fantastic. I bet it was a lovely surprise.

RES

Oh, it was. I mean, the last thing I would think was actually being retired and be retiring when I'm in hospital. Can you understand?

INT2

Sorry your internet broke up a little bit, but did you say ….

RES

The last thing I would think I would actually be retiring when I was in James Cook

INT2

Yeah, it's not a situation you would expect to find yourself in is it. So how have you been sort of filling your days really. What does an average day look like for you?

RES

Usually, I try to go for like at least around a two mile walk in the morning. So, I get myself some fresh air. I know the route because I'm aware I get lost when I go out and forget where I am but it's a route Ive walked loads of times, so I know where I am. Just like a lap round the estate where I live just round the local stress. no Just like lock run, this state was a local street. So, I get a paper and come home. The main reason for getting a paper is so I could do the crossword and keep my brain active. So, I've got to do that. Then I come down, watch a bit of tele. What I've been mostly doing is reading my Kindle. So that keeps me occupied and studying and focused on something and takes your mind off what else is going on

INT2

It sounds like you've adapted very well to sort of keeping yourself occupied and making sure you're not sort of just sitting and stagnating and such. And have you always been very active. Have you always liked to be busy?

RES

Ive always been active. I used to go to the gym, I used to go out running, but I get too tired for the gym, so I stopped that and then I'm wary about getting dizzy if I start running, so I prefer walking now and taking my time

INT2

And are you quite happy with the amount of activities that you are doing at the minute?

RES

Yeah, yeah.

INT2

Brilliant. So, if you can think just changing the subject a little bit here? And how would you define the term loneliness?

RES

How would I define it? Being basically on your own, not having any social contact with anybody

INT1

And is that something that you've ever experienced?

RES

Not really, I keep myself occupied. I keep in contact with a few people, friends, family, volunteering helps as well…So I wouldn't say I've ever been lonely as such. Having a brain injury it's easy to drop into being depressed and so I'm well aware of that and make sure I don't go down that road if you know what I mean.

INT2

Have you always had that outlook to always sort of make sure that you're keeping yourself busy? And you're looking after your own wellbeing?

RES

Yeah, it's like, I know how easy it is for people to drop into depression. The example I give was when I first cut the lawn on my own it took me half an hour or so. Whereas before I could do it all in about 20 minutes. So, what I did was set it as a challenge for myself to see if I could do it quicker the next time and build on it from there

INT2

Brilliant, a great, great attitude to have, keeps you motivated. If you were ever to have feelings of loneliness, or to feel like you were perhaps getting a little bit depressed would you feel comfortable talking to somebody about it? Do you have people that you'd feel close to that you could express how you were feeling?

RES

Yeah, I wouldn’t feel a problem talking to….me talking about my depression or somebody else who's depressed?

INT2

You. Yeah. Would you feel comfortable sharing how you were feeling?

RES

Yes, I'm sure I felt depressed, or whatever that's going down that road, I would tell somebody straight away.

INT2

Yeah. And do you feel that people who would be quite open to if somebody was experiencing it to either to pick up on it with somebody else or to be open to talking about it with them?

RES

Yeah, they would do. Yeah.

INT2

Do you think lockdown has impacted how you think about loneliness? Or do you not think it's made any different?

RES

I tend to put loneliness at the back of my mind during lockdown, and try and concentrate on something good? If you know what I mean

INT2

Do you put it at the back of your mind in case It's a slippery slope that you might fall into? Or is it just something that you want to… You don't think you need to think about if that makes sense?

RES

I try not to think about it. I mean Ive gone from have 30 odd people on a shift working and im lonely some of the time, but I tend not to dwell on it

INT2

And just focus on those positives?

RES

I have a positive attitude.

INT2

Brilliant. So, while we’re on the positive attitude. Has there been anything in particular that's helped you during lockdown do you think?

RES

I don’t think there’s been one thing, it’s just...keeping positive when I’m dealing with stuff. I mean, there’s times when, sometimes my wife works nights. So, I just keep myself occupied and plan what I’m going to do and keep myself busy and I’m fine really.

INT2

Brilliant. And has there been anything that you found particularly difficult, or would you say that really your life’s not being much different to how life was before?

RES

Beforehand, before the accident, I was quite a bubbly outgoing person, but now I seem to be more reserved. When meeting new people or people I don’t know instead of starting conversations I might add in the odd comment here or there as opposed to being at the forefront.

INT2

And have you missed that interaction? Have you missed those conversations like that?

RES

Yeah, I have

INT2

So, if we think about just sort of the last 12 to 14 months during lockdown, do you think there's anything in that timeframe that's been especially difficult, or had you already adjusted or adjusted socially anyway?

RES

It probably helped having a brain injury because Ive become adjusted. Everybody's in the same boat, so to speak. So, I know it sounds strange, but lockdown might have helped me because everyone's feeling the same. Like my neighbours, they come across and chat and talk a lot more than they used to. So I think they've realized that everyone's in the same boat during lockdown

INT2

So, in that respect, do you think it's been positive in terms of reaching out to neighbours and sort of the community around you that you perhaps wouldn't have done had we not gone into lockdown?

RES

I talk to my neighbours a lot more than I did before

INT2

And what about sort of once lockdown is over and everything opens up again? How are you feeling about that happening?

RES

I feel a lot safer in that I've had both my jabs so Ive had both so in that concern but it's a bit wary, a bit worrying at times like everything suddenly opening up again, getting back to what it was like before. So, like you said, it's just like having a brain injury. Just take small steps and get back into the community and get back, readjust yourself back into the community.

INT2

So, would you say you see it again as like a bit of a challenge like mowing the lawn that each day you're going to sort of give something new to adjust a little bit easier and adapt to it?

RES

I mean that’s what I’ve always done and so its built into me, so it does help.

INT2

Yeah, it's a great it's definitely a great attitude to have. Definitely keeps you going

Interviewer(s): ***INT1***
INT2

Respondent(s): RES (P13)

INT1

Okay, so can you start off, If you don't mind? Could you just tell us a little bit about your brain injury? Was it 2018?

RES

It was, yeah, so in August 2018, I had a cycling accident, I was hit by a car. And as part of that I got a traumatic brain injury.

INT1

And how much of that is affected your daily life since then?

RES

Every day, I have, I guess quite a few things. So its caused quite a lot of damage to my executive functions, which is probably quite common. So every day, things like my, I've had, I've had numerous psychological testing. And that came back with things such as my speed of processing is about 10% of what it used to be. My working memory is likewise at about 10% of what it used to be. So some things are very difficult. Trying to do, trying to do simple things like follow a recipe and cook it at the same time is a is a bit of a disaster. And everyday tasks, like shopping are problematic. leaving the house can be difficult, I'd like all these things. You know, for the last nearly three years, I've learned ways of managing some of them. But those basic administrative daily functions are very difficult. Speed of processing means that a lot of other things are affected. So I can do some activities for short while but then my brain quickly gets overloaded, so I can drive for about half an hour. But after that, information processing is too effortful, and I'd have to stop or things such as I also can have problems with bright lights, and particularly sound sensitivity, really difficult with sound sensitivity. I have got quite bad fatigue on a regular basis, and not made any better by disruptive sleep patterns as well. And I used to have anxiety and depression and PTSD aside from that I'm fine.

INT1

And how's that affecting your work life? can I asked what do you do for a living? Sorry.

RES

I'm the head of social science for a government agency. So one of the things that surprised me right from the outset is that my research brain is, is absolutely high, that 30 years of research is still chattering away there. So I'm fortunate I have a very supportive employer and I am the head of profession there, So that's come with quite a lot of discussion about what I can do what I can't do. So I have weekly treatment with the REDACTED brain injury service. And my newest psychologists have meetings, for VA meetings with myself and my employer and to discuss quite a lot of these issues. So there's things that I need, I guess what's called reasonable adjustments for so I can't manage calendars, or I can't manage spreadsheets. There's a lot of things around that. The filtering emails, that sort of thing, prioritization. However, still having a specialist research capability. In quite an unusual field of work means that I'm quite valuable to my employer from on that basis. And therefore, I've retained my job with some adjustments and with administrative support. And that has made a big difference. I've had some substantial periods of time off originally, six months to begin with, and then three months of faze return, came back to work for a year with not much knowledge about how my brain was functioning, and then had to stop work again, for another eight months, had another couple of months of faze return. And I'm now back again with some adjustments at work. So yeah, there's I think it's a complicated picture. With brain injury and employment. I'm very fortunate I have an employer who's been prepared to come on what has been an extremely high learning curve together on that basis.

INT1

brilliantly it sounds like the they've been quite supportive and willing to sort of adjust and adapt to your needs as well to make sure that they keep you. Did you have any rehabilitation when you first left the hospital or since have you had anything at all to help?

RES

in terms of work or in general?

INT1

In general,

RES

Okay, when I first left the hospital I did, I did have multiple other injuries. So I had extensive facial fractures and spinal damage, damage with my breathing and so on. So I think I think certainly, initially huge amount of support was on critical care around that. So obviously, ongoing physio, dieticians, because obviously I couldn't eat for quite a long time. And at that point, I still hadn't been diagnosed with a brain injury. So I would see my GP with increasing problems. At that point, it was classified as concussion. And then a growing realization that it was more extensive than realized and then I think it was probably a good eight months after my injury before I had formal neuro psychological testing. And that was a four hour test, you know, over at Liverpool. But until that point, I had no treatment for a brain injury, headway were brilliant. access to their helpline and

information leaflets was crucial. My GP was really supportive, but obviously not a specialist in this field. She was very good. She put forward a referral to me to the north Wales brain injury service. But at that point, as I understand it, they were not, they wouldn't take any referrals within the first six months of injury. I mean, it's massively oversubscribed, services, as I think many NHS services are. So I didn't in the end get a referral and an assessment with a low for brain injury service until probably I think it was knocking on about 18 months after my injury, maybe a bit shorter, maybe about 16 months. And since then, they've been absolutely superb. So I have weekly treatment sessions there. I've seen both a neurologist and now I'm under the care of head of service users who’s a psychologist.

INT1

Are you finding that that's helping making any changes to how you're managing on a day to day?

RES

I would I would say it's critical. Yes, I don't I don't. I see in the situation I'm in now. in many, many ways, I think understanding in terms of what on earth is going in my brain and why. Tactical techniques to help try and manage those things. But I think probably the biggest one has been psychologically the adjustment you have to go through if you have a brain injury is probably something you can only really understand if you've had a brain injury. And I think without that weekly treatment, that would have been very, very difficult. So so yes, I think I think without that weekly treatment, I wouldn't it I definitely wouldn't be in the situation I'm in now.

INT1

That’s good, has there been anything particularly difficult you found with the rehabilitation? anything you found really hard?

RES

I think there's one, there's a massive difference between knowing you have a brain injury and even knowing the reports. And knowing some of the wider research. I'm a researcher, one of the first things I did was spend the last two years going through more than I've ever thought I'd ever really know about neurology and psychology and brain injury, but there's knowing about it, and then there is accepting and adjusting. And I think the accepting and the adjusting is a much harder and much longer journey than I thought it would be. So that's probably been the hardest aspect of, of rehabilitation. And unfortunately, in my view is that you can't really take onboard the techniques to manage without really accepting the type of injury that you've got and the implications of that. I think I've had many a conversation when I've been asked how many times you're going to run at the same brick wall before you realize you can't get over it, I thought probably quite a lot of times really. But yeah, it's, it's a different, it's a very different aspects to it. And they really do need to go hand in glove.

INT1

Ok, Can I just check as well? Sorry, did you lose consciousness at the time of the accident?

RES

I did

INT1

You did?

RES

Yeah. And I have I've had I've got a period of quite extensive partial memory loss. So I lost consciousness during the actual accident, but then there's quite a quiet period afterwards, where I've only got, I guess, kind of snapshot memories, fragments of memory.

INT1

And were you in a coma for any time? Or was it quite short? Who was it just when the accident happened that you lost consciousness? Or did you stay in hospital? in a coma for a while?

RES

No, no, I wasn’t in a coma so it was just during the accident.

INT1

Ok, So if I can just move on to sort of the last, what are we now? 12 to 15 months whilst we've been in lockdown. Can you tell me a little bit about sort of the people that you've seen on a regular basis that you've helped that you've had any socializing with either face to face or online? Were you still working, were you able to work from home for some of that? Or are you who you are.

RES

Yes, I guess I guess this is where I mean, everybody's life gets complicated, doesn't it really I had just stopped working the February before lockdown started in March. That was, to be honest, a pretty bad point for me, I'd had a close family member had just died. And that combined with not being able to cope at work, combined with only really, I guess, starting to have some treatment. So you know, it had been the realization that I in terms of my brain injury, that it wasn't going to get better. And by definition of that, and as I wasn't going to be able to overcome it on my own, that I couldn't cope with work that I just lost one of my closest family members. And then COVID came marching over the hill about a month later. So it for many, many ways, it was a very difficult time. When

COVID first started, one of the things I didn't realize it would trigger was a very similar response to my initial PTSD straight after my accident. So I had, I really wasn't expecting it was a very much a heightened threat reflex. And that made me retreat significantly. So I guess really, by, by the end of March, I wasn't working, I was feeling very isolated from family during the period of bereavement. And psychologically, I perceived every other living human being as a substantial threat, which meant that I couldn't, if I saw another person on the horizon that was too threatening. So I would go out at about four o'clock in the morning for a walk, when they could guarantee that there would be nobody around and retreat back inside before I thought anyone else on the planet would get up some of the messaging around COVID I think, maybe it hasn't been recognized that for people with a heightened threat response, the messaging around now anyone can catch it, anyone can pass it on anyone can die of it is the any one thing is not a really very good message. And a lot of the very heavy sort of visual imagery that went around with it with with some breathing issues as somebody who struggled to breathe as part of their trauma was again quite a triggering visual imagery with a lot of the big danger signs and everything. So it created a very threatening environment and without really any particular conscious thought I immediately just sort of shut myself away for a long period of time. switched completely to doing you know, basic functions like getting food on 100% online delivery. So that was that was initially that was a very difficult time So, so yes, but obviously certain things shifted to online gradually I mean, I'm, I'm really fortunate I've got a fantastic network of really superb friends from, you know, before my brain injury. And they are, you know, quite comfortable with online technology and with the with the lockdown an awful lot of them after a month or two started to shift to setting up ad hoc online communication networks, private messaging support networks. And I was able to kind of engage with those without feeling then threatened by the mere sight of people.

INT1

And how long did that last for? How long did you see people as a serious threat for. everyone was a serious threat to you sorry.

RES

I still have some of that actually. Still now. So I haven't been in indoor places with a another person since COVID. I'm much better now outside. So I've been able to do a lot more outdoor activity. And that gradually improved. So you know, I guess probably like a lot of people for the first few months you got used to conversations were had, you know, on the doorstep with your neighbours on the other side of the street. And then I think you could finally meet one of the person you know? two meters apart. And I gradually started doing that. I mean, I've always been you know for 30 years, I have been a mountaineer, and a competitive cyclist. So I'm used to spending time outdoors, all of my friends and social network are outdoor people that are climbers and cyclists, and bikers and, and everything else. So outdoors was somewhere we were comfortable. So meeting people, one to one outdoors became a really valued part of my life where, you know, when we were allowed to do that larger outdoor groups was a bit more problematic, because I can't filter background noise. So I'm fine with a one to one conversation. But if I'm with a group and someone else is having a conversation, that's really difficult. But I'm lucky that the groups that I go out with, they were never very large anyway, maybe be about eight to 10 people, you know, mountaineering, friends and stuff who'd go out. And they were quite, they knew about my accident, they're quite comfortable with

the fact that I would, in effect, walk with them, but not in ear shot. We'd kind of go up the mountains, I'd be maybe know 20 meters away, upwind or something where I couldn't hear the background noise. But I still felt I was able to be part of something and it was safe from a COVID perspective. I've just started to try and address the sort of threat response from being an indoor environment with support from the brain injury service. So we're starting I do have quite severe panic attacks if I have to go into somewhere and there's another person, which is unpleasant, but manageable in a, in a sort of therapeutic or clinical environment. Not the sort of thing you want to happen in Tesco's. So, so yeah, so that those sorts of things are a work in progress. And like everyone, there's been the ups and downs of when you're allowed to see people and then when you're not. And an awful lot of my social life has gone online from online film nights to you know, online chats to, you know, I'm sure we've all been there. I've even gone to birthday parties now online and made birthday cakes at the same time. So, you know, there's been all of that online activity. Which is, you know, which I've been able to be a part of from people from all around the UK friends from all around the UK.

INT1

And how have you found that online communication? It is something that because of how your feeling without being surrounded by people it's actually been a bit of a benefit to you? Or do you want to get back to sort of face to face how have you thought about the technology side of things?

RES

I think for me it's a strange one. It's certainly made socializing much more feasible. Before COVID came along. There's so many things I couldn't go too you know people said you want to come to the cinema? Or do you want to come out for a meal? I mean, that's the worst one was like, Do you want to come out for a meal? Or maybe even go to the theatre? One night? And you know..

INT1

Can you hear her REDACTED?

INT2

No. I think Sue is breaking up.

RES

Sorry, that's better I'm my reception is trying to come away the thing at the

INT1

It's okay, I can hear you now you're videos frozen, but I can hear you

RES

Is that any better? No videos still frozen, are you alright to just hear me?

INT1

Yep, perfect If you wouldn’t mind just sorry, I didn't get any of the bit about that the event that you were invited to?

RES

Yeah, so, so, so going out or even something like the theatre very difficult, because people would just chat. And I tried a lot for a long time to go out and try things I'd go out trying wearing earplugs. So I'd go out trying for five minutes and not being able to cope. So shifting to online has been an awful lot better. And I've been able to be part of things that I wouldn't have been able to have been part of. at all, sometimes it's a bit difficult. If you've got one of those zoom meetings when everybody wants to chat all the time. I'm sure we've all been in them. But generally, it's been a lot better. And also the other thing is, is not having to travel. So say I can I can drive a bit. I used to get trains, which was okay, if I could just get on one and then get off at the end. But changing trains was difficult in terms of processing the information. So yeah, shift to online has meant that I've been able to be part of things. The downsides, I guess is. But as we all know, it's very two dimensional. And having more personal conversations are difficult online. So not being able to have those more detailed conversations with friends was difficult, particularly, you know, the combination of mental health, brain injury and belief and other times when you want to have good conversations with people in person. And I think the other thing is lack of physical contact that we talk about social contact as if it's purely verbal. But there's so much about social contact that is nonverbal, I have about an hour usually where I can talk to people, and then my brain starts to shut down. And I need what I call kind of quiet time. And that's really difficult. If you can't be with people in person. I don't think anybody particularly would enjoy spending time on zoom in silence. It's not it's not really going to work from that perspective. And I think the other thing is, is that I was probably at that point would have benefited from having the opportunity to meet with some of the local support groups for local headway supporters if you’ve had brain injury but of course they’ve completely shut down. So although some stuffs gone online. And not being able to be of any group in person has been a bit difficult. There's so much more communication that goes on in person than it does on a screen. But overall, I would say that COVID has on balance been more about facilitated more integration than isolation for me. But that's not a black and white. It's, it's not that it's been I felt more isolated. It's not that I felt more integrated. It's actually caused both and I think we should see it as both and not as an either or.

INT1

Sorry so it’s a steady balance of pros and cons for various reasons but nothing outweighing each other?

RES

No, I mean, I say on balance its probably been, I've been more socially engaged, the less, but the close personal relationships and not being able to see, for example, my surviving family, you know, it's very difficult. And I think, you know, anyone who's lost anybody during COVID knows that the psychological effects of not actually being able to be with your family members during bereavement probably understands a lot of that.

INT1

Yeah, it's it's, it's not been a very pleasant time, especially for something like that. It seems like it's, it's come definitely at the worst time it could have done for yourself as well.

RES

Yes, I suspect timing wise, it wasn't. It wasn't great. And I honestly think one of the other things that happened, doing some thinking around at the moment is that I'm going through the process of adapting to a brain injury. Is one where I think probably quite a few people have a period of not denial, but attempt to carry on as normal as much as possible. And then a period of slower adjustment. And then a part of that adjustment is being exposed to situations which are difficult, which allow you to try and build compensatory strategies or coping strategies. So for example, for myself, not being able to be in a place with other people, because of not being able to filter background noise, or visual complexity is the other one you know, if I go into somewhere with lots of signage, or bollards are very difficult but COVID meant that that was should have been probably a year and a bit of gradual exposure, practicing techniques to manage, and learning how has almost gone the opposite way. So

I've not been exposed to any of those faces, which means I think the process of rehabilitation has been stalled, because I've not been able to engage in places in that way. And I think as a consequence of that, my ability to cope socially has deteriorated. And now we're at a point where people are going out more and doing more things. I feel even further behind. So that sense of feeling I'm actually now feeling well, I'm less able to cope socially now than I was before COVID. Because partly because of the psychological threat section, but also partly because I've lived in a silent world. For a year and a quarter I've my ability to manage background noise or different conversations or busy visual environments has really deteriorated and probably would have done even if I've not had a brain injury, but what it has disrupted is that process of rehabilitation? And the consequence of that, I think is really quite a strong sense of an expectation of future increased loneliness.

INT1

How are you feeling about sort of lock down lifting and social engagements becoming more popular and people being out more? How do you feel about life, returning to whatever normal is?

RES

The straightforward answer to that would be left out and left behind. And that there's been a lot of discussion around the empathy that's been generated through COVID that people have an insight into what it feels like to be socially isolated, to not be able to join in. But I think human nature leaves people to feel excited about the option of that ending. If I had the option for my brain injury to end, I think I'd be overjoyed, but I don’t so those challenges around social engagement for myself will remain with the reopening society. I think there's a double edge aspect as well one of its I could say I think my coping mechanisms and that process of rehabilitation has been disrupted and held back I would say so for the I don’t want to say this again. And the second of which is that it feels that you feel more isolated because you're not able to take part in the general feeling happy about things, and the online social engagement that I used to have will go, you know, inevitably our friends are able to actually physically seeing each other in person in groups or get the cinema, then those online activities that so many friends had set up informally, will disappear. So I, the way I described it, it's a bit like I don't know if you ever get the train for where you are. But when you used to go and catch the train, and the trains been delayed, and you all stood there on platform together, you know, all not able to get on the train or feeling grumpy, and stuff, or whatever, and then train comes along, and everyone else can get on it and go off on their life journey. And you're the person who has to stay stuck on the platform, you cannot get on that train, and carry on that sort of social engagement. And it, it does feel that the return to normal is not an option for people with a brain injury. And I guess that also then that brings that psychological impact, that is the one that I probably struggle with the most, which is that brain injury doesn't get better. You adjust, you learn to cope, you can be very fortunate as I am that you're able to retain, you know, a lot of functions. But it doesn't get better. And I think that optimism that other people feel like they're being released from lockdown is great. So you can go and join in and go to the pub. And when you can't you sit there and you think God, the last time I enjoyed going to the club was three years ago before I have an accident and you know, I can't enjoy that experience again, I can go for a short period of time, but it is hugely effortful and very painful. And so it's that I guess it's a bit like if you had an injury, and you thought everybody else in the population had the same impediment as you. And then suddenly everybody else was able to get better and you weren't. And it's that kind of feeling really.

INT1

Do you feel that in some ways, lockdown has made your social situation worse, because had lockdown not happened your rehabilitation and your adjustments that you would have been making over this last 18 months, would have possibly put you in a better position now?

RES

I think it would have been different. So I think in some ways, it's made things worse, because I’ve not been able to continue with the process of factors and desensitization, I don't know how well I would have advanced and being able to cope in different places if we hadn't had COVID. But at the same time, because COVID disrupted everybody's lives, you know, all of us. It stopped me trying to fit back in again to my peer group situation, because my peer group just was disrupted as was everybody. So where as before, I was still trying to you know go to an international conference for work or I was trying to go into the office sometimes try and join back in with my cycling groups. And each time I would push myself too hard in an attempt to try and fit back in to the life that I used to have. But having the whole of life disrupted for everybody has given me also that breathing space to

acknowledge that I can't continue in the way that I had done and sad though that might be at least it is also a degree of acceptance. And I think with that acceptance means that I will probably adjust better from that psychological perspective. So I think COVID has had those two really different aspects. It's sort of reduced my ability to develop compensatory strategies but it also had the benefit of buying the space and time to actually break from my former lifestyle and give me space then to maybe form a new lifestyle and that's very difficult to do when you first have a brain injury because you automatically want to try and go back to as much as what you used to do before. And so it's given me that space to change

INT1

Ok so a slight change of questioning, the term loneliness, how would you define that?

RES

I'm trying to give you a person answer rather than a work answer. I'm a social psychologist, so I could give you a really good detailed theoretical response. But I don't think you want theory, you probably got enough of it already. How would I define it? I would say an internal sense of lacking social support, not out of choice. So there's times when I think you can be alone, which is very different from being lonely. And so I think it's, it's to do with the choice aspects, and also to do with the sort of feelings of sadness or needing support from other people.

INT1

And is that's something that you've experienced?

RES

Yes, I think I think quite a lot. Both before COVID and doing COVID.

INT1

And I know, we've mentioned earlier about sort of talking via technology in about personal things, or things that have been bothering you. is it something that you think you could talk to a friend or somebody about, when you have been feeling lonely?

RES

A little bit, it's one of those ones, that's quite difficult to talk to people about, although I think it's more of a general discussion amongst my kind of friend groups around. It's important that we support each other and that we're there to talk too if anyone feels lonely. One thing I notice is that a lot of people offer support to anybody who might be slow, like feeling lonely. But not many people

actually say I feel lonely. And that is not something that you hear that often. And it's a difficult conversation to have. It’s a difficult conversations to have, I think, for a number of reasons, one of which, because it might, it makes me feel you know, in some way weak or not wanting to, the biggest one I guess is not wanting to say it because you don't want to guilt trip your friends into feeling that they need to be there for you more. So you kind of think or don't say anything, because I've got such nice friends that if they thought I was lonely, they'd be you know, in contact, and therefore, for you then feel like, Well, they've got busy lives, they've got a lot to do, I don't want to be a burden. I don't want to make my friends feel guilty or awkward or feel that they had to do more or should do more. So you tend to be silent, partly because, as I said, the predominant voice that that I hear is people offering to help people. You don't hear many people actually say I am a person who needs help. And it's much harder to ask for help. Much, much harder than it is to be the person who offers help.

INT1

Yeah, definitely. I can understand that. Do you think that either locked down or your brain injury has altered how you think about loneliness. So you mentioned that you had feelings of loneliness before we went into lockdown, has that been the same or have you thought any differently about it?

RES

I Think certainly since my, when I have my accident, not being able to be part of things socially. First of all was a choice, And secondly, then it became something I tried to overcome, COVID came along. And I think loneliness has been much more of a public discussion. And that I think, has meant that I've also reflected on that more on myself, because it's being more publicly discussed, rather than it being something that I've kind of buried away and sort of oh don’t think about it. It's been something that I’ve brought out more in my own mind in terms of reflecting on it myself and thinking, when do I feel lonely? or Why do I feel lonely? And it's an interesting question as to whether or not that self-reflection during periods of isolation, is it? Is it? What's the impact of reflecting on loneliness? Whilst you also feel lonely? And it's not? It's not an easy answer as to what that process of reflection might actually be? Should it? Does it? Does it cause a bit of a kind of downward spiral of reflecting on something in a situation where there's very limited opportunities to do much about it?

INT1

Yeah I can totally understand that. Has there been anything that you think had stood out

that has helped you get through lockdown at all? I’ve just noticed the time, Are you okay to continue? Let us know, if you're starting to feel tired, or you want to have a little break or anything? It’s not a problem?

RES

No That's fine. I'm okay.

INT1

So, is there anything that you'd say that stood out that has helped you through?

RES

Um, in general, in terms of lockdown? Yeah, I think I think a couple of things. One of which is living somewhere where I have access to extensive open moorlands, a big, wild empty beach, and being able to be outside in a somewhere where I don't feel threatened, and where the visual distraction and the background noise is very low. So it's good for my brain. It's been good for me psychologically. But it's got me out that that made a big difference. The other thing I started to do, which really helped, was to dig out, sort of a 30 year old interest in photography, which I hadn't done since my 20s. And that process of focusing particularly kind of close up or detailed, really helped to manage some of the anxiety and so on. But I think that I think the things that probably has made the biggest differences is what I call my kind of one to one walk and talks. So throughout the last, you know, as soon as we were able to meet one other person, there's about you know, four or five really good close friends that I meet with, we tend to meet up for about one or two hours. And we have really good quality engagement with each other that we would not have done otherwise. And that, to me has been the absolute benefit of COVID and a brain injury. So whereas before I would have known them, and seeing them in social groups and social settings, and we might have occasionally had a private chat now and again, but most of the time, it would have been a you know? Hello, how are you? Great, fantastic, whatever. Being one to one, and walking somewhere where there's not many other people has meant that the quality and detail of those interactions has been really, really valuable. And I think without that I would have really struggled and it's one of the big things that I'd really like to continue with after lockdown. So I think I think a couple of things that's really helped me get through as being those one to one walk and talks and also the weekly treatment sessions that I've had. Without those it would have been a bit difficult. It's been a bit of a venting experience. Having reached to let it all out

INT1

Yeah definitely, I like the walk and talk I might pinch that little phrase I think. What about the On the flip side, what do you think's been the hardest thing? What's been the most difficult for you?

RES

The uncertainty. I think having a having a brain injury means that you have to face a huge amount of uncertainty, per se. Learning about I still learn about I still find every day it's like, you know, usually goes long along the lines of shit I don’t know how to do that. So, and also uncertainty about, well, how long will it take me to start to adjust? How long will the uncertainty about learning what compensatory measures I will learn, there's a huge amount of uncertainty in brain injury, and there's a huge amount of difficulty and COVID in terms of uncertainty as well. So for me, it's sort of the psychological impact of feeling that your personal sense of self is incredibly uncertain. And then the social sphere in which we all operate is also incredibly uncertain as well. And that total instability is very, very difficult and very stressful because I think uncertainty does cause a huge amount of inner

stress. I guess that that's how that's been the hardest thing is layers of uncertainty, and I think they compound each other.

INT1

Thanks I can see how that's definitely been hard. And, Steven, do you have any questions that you want to ask?

INT2

I think you covered everything.

Interviewer(s): ***INT1***
INT2
Respondent(s): RES (P14)

INT2 **If you can just start, if you don't mind telling us a little bit about your brain injury.**

RES Yeah erm. The technical side of it. (unclear, 0:08). I've learned more in recent years in 2004, erm I found out that I had a brain tumour that had been there a long time. So it'd been growing since I was young. And now know erm that in my 20s, it was starting to cause problems, because of the pressure being there, and it was growing faster. So things like headaches and nausea that I was experiencing at the time, of character, they said it's as a result of damage, and my concentration isn't very good. And, My working memory, I'm told, anyway, went on to have my tumour removed in 2004. But then I developed an infection and a CSF very quickly after that, I went on to have erm (unclear, 1:01) inside around the brain that got infected. And then I ended up having a shunt. I have I've had several and I've had quite a few erm shunt revisions where it snapped or, you know, those kinds of issues. Erm but really, the initial, the major part major hurdle was the infection around the brain after my tumour was removed. So I've gone on to got a really good neuropsychologist. Erma and I've had some cognitive function tests because my mom was alive until 2013. And I heavily relied on her without realising with things like, if we were in, you know, with a group of friends, I'd be saying, What did they say? What's this saying? It's all that kind of thing. So (clears throat) it came to the forefront. Should I say? After I lost my mum so.

INT2 **Can I just ask sort of what erm, how it's affected your daily life. So before the tumour, and now obviously, you've already started to notice that things were not quite as they should be.**

RES Yeah I mean, I was working. Until that point, I'd also gone to university. So my, my plans for my life changed beyond what, like everybody else, you can't plan your life. I know that, but what I'd intended to do, so I'm not as outgoing as he used to be. I'm not so energetic, I tire very easily. concentrations poor, erm, I've got a number of conditions that made me I wouldn't say regimented and controlled by them. It just means I'm not so free spirited as I used to be, and just drop things and fly off somewhere. I'm none of that anymore.

INT2 **And you mentioned about having some cognitive functioning tests. Have you had any rehabilitation at all? Because of them or anything like that? No?**

RES No, it wasn't offered any

INT2 **Okay. Do you think that would have been something that potentially you would like or that could help you? Do you think or? (unclear, 3:19)**

RES I think at the time, it would have helped because I think like everybody else that goes through neurosurgery, you don't know what to expect. And afterwards, I used to say this, the only example I can give is say if you had a heart attack, and then you left hospital, you'd have like a leaflet with try walking for half an hour for the first few and then, you know, you have all that with and you'd have something there black and white. When it's brain you don't, and because everybody is different as well. Whenever I was going back for follow up appointments and asking loads of questions, and just the neurosurgeon was like, with me. So you kind of taking a long time to find out. And now I've gotten a psychologist to know more. So I've probably erm gotten the maximum out of what I would have got, but it's just taken a long time to get there, which adds to the confusion and the tiredness and everything else which you can't make sense of it at the start, if that makes sense.

INT2 **Yeah, definitely. There's so much going on and to try and take in it becomes impossible, doesn't it? Erm so what about if we think about the last sort of 15 months or so whilst we've been in lockdown? Err can you tell me about the can, how many people you've seen who've been in regular contact with?**

RES erm. Well, I was shielded. So I live on my own. I'm in regular contact with my auntie although she doesn't live that close. Erm, so speak to her most days. And then erm my neighbours obviously, I've got very little family left. So it's been mainly friends that I speak to but what appened at the start lockdown was very different to where we are now. So at the start, everyone was off work. Everyone was at home and nobody was doing anything. There wasn't any social distancing. And everyone was in contact all the time or messaging, oh do you need anything? And then obviously, society starts to change. Doesn't it and then people stopped. When I knew I wasn't under any illusion? Because I was thinking, these people stopped to think that actually, I'm on my own all day, every day, and I have to manage. It was hard at the start. Yeah, because I couldn't book slots for things like shopping, erm. But there are days when I'm really poorly and unable to do anything at all. And I have to just find a way to manage. So it start, everyone's really Oh, you know, because they haven't got anything to do either. I know, they've all got jobs. I know that what I mean, is the home thinking about oh, you know, I've got time now to think about something else. So the first lockdown, I was kind of getting bombarded with messages. And it was getting a bit draining cause i thinking, you know, I've got this appointment to go to, or this to do, or that to do in my house. And it was like people ask me all the time, then they start to filter out. And then it was like the problems with you, you need to get on with your life, we're all back at work. We're going to a party, you know, even though like those restrictions, people then started to go back to, you know, getting together in houses, although they shouldn't have done. So it's kind of been a bit of a roller coaster really. But I wasn't under any illusion, because I did

think, how come over all of a sudden these people were contacting me and I was thinking what happens when I go back to work, and I was right.

INT2 At first, I know you said it was quite draining. Did you appreciate where you're quite happy to have the extra contact that you haven't been having before?

RES Yeah, I guess I was I was. but then you've got in the back of your mind. What do they think is usually going on in my life? And they're all like, why don't they realise that? You know, I've had to manage all these things on my own before. So you've kind of got this. It was like, I don't know, I might have overanalyzed it at the time. But I was right. Because my hindsight, when I look back, do you think these people wont bother with me soon, you know, oh, come and stand outside at the window and see you, okay, and all that will come and stand outside your kitchen at the back, you know, and we can talk to you. And then they'll kind of get sick of the restrictions. But I've already got my restrictions, because I've got a bladder condition as well, which restricts me as well as head injury and tiredness, and fatigue and head pain and all the rest of it. So I've already had to adapt. And then you start getting the what you do all day, comments. And so you think, oh, you'll be going back to work soon. And this is where people changed here. So it was it was it was great hearing from people but did get a bit tiring with the questions I think.

INT2 Yeah. And it sounds like you went from not hearing a lot from these people to all of a sudden, they went into lockdown had some spare time, and we're contacting you more. Is that? Is that a good thing?

RES Yeah, yeah. I mean, I met these people. So Friday nights in the pub every weekend. But then it was like from eight o'clock on a Monday morning. Like it was relentless messages. I can remember being on erm a call to consultant and I had so many messages. I was like, Oh, no, afterwards, I can't take time to read it all. You know, it's like that. So I don't know. I guess that's how they all handled it.

INT2 If we think back to before the pandemic, erm what was your social life like them? Were you out? And about much did you be seeing these people (background noise, 09:10) contacting you?

RES Yeah, in the pub on a Friday and Saturday night. But I'm really unhappy to go out on my own and leave when I need to leave. So I was more in control of my interactions and I'm not anti-social, I'm quite the opposite. But I've also travelled alone before my brain injury. Erm and I've been to places like Hong Kong and Dubai on my own. So I'm quite independent. That is the night goes on and starts to get tired and concentration goes down. I've got this bladder problem. And I think now it's time to go because I'm not really listening to what anyone's saying, so I was more in control. Pandemic come along, and like saying oh we will come by

tomorrow. And you know, we'll do this at such and such time and it's bit like overwhelming at start. If I'm honest.

INT2 **Did you find it was slightly more on their terms, if they'll come to your window at this time because they were out for a walk as opposed to what time would it be suitable for you?**

RES Yeah, it was the time that'd be suitable for me, or are we gonna be passed in or we'll be in town, do you want anything to was never erm intrusive in that way? No, it was? No, it's never been like that.

INT2 **And then so in terms of sort of your daily activities. How have they changed? Obviously, you've been shielding, have you doing anything else to sort of keep yourself busy or changed your daily routine during the lockdown?**

RES Yeah. I erm my aunty has got smallholding in the nowhere, which is great. My GP said to me erm, If you if you're going to go out for a walk, I'd rather you kept away from people. So I'm happy for you to go there. So i started going every Sunday, which I never used to do. And they've got a river going through their fields. And I wasn't used to going out walking every day. So I was doing that at first, just on a weekend. And then I started walking locally on my own. Erm I have to go like when I've had neurosurgeon saying you've got to have drank plenty and that my bladders got to be empty, which is my other problem. So at the start of it, I'd say I wasn't active at all. But I was getting enjoyment out of going over to my aunties on a Sunday and seeing the changes to the seasons and they've got horses and animals. So it opened up my eyes really. And I was actually thinking, I would never say it in front of her. I thought to myself, she's been in 30 years, and I've never walked around her fields, that is so bad. Whether she's noticed that I don't know. But she hasn't said anything. So that's been really nice to get to know, it's nice to see changes and learn about keeping you know, there's rules with agriculture of what you can and can't do. And really I should have known because she's been there 30 years, and she's my closest relative. And I was thinking actually she's but it's been really it's been education of rarely, you know what you can and can't do and public footpath and all that. So erm yeah, it's, it's been an eye opener. And actually, it's given me an opportunity that I wouldn't have taken.

INT2 **Yeah, definitely.**

RES I'd be still sitting here ignorant, not knowing, you know, about whatever that what the horse is doing and overfeeding them apples and all the rest of it. And yeah, it's good. It's been good in that respect. Got something from it.

INT2 Brilliant. And is that something you'd like to continue again?

RES Yeah, so now, you know, I noticed things and if the lands because part of her field floods and this farmers on neighbouring properties. So I've noticed things now. So I'm keen on a weekend to go walk around and have a look at what's going on. So yeah, but I'm walking locally as well.

INT2 Excellent.Yeah. Erm so slight change of sort of conversation. How would you define the term loneliness?

RES That's difficult. Well, how can I define it? I can't say to you, it's been on your own.That's not loneliness, because you can be with somebody and be lonely. Erm think loneliness is being able to communicate how you feel as people. And some people, you can't do that I wouldn't open up to my auntie. But then there's certain friends that I really can open up to, I've got some close friends that aren't in this area, in other parts of the country, and I've always had them at the end of the phone. And I noticed this problem here. So I've never felt that isolated locally, in that there's nobody on my wavelength, but then I've bumped into old friends of whoever and formed some good friendships locally. Not many because some people are very judgmental that one I'm on my own, two I will never have children three I don't drink when I do go out. So they've all got this stigma with me. And I find that isolated that I don't get invited to things because I don't drink. So that side of it, that does make it a bit lonely, but I can't say to you, it's been on your own. So I think it's communication thing. Is it? I don't know.

INT2 Yeah, no, that's perfect. Whatever, whatever you think it is to you, that is that is

RES a close friend in a marriage that she's been in for a long time. She can't communicate with her husband. So that's that's loneliness.

INT2 Yeah. And you feel if you were experiencing or were feeling lonely that you've got close friends. Did you say that you could telephone?

RES Yeah.

INT2 Be able to talk to?

RES Yeah. I'm gonna, don't make sense thing

INT2 Probably the ones that erm Yeah. Go on.

RES If this Anything medically that I don't make sense of, then I, well quite often I do I've got a very good neuro psychologist as well. So that helps.

INT2 Brilliant. Err sorry where you gonna say something

RES yeah I think you questioned, when I find myself questioning myself a lot, like why have I done that? Why've I forgotten that?. And then when, my newer psychologist will say, you know, when my shunt plays up, when my shunt plays apparently, they've seen that my memory goes my functioning goes, my communication goes or I get UTIs. And again, they they change my cognitive function dramatically. But then I start thinking, Oh, god, is this Alzheimer's, I'm questioning myself because I forget so much. So when she phones, I'll say I've been like this. She'll say, Yeah, but if you have this that and the other going on, yes, I have. And she'll say but we've seen this before with you. Because one of my one of my tests, I came down with a UTI. We had to break the tests up, because my concentration being so poor. And a couple of sessions are turned up. I wasn't well and I just went to pieces.

INT2 And for you, do you find that it helps having the neuropsychologist explaining to you are you better with more knowledge?

RES Yeah. Yeah. Because you question when you come out of hospital, and neurosurgeons don't talk about anything. How this is the effect its going to have, you don't get any of that. You know, you might feel differently. You might be this, you might be tired, you might, you know, have memory problems. Nobody says that to you. You kind of learn along the way. And I think I beat myself up for a very long time, where I was forcing myself to do things that even if I was tired of got a bad headache, you forcing yourself because you think you really used to be like, questioning and I nevre used to be like this, I used to do this. So you spend a lot of time with. For me it was an internal battle, if that makes sense. Until new psychologists come along and said Actually, no, this is how you know, this is how you're responding.

INT2 And have you found you've had much support from like, headway? And in places like that where people have gone through similar things? Do you find that that helps?

RES Yeah, used to have local headway meetings. I don't know what happened, they stopped, they started. Erm I don't know if they've resumed again, because the person kept changing

but I do read a lot of their stuff on social media, which is really good. It's very good. And it helps. I don't always read all the comments, because I think some people are sometimes I find it a bit overwhelming to read other peoples comments I will say that. I don't want to, I tend to distance myself because there's times where I don’t want to reply to people, within groups like to headway and then people use it to contact you and offload their problems. And I, I'd rather just read it, digest it, and then deal with it my way as opposed to sharing. If that makes sense.

INT2 Yeah. That makes perfect sense. Erm I've just got to ask, earlier you mentioned about feeling isolated when err your friends are going out. And often, they don't invite you because they know that you're not going to drink. Is there.. Have you ever felt comfortable enough to be able to tell them how that makes you feel? Or to share that? And have they changed or adapted because of that?

RES I've only said it a couple of one time recently because I've been happy to shield until after my second job. So at the moment, just I’ve said it not directly to somebody I said it to somebody else as part of the group. So no I've not said it. But also I suspect that sometimes because people see me for an hour in a pub on a Friday night, an hour or two hours maximum and now we’ve moved away from the pub being standing together. Now say it's a bit difficult when you’ve got brain injury, I think they think excuse again because they've seen me on Friday night dressed up looking different, make up on, having a good time cause there’s’ a live band, to well she’s not usually like that when she comes in the pub, there's a lot that and she looks ok, what’s she on about brain injury again, you know, she had the tumour removed. What's the problem? There's a lot of that, and a lot of an undercurrent of we look okay. There's a lot of that. So that's, I don't battle it. I just think that's them.

INT2 Yeah, definitely. It's definitely more their problem than it is yours. How are you feeling about society opening up again and or the lockdowns lifting?

RES I'm going to be honest, I’m a bit prejudicial with I'm happy being around people that are vaccinated and some friends that have refused it vaccine because they don't believe it exist err, that bothers me because there’s a lot of people like that it's not just my circle or my extended circle. Erm but that like yesterday I went for dinner with my aunty and sat outside the pub so I was fine. I don't know what the minute, I don't know. I'm here, one minute, oh, you just need to get on with my life now instead of being stuck in, like I like going out. But then the other part of me thinks you haven’t had your vaccines and you're working with these people. So I'm mixed emotions about it still. I'm not sad. But then now I'll be I don't want to go into hospital either.

INT2 No, I get that. Erm do you think if it came to it, would you lose friends if they hadn't been vaccinated?

RES I distance myself. Erm won’t completely cut somebody out. But I've distanced myself

INT2 Yeah. Is there anything that's been particularly helpful for you through lockdown or anything that you found that's helped you?

RES I think things like headways updates, social media, erm they've been helpful. Going out for a walk every day is definitely helpful for your mental health. Without a doubt. I always think you should find something beautiful in every day. So taking a photo of something nice that you see. That's helped, and my garden, love me my garden. So but also, like said that start going to my Auntie's and learning about countryside and rites of way and all these kinds of little things. We can't put the bush there because it's over right away just little things that you learn and then people come walking through where they shouldn’t, just lots of things like that I've been really interested in I found that's helped actually. But then I'm lucky to have that.

INT2 Definitely, I think err, I don't think you're alone there I think a lot of people have found things that they didn't expect to even explore and all of a sudden they’ve had this extra time and..

RES Yeah yeah. I went to a very close village yesterday I haven't been to before and it's literally 10 minutes away and it's beautiful. And I felt really guilty walking on the river erm. It was beautiful place and I just thought there's a few things like that there's a place that I used to go to when I was a child in a field where some rivers, we call it lady broth and people were mentioning that was getting ready to go there and then you think why haven't I been here before? There's a lot of

INT2 Yeah, definitely beneficial

RES i think i think that's beneficial type of byproduct.

INT2 Yeah, definitely. And we're the same we used to always go in the car to a beautiful natural place and yet when we went into lockdown we’ve got some gorgeous woods with little river rolling through would never have found it we hadn’t of been in lockdown.

RES Yeha, that’s so good.

INT2 What would you say has been the most difficult thing for you?

RES things like accessing shopping erm, other people's attitudes towards it like oh it doesn't exist or get on with your life or it's you know. It's not really out there Lis, it's conspiracy theory all the that doesn't help. And I think social media doesn't help that just about with some of the tabloids as well as a lot of negative media and there's a lot of bullshit out there that doesn't help. Erm otherwise, the only other thing is things like medical appointments, where the time to anything, you know, when you need them see what I'm trying to explain something to neurosurgeon, because they want to know exactly what pain is and I'm doing this on the phone pointing to it, think he can't see me. Those kinds of things, don't help. Without doubt.

INT2 Yeah, definitely. Have you found that you've had any problems getting appointments due to lockdown and COVID, or have they been ok?

RES I'm probably one of the lucky ones, I've been fine.

INT2 REDACTED, is there any questions that you want to ask before I move on to the scale?

INT1 Yeah, the only thing I erm wanted to ask more about, I guess was, so you've been shielding for the past 14 months or so?

RES Yeah.

INT1 Have your levels of loneliness or isolation been more over that period of time than prior?

RES No, in all honesty, and yet, I think people would expect you to feel more lonely. It's been the other way. Because people haven't been at work, or have been, you know. The only time I've really felt it is when a group of friends gathered together last June, for a close friend's birthday. And then they were all together, and they shouldn't have had a house party. There's more than 11 households in the kitchen when I had a face time. Right. And then I felt it. And then I thought actually, no, had I come away. We've already had a bereavement in the family this year, we've had a lot of bereavement. Stress somebody else. So that's when it's hard. But then people have probably been a little bit more thoughtful as well. So it's just now they've gone back to their lives, if that makes sense. That's where it's hard. That's where it starts. Because you think actually, you've you've really made the effort. Now suddenly, you've gone back to life and you've stopped bothering. That's where it gets hard. Because think, how do you think I feel.

INT1 I think what you're talking about before is that hidden disability aspects of the brain injury, isn't it?

RES Yeah, friend phone last night, I said, Oh, such and such it's really fed up hes only had his daughter to speak to since Friday, and it was Sunday and everything said well, he goes to work every day, some of these people, because I'm not always that sympathetic. Especially if it's someone I'm not keen on and I said how do you think I feel I'm on my own all day and I actually said it yesterday then thought actually I'm not that bothered, I get tired like, if a friend came Saturday day and was in the garden after a while I get tired cause I'm concentrating. So it's one of those I can't find a balance can I.

Interviewer(s) **INT1**

INT2

Respondent RES (P16)

RES2

INT1 If we can start would you mind telling us a little bit about your brain injury?

RES I can't remember the dates. But was it eight years ago?

RES2 Err No, it was 12 years ago,

RES 12 years ago, we were working in Spain, and in a house near Estepona, in the south of Spain. And I was driving a JCB to pick up some stones, because we were building things. And with the JCB, I went down the hill, off the off the track. And then I ended up in a valley somewhere. I was in coma for four weeks. So and then I was taken to hospital in

RES2 Malaga,

RES Malaga four weeks there, or in coma. And then finally, err I was then flown back to Holland, where I'm actually from, and I was there for, I don't know how many months

RES2 REDACTED was then in a residential neuro rehabilitation centre in Holland for another two months.

RES Yeah, yeah. And then after that, I came to England, where I'd always lived. And then

RES2 since 92,

RES since 92, and then I've sort of potted around for a while. And then I've got a job here in Reeth.

RES2 Sorry, REDACTED REDACTED has been in the UK since 92. We've been together since 92. We met in 91. in Cumbria, REDACTED came over, she did a master's degree at Lancaster

University in linguistics. And I think in the context of things, I think that's quite important as well. erm. She was then a teacher of English in a secondary school near Blackburn

RES Teaching English to English kids (laughs)

RES 2 Erm And then special needs classroom assistant, gardener, err horticultural tutor at a centre for adults with disabilities down in Surrey, err managing a team of staff 30 clients a day, etc. So I think that, that puts all the things in context or hope it does. Yeah

RES Because I was managing, I was managing other people what to do. And now if you ask me a question, I have to ask you twice, probably before I remember, what what, what, what is she actually asking me to do? So I have to remember these things. And it's just gone. And what did you say I often say and? Yeah.

INT1 What? So you're still so currently having like memory issues at the moment? Did the brain injury leave you with any other impairments at all.

RES Erm... As you can see, I'm wearing a patch (laughs). And it's because if I take my patch off, I can see two of you sitting there and two of you sitting there. And that's part of the role I had in the JCB I banged my head on the left hand side or whatever. And I also have something with my nose I can't smell everything. And and it's always runny. So yeah, lost, lost my kidney.

RES2 Lost a kidney and Titanium in the arm here.

RES Yeah.

RES 2 Can I just mention a few of the other problems, REDACTED's aware of them may not be able to hold them over. So all those things that I understand as executive functioning or the ability to to plan and to sequence erm... you know the memory to you know REDACTED knows all of her childhood, but we've been working in a garden this morning and REDACTED couldn't remember where she put the tools yesterday, etc, etc. You know 101 different memory issues. And another thing that I use as quite interesting so REDACTED used to be horticultural tutor manage clients manage staff managing 15 acre site. We have four vegetable beds REDACTED now struggles to organise and manage and plan three vegetables in four veggie beds. You know, it's that's sort of it erm, unable to see patterns and to determine what she requires, even though we have three or four veg may be required to try and sequence that during a four month season, damn nigh impossible.

RES It's the organisation of things. It doesn't happen in my head. as such Yeah.

INT1 Did the rehabilitation that you had in Holland, did that help it? Or did you find that beneficial?

RES You know, I can't remember a thing of that. But the only thing I can remember is there was a bike, erm.. exercise bike, and I loved going on the bike. But

RES2 So, so during that period of erm posttraumatic amnesia REDACTED couldn't remember where she'd been working, she couldn't remember where she lived. She couldn't understand why the staff was speaking Dutch

RES speaking Dutch?!

RES2 because by that point REDACTED was speaking English all the time. REDACTED couldn't remember any of the tutors on a day to day basis apart from the physio who would who would allow her to use the exercise bike on a daily basis, (chuckle) and that's an interest of REDACTED's. So I mean, it was a good place, but I think it was so early on in the recover, in the post accident recovery, that it's difficult to determine what what role is played now if you know what I mean,

RES I think that with a brain injury, you never know, what's going, what's going on in the in the head. So you never know what is going on. And, and even though they are trying their best in providing me with whatever, it didn't go in, or it didn't happen in me at that stage, because it was too early in that respect.

INT1 Yeah, I get that. And you haven't heard anything since then. Any sort of rehabilitation at all.

RES In, in

RES 2 so when REDACTED came back to the UK, which was probably err six months after the accident, by the time she'd spent time in medical facilities in Spain and in Holland, were then put under the supervision of South Lakeland acquired brain injury service in Kendall,

RES In Kendall. And they will good for or I can remember them.

RES 2 So REDACTED had a brief period with a physio she had time with the speech and language therapist and an occupational therapist.

RES And they were, I'm saying they were good that they were doing their work, but I can remember certain things because that was later in my life as such. So I remember certain bits there more so than I did in Holland, I can't remember anything from Holland, for instance, even though they tried their best.

INT1 Yeah,

RES I can't remember a thing, but in Kendall, Yes, I remember certain things

INT1 soon after the accident. I've just got to pause for two seconds. If you don't mind, if I make you the main screen, just because I'm only getting half of either of your faces. Is that?

RES 2 Is that better?

INT1 No, you've All right, I'll make it up on the spotlight. And I think that will work. Brilliant. Okay, so I can I can hopefully see you both now. erm So how have you found erm? Did you did you find that the rehabilitation here? And you can sort of remember, do you find, did you get any benefits from it? That you can, that you know of?

RES from from Kendall. I probably I can remember a lot. I probably have learned a lot but I can't remember what I learned.

INT1 Okay,

RES because those are the things that I would usually do go shopping, go biking, go walking, go take the bus to whatever, I would usually do that anyway. But they taught me that they get all this sort of taught me that again. I remember that but nothing, nothing.

INT1 So you

RES I just remember they were I just remember they were so kind and so nice. And yeah. I remember that.

RES 2 I think a couple of them were quite outdoorsy. Yes. I think REDACTED identified, REDACTED identified with that very much, just as she did with physio and the bike machine and err (laughs) (unclear 09.37) yeah

INT1 Okay, so if we can em think about lockdown and the last sort of, what are we now, 12 to 15 months ish. Can you tell me about the people that you might have seen on a regular basis or anybody that's visited erm

RES I can remember err that. I don't see anybody from my work. But I remember seeing or I remember seeing Sue and Nick, who, every weekend, they are here nearly every weekend. And nearly every weekend we go cycling, and we go running and we go walking together. And other than that, I can't remember anybody or anything anymore, or anybody else. But

RES2 Sorry I'll try not to interject, but I feel a need to. So

INT1 no, go for it.

RES 2 I think most of the friendships, that REDACTED has are the friendships as couple to couple friendships. For me, the big issue is, given that we're tied at the hip, and we spend, you know, 95% of life together, it's, it's the ability to have one on one friendships. That is the biggest deficit. So very few people who would have known REDACTED, prior to the accident, are still in the equation at all. And REDACTED's ability to make new friendships if, if you can't remember faces on a day to day basis, or if you can't remember

RES names

RES 2 your colleagues names. And indeed, if conversation is difficult, how willing are other people to actually pick up the reins and run with it and run with the relationship if REDACTED can no longer do that. And that, for me is the biggest issue. It's, I would love REDACTED to have a life and friendships away from me. And for me, that's the biggest issue. And I think it is for REDACTED as well. Her ability to have those one on one relationships to establish them to maintain and then develop those relationships is so much more difficult now.

INT1 **So the friendship groups that you have that you've been that you go cycling with and things, obviously during lockdown, where have you still been able to get out in cycle? Is that something you've done? still quite regularly?**

RES On my own.

INT1 **yeah.**

RES Yeah. And in that respect, fact, we living here in a very nice area, you can still walk, run cycle without seeing anybody.

RES 2 *But Nick and Sue weren't coming up cycling?*

RES No, because they they were, they are living somewhere else. And they have their second home here in Reeth. So they only came here every weekend. But not during lockdown of course, they had stay where they live.

INT1 **And were you keeping up any communication with them via sort of like a video call like this or anything like that**

RES Just email every now and then, yeah Nothing, nothing big as such.

INT1 **And what about the changing sort of your routine? If you think about what you were doing, and obviously you're going to work before the pandemic to what you've been doing during lockdown. How has that changed?**

RES Erm I've not, I've not cycled to and from my work and done my work. erm which is... which is a big thing for me, because it's not wonderful work and fantastic job. But at least it is something that I was doing. And that's gone now as well.

RES 2 *And you were meeting people too.*

RES And I was meeting people. Yeah. And especially there, I met quite a few people because it's an an outdoor education centre. So a lot of schools come in to do canoeing, hiking, or walking or whatever. So you'd meet a lot of people and I always I was a cleaner, but I always

met people and and I and I lost it in a way and (sigh) yeah. And I feel I'm not doing a wonderful job there. But at least I did do something.

INT1 **And yeah, I can understand that. It was a bit of independence for you and**

RES yes

INT1 **Something for you. Yeah, I can get that totally. And so have you managed to erm so obviously you mentioned about sort of gardening and horticulture and things like that. Have you managed to keep busy with that at all whilst we've been in lockdown has that increased at all**

RES not increased but um, looking after a holiday home, garden and a holiday home. So I do the cleaning and the garden in that holiday home

RES 2 *Maybe once a month*

RES Once a months yeah and that. So that's but and yeah, that's it really, really?

INT1 **And do you feel that whilst we've been in lockdown that you would... erm.... you would have liked to have done more? Have you felt restricted? I suppose is what I'm trying to find out. Have you felt bored? Maybe?**

RES Not not bored. But yes, restricted. So I would like to go to my work, but I can't, I'd like to do a bit more gardening here, there and anywhere but I can't sort of thing you know, it's this feeling of not not able to do what you want to do.

INT1 **And... Have you started anything new or anything during lockdown that you weren't doing before that you are doing now? Because of us been so restricted?**

RES No, no I don't know. I don't feel I've done other things now, or

RES2 *Sorry can I sorry, can I chip in*

INT1 Of course you can.

RES 2 I resigned from my job of nine years. The previous 11 months have been stacked virtually in my living room, you know, on teams call every morning, etc, etc. One of the reasons for me leaving that work was was because I'd rather be here doing things with REDACTED, knowing that she doesn't have anything to do, then be going out and looking for a job having stayed in that job. Because REDACTED has always been such such an active person that as long as she had a couple of part time regular jobs, which she did in the past cleaning jobs one in a hotel one in an outdoor centre, I felt she had enough to hang the rest of the week around. Whereas since March last year, she's not. And now one of the reasons I am not actively seeking work is because for the time being at least, I would rather be doing things with REDACTED, on a daily basis than actually feeling crappy about going to work. And knowing that REDACTED that active and generally motivated, as she used to do stuff hasn't, hasn't got that and REDACTED's ability to go out and ask for garden work which she could have done previously, REDACTED doesn't have that ability, and even doesn't even to convince somebody else that she can do the work is so much more difficult. So, so we're both off, and we try and we try and keep busy. The thought of me going going and doing another job without REDACTED doing doing something would make me feel pretty damn. Awful, actually.

INT1 And I don't know whether how to put this, would you say that? Actually, you're not being at work and being able to spend more time together? Is that a positive that's come out of this? Would you say

RES2 Don't know how to answer that, you answer first,

RES in a way, I'd love Laurence to do a job and working as such. But on the other hand, I want him to be happy with what he's doing. And if he's not happy with coming back home and see me on my own then ahh

RES2 there is an upside to us doing things together. As I say we've always been joined at the hip before or after the accident, but erm... it's now we're doing things but there's a lack of purpose and objective which I think most most of us need whether that's in work or other stuff

INT1 I feel like at the minute you feel, do you feel a bit like you're in limbo land we're just waiting for lockdown to lift so we can get back to work and get back to those lives that you were you're enjoying previously.

RES Yeah.

RES 2 *The other reason so the only reason to live in limbo and it may be stretching stretching your research, we are within within an area in the Yorkshire Dales where there's so little opportunities that we're seriously looking whether we need to move to a more populated area. Back to a couple of places in Cumbria for instance where if REDACTED's work now is limited to cleaning. And not that we have an issue with our because REDACTED both of us were cleaners back back in our other life anyway. But in somewhere like Keswick or Windermere, where there are lots of hospitality jobs, where we're working on the premise that REDACTED can probably get work in those places. Whereas here there is, there is so little work. And even if the residential centre opens up for residential groups, of which the cleaning might start again, it might still only be six hours a week. So we're we're in a real dilemma as to whether we move from this lovely location, to an area where REDACTED's chances of purposeful occupation may be far greater than they are here. And then I can look for work once and our REDACTED has got something.*

INT1 **Okay. What about the erm sort of the community and support from the local area where you're currently living at the moment? Do you, Is there a sense of community at all? Is it sort of a small village? Or are you quite literally in the middle of nowhere?**

RES It's a small village. Yes. But there's, I don't know anybody as such, I know, the local shop owner. And that's about it, really. And the person who lives opposite me and that's about it.

RES 2 *So a lot of people will probably recognise REDACTED and may also say hello, but REDACTED may not almost almost always immediately recognise or remember the person who's saying hello. And therefore it is difficult for that before even that passing relationship to develop.*

INT1 **Would that be something that you would like REDACTED? So if you were in sort of Kendall or Windermere where there was a lot more things going on? Would you like to join some, even some support groups or some think besides work where you were seeing regular people possibly every week? Would that be something that you think you'd be able to manage?**

RES I don't know about that…. no

INT1 **No?.... erm So if we think about erm the term loneliness, how would you define that?**

RES Yeah, well, when Lawrence was still working, I did feel loneliness... But... yes, I do feel it sometimes still but.... And another thing is that, in the past, I used to do running with a

running club, so we would meet every week and run whatever. And that's something else that I'm missing in a way.

INT1 Is it the exercise and the challenge of the run that you're missing or the social connections.

RES It's probably the first one the exercise but and that's what's nice about this place is that if you go running here, you can run for hours and not see a soul. It's lovely, however, yes.

RES 2 I think in the past, for instance, I mean, REDACTED, there was a really good fell runner. There's a plaque on the wall. REDACTED did the Bob Graham round in the Lake District, which is 42 peaks in 24 hours, etc etc. And, and REDACTED used to do mountain marathons with either with another female or with another chap, (laughter) REDACTED now can't run at anywhere near the level that she did. Partly cognitive reasons, but partly because of the eye

RES I can't see everything's

RES2 So so REDACTED's ability to be independent. err REDACTED can drive now for instance, and especially in a rural area that's very limiting. So those things that REDACTED would have been able to do off her own bat without me getting involved in and be and take the initiative in in arranging something with somebody else. That that is almost that that's almost gone gone completely, that that's ability to have friendships in that way.

INT1 Do you feel and you seem to have a really close relationship with with Lawrence, if when you have been feeling lonely, has it been something that you found easy to talk to or talk about something

RES that it's it's not something I feel I have to say anything like, “oh, Lawrence, I feel lonely today”. Because err Lawrence knows that if I'm on my own all day long then he sort of” what have you done today?” “Well, not much, really”. And then I know that he knows that I've been lonely as such.

INT1 You’re quite intuitive, and it was Yeah.

RES Yeah.

INT1 Do you think because of lockdown, and obviously you're not working at the moment? Has that changed? how you've thought about loneliness?

RES (sigh) No, I don't think so. But I do feel I am lonely. So there must be some so many other people around here who are lonely as well. But that's about it. And maybe

INT1 Yeah, I think erm lockdown has been very hard on a lot of people. And yeah, and not even people living by themselves.

RES No. And, and also thing are people who are elderly people who live on their own or whatever. They must be so lonely. But yeah.

INT1 And I think I already know the answer, but I'll ask anyway, how you feeling about lockdown lifting?

RES Oh, yeah. I mean, that's good in a way. And another thing that I sort of miss at the moment is, working during the week and then on Friday, go for a drink and a pub meal somewhere. So the end of the week thing? Yeah. And then we can do some exercise. You know?

RES 2 You don't get that Friday feeling so much do if you haven't been, you know, if you haven't been hard, hard at it all week. Yeah.

INT1 Definitely not. Yeah, it's not quite the same, is it Fridays at the moment? But you're right anyway, doing nothing. Yeah, I get that. And so in terms of sort of the virus are you concerned in terms of any health reasons or anything for getting out and socialising? I know, there's not a lot of people where you are. But is that a concern for you at all?

RES At the moment, not because I'm always away from people or search as if you walk or run, you always go round people. And if you go and drink, have a drink at the pub, and make sure you put your mask on and stay away from people so i i do agree with that. Or I do understand and I do agree with that. But yeah, I'd love I'd love to just walk as normal and sit at the pub as normal.

RES2 But You're not you're not overly anxious about it at all

RES oh no no no no it's just as it is.

INT1 **And whilst we've been in lockdown, have you managed because exercise and obviously you can see pictures behind you you really like to challenge yourself? Has, have you been, have you felt like you've been able to do as much as you would like in terms of exercise?**

RES Oh, yes. I've maybe. Maybe I've done more so than if I just worked normal somewhere. you know... yeah

INT1 **And have you found that a positive thing? Have you enjoyed that?**

RES In a way Yes. Because I always feel nicer, better when I come back from a run or come back for a bike ride. Yeah

RES 2 *Something that's dropped off is since I stopped work, REDACTED is no longer, no longer going over grinton mortar in layburn to pick up the shopping in a rucksack. So she probably hasn't had as many hill miles of late*

INT1 **With a heavy rucksack with all the weeks shopping Yeah. Okay, so in that respect, then what would you say has been the most helpful thing for you throughout lockdown. If there has been anything**

RES .....I don't feel any helpful things with lockdown,

RES2 *During lockdown you've always said how lucky it is that you lived in this area. Yeah, well it can still go out. do exercise or yeah*

RES Yeah, I've always said, yeah. Especially cycling around here is wonderful in a way. Because there's not many cars around anyway, but around this area. erm... And that I guess, and then you get the lock down. It's got less cars around. So it's Cycling is wonderful around here. But yeah.

INT1 **What then, in reverse has been the most difficult for you**

RES errm no, work. Well. If you go shopping, you have to put your mask on. And there's only so many people allowed in the shop as such and but everybody has that problem as such. you know, i's not just me. Yeah, no work. That's it really, yeah

INT1 what do you think the first thing you'll be looking to do? When lock down? Does lift? .

RES err. To get back to the work?

INT1 Yeah. I can understand

RES And it's not so much the shopping. Because even though you sometimes have to wait before you can go into the shop, etc. Everybody is doing the same thing. So. But yeah, it's for me it's work really? To do something.

INT1 Yeah, I can tell you, you've really missed that independence that work gave you?

RES Yeah.

INT1 And yeah.

RES And even though it's not wonderful job, at least that something? Yeah.

INT1 And if you find that you've struggled at all, in terms of motivation at the moment, because you haven't got much to do. Are you able to motivate yourselves as much as you were?

RES Ahm I, I always write a little list of things I have to do that particular day. And then once I scrub everything off the listing, yes, I do whatever I needed to do that day. So the motivation in that respect is still there. But

RES 2 there are days when you don't hav,e where you don't see yourself as having a particular objective. I think you feel it.

RES Yeah.... yeah

INT1 Yeah. So if there isn't anything particularly pertinent on that list those days that you're struggling a little bit more,

RES yeah.

INT1 Okay. I can see that. Sam, do you have any more questions before I move on to the scales?

INT2 There's one or two things I have thought or stood out and I'm not really sure how to word the first one. It's from you speaking about loneliness. And you spoke about if you've been at home alone, during the day and Lawrence will come in, he might like just instinctively pick up that you've been feeling lonely? What do you think about like, how kind of the connection between being alone and being lonely?

RES I'm not quite sure what you mean by that. L

INT2 Like, as an example, like some people are okay with being alone and not being lonely. So do you feel like there's more of a connection between being alone and loneliness for you?

RES Yeah err Yeah.

RES2 So I think if you if you felt you had a purpose, you'd been asked to dig a hole which you'd love to do, or go and mow that lawn. You will be on your own. But I don't think you'd feel lonely

RES No. Because then at least I feel I'm doing something purposeful. Whether I'm on my own or 16 people around me chatting away to me, it doesn't really matter in that respect. But I feel I have to do something. If that's

RES2 so if you were alone and didn't have something to do..?

RES Then I feel lonely as such. Yeah. Yeah.

INT2 (unclear 34.34) trying to get at

INT1 it's more the purpose that gives you a sense of belonging If you like, to have something to do prevents you from feeling isolated.

RES Yeah

INT1 Was there another question Sam?

INT2 Err I'll just have a quick scroll think that was everything. Yeah.

RES Thank you.

Interviewer(s): **INT1**

INT2

Respondent(s): RES (P17)

INT1: Excellent. So can I just get a little bit of personal information about yourself? Can I just ask how old you are?

RES: I'm 53 years old.

INT1: And what would your marital status be?

RES: I'm single now, yeah, single.

INT1: Yeah. What about your current living arrangements? Who are you currently living with?

RES: I live on my own.

INT1: And do you have a primary caregiver at all?

RES: No.

INT1: And which region of the UK do you live in?

RES: I live in [location].

INT1: And what would your employment status be classed as?

RES: I'm a voluntary worker, I'm- I- I live on benefits. So disability, but I volunteer, you know?

INT1: Yep. Brilliant. So can you star- would you mind just telling us a little bit about your brain injury?

RES: Yeah, well, for me, personally, I had what- what was known as a minor head injury, it's well over, like, well, over 20 years ago, in fact, you know, but erm... it was due to the result of a serious assaul- up in Scotland it's called a serious assault, I think in England it's called GBH-

you know an attack, I was attacked you know like a serious attack, the perpetrators went to jail and all that, I had to go as a witness to the court. But it left me with some damage, you know, permanent, you know, which affects my life. But, I'm- I can operate- I'm one of these people actually, probably in a category of presents well, you know, I've got underlying difficulties. No, the kind of invisible illness of head injury type of thing, you know?

INT1: Sorry I broke up a little bit, did you say, erm, how much- were you talking about what is- how it's affected your, your daily life, for instance, sort of what impairments has it left you with?

RES: A lot of the problem I have is more, like, when I'm under stress, you know, it's my relationships with people, you know. It's always hard to talk, but you don't want to give an impression of being a- a bad person [laughs] or something like that. Under stress, things like- things that irritate me, I find that hard to control my emotions with things that irritate me, if I'm on a bus, you know, and some- someone's like playing loud music behind me, you know, most people, it would irritate them. If they didn't have a head injury, they would just probably keep theit mouth shut [laughs] as much as they could. But I lose control of the ability to reign myself in and I don't- I'm not violent at all, but I've got myself into quite a lot of arguments and scrapes with people over the years. And that's what makes it quite difficult for me to work full time because I end up not showing enough respect to bosses and all that, arguing with them and things like that, you know.

INT1: And, erm, after the attack, did you have erm- so were you in a coma, were you unconscious at- attack or- when you were attacked?

RES: I didn't lose consciousness to the point of the attack I went into an ambulance who took me to- I was with another friend who was attacked as well. They took us in an ambulance to the nearest hospital. And then I los- drifted out of consciousness in and out at that point, but not- I think the adrenaline kept me going until- I had to get to the police station to- to- alert- I managed to escape after I've been hit and I'd got to a police station to alert them. My friend was still being attacked by three people do you know what I mean, with weapons, you know. So I think the adrenaline kept me going until I was able to get you know, get into a police cell and then I think I started drifting off, you know.

INT1: And at the time did- did you realise that you'd- you would be left with a brain injury or did you just think sort of it was a bit of a head injury and you'd recover and there was no rehabilitation or anything like that?

RES: It was like erm, I think that things have [inaudible] a lot in- over these 20 odd years I did have follow-on treatment, but it was never to do with the cognitive thing. It was more to do with like physical like the- is the fracture healing? And all that type of stuff and I would get all sorts of x-rays. I had to go with my- I was still young at the time, in my mid 20s or whatever, I would go with my dad up to hospital in [location], I didn'- I came from [location],

I didn't come from [location] so he would run me up in the car and I would go for all these scans and things but nobody told me about the effects of the cognitive stuff or any of that, not at all, no.

INT1: And then if we have a... a think about lockdown in sort of the last 12 to 15 months or so since the pandemic started, can you tell me a little bit about the people that you've seen on a regular basis that have been helping you that've visited you or you may have visited them?

RES: Well, I was actually, I mean- I was already in quite a lonely situation to be honest with you because I mean I've been- I was in a long-term relationship with- you know a partner for seven or eight years, you know, but that ended a good few years ago. And erm... my family- my mother and father are both dead. I had a young brother that died years ago, you know, I don't have children myself. I have nephews and nieces, but I don't really see them. So my position was already quite lonely, and then obviously, when the virus struck, you know, and the regulations came in, that was- it was obviously harder, it made me, erm- because I'm a member of Headway, [location] you know that's how I know about this interview and they've got a place that's open you know every day of the week in the centre of [location] and you can always go up there and chat to people, but that's been shut for a year and a half now, you know.

INT1: And have they taken on any sort of virtual events have you been doing sort of Zoom calls or anything like that instead?

RES: Yeah, yeah, that- that's been a bit of a lifesaver for someone like myself really, erm... it's- the- what they did it took them a couple of months to get it up and running as you can imagine, what with all the- I mean, it hit everybody like a thunderbolt out the blue as you know, and erm- but eventually after a couple months they had all the classes running by Zoom. So, I generally go in twice a week, I mean the sessions last for about a good couple hours, two, two and a half hours and it's just a drop in group you know, I don't go to the art classes or the music or any of that I just wanted to drop in chat things. But it's a real lifesaver for me definitely. It's been a it's been a great thing, you know.

INT1: Brilliant. And did you say you were doing some volunteering as well? Have you been able to do any of that whilst we've been in the lockdown?

RES: No, I had- I've had- I was actually a volunteer at Headway right before I was a client strangely enough so I- Headway shut down so that- I do my bit for them, helping them out with quizzes and all that stuff. But I'm not an official volunteer, I was a volunteer tour guide at [exhibition centre] in [location], you know, one of the big tourist places for a good couple of years, but that stopped after the craic with COVID because there's no visitors coming you know, noand it was shut [laughs] shut anyway you know?

INT1: Yeah. So before lockdown would technology sort of like Zoom would that have been something you would have ever used to keep in contact with friends or anybody else before?

RES: I had never used Zoom before, I mean, I'm usually I'm on texting and all that stuff, you know, and emails, but I never used Zoom at all before the lockdown.

INT1: And what about social media? Do you use any of that to keep in contact with anybody?

RES: And I don't really- I- in the recent years have started following Twitter but I don't tweet on it because you know, I'll be scared of getting arrested [laughs] I'm too opinionated [both laugh] I like to follow politics and all that, so I've realised in the last couple years by following people on Twitter you can learn a lot on that you know, but I don't tweet myself, you know?

INT1: No, sometimes it's just quite amusing to read all the other comments isn't it?

RES: Yeah.

INT1: So would you say, erm, Headway before the pandemic was the Headway your main social gathering- your- your main way of connecting with people would you have said?

RES: Yeah, it was probably- it was one of the major ones I mean, I do have friends and things outside of Headway, I don't have a lot of family but I have got a few friend and- about [location] but yeah, definitely one of the major the major blocks yeah, yeah.

INT1: And have you managed to keep in touch with your friends whilst we've been in lockdown?

RES: Yeah, but not- not so much meeting- well not so much I've got friends like outside of Headway, who I really know from the area, of [location] I live in. I've known them for years, right, but haven't really seen much of them, because of the restrictions and all that you know, you weren't allowed to meet up and all that. But I've got a friend from Headway, my pal, [name], and we're all part of the Headway walking group. And as soon as the restrictions are lifted enough, we- we always start off and- unofficially with the walking group. So we're back- me and my friend, [name], go out on a Friday and it's unofficial- it isn't- Headway can't authorise it, you know. But anybody- we put it out, if anybody wants to come and join us, they can come round and we do the walk. So quite a few do from time to time.

INT1: Brilliant. Have you found that that's helped getting out and going for a walk? Do you go far?

RES: Yeah, I'm actually- I mean, I've actually got quite a few physical impairments but I used to be actually very sporty, athletic, in my young days, you know, quite a high level, so, even though I'm getting on a bit now, I mean I've got arthritis and things but walking actually helps that to keep the joints moving if you know what I mean. So- yea- the answer is aye I do- I do fin- even during the lockdown [inaudible] I was just in myself I had get out the house, you know, so- always planned it for like- even in the strongest lockdown, I would go out and walk for four or five miles and do my shopping and come back, you know?

INT1: And Was that something you were doing before the lockdown as well? It's just that perhaps now you were doing less of it or more of it because of lockdown?

RES: I mean, I do a lot of- a lot of walking anyway, I've always been a person that liked walking, you know? And because I can't do it- I used to be into lots of weights and Judo, squash, all that. I can't really do them now, because my age and because of my ailments. But I can obviously walk. So walking was always a big thing for me anyway. And also I did a bit of swimming in the health club but the health club shut down again for a year and a half as well.

INT1: So currently, what- what kinds of things are you doing at the moment to keep yourself busy whilst everything's been so restricted? How are you- what's your- an average day look like?

RES: Yeah- I mean- I don't rea- I get through the days quite well just cause I'm pretty much a person of routine if you know what I mean. And... I mean, most days I wake up at seven o'clock you know I put on the radio, I have a cup of tea I kinda don't get up till about nine cause there's not any [laughs] any major reason to be up. But then I get up, I watch a bit of TV, do a bit of reading, any jobs that need done round the house because I'm the only person that can do them, you know, hoovering, washing, whatever. I do that in the mornings, a bit of stretching because I'm getting a bit stiff now in my old age [laughs], a bit of stretching... I'd usually plan to go about half past one. Once I get to after one o'clock I start to get a bit agitated or needing to get out. Now that the restrictions have lessened off a lot I find myself doing a lot more like now I'm getting back into [location] city centre- I'm a big reader, so I like to go to Waterstones in [location] city centre. It's the big- it's the biggest bookshop in the whole of [location], you know, and they've got cafe in it, and so I get a couple of books and read the books and that passes a lot of time away, so, I don't want to go on too much sorry.

INT1: No, no, that's perfect. So when you go to Waterstones and yet you sit in the cafe do you like to sort of- do feel good just being surrounded by people even if you're not necessarily talking to them? Do you think that- that helps as well?

RES: Yeah, that's true, although I do- actually because I'm quite a regular in there I know a lot of the staff and all that anyway they know me quite well so I always have- I don't stand having big conversations- but it'll be a bit of a- an interchange and erm, I missed it really really badly during the severe lockdowns really badly, you know, so the answer is yes to that.

INT1: Yeah. And it's that very different to how life was before lockdown?

RES: Erm- what do you mean during the seriou- severe lockdown? Before lock- before the lockdown I spent most of my time- I would be in Headway always on a Monday afternoon for the drop in. I would do Headway walking group on a Friday, which takes up the whole Friday afternoon. Erm- during the Tuesday, Wednesday, and Thursday I would be like that going to Waterstones, I've got another cafe- I live in the West side of [location]- I've got another cafe up [location] area where they know me as well, if I can't be walking into town. So I do all my own shopping obviously because I live alone so I've always got lots of things to keep you busy like that. But the lockdown obviously when you're only allowed to go for- supposedly- I was going for a couple hours a day, but whatever it was you know- that was really- I got used to it, but it was hard at first, you know?

INT1: Yeah, definitely. So how would you define the term loneliness?

RES: I don't know. I've actually suffered at various times, I had never really experienced loneliness until I was about the 30 or 31 and I moved back to [location] again. I was a student here when I was young, a physical education student, [college], I moved back to [location] and a worked in a sports centre and all that. And then I come back up here, I lost my job because of the problems I've told you about, you know, I wasn't getting on with the manager and I was arguing- things I would've never done normally before that. I was you know what I mean, you know what I'm saying, so like er- I lost my job, I had an industrial tribunal all that stuff. Had severe depression, moved up to [location], and that was my first real experience of loneliness, because in my hometown, I knew lots of people down in [location] it's a small town where everybody knows everyone so I could really be lonely there I could go down the pub and I knew guys I'd been to school with or in the sport centre. I would de- I would describe it as like living in a vacuum, that's what always felt like to me as if your life's got no context, you know.

INT1: Great, that's a really- a great expression for it. And do you think you've felt lonely recently?

RES: I felt more lonely if I go for more than a couple of days. I can put up with a day or two on my own totally you know without, you know, having any real conversation, but after that, it starts to hit me quite hard with anxiety and stuff like that, you know? Erm, sorry, I forgot the actual question, sorry, would you say that again?

INT1: Just whether you'd felt it recently- whether you've felt lonely recently?

RES: Yeah, it's been actually a bit better since the restrictions have been lifted, obviously, because I'm out and about a bit more, but erm, mor- it was more during the actual severe lockdowns they were definitely worse but I would like to just mention before I forget, there's are women as well, from an organisation called REDACTED, I don't know if you've heard of that. And then- I... I'm a member of [location] Disability Alliance as well. And I actually spoke to them at the start of the lockdown. And I told them "look I'm living on my own here and all that and I'm quite worried about this" so you know, I don't know what was- just mentioned it- what they did was that was they contacted this organisation, they're actually based in Manchester, you know, but for over a year, they've been phoning me up once a week, you know, just to chat for half an hour or so like, that's actually helped. Because sometimes during the severe lockdown, I was only really getting the two Zoom conferences, which was Monday and Friday. So the midweek was pretty hard, so she phones on a Thursday, which actually helped a bit as well, you know.

INT1: And do you find because it's that one-to-one telephone conversation as well, it's- it's slightly different to the Zoom, where you've got a lot of people talking, and sometimes they can be talking over each other and it's hard to always have a conversation that way? Did you find that the- the telephone call was better for that?

RES: It's a different type of thing. I mean, I wasn't too bad during the Zooms because I'm probably one of the [laughs] loudest people there, you know, I'm actually pret- I mean, I'm a talker anyway. So I get- I- you know, I'm probably one of the people that does talk a lot on it. But I always try to be respectful of other people, you know, coming in, I dont't to sound as if I'm trying to push anyone out the way. But, erm, it's a different type of conversation, you know, and you maybe be talk about more personal things and all that, whereas you know on Zoom it's like a kinda- it's like sitting in a drop in, you know, you've banter in general things, you know.

INT1: Yeah, definitely. Do you feel when you have experienced feelings of loneliness, do you feel that you have people around you that you could talk about it with?

RES: Nah not- and that's the problem for me, really, it's not- not really know it's erm, and also, as well knowing- as I think it is different between men and women in that regard, you know I've got mates and I don't expect them- because I know- I know they're sick- I know what the score is if you like, you know, I mean, you talk about football and talk about that stuff like that, but you don't really go into personal feelings. So I- there wasn't a lot of an outlet for that, biggest outlet would be that- the woman that phones me from Manchester really on a Wednesday, and that would be if I had anything like that, to speak about, that- there wasn't a great outlet for it to answer your question.

INT1: And did you feel comfortable enough talking to the lady that was ringing you about how you were feeling? Or again... would you- would you keep that to yourself?

RES: Erm, I mean, it doesn't go as deep as like a counselling session or that-

INT1: No no.

RES: -no I know you know that but what I'm trying to say is it's kind of like a- between a general conversation and a counselling session it's probably like halfway there do you know what I mean, like-

INT1: Yeah.

RES: -I don't know her well enough I would really want to- and I don't think that's what they're there for anyway, I think they're there to- to... have a general chat and- but you can obviously touch and stuff like that, you know, so that's probably the best I can an- yeah, I'm quite fine with them they're quite- they're decent people I mean it's quite good they volunteer their time to do things like that and all that, you know so. But yeah they're alright I'm quite happy with them.

INT1: Good, and I imagine as well- yourself you've got the Headway groups as well but there will be people that will literally just have that, that telephone call once a week and will go the rest of the week probably with speaking to very little people so, so it is a really valuable service definitely. Do you think lockdown has made you think differently about loneliness at all?

RES: I find, you know, in a strange sort of way it made you feel little less isolated and loneliness because every time you turned on the radio or television everyone was talking about how lonely they were and all that so in a way it made you feel you were kinda part of something a bit more in a strange sort of way, you know. But on the other hand when they took the lockdown and people going to hug their grannies and their- all that stuff and their sisters, it's started feeling lonely again, because I don't actually have that way. I've actually got loads and loads of cousin's down in the hometown right in [location]... my mum had five sisters, you know, and they all had kids and all that, and so I've got loads of cousins and they've all got kids now as well, you know, and my brother's son and all that down there with his family. But I'm not really in touch with them the- the problem is- the anti-social problems caused by head injury isolated me from a lot people I- did lose the plot I've got to be totally honest with you, you know and I plunged into a lot of drinking, and... you know just being a real pain to a lot of people and I couldn't really control it, really, to be honest, so I destroyed a lot of relationships with people down if you know what I mean- not by violence or anything but just by general anti-social- you know, you know what I mean yeah.

INT1: Yeah, I can understand that. So... what- what- how are you feeling about- sort of society opening up again? So I think in Scotland have you had a bit of easing anyway? Are you similar to us in a few weeks she's looking to open it up further?

RES: Yeah, I don't really know, it's hard to know what's going on because we were definitely opening up there as you know all the way- listen I mean, what they done in Scotland I think- I mean, without going into politics, it's just a cat and mouse game, you know, like, there's not that much difference if- if Boris REDACTEDson does something that, you know, they'll do it here two weeks later and if he thinks it's- you know, it's just- it's pretty similar. The only difference is the timing, you know, you might get something a week or two before we get it but... [location] itself has been under very severe restriction it was nine months, we just came out last week to level two- nine months in lockdown, no pubs open or anything. So opening up is- I would say it's good for me personally, because I can get out and about and talk to people a lot more. One Tuesday I got to my health club, it's like a private gym place you know in the- in the hotel and it's the first time I've managed to get in since the lockdown so I had a swim and I sauna, a steam room, all that it's absolutely brilliant you know, so, when I go to things like that because I'm a natural talker, I talk to people, you know, always talk to people. I suppose for me, it's the close- the really close relationships, I don't really have you know, I mean.

INT1: Yeah.

RES: But I've had that in the past, you know.

INT1: Okay. You seem really sociable and I can imagine from what you're saying that you'll sit and chat to anybody and put a smile on people's faces as you as you pass along. What do you think one of the first things you'll be looking forward to doing once everything eases what you- what you're looking forward to?

RES: Well now that the health club- I would have said to you last week I was really looking forward to getting down for a swing and a sauna you know, but now that that's opened up again I've done it, I was- another thing I do quite a lot is that I really love travel, you know- because I'm on benefits I don't have huge sums of money but I do a lot of travelling, even down to Carlisle but mostly in Scotland and maybe the North of England, so I started that off again I was up at [location] walked in [location] a couple weeks ago, I've been in [location] a couple of times and, you know, so I like stuff like that, that's really good for me as well. The only thing that's left now is like for Headway drop-in to open physically and get back down there, you know.

INT1: And do you think anything- what would you say has helped you through, especially this- the really strict lockdown those- those nine months, what do you think has helped you you through it?

RES: Well the most of- the things have helped me most have been the Zoom conferences with Headway, the REDACTED thing in Manchester I was telling you about, and on the weekend my mate, [name] who's just one of my [location] mates you know nothing to do with Headway or anything, he texts me a lot of football and things like that, so usually at the weekend even though I'm not speaking to him I'm getting getting a lot of texts going back and forward and all that and emails and about football. I should say to you earlier on as well I'm missing football stadiums opening up and, er- I don't drink but I go to the pub to watch football, I'm missing that as well you know? [Laughs].

INT1: Are you excited for the Euro Cup starting tomorrow?

RES: I think I'm quite excited- I'd like a lot more if tthere was lots of fans in it- I think the Scotland-England game at Wembley would have been a real cracker with fans.

INT1: Oh yeah, gosh could you imagine?

RES: I'm more of a club fan- I'll support Scotland like- I don't go to a lot of Scotland games but you can imagine the Scotland-England game getting everybody up you know geed up and all that you know? [Laughs]

INT1: Definitely, especially after a few months in lockdown as well. What do you think has been the most difficult thing for you?

RES: I just think the hardest thing was during the very first lockdown, the severe lockdown I fou- you know when you were only really going out- you had to focus your whole day on just your walk- the walk was the thing of the day you know the- the big event of the day and then the thing I found out was a sheer restriction of that like you couldn't even go on a train, I couldn't go down- I regularly still go down to my home town in [location]. You know, it's a seaside town and it's a nice place. I go down every few weeks, I couldn't do that, I couldn't go any football there was just nothing, that- that was- the first lockdown was the hardest thing, just the sheer hardness of the restrictions, you know.

INT1: Do you think that, the sort of shock to the system as well made it difficult? Because it was such a- all of a sudden everything stopping, there wasn't a gradual process?

RES: Yeah, it was really incredible actually looking back, you know, I mean- being in that, er, the health club the other day, the- when I was down for a s- you know, I had this memory of- you know how your phone now tells you when you were last at this venue? You know, your smartphone and it said you were last here on, you know, February the 28th 2020 or something and then, I remember sitting in the sauna and it was two older guys who I new- I

didn't know them well, but I know- I knew their faces from the club you know and I heard them talking like typical [location] guys and they were saying "Coronavirus what a lot of nonsense that is it'll not stop me it'll not have any effect on our life" [laughs] if only we'd known back then but- and you know, it was only a week after like, you were on the TV and it was like "lockdown's, coming" and it just came down like a curtain, you know. I remember rushing about all the shops and trying to get as much food in as possible. And guess what? It's still sitting there [laughs] I've never used it- in a hamper. You know, I bought a box and put all these tins in as if I was preparing for a nuclear war, but they still managed to get food, you know.

INT1: Yeah, definitely. I think we were all a bit the same weren't we it was er- yeah, it was- came down like a tonne of bricks really didn't it? I just want to- to come back to what- a point that you made earlier sort of about when society is opening, and everybody else is sort of rushing off to those- hug their grannies and getting back into family and things. How did that how did that make you feel for yourself when you didn't have family close by?

RES: Yeah that- that- I don't really have... I mean, I've not really got family that I would be going back to see anyway, anywhere, close by or anywhere else, you know erm, so it does- the thing I was saying earlier that- that's probably one of the hardest things, the actual, the loneliness of the lockdown, in a way, as I said to you earlier it made you feel part of something because I see so many people are there. But it's the opposite when it starts to open up and you watch the news at night and you see folk going to hug their kids or hug their grannies and all that stuff. I- I won't have that situation. I'm not- I'm not- it's just one of these things, it's just the way it's worked out. I mean, I've had the long-term relationships and all that before, I've been an but it's just the- after the last one, I don't know, I think sometimes you get a bit older and I've- you've just been through the mill, and I just, I don't know I just can't be bothered [laughs] anymore, you know? And I kept fighting "if I was to meet somebody I'll meet them, but if I don't I would just live on my own", do you know what I mean, that's probably how I feel now, you know?

INT1: Yeah, it's a great philosophy to have as well, I think. [INT2], do you have any more questions before I move on to the scales?

INT2: Erm, not particularly this time. It's been an interesting, listen, I've met a lot of notes and most things have come up multiple times. So I don't think there's any further to try and dig into things.

[Progress through scales]

INT1: And you felt nauseous or had stomach problems when you thought about or was exposed to information about the Coronavirus?

RES: No, no, not at all. Can I just see something on that? You for myself personally, I've never actually feared the virus itself, you know, I don't why, maybes because I live on my own and I don- I wouldn't want to get it, right. But I've never been terrified of the actual viruses it's more the loneliness of living on my own during it bothered me if you know what I mean?

INT1: Yeah, yeah, totally. We found with this scale, particularly- I think we're on about, how many have we done now about nearly 30 participants, and we've had perhaps two people that have ticked into the rare box. And everybody else has said not at all. So you're you're not alone on that one. It's- it seems to be quite a common theme to- that people aren't really fearing it.

[Progress through scales]

INT1: And you often feel rejected?

RES: I would see more or less because I don't feel people actually reject me, but because my isolated possession I feel I'm outside of society sometimes, you know?

[Progress through scales]

INT1: And so how much of the time during the past week, would you say that you'd felt lonely? To be none or almost none; some of the time; most of the time; often; or all almost all?

RES: Probably between some of the time and most of the time, but I'll go for some of the time. Because- I want to say something with that, when live on your own and like I do, you do have quite a lot of coping mechanisms that probably gave you more endurance to it than most people have. So if I didn't have those would be probably often, but I'll go for some of the time because I've got coping things to do that help me, you know?

INT1: Yeah, definitely that you've sort of learnt over the- over the years. So the next one, again, we're thinking about just the last two weeks. And again, it's how often have you- have you been feeling these things? So the first one is you've been feeling optimistic about the future? Would that be in none of the time; rarely; some of the time; often; or all of the time?

RES: I am optimistic at the best I would say some of the time because I do find in the mornings I'm less optimistic, but once I've been out for my walk and all that and had my endorphins and all that stuff I feel better at night. So I'd say some of the times.

[Progress through scales]

INT1: Brilliant, and you've been thinking clearly?

RES: Yeah, I don- I don't think my mental state- I've suffered from anxiety, depression in the past, you know, but again, I- I stopped smoking and drinking a long time ago and that's always helped me with that so we'll say often for that.

[Finish scales, brief conversation wrapping up]

RES: I won't keep you all day, but I've just seen them in terms of- my friend, [name], that I go walking with on a Friday that I was telling you about, the unofficial walking group, he's a stroke- a person that's had a stroke, you know, and the thing you're talking about, loneliness, I was up in [location] City Centre just last week, and I saw my friend [name], who's also a Head- I mean, Headway [location]'s huge, you know, because it covers the whole city so we- [name]'s a friend of mine and I hadn't seen him for well over a year and a half and I was asking him "how are you coping, [name]?" He was a stroke guy as well, he used to work for a trade union or something and then he had a stroke in his late 40s and he- it's a really hard thing when you're that age you know, you're never gonna work again you know? Still compos mentis but he's just got the left side of the body thing, you know, the only problems with that. I met him and I talked to him in the street for about an hour, and he was- he's coping no problem, you know, like some people can- he is a guy that can go- I've even talked to him about spending Christmas alone because I've had to do that myself a few times and er- I talked to him about it, because I know he's in the same situation. But he's one of these guys that just seems to really fought through it he can handle it like no problem, in fact, he's happy with it. So everybody's different and things, I'm a kind of, middle of the road, you know, go long periods, but anyway, everybody's different.

Interviewer(s) **INT1**

INT2

Respondent: RES (P18)

INT1 If we can start, can I just ask you, erm if you wouldn't mind just sharing a little bit about your brain injury with us?

RES Yeah, so I erm.. God was it October 2018 now. So I… felt really dizzy err when I went for a shower erm it must have been about half, four in the morning because I was getting up for work. erm and I just felt really dizzy and when to step out but as I've stepped out, I've just completely gone erm so I hit here erm off the side of a radiator erm so I lost a lot a lot of blood, and got brought into resus and had some bleeding on the brain, erm which I'm kind of still recovering from stitches, loads of blood loss it was err, I was in hospital for about a week. Erm and then after that, I don't think I realised the seriousness of it, to be honest, until I went back to see the neurosurgeon err probably a month or two after erm he referred me to like a rehab clinic, erm through the NHS. So I've been gone to that I've just been discharged from that erm just because of resources and stuff moneys been cut, erm what I seen them occupational therapists and dietitians and stuff like that erm psychologists Is that what you call them did loads of test and and stuff. Erm… Did that for over a year and a half. So that was quite good. erm but still kind of feeling the effects of it, to be honest. But yeah, they don't really know what caused it, because I don't tend to faint or anything, or get dizzy, erm just a low blood pressure. So I just keep an eye on it now, erm that's it really not very exciting.

INT1 And what about sort of erm you mentioned, you're still having sort of ongoing issues and things. Can you tell me a little bit about how it has affected you your daily life?

RES Yeah, so (sigh) tiredness was the main thing for me. I mean, the first couple of weeks and months and stuff, I was just, I was sleeping constantly. And I mean, I went back to work two weeks after. And I really shouldn't have because I shouldn't have been driving or anything, the doctor signed me off for eight. erm But I just wanted to get back get back into routine, but it's probably the worst thing I did to be honest. erm Because I was just exhausted all the time. And even now I still like nap through the day and stuff, because I just be so so tired, and work are aware and everything's so they're fine, but it's just it's more of an annoyance than anything else, erm and then I did the test. And so I've had loads of memory issues. And so I did the test and with the rehab guys, and they sent like a formal letter and stuff, just with all the test results and that on it. So erm there was the memory, the tiredness, fatigue, and then some depression as well. So I actually went to a doctor, and they put me on antidepressants just on the back of the accident and everything that happened. And so that I'm still taking tablets for at the moment erm. But mainly tiredness memory is what is affected me day to day. I'm just saying stuff, I probably shouldn't sometimes just comes out. I can't help it (chuckle) erm But they're the kind of big big ones.

INT1 **And you mentioned that obviously, you've had quite a lot of psychological erm tests and things, did you have some rehabilitation for your memory problems at all, or you've been through some some rehab?**

RES Erm No, so we did the test erm just to kind of confirm it was there, erm that was then when I was seeing an occupational therapist. So she went through loads of stuff with me, she sent me once a week, and then it went to once every two weeks, she's to come to the house. But erm she used to give me loads of exercises and stuff to do and recommend loads. I've got lots of books and stuff that I can do little memory exercises in. erm just ways of managing it like writing everything down. So there's just notes everywhere at the moment, because I just don't remember anything (laughs). So yeah, erm just just learning the old things that will help make it easier.

INT1 **Yeah. And have you found that that has helped? Do you find the tips and things that she's she's given you have made a difference?**

RES Yeah, no, definitely. I think that the hardest part of it is getting because even if it's, I drive for a living. So a lot of my phone calls are on the phone and stuff. It's more explaining it to other people because I'm like, please send me an email on that. And they must just think I'm stupid, but I'm not explaining that to them all. So I'm like, please send me an email because I'll forget by the time I get home, so it's more difficult trying to explain it to other people than anything else... erm.. But yeah, definitely helps at home writing everything down and stuff It's just when i'm out and about. I kind of struggle with it. And I do forget stuff. It's gonna happen. So...

INT1 **Yeah..So if we think a little bit about lockdown, and the past sort of year to, to 18 months whilst we've been erm restricted. Can you tell me a little bit about your er support networks. If you've seen anybody er who may have helped you over this time**

RES Erm Last year when I was kind of in full swing, like COVID, and stuff, there was a period of like six months where I just didn't see anyone. Because at work, we weren't allowed to go out either. Erm so that was really difficult. So they worked. I lived on a building site. So the only people I was saying is looking at the builders during the day, whilst I was trying to work, you know, what I mean, it was just it was awful. So I barely seen anyone. Erm this year has been better, because we've been able to get out in shops and stuff and construction sites and kind of just see people and be around people and go work in offices if we can. But last year, last year was pretty difficult to be honest, until it eased off towards the back end of the year. Erm so just friends and stuff were coming right then partner. Erm ..but I think it was for that first six months, I don't think I think anyone really

INT1 **Do you mind if I ask what you do for a living.?**

RES Yeah, construction manager for Co Op.

INT1 **Okay. And did you say you were working from home for some of that? So you could transition fairly easily, say,**

RES For when COVID first started it was March wasn't it last year for the first four months, maybe it was just solid working from home because everything was just halted. But then construction started back open, err maybe June time last year. So we were able to start get stuff going again. And so I was out and about a bit more after that. Just go and well not I

usually am wait four or five days a week normally. But it's been like one, maybe two maximum. So it's been horrible (laugh). I mean, yeah,

INT1 A big difference?

RES Definitely.

INT1 And so in terms of erm your social life that you mentioned to friends and your partner and things, how do you manage to keep in communication with them either via telephone or, or virtually like this during the lockdown?

RES Yeah just facetime, and erm just WhatsApp calls and stuff like that. And then with family back home, we did a few quizzes, but they didn't last long, because it turned out to be a massive effort. So we did about three of those erm and called it a day. But, yeah, just just speaking to loads of people on the phone, erm aunties and everything as well, just anyone, cuz everyone was on the same boat weren't they really.

INT1 That's it. And I'm guessing then it feels like that was quite a big difference to how life was pre pandemic. I'm guessing you've seen people face to face outside of work as well in terms of their social life if you like,

RES yeah.

INT1 And, and did you, I know obviously the very first lockdown where you didn't see anybody for the first six months or so when it started to lift a little bit and we could bubble up and eat up outside? Erm more did you? Did you find that your social engagements increased then to a level where you were a little bit happier with? Or was it there's still quite a big difference between then and pre lockdown.

RES Erm I still don't think it was. So a lot of people in lockdown in Leeds kind of went home and move back home at that stage. So a lot of my friends actually left or went abroad for work and stuff. Err And so even when it did open up, there was a select few to meet up with do ya know what I mean, it wasn't like you had that large selection of friends that was there before because they just weren't there erm so it was kinda, It wasn't as it wasn't as good as before. But it was it was better than than COVID lockdown was definitely....

INT1 So if you think about during erm lockdown, obviously, you were working for some of it. But what else are you doing to try and keep yourself busy? Or what kinds of activities were you doing?

RES Erm not a lot to be fair drinking wine (chuckle) ... erm walking I did go out walking erm most days to be honest, I tried to keep into routine. My last lockdown. I haven't been doing that because I just have zero motivation, plus I'm working loads. But yeah, I used to try and get out every day at lunchtime just to break the day up and just get out of the flat. Get some fresh air.

INT1 Yeah. And erm did you find that that helped getting out and fresh air and what you needed to

RES Yeah I think it was just work were like, Oh, it's fine. It's, you know, do what you need to do. But then it was kind of like why you not at home working when they would ring while i'd be out. So you felt bad for going out as well. So yeah, it was just one of those, depends who you're speaking to. (laughs)

INT1 **(laughs) Ok Were you quite? Would you have gone for walks before? Was that something you would have gone, done..**

RES On the odd time but not not every day? To be honest, probably on the weekend. erm because I'm just doing social things after work erm but not, definitely not every day, but it, it did help. Definitely.

INT1 **erm If we think about the term loneliness. How would you define that.**

RES Erm I think it's like having like loads of people around. But still, like you have the contacts and you have everyone there, but you still feel alone if that makes sense. So I don't think not necessary. I don't think it's necessarily just people not being around, or you being on your own, I think people are around and you're still feeling lonely and feeling down?

INT1 **And is that something that you've experienced?**

RES Yeah, definitely.

INT1 **Yeah. And erm when you have felt that, have you felt comfortable talking to people? Have you have you got people around you that you would feel comfortable talking to them about?**

RES Erm I don't think I really did to be honest, I think I just went to the doctor at that stage, because it was last, just at the start of lockdown. Erm, and yeah, I just don't find it that easy to speak to people I know. I'd rather speak to someone that I don't know. Do you know what I mean? less awkward. So...

INT1 **yeah, that can definitely understand that. And that was that sorry. Yeah. Was that the time erm that you started to feel depressed as well, at the beginning of lockdown went to doctors to their antidepressants was at the beginning of the first lockdown,**

RES it probably wasn't the start because it was all them after the accident, so they put me on some after the accident about two months after, but they just didn't agree with me. err So I stopped taking them. And then I went back, just the beginning of lockdown again, because I just thought it's it just wasn't getting any better. erm So I did go a few months just trying to cope on my own. But I just went to the doctor in the end, cos it just wasn't helping.

INT1 **Do you think that your erm feelings of loneliness have changed? Over lockdown or because of lockdown?**

RES Yeah, definitely. I think it's kinda not been able to get home and stuff as well. Because obviously, all my family are in Ireland, erm my sister's over in Liverpool, brother in London. But it's, you've not being able to go anywhere, see anyone. erm they've all got family and stuff. So obviously, they've all got each other, but you're just kind of sat here on your own. Erm so yeah, I do think it worsened, definitely

INT1 **erm has that made you feel sort of that erm distance between your family and your friends that you haven't been able to close**

RES Erm. It's just made me want to move home to be honest I've been over here for like eight years, oh no 10 year Sorry. I'm considering moving back to Ireland next year. I think it's time. So I think all those things just add up and add up and stuff and you just kind of just enoughs enough, you know, err there's loads of them over there. Whereas there's just not really anyone here anymore. erm I think it would just be good to be around. More people definitely for me. So.....

INT1 **Yeah, I think it seems like a lot of people have sort of reassess their priorities a little bit during lockdown and questioned where they're at and where they want to be. Yeah, definitely... erm So if we think about lockdown, is there anything that has stood out for you that's helped you through it**

RES …..hmmm .... I suppose just calls with with REDACTED, the OT and her coming to see me during rehab and stuff. erm And just speaking to people as much as I can, like, I mean, teams has been great because that we use it for work as well. Erm but it's just been great for calling people and being able to see people erm... other than that. Not really getting out again, like I say, getting out and doing some exercise and stuffs being good. erm that's really that's really all I was doing, to be honest, was very boring. (laughs)

INT1 **(laughs) And to use social media at all to keep in contact with. Yeah. And has that increased or decreased over lockdown? Do you think**

RES Erm....it's probably about the same to be honest, I try and leave my phone kind of away, say that I'm never off it, but I try and kind of even if it's just on a weekend or so just leave it somewhere and don't go near it just because I'm sick of looking at it. erm so I think it's probably about the same I don't think I've used it any more over lockdown just like I say the

teams and the face time and calls and stuff that will probably be about all that's been a bit more.

INT1 **And then on the flip side of that, what would you say has been the most difficult thing for you?**

RES erm... Probably just working in the middle of it just with the tiredness and everything still from the injury as well. It's just been and because you're at home all the time. It just feels like you're even still you know staring at a laptop all day. It just feels even more exhausting. err. So I'm having to take more regular breaks and stuff. But I think that's probably been the hardest bit not been able to get out and just see people, because that's, that's what I love to do. err more so than sit and do admin it's just not for me. So that's probably been the worst thing to be honest.

INT1 **... Yeah, I can get that. Erm how are you feeling about sort of all the lockdowns lifting and society opening back up again?**

RES Erm fine. I'm kind of just like not that bothered to be honest. I kind of like don't. I don't know. I've been out a few times and stuff, but it's just having to book everywhere. It's just an inconvenience now so I might as well just wait until further down the line. But I'm not I thought I'm not really, it doesn't really matter. To be fair, all I really do is work and come home anyway. And a little bit of socialising. So it's not really gonna affect me, other than what I've already been doing, you know?

INT1 **Yeah, that's it you're lifes going to pretty much just gonna continue pretty much as it is now anyways,**

RES yeah.

INT1 **Do you think you'll be back erm working or not working from home fairly soon? Or is that something they're looking to still keep running awhile longer?**

RES They're looking for like erm getting people back into the offices in Manchester. But I don't really go in any way at work more from the depot, if I'm just either at home or on site. But erm ... I should be out three, four days a week, maybe five. erm But I just manage my own time. So whatever I need to do that week, I'll just book my diary around it. But yeah, we should be hopefully we should be out and about a bit more erm. Because then we don't have to do all the two metres and stuff in stores as well. But that's still in place at the moment. So

INT1 **Are looking forward to that do you prefer being out and about than being stuck at home?**

RES Yeah. I moan about the driving but It's much better than being sat in all day in a chair here. (laughs) Yeah.

INT1 **Definitely yeah I know, I sort of wish that sometimes we could still have those things that we took for granted and moaned about continuously, like, the commute and all of those kinds of things. Yeah. And, erm, Sam do you have any questions at all Before I move on to the scales?**

INT2 ***I've got two and one is a more simple one starting with what is your gender identity? If you don't mind me asking***

RES As in what? what?

INT2 ***As in a male, female, non binary.***

RES Female, female (laughs) I don't think I've ever been asked that before.

INT2 ***We asked that all the times on their surveys and things. And then the other one was just how you mentioned about like, family back in Ireland, and then also kind of scattered more around England a little bit as well. erm When things open up again, do you think that you're gonna want to try and like, see more and like, go travelling to see family and stuff? Or do you think that's just something that you might hold off on until you can move back to Ireland?***

RES No, I've actually been back to Ireland twice. Since Yeah. Well, I'm a key worker, so I'd be flying anyway. What I have actually been back twice this year already. Erm I did go down on your team brother in London the week before last. So and that's probably the first time I've actually called in him with them and seen where he lives and stuff. But it takes kinda something like COVID to kind of open your eyes doesn't it. The one thing for me is because I am driving all week, I tend to not want to drive on a weekend and just park up and just walk everywhere. erm but I definitely need to get back I'm looking to the back to Ireland and for two weeks in September as well. And I get back down to London and across Liverpool. So yeah, I'll definitely see a lot more of people. I think it kind of opens your eyes to stuff doesn't it when you can't see anyone so yeah.

INT2 Hmm… Now on to the scales

INT1 Erm I've just got to just add on to the end of that, would you say that that is perhaps been a positive thing that wouldn't have happened if COVID hadn't come that you sort of have reached out to family and more than if COVID hadn't hadn't could have just carried on without really thinking about it

RES Yeah it's made me definitely appreciate people more anyway. Like I would have went home probably once a year before COVID because I just hated it and I just don't want to go or I go for like two days over Christmas and I go on Christmas Eve and come back on Boxing Day. I just hated it. I don't know why erm but now when I go back I really enjoy it like and seeing all the kids and because my sister's had kids recently so like it's now it's like really enjoyable and I actually don't like coming back here. So it's definitely made me appreciate everyone more to be fair.

Interviewer(s): **INT1**

INT2

Respondent(s): RES (P19)

INT1: Okey dokey then that's great. So if we could just start by getting a bit of demographic information about you? Could you- would you mind telling us your gender that you identify as?

RES: Female.

INT1: Okey dokey, your age?

RES: 50 [laughs] just, just, just.

INT1: Could- your employment status?

RES: I'm actually going through ill health retirement just now, I'm just- think it's gonna take about four months.

INT1: Okay. Your living arrangements. So like, if you live by yourself, with family, with a partner?

RES: With my husband and my daughter.

INT1: Okey dokey, oh and what region is it that you live in? I think that's all of them.

RES: Scotland, west coast of Scotland.

INT1: Okey dokey.

RES: On a wee island, if you'd like to know [laughs].

INT1: Okay, then. So with all of that said, we'll basically start by just having a bit of a chat about living with brain injury, and then also... just the experiences of lockdown. And then finally, we'll go into- I'll share my screen just with a bunch of our like, questionnaires, and it's just multiple choice questions, basically from there.

RES: Okay.

INT1: And so if you could start just by telling us a bit about your brain injury?

RES: I didn't have any- don't know what you would call it... didn't have any... don't know what the word is, but, I was at work I was going for my lunch and I walked out the surgery and just outside the grounds, a heavy wait came on my head and I collapsed. The next thing my colleagues, the doctors and that had to help get taken to the hospital, I then had the retrieval team came over in a helicopter. I was basically fighting for my life. And I was taken to Queen Elizabeth in Glasgow. And then that was it, an operation, coiling somewhere about here, I think [points at head]. And I was in hospital for 16 days.

INT1: Wow, did you- were you unconscious, for any of that time-

RES: Yes.

INT1: -you'd collap- how long were you unconscious for was that?

RES: I'm not actually sure, but I think what my friends and everybody told me was the had to- a doctor came running down as well with a crash bag. And then somebody gets sent from the defib because I was... what was it shallow breathing? and they thought I was going, actually.

INT1: Yeah, now I think it must actually be quite hard to know how long you've been unconscious less than one tells you?

RES: I know- I don't- I think it's the fear of what did I say when I was [laughs] gonna not knowing more what I was saying.

INT1: How long ago was that again?

RES: July 2019.

INT1: Oh, so quite recent, actually. How much of your daily life would you say that it has affected for you?

RES: It's been a major change. I can't be the person that I was. I tried to be or I'm trying to do it, but my body's just not following with me.

INT1: In what ways do you think that- do you feel like things aren't, like working?

RES: I can't do things that I wanted to do, like I can't walk. I used to love walking. I just can't do it. I'm unsteady on my feet. I have to have somebody out with me. I can't bend down. And it's just- I get really dizzy all the time. But it's a part that apparently it's something to do with the area of the brain that was affected that controls the kind of dizziness and the movement.

INT1: Have you had any rehabilitation, like, for the-

RES: Yeah, actually, that started- finally, a GP last March erm before COVID kicked off, referred me for rehab, but it was put on hold because of all the kind of restrictions, so I started seeing the rehab doctor in October who then involved the OTs which was never done before, the OTs, I've already had a physio he's been brilliant, the rehab consultant seen him again in March I got a lovely steroid injection, I've got problems with my shoulder and he done that and he's been brilliant because he's somebody that you can talk to that can give you the answers that you never had, when the GPs don't have, um, I would say a good knowledge of particular brain injuries.

INT1: Do you- what- what would you think is- do you think there's anything that's particularly helped you... basically get back to feel like you can just like you said about walking and things like that is there anything that helped you?

RES: Yeah I mean he's, he's trying to help, he explained everything where I didn't actually know that bits inside my brain have been destroyed and it's just being able to try and do as much as you can but your body just might not do it, so it's learning to not accept it as such but I think that talking to him was the best thing ever. He's been really good... explained a lot of things he's a good guy. Yeah.

INT1: So anything- is there anything about that you think has been quite hard as well or do you think it's only been like a positive thing the rehab?

RES: The rehab I think has been very positive, especially when it involved the OTs, the OTs helped they done a lot with my movement and my arm although this- it affect- my brain injury was on this side but I've got problems on the opposite side the OTs helped they done as much as they could but because of this dizziness thing they can't do anything for, so I'm just gonna... I'm glad that the help that I've got that certainly helped me move on a wee bit I'm still not back to what I was and I'm not gonna be if that's- because I'm going to be basically losing my job in four months because I can't do it.

INT1: You- you know about how- like obviously it was quite- it happened like not long before the first lockdown, do you feet like there's anything to do with, like, your rehab that lockdown like have actually like impeded like it could've been easier if not for...?

RES: Well yeah, like the doctor referred me I think the 7th of March and then COVID kicked in. So everything was quite... just a big change, so when I got that letter, it was a bit of hope because it's gonna be somebody, a neuro who knew what I was going through, erm, but yeah, it was delayed by what, seven months or something? But once I've seen him that certainly helped a great deal. Yep.

INT1: So you feel like the only real thing that lockdown has done like sort of hit back on that it's just the delays that have caused rather than....

RES: Yeah the delays but also like you couldn't- you had not- you didn't have a fear of going to the doctors but you were kinda like let not wanting to bother them. It was in a situation where you know people did have a bit of fear you didn't want to go to the health centre because what if you went to the health centre and you picked up COVID? The whole GP thing's changed now yet, you know, you used to be able to go and see a GP in the morning or the afternoon. Now, you just have to phone up, you then get an appointment on the phone for the doctor to phone you back. And if you're lucky, you get to see him in the afternoon. So COVID's changed a lot, I think, with how you approach services.

INT1: Yeah, just from personal experience, I know that my [family member]'s had a lot of those sort of issues as well. You know, when you mentioned about being able to go out walking as well needing someone to be with you when you go out for walks? Since lockdown actually came in have you found that you do go out often? Or not?

RES: Well, no- my friend used to come- it was every Thursday, she would come and get me at the door, we'd walk across the supermarket, and that was like the highlight of my day, and then I would walk back. But with lockdown, she couldn't, you know- I didn't see her. So my daughter was off, she was doing exams, so she was in the house, she was studying. And we would try every so often to go out for a short walk. But it just the whole COVID thing, I think just changed a lot. And then when the restrictions relaxed, then my friend was able to come again. And although we didn't get into each other's houses, we would maybe walk and sit down. There's like the putting greens? That's like a wee grassy area with seats, and we just sat and chatted for a wee bit. And that was me getting out the house. And it gave me something to look forward to- my daughter, you know, she would come and make sure that I was going out so I wasn't just sitting in the house all day vegetating.

INT1: Do you live with your daughter?

RES: Yeah, she's, she's 15. Her and my husband, my son's in Edinburgh. He's what, 24? So yeah, COVID mean that, you know, I couldn't see him. But thank goodness for FaceTime [laughs].

INT1: Do you feel like- like with living with your daughter, as well as your husband as well and like having your friend that you can go and walk with, do you think that there's- there are people that you've had that's been able to help you get through lockdown and be able to get like- helped you during the pandemic? And is there anyone else that you might have potentially relied on? Or just had around?

RES: No, well, my cousin, she got me involved with her, erm, friends, and it was kind of weekly Zoom meeting. And we all got together for you know, a chat, you know, you couldn't go to anyone's houses. So that was something that I looked forward to. And then somebody started doing a quiz now and again, although sometimes I can't concentrate. But it was being part of it. So something that gave you something to do.

INT1: And was that like, pretty much every week as well, like that you would be able to do?

RES: Near enough every Saturday, when people could manage it. And then somebody started the bingo [laughs]. So that was fun and we played for like sweets or things and it was quite good. It was fun, it was a fun part. Yeah.

INT1: How do- do you think it, like, helped you get through lockdown as well, like been able to interact with people, whether it's online or in person as well?

RES: Yeah, because then like although lockdown, you know, you couldn't do things and... to be able to have the technology, I don't know what we would have done if we didn't have the technology. Because people had the fear you couldn't go to somebody else's door. I think technology's helped if it's Zoom or like Messenger things, whatever. It's a way of keeping in touch with people. But you know, it's still hard because everybody else is still thinking about COVID and everything as well. But if you make an attempt to come together, then I think it helps. And everybody can, you know, have a laugh, or discuss serious things as well. So yeah, it was good to have that. Yep.

INT1: That's good. When it comes to like, obviously, you've had your the- wee- the Saturdays for the Zoom stuff. What else would you say that you've been doing in the average week throughout lockdown?

RES: Not a lot [laughs]. I don't-

INT1: Same [laughs].

RES: -I don't have the telly on. I- we watch the news in the morning. And then like, my daughter would follow a routine of what she would do if she was going to school. So she would go upstairs. My husband was a key worker. He was out all the time. So I would just kind of sit, I would maybe go on Facebook or go on Amazon, Amazon Prime is the worst thing ever, well it's not but it is because you could order various items that you didn't really need but we could order them so-

INT1: I've definitely done that.

RES: -yeah, it's it's just a bit different. Yup.

INT1: Do you think that like, the average day has changed as well, like, as you have gone through the like process of restrictions, like, getting lighter and then back down and then I'm not sure if yesterday applied to Scotland, but obviously we've got the extra four weeks now.

RES: All right. I think it's... yes, things are more relaxed, people can do more things. Yes, I think you're allowed in houses. No, but nobody does because I think there's still the fear. You know, I don't know how many bottles of hand gel on different things, you know, I've ordered, but it's been more cautious of things. And even, like, I've had to go away to hospital appointments and you think "Oh my god, I'm going in a hospital, I'm going to get COVID." But your not, because you go in there sensibly. You know, you make sure you gel, you've got your face mask on, and that's it. Try and continue as normal as best you can.

INT1: How do you feel about what you tend to do on a daily basis during lockdown? Is there anything that you would prefer, like, is different?

RES: I think because you couldn't maybe like, although I couldn't walk far, but going out to the shops was a big thing for me when somebody would take me out. So if all the shops were shut, you couldn't go to the shops, but you could still go out a short walk. And that was that, and yes, the supermarket was open, but at the start... you know, we wouldn't go in there. I didn't want to send my daughter for the shopping, so you know, my husband would have to do it and I felt that was kind of hard, because he was a key worker, and there was a massive queue outside the shops, and he would have to stand in that for ages to get a loaf of bread, it is crazy. But I couldn't go over and do that myself.

INT1: Yeah.

RES: But I was that I was annoyed by myself because I wasn't fit enough to go and do it and he was working all day. And he had to go and stand in the queue to get a loaf of bread. It's just silly things. I just wish I was stronger to be able to do the stuff.

INT1: Yeah, that must be quite hard as well, and then also the fact that it's not even your fault either.

RES: No.

INT1: Must be frustrating. Do you, what would you say you've found the most difficult during lockdown? We might have kind of already touched on that a little bit. But I just specifically like one thing that's been the hardest?

RES: I think not... I think not being able to maybe see people as much. My parents have both passed away. My husband's mum's still there but we couldn't go and see her. You know, you couldn't see your friends. You couldn't go into certain shops, you couldn't get off the island, that was a problem. Not that I was going off the island, but there was a lot of issues where, you know, you coudn't travel out of different areas. And just, like, my son he couldn't come and see me. You know, I- I- there's no way I could go and see him. But, thank goodness, as I said before, for technology to try and help the kinda, loneliness. You know, you could just call somebody, you know, although I absolutely hate being on camera [laughs], so it was a big thing going on Zoom calls but we done it because it was a way of everybody keeping in touch and keeping everybody's spirits up to try and deal through a thing that you'd never been done before.

INT1: Yeah. When you talk about leaving the island as well and your son, do you think that like when things are more normal again, you probably would like to do them more.

RES: I don't know if I'm fit enough to get up- he's actually now in Glasgow, he got a flat, so we're had to deal with that all through lockdown [laughs], which was something else. And I had to deal with every process with him, but he came down a couple of weeks ago and it was the best thing ever. We had Christmas, but we didn't because I didn't have a turkey.

INT1: [Laughs]

RES: And we made him wear his cracker hat... and actually Christmas dinner was macaroni cheese because that's what he loved, so we got that for him. But it was having a Christmas dinner and he was able to open his presents. Yeah, that was good to see him. Definitely.

INT1: That's good. I'm just changing up a bit- to a little bit away from lockdown. How would you define the term loneliness?

RES: I think when you don't have any way of interacting with family, friends, people in the community and you're locked away in your own wee world. And what you don't want is to be in a place where you have no contact with anybody. I think it would be worse if you just sat and "aw this is lockdown I'm just doing nothing." You've got to make an attempt to try and keep in touch with everybody. And erm- it's everybody sticking together and trying to help each other get through I think a difficult time.

INT1: When you mention a difficult time, obviously, the last year or so do you feel like since, like the last year, maybes a little bit more from the start of lockdown. Your feelings towards loneliness have changed during that time?

RES: I think when the restrictions had relaxed slightly, people were more "Hmm, yeah, I can do this" and, like, I mean, a highlight for me was when my hospital appointments [laughs]- I've never done a Tesco click and collect before. So I was able to do my shopping and then- they just chucked it in the back of the car, and that was brilliant. And then I went to my hospital appointment. That was fine. Yeah.

INT1: That's good. I've never-

RES: Sorry I don't know what you'd said there I've forgotten.

INT1: It was just your feelings towards loneliness over last year if they've changed at all, or what you might have thought of loneliness before the start of lockdown?

RES: Yeah, I think... I didn't really, maybe at the start of lockdown there wasn't really anything, but then it was kinda space where nobody really knew what was happening with COVID, nobody- there was uncertainty. Nobody knew how bad it was gonna be. And then maybe it was a bit in the middle where you thought, you know, this, you know, I've just got my family. But I've got somebody, I think it would have been harder for somebody if they were on their own. Erm, but I had my family and I had the support from them. But I think maybe at the- before the lockdown, I was okay, as in the middle, I was maybe a wee bit kinda unsure, lonely. And then as I said, when things started to relax, and then the Zoom calls, it made a big difference. And you didn't feel as kinda out on your own because of what's happened to me, and I can't do things. But if you involve yourself with your friends, and you do the best you can to try and keep it going and not be so lonely.

INT1: So you- think that, like, loneliness could have crept in a bit more if you hadn't had those things?

RES: Yes.

INT1: And the- being able to maintain like, your social relationships during that time through technology has been helpful? Yeah?

RES: Definitely most-

INT1: Not to put words in your mouth.

RES: I think if you didn't have like you- your technology, you know, I feel maybe for the elderly- I think it must have been hard for people who didn't really deal with technology. And all you could see was- or if you had your television, but you didn't really go to the outside world. I think for an older person, it must have been a lot worse.

INT1: Yeah. Yeah, yeah- I can get that, especially knowing, like, my granddad and people like that. I think just the final of the questions before I throw to [INT2] would be how do you feel about life post lockdown, and things opening up and getting back into society, basically?

RES: I think it's... it's a good thing. You know, it's unknown, because you've been through you know a serious big change, everybody's been through it. But it's moving, I think you've got to move forward. You've got to try and be more confident and just go out into the big bad world because the big bad world doesn't- isn't as big and bad as you think it is, you just have to go out and try and get things back to normal and act as normal, but still gel, still wear your mask, but just try and get back to normal as best you can.

INT1: Yeah, good, positive attitude to be having. Erm [INT2] is there anything that you would like to ask or if there's anything that I've missed out? [Laughs]

INT2: Yeah, no, just a couple of questions, if that's okay from me-

RES: Yeah.

INT2: -I was just wondering, you mentioned obviously you've used technology quite a lot during lockdown. Was that something you'd use before to keep contact with people?

RES: Not in a great deal. But you know, I had my phone, I think was last August. I thought- oh, during the lockdown as well, in the April my dad passed away. So that was difficult because you couldn't do a funeral there was only eight people allowed and it wasn't, it just wasn't right. And so I decided that when I got some money, and I thought, I'm going to buy an iPad, because then it gives me a bigger screen.

INT2: Yeah.

RES: And so yeah, technology, I think has helped so much. I think you during lockdown for people to cope with things.

INT2: And do you think going forward that's something you'll continue to use?

RES: Yeah, because I think although we've kind of- the nights has been quite bright and people are out walking and things, so we haven't had our Zoom meetings for a while. So I think it still will continue, because it was a good laugh we've still got our group in Messenger and there's wee chats going and different things, but I think it will continue, because it was a way of people all meeting together, discussing the good and the bad points. And I think it was just a great way of keeping in touch.

INT2: Yeah, definitely. And then I was just wondering, you mentioned sort of, I think it was before lockdown, you'd go for a walk with your friend to the supermarket, and it was sort of your highlight of the day, how would you say, or how much of your activities have changed or different from pre-lockdown to during lockdown?

RES: Well, we didn't- we would try- my daughter and I and go out, you know, wee walks when we could, how I was feeling... today is actually not about the so I'm able to talk. But, you know, I miss, you know, meeting my friend- because there's things that you, you know, you're with your daughter it's fine, you have a good laugh, you talk about things, you talk about things for the future, like, a big thing for us was... we were meant to be going back to Disney, the year I collapsed, so that all got cancelled. So we're being positive, we're looking to "will I be able to fly again? Let's be positive, let's talk about planning something." So that's what we've been doing. Yeah.

INT2: So just trying to keep really positive about things.

RES: Trying to- trying not, kinda then go into a situation where I think, you know, some people could maybe start getting the old depression like, you've got to really try. What's hard after having a brain injury is deal with everything and try and stay positive and not start... kind of, you know, going into... going into kinda, not a cave, but you don't want to do that you've got to try and stay positive and continue and do your best. That's, that's me.

INT2: If you did start to feel sort of a bit like that like you're slipping into a bit of a depression or you were starting to feel a bit isolated and loneliness was starting to creep in. Would you feel that you had a good network around you that you could share how you were feeling with? Would you feel comfortable to talk about it?

RES: Yes. And I think as well- somebody told me it was through the Brain and Spine Foundation, there was a LinkedIn group on Facebook. And that has been a good thing as well, because it's all people who have had the same as me. So it's people who know exactly what you're dealing with, and how you have good days and bad days and what you can and you can't do. So yeah, that's a good thing as well.

INT2: Brilliant, and have you found that support that peer support helpful, not just in lockdown just in general?

RES: Yeah. And also like you wou- I wouldn't have been able to travel to Glasgow. And when I heard about Headway, and we have the Headway Zoom meetings, they've been brilliant, because it's all people, again, with brain injuries. And you know, you have Zoom meetings, and everybody has wee chats about different things. And I think the support from Headway has been fantastic as well, because they're at the other end of the phone, you know, your weekly Zoom calls, or a monthly brain injury group is a brilliant way because it's all people who understand exactly what it's like whereas somebody from the- didn't know, and think, different maybe ways you're reacting. But- they call it llike an invisible illness because I don't have a big scar on my head, I don't do things- but they don't know what it's like for you 24 hours a day. So when you're in a group of people who understand, that's good. My friends are close friends understand what I can be like. So they're supportive, my friend, if we are out and she can see you can start to have as I call it one of my iffy moments. I can just go suddenly quiet and I feel that I'm going down and she knows and she'll keep me talking and... yep. My friends know how to deal with me [laughs].

INT2: That's always good- it's always good to have good friends- a good support system around you. And do you find- is fatigue and tiredness, another sort of thing that you've been affected by?

RES: Very much so. I can fall asleep- it can come on, suddenly, my eyes just close and I can't get to my front door to lock it. I had this before I went on my medication for my tremor. So it's just something I have to live with. I can be really- just really, really tired. But I'll go to my bed at about 10 o'clock, and I'll sleep till maybe about five, and then I'm awake and I'm up [laughs]. Try not to make noise.

INT2: And then my last question for me, and it's purely just because I'm nosy. Which island are you on? Which island are you living on?

RES: It's the island of [location]

INT2: Oh lovely, very nice.

RES: If you Google that and look up some nice pictures, you'll [inaudible].

INT2: Yeah it's beautiful. Lovely. That's it, that's all from me, [INT1]. Thank you.

INT1: Alright, thank you. So now I'll share my screen and we'll go through the questionnaires basically. So if you can see yourselves there.

[Progresses into scales]

INT1: I lost interest in eating when I thought about or was exposed to information about the Coronavirus.

RES: No I've put on about five stone since it happened so no, not at all [laughs].

INT1: And then finally I felt nauseous or had stomach problems when I thought about or was exposed to information about Coronavirus.

RES: I'm going to say not at all. I mean I think it's now it's excepting the Coronavirus is here and how you best deal with it in a healthy way.

INT1: Yeah. Yeah, definitely not the first person I've said not at all for all of them. And something that I've felt like I've found is there's like, once or twice I've like had a little bit of worry that I've actually got COVID. And like, I've only actually been concerned about COVID when I've actually thought I might have it like if I haven't thought that I've got it. I've haven't been worried at all. I've been wanting I've wondered if I know these other people might have been my life.

RES: No, I mean, that is a worry about COVID. But I think it's now going to be just how life is going to be, and it's how you best deal with it. I think you've got to be positive and get on with it. Like my daughter tests twice weekly for school. I managed to get a box of seven tests [laughs] but I haven't taken one out of the box yet, but-

INT1: They're not pleasant to do.

RES; -there is a fear of it- getting COVID but you've got to continue with your life as best you can and with the problems that I have.

[Progresses through scales]

INT1: How often would you feel that you're like companionship on a scale of hardly ever; some of the time; or often?

RES: Well now I felt a bit strange when the schools did go back can't remember when they did go back, and then a lost the fact that my daughter wasn't there to rely on for things, but you know, she would make a packed lunch in the morning, and she would make me something to eat for my lunch. So she looked after me. So when she went back to school, that was somebody missing- somebody face to face. But yeah, I did like companionship... maybe some of the time. Yep. Maybe. Because she's went back to school, and there's nobody in the house. But then you, you give yourself things you put the telly on at lunchtime. And you know, you do things and you've had exercises to do as well. So, yeah, some of the time, yeah, go with that.

INT1: Fair enough. How often do you feel left out?

RES: I would say... I don't know, it's between hardly ever and some of the time, because I can't do things the way I used to, but it's not- is this to do with the COVID or generally during in the lockdown how do you feel left out?

INT1: Generally, I believe.

RES: Yeah, well, yeah, I would say, between hardly ever and some of the time.

INT1: If you had to veer towards one or the other?

RES: Yeah. How do you feel left out? Some of the time then, because I can't go and do things like if people that are going for walks over the hills and things I can't do that, people are going out to the gin garden. I can't do that because I haven't drank for nearly two years. Yeah, you do feel left out some of the time, yep.

INT1: And how often do you feel isolated from others?

RES: I would say hi- hardly ever because I've got somebody on the end of the phone, you know, I'll phone different friends, they phone me when they come in from work, or, you know, one friend works part-time, so she'll phone me after she finishes work at lunchtime, different days through the week. So know, how often do you feel isolated? Hardly ever? Yeah.

INT1: I've been feeling optimistic about the future?

RES: I would say often because you've got to look forward to things. You can't dwell on things that's happened in the past. You've got to move on. Yeah, often.

INT1: I've been feeling useful?

RES: Some of the time, because I'll maybe one of my exercises is having to do the breakfast dishes in the morning. So yeah, some of the time. It's not much to some people, but as to me, yeah.

INT1: I've been dealing with problems well?

RES: Yeah, I've got to watch my stress levels because I've got another one that's not been operated on apparently that's in my eye. And so yeah, have I had problems dealing- dealing with problems well? Dealing with my work's an issue. So... some of the time.

[Progresses through scales]

INT1: I get worried when I find out my support network are having fun without me?

RES: No, because I know I can't do things, but they do involve me in things. And yes, Facebook, you see pictures, you see what they've been doing. But you just wish you could do it but you can't. Yeah.

INT1: And when I go on holiday, I continue to keep tabs with what my support network are doing?

RES: Well, I don't go on holiday now [laughs].

INT1: If you were to.

RES: I think since I've been off work for two years, it is a big holiday... not. Yeah, I mean, we all keep together, so we keep in touch and we find- you know, we- we have our group chat, so yeah. When I go on holiday. Yeah, very true, then. Yep.

INT1: Yep. Okay. And that is the final one. And I'll stop sharing my screen [RES and INT2 can see themselves briefly through screenshare].

RES: Oh, no! Don't do that to me.

INT1: So yeah, that was everything. It's been lovely talking to you, thanks for joining us.

RES: It was nice to talk to you too yep.

INT1: Thank you, so basically, is there- before we end this is the anything that you'd like to ask us or know more about the project or anything like that?

RES: No, it's just when that see that and you know, thing from that was sent from Headway I thought it was a good thing to maybe get involved in and you're sharing your experiences and so that maybe in the future... people who've been through similar situations, they'll know if there is papers and different things they'll be able to understand and maybe like the consultants and things then they'll have heard from everybody and they best cope with it. Yeah, so I think it was a good thing to get involved in even though I'm absolutely camera shy and-

INT1: Thanks for having it on.

RES: -I do look awful-

INT1: No you don't!

RES: -so, yeah, yeah, so five stone. Yeah, that's what I've put on.

Interviewer(s): **INT1**

INT2

Respondent(s): RES (P20)

INT1: So yeah, we'll just start with getting some demographic information about yourself. That's okay. So would you mind telling us the- your gender identity?

RES: Male.

INT1: Okay, your age?

RES: 47.

INT1: What region of the country you're living in?

RES: East Midlands

INT1: And your employment status?

RES: Unemployed.

INT1: And marital status?

RES: Living with a partner.

INT1: Okay. And I was gonna say, living arrangements there, but, that was covered by that. So for the marital thing, are you married?

RES: No, living as if.

INT1: Alright, so cohabiting would that go down as?

RES: That sounds so dirty when you say it like that [both laugh]. It's cohabiting, yeah.

INT1: So if you could just start by telling us a little bit about your brain injury if that's okay.

RES: Yeah, there was multiple actually. Started in 1993. I've had one, two, three... five, five. The first one was, I was assaulted in 1993 and I had head stomped on. The second one, I was hit round the head with a metal bar in 1994. Third one, I fell off- I fell into a hole and I smashed my head open. The fifth- fourth one, sorry one, two, three... the fourth one was a motorbike accident. And the fifth one was, I was hit around the head with a piece of wood... yeah. And that was in 2001.

INT1: So from there, has there been any sort of significant ways in which your life's been affected by the various injuries that you've had?

RES: Yeah, personality change. I was quite an artistic person but that went... I see double all the time, I've got double vision. I sweat, like, profusely... got an intolerance to loud noises and sound. Got sleep for- extreme sleep difficulties. Whereas I, it's like a bit like panic attacks, but it's not a panic attack, you know I jump up in my sleep quite a lot. Can be three or four times a night. And that's been going on for 25 years. You know, I mean, that's in a nutshell, it. But and I have memory problems as well I can forget things quite, quite easily.

INT1: So it sounds like there's a lot of ways in which your life has been changed by-

RES: Yes.

INT1: -day-to-day activities. How would you describe your average week and the typical day?

RES: It's difficult. That's how I would describe it, in a nutshell... every day is challenging. I mean, it doesn't end it's morning, through the night, you know, I mean, it's through that- it's 24 hours a day. I mean, I hate to say but in lockdown [laughs]... at the beginning of lockdown was fantastic. There was no cars on the roads. There was no a lot of people out. And for me, that was beautiful. It was like heaven. Because it wasn't noisy. There wasn't people everywhere. Because if there's a lot of people about I kind of, it's like I see everything and it all comes in, and I can't- I can't get rid of it. It's everything's at once, you know what I mean? So, yeah, so that's how it affects me on a day-to-day basis.

INT1: So would you say erm... when whereas, like a lot of people have kind of struggled through lockdown, do you think that's actually been something that's been quite beneficial to you?

RES: Was- it was initially yeah, the first couple of months, it was- it was... I mean, it was- it was a rest, it felt like a rest for me. But then, like you got it- you know, the lockdown was coming, and then it was ending, the first one, and then things were going up and down. And it was

like mayhem, it was like, starting again, right- right from the beginning. Because there was- everybody was doing more than what they would normally be doing. Like, I got abuse at the shops as well for because I sweat, and I get fatigued really quickly, when there's a lot of things going on. People thought I had the virus, I was getting abuse for that. So that kind of put me off going shopping. But, you know, that's the only kind of bad thing at that time. You know, I mean, but when when lockdown was ending, it was, it was, it was all over the place. You know what I mean, I mean, it was in- some of my symptoms were a lot worse, I become fatigued quicker, and more regularly than I normally would do. I can kind of avoid those situations on a day to day basis, well try to. But when it was so you know, not predictable, there was nothing predictable outside. It was very hard.

INT1: So without- without putting words in your mouth, that sounds a bit like, you're, erm, suggesting that like as you first had the brain injuries and changes that you had to adapt with, it's almost like as we went out of lockdowns before we came back into them that it was like kind of readjusting to-

RES: Yeah, it's exactly that. Yeah. It's like emotional control. I mean, I used to be- when I first had the first couple of injuries to my head, I was- I was always emotional, I was crying all the time about everything. And I found that coming back, even though years later, I have not been like that. You know what I mean, in fact it's been the complete opposite. You know what I mean, I found myself when my first child was born, you know, I almost felt guilty because I didn't cry. Do you know what I mean, you know, you see other dads walking around rubbing their eyes crying, and I'm just standing there "Well, you know, he's been born and god for that" that's what I thought you know what I mean? [Laughs] But yeah, so no it felt like that part of me had gone. Do you know what I mean, but now it's, again, it's- it's all over the place. Do you know what I mean, and again, because of this, we're coming back out of it again, or we've come back out. You know, it's- outside is a bit of a nightmare again. I mean, I can't wait to get home. You know what I mean, when I'm out I can't wait.

INT1: Yeah.

RES: So yeah, you're right there in what you said it's- it feels exactly like readjustment again.

INT1: Speaking like of the initial adjustment, as well, when you've had the various injuries that you've had you have any- any form of rehab to try and help you?

RES: Yeah yeah I have, in fact I still continue to see a rehabilitation doctor here in [location], where I live. I mean, there's only so far they can go, you see. And he did- I hate to say, you know... it's like... I feel like a bit of a pain do you know what I mean? Because there is a lot of people have been suffering, ain't just me, do you know what I mean? There is people out there in a lot worse position. And, you know, it feels like I can't contact people for guilt I feel guilty for you know what I mean? So, you know, I just feels like, I got through this before, I can do it again. But it's not easy. [Laughs] It's not easy.

INT1: When you speak about not being able to contact people and stuff like that do you mean, now with us being during in lockdown, or specifically, just, like, when you had the first injuries and you were undergoing rehab?

RES: Well it's exactly the same like I said, it's exactly the same as back then. I grew up in a military background. My dad was in the army. And his his ethos was, "if you can walk on your own, you're okay after an injury. You can walk, you can talk, you're fine." It doesn't matter what happened afterwards. You know, the kind of topsy turvy life that was trying to adapt to, for me panic attacks initially and then it was like all sorts of things that came along with that, because I didn't know what was wrong with me. And, you know, it kind of is like that again because... I've... I feel guilty asking for something do you know what I mean asking for help, do you know what I mean? And that's just an inbuilt thing. It's just something I grew up with, and you only ever go if you've got something seriously wrong with you [laughs], which is a bit ironic really, when I did have something seriously wrong with me and is maintained. If I had done something about it back then I may not have any of the problems I've got now. But I can't remember what your question was sorry [laughs].

INT1: It was just about if like feeling like you can't ask for help is specific to-

RES: It's like neuro psychology- I'm in contact with neuropsychology here in [location] as well. I mean, in fact it was them that put me through to what you guys are doing. And... you know, I find myself apologising for phoning them- my partner can only do so much like, you know, another perspective, there's distance from me is always better than, you know, a personal perspective. And I don't like to burden my partner as well, so... yeah, it's exactly the same as it was, I don't want to do it, you know what I mean? But I know I have to, because otherwise, you know, I was gonna get yourself in a state and I just don't want to do that. You know what I mean? And I forgot your question. Again. I apologise [laughs].

INT1: No, it's fine, you've- I feel like you've answered it. When you mentioned your partner as well.

RES: Yeah.

INT1: You okay there?

RES: Yeah, sorry, I've just got an itchy eye [laughs]

INT1: Okay, I was just gonna say you can take a minute if you like. When you- when you talk about your partner as well, and like, there's only so much that they can do to feel like... when it comes to the pandemic and stuff, do you think that there's- you've had people

around you that you could speak to and like, and be help- get help from and stuff like that. Although you did say that you might have found a pandemic easier if anything?

RES: Apart from my immediate family, no. My- the rest of my family live quite far away, so, that was all out of the question. You know, I mean, there was things going on with him as well, so... I mean, for me, it's very easy, it's very easy to, like, just to have my immediate family. Other members of my family make things complicated. Do you know what I mean and they've got complicated lives, and they're doing their own thing. And, you know, for me, just being in this little bubble of mine is fine [laughs]. Because anything else is too much do you know what I mean?

INT1: More quality than quantity?

RES: Well, exactly. Exactly that yeah, exactly. Yeah. So again, I mean, my parents were coming up to visit me every couple of weeks or so, prior to the pandemic. But, you know, it was such a stressful time when they were coming up. I wouldn't say that I didn't- I wasn't liking them coming, of course, I was liking them coming. Because when they were here, it was fine. You know what I mean, it was the fact that, you know, there's all the build up to it, you know what I mean, it was- it was difficult. So almost like- it's almost like as well, my family, I don't know them, do you know what I mean? So every time they come I'm reacquainting with them, do you know what I mean? But after you know, [inaudible] is okay. But you know, it just goes through that every time so during this period it was being good, I've been on camera with them... you know, it's been good for us just to be this unit, just us it's just so easy. So that's helped in a lot of ways.

INT1: So when it comes to like technology and stuff like that, to use much of that, like Teams what we've- what we're on now?

RES: No, I was using Duo on my phone with my parents. This is the first time I've used this so, it frustrates me to be perfectly honest do you know what I mean? Just now I was just- couldn't find what I was looking for, you know what I mean? I only found it yesterday I found it I did see [laughs]. Like today- I was practicing yesterday, you know what I mean getting ready for this. But yeah, so you know, it's a bit funny, but I found it's on here and that's fine.

INT1: That's good. How do you feel about what your life is like day to day and what you do and potentially how it might have changed? Like obviously, you said about how it's probably better with lockdowns. How do you feel about the two?

RES: Well I'm daunted by the fact that- that it's ending. There's no ending but... I mean I wouldn't say that I would like it to continue, lockdown. Because there's things I miss, you know, there's things I miss a lot. And there's things about partner misses, you know, I'm very aware of that, you know, but... sorry, if you ask the question again, sorry.

INT1: **Um, it was basically just about, like, what the, how you feel about the differences between your daily life basically, in and out of lockdown. But the way I think you're talking a little bit how you feel about what life will be like after lockdown, which I suppose is the kind of go together those two questions.**

RES: Yeah. You know, I mean, now... during lockdown, like I said it was- it was... it was easily managed there was things where, you know, everything was easily managed. There was nothing, you know, of a surprise apart from obviously the pandemic, but coming out of it now. Even though we are coming out of it, things ain't the same. Nothing's the same. So it, you know, just going into shops and watching people, you know, like I said, I get flooded, everything gets coming to me too fast. You know, I can't get rid of it. It's all there. So when I look around, and there's a lot of people about, I get very tired very quickly, I get extremely quite- I feel ill to be perfectly honest with you. And I'm not relishing this because people do- are doing things now they will normally wouldn't do like I said, you know, people wouldn't normally gathering great big groups outside, but because they can now they will. And, you know- you know, for me, it's just a bit of a nightmare, you know, traffic, traffic noises and everything, I'm not looking forward to things becoming normal, so to speak. It's not particularly good thought for me.

INT1: **Yeah, I understand that. Like, I kind of feel a little bit like that, like, and othe- I feel like other people might have a sense about... there's a lot of caution about things going back to normal. Like, especially like people who haven't been vaccinated and things like that. And, so I think that the next thing to go onto would be like a little bit of a change towards erm- how would you define the term loneliness?**

RES: How would I define it?... For me, loneliness would be not being able to reach out, for me is a loneliness factor. I've got a lot of people here, I've got three children got a wonderful partner. And, you know, for me, I feel bad for saying that sometimes I feel very alone. And right now I feel alone, simply because of... I don't really, it's like an inability with me to try and get help. If I need it, it's at the end of the phone. But for me, it's like an inability, I have to put it off and put it off. Do you know what I mean? So for me, that's what lonely- I define loneliness as not being able to reach out when needed. It's more of a, you know, like a... [laughs] I don't know like, this- that's what I view it as.

INT1: **It sounds like it could be like in that's- like sort of way could be like a little bit more like a cycle that's quite hard to break out of as well if like you feel alone and you don't want to tell anyone that you're feeling alone, they could, like, feed into each other. And it sounds difficult.**

RES: It goes through the roof for me as well you see, because I have studied once before, and I was put in touch with psychiatric services, and it wasn't needed. It wasn't a psychiatric problem. He was and they were saying I have anxiety and all this and anxiety is part and

parcel thing, but that's only the after fact it's more a symptom of rather than, you know, a cause of. For years and years I was going down that route. So there's a lot of things as well the way I was brought up and the fact that you know, it always feels like I'm pushed into another sector do you know what I mean, and then I have to go back round again and start all over again. So it's kind of put me off to be perfectly honest with you [phone rings] sorry about that neuropsychology for me has been fantastic. When I got in touch from them. Very, very helpful. So it's not that easy to for me to just reach out.

INT1: Might be some worth and potentially- if like there could be scheduled things for them reach like- to ring you-

RES: They are now every couple weeks, because I had a bit of a thing you see with some people who I decided to divulge what was wrong with me. And in backfired [phone rings] on me, it backfired on me and we became the butt of people's jokes, you see. And this was during lockdown, I thought it would being helpful to me, but it turned out that they were just trying to get more information about me. And, you know, we were getting a lot of harassment at the door and all the rest.

INT1: That's awful.

RES: Yeah, I know, I know, I mean what do you do? But that's- so I had no choice but then to call- to reach out to, again to neuropsychology, and they're phoning me every two weeks now to find out, you know, how things are and what's going on. So, yeah, that's basically like you said, you know, a routine kind of thing. You know, which helps, it does help.

INT1: Suppose that's one benefit, even though it's a negative way that it had to come about.

INT2: Is there anything particularly about the rehabilitation that stands out as being helpful for you?

RES: It is more over that, I occasionally- occasionally I catch what happens with other people. Because then I relate straight away, you can be in a like a- like a conversation. And, you know, a couple of times the rehabilitation doctor has said, "oh, I've got this." And if I've watched people's body language, as well, and if they look normal and relaxed, I mean, you know, he passes me a pamphlet to read or something. I think it must have happened to somebody else do you know what I mean? So then I don't need to go down that route with him. I don't need to try to remember things or whatever. I can see what's- what's going on in front of me. And like I said, you know, there's a, like a relate kind of thing to it, do you know what I mean? And occasionally I will say, "I've got another person that does this or that." And they might help them. So yes, it does. Yeah. Yeah. I take away quite a lot from you know, like I said, I see everything by choice or no choice, everything I see. So I pick up on people's body language and the way they are, which is helpful, I suppose.

INT2: Yeah, definitely. I was just, sorry, [INT1], I just gonna say as well, with the Headway, have you been erm-

RES: Yeah I've got- well, I'm in contact with them. But it's like early doors with them at the minute because, you know, again, they've sent me a list of things that they are able to do and can't do. Again, you know, things are just starting to open up again. So I've got a Headway card and, you know, occasionally I flash that around when I get a bit irritable, but the thing is, you know, I'm waiting for them to contact me, still, but they are going to. So they've- I mean, I wouldn't see the thing is that I know that people are in a lot worse of a state than me you see, people can't walk, you know, there's a lot of people that are in a bad way. And to look at me people say that wouldn't think there's anything wrong with me, you know what I mean? You know, they don't know me really you know. So, to answer your question, yes, I'm just waiting for them to open up a bit more in the services that they provide.

INT1: I think when it comes to as well, the thing about like you say about people looking at you and not thinking there's anything wrong, like there's other people's we've spoke to who've used the term like 'invisible illness'. And like, that's exactly what it is. And like when you, like talk about other people having worse than it's kind of all relative, like, if something's a problem for you, then it doesn't really matter if it wouldn't be a problem for someone else. Like that's- I'm not telling you how to live I'm just like it-

RES: I know but this was in early lockdown. People that normally wouldn't speak to me were speaking to me, do you know what I mean? I didn't know how to reply, because you- you know, usually when it's people talking to me, I don't know what to say do you know what I mean, it's just, I go blank. But the thing is you know, I found that people were- that we came across were friendlier and it didn't matter what you know, what their backgrounds what they look like, you know, for me, you know, it just seemed like a friendlier world, you know what I mean? It really did, it honestly did. But now again, it's just his back to how it was in fact to find it worse than it was to be honest, a lot worse.

INT1: When you, as- like say about how it's changed as well when we were talking about loneliness do you feel like how you feel about loneliness has changed since the start of lockdown?

RES: And I feel like I've been justified in the way I thought... I think it's justified. A lot of you know- it's for me, it's like, it's now become an excuse do you know what I mean? I don't have to talk to people because they're not nice. That's the way I see it. And... but the thing is I've got three children, I've got a five-year-old and a six-year-old and a seven-month-old. I used- I was during lockdown, I was able to take my two children to school, which I've never done, I've never done. Because I was able to contact the school and ask them if they could arrange something that I could bring them to school because I didn't want to be around a lot of people. And they did. And they accommodated it. And I was able to take him to school. But

then things started going back to normal. And they couldn't do that anymore. So I had to stop doing it. And then my children, were for not invited to parties and things. So it feels like my loneliness, the enforced loneliness that I have, has now become my whole family's problem. Do you know what I mean? Because people are aware there's something wrong with me. And because I volunteered this information, just out of friendliness, as an introduction to me, do you know what I mean? They weren't advice parties and all the rest of it, and you know, we get treated quite badly, to be perfectly honest. But that only happened after I wrote this letter to say my name is so and so, I do this, you know, I've- I've suffered from multiple head injuries and brain injury. And it seems now that they've seen me as a weak person, do you know what I mean? So, but it feels now I've encapsulated my whole family in this kind of thing. And it makes me feel even worse about what's going on.

INT1: That's rough that like. I do feel like it's... obviously it is a social aspect. Do you think that your approach to social relationships has changed because of this attitude as well?

RES: Yeah.

INT1: Yeah... Do you, when you, speak about the enforced loneliness as well. Do you feel like, like, people around you like family they understand how you feel about that, and you can talk to them about it if you need to?

RES: My family? Well my- luckily my children are young enough to not really care, to be honest. And this was told to- this was given to- this information- because I didn't think, to be honest... neuropsychology said to me, they're all they care about is their toys what they're having for their dinner and where they're going. As long as they're having a good time it's all good, for them. My partner... she's not that bothered, she's told me she's not bothered, you know, I mean, like- so there's enough people here, you know, we've got a wonderful family, you know what I mean, so... But yeah, so, you know, that's- you know, I'm try to digest that, you know what I mean? It's just guilt- I feel guilty for, you know what I mean, seeing them into a position that I'm in, or that I'm kind of, I say used to be in but I'm not anymore, because it's all fluid, very fluid, and it's up and down. So yeah, I think they're alright. I think my family are okay, as far as everything else goes, they don't look that bothered to be honest.

INT1: And do you find that it'd be helpful, like, when you talk about guilt? Do you think that that takes a bit of a guilt away from you and make you a bit more comfortable?

RES: Yeah it does, yeah, yeah.

INT1: That's always good then.

RES: Yeah, yeah.

INT1: I think that- that's kind of everything from me, [INT2], if you have anything you'd like to chip in?

INT2: Yeah. I just wondered whether there's been anything particular that stands out that's been especially difficult for you through lockdown? We spoke sort of about how, especially in the beginning, you actually quite liked it, because it was a lot quieter and less people about has there been anything that you found hard?

RES: Oh, yeah, he was going into shops, shops, shopping. Before it was- it wasn't intolerable, but it wasn't tolerable either [laughs]. It was, it was doable. But then, you know, the shops, you know, at the beginning, everyone was going crazy, you know- you know the toilet paper thing, and, you know, everybody [inaudible] houses and all the rest of it that they were trying to do. That was a nightmare. You know, I could not go around, I just could not go out. So that particularly is bad. The facemask thing... I think it dulls one sense for me, so that's one less thing to worry about sort of thing. So wearing a face mask for me, you know, it's okay. You know, then I've only got to worry about my eyes and my ears, rather than, you know, taste, smell, and everything else. So that was okay, I didn't mind that- erm queuing, no, can't do it can't queue. So that was a nightmare. But off the top of my head, I can't really think that there is anything else apart from that- oh, parks! I used to like going to parks. But there was so many people in the parks that that became out of bounds [audio cuts out] but on the street, you know, on the street was new for us [laughs], you know what I mean? We don't really do that much. We go to the parks. But yeah, so I think that's about it really, as far as intolerance of things.

INT2: And what about sort of how you- you filled your days, what kinds of things were you doing through the day? Before the pandemic and then during? Has that changed a lot?

RES: Well, no, but erm, drawing, I mean, that's an ability I lost a long time ago, along, you know, straight after I got attacked sort of thing. What I was able to do, however, it took a long time to do it, but I was still able to do it, we got all new furniture, like flat pack furniture and stuff like that. And before, you know, it would be a no-go at all. But I was able to do that, because, you know, I built like a table, a TV unit, cabinet, a thing bu- bunk beds, you know, it was- it was- it was really good. You know, I was so proud of myself, you know what I mean I built this I built that I built that, do you know what I mean? Which is something I haven't had the pleasure of for a long time you know being able to physically do something. But yeah, I mean, it took longer than it should've done, I'm not gonna lie [laughs], but I still did it do you know what I mean, it was just fantastic, so-

INT2: Yeah.

RES: -well happy with that and erm-

INT2: I bet!

RES: [inaudible] with myself, yeah. So yeah.

INT2: Excellent, next time I have a trip to Ikea I'll know who to ring here to rcome and build it all for me [all three laugh]. So would you say that really, especially in the beginning, that- that lockdown has actually been better than life was before, in some respects?

RES: For me definitely, yeah. I mean, the other- you know, the shopping things and all that. I mean, they were... you know, they were- it's not something I would ever want to recommend doing again, for anybody, but definitely, it freed up space in my head. It just felt like it freed up space. You know what I mean? I didn't have to think for myself, I was told what to do. And that's what we did. Exactly to the letter, and we only went out when we had to, we exercised we did whatever. And we've- we got through it. And you know, so, I forgot your question as well [laughs] oh sorry.

INT2: It was just to say did you- did you, in a way did you prefer the lockdown in the beginning as opposed to- to life before?

RES: There was an initial shock, obviously, as it was to everybody. But as soon as like I realised, as soon as we went out, in fact that the roads are empty for me it's a dream. It was perfect. It was just silent, all just silent, was lovely.

INT2: And would you have said looking back now that if you if we go back to before the lockdown. Have you adjusted quite well, to your- to your brain injury, would you say? Were you sort of, as far as you were aware, ticking along with life quite well? And it wasn't until lockdown happened, you realise the difference?

RES: Far from it, far from it. In fact, I caused myself quite a lot of harm trying to get used to what was wrong with me, in that... I used to manage the way I- I didn't know what was wrong with me. So the way I used to manage it was I used to drink I used to go to work and drink. I used to be a chef. I used to drink at work and- and I eventually caused myself so much damage just by managing my- my life through drink. I nearly bled to death. So- I've forgotten what they're called... varices, a lot varices and cirrhosis of the liver and all that. I mean, I haven't had a drink for 14 years, but it's just a case of, you know, when I came here and all the rest of it that I was told what could possibly be wrong with me, which is an eye opener, because for years I was told it was just anxiety. But it obviously wasn't. And then when I was told what was wrong [laughs], you know, it was like a weight was lifted off my shoulders then but then there was, you know, well, "what are you gonna do about it? You're gonna fix me then?

Am I gonna get fixed?" Do you know what I mean? Nobody's got an answer for me and I'm like "Okay." So- but still it helped then from that moment on, yes. But before that, no way it was just bad, it was just bad.

INT2: I think that was all the questions that I've got, thank you [INT1] if you wanted to move on?

[Runs through scales]

INT1: Okay, I had trouble falling or staying asleep because I was thinking about COVID?

RES: No, I slept brilliant. That's another thing when I get stressed I sleep like a baby. When I am relaxed I jump up and my sleep it's bizarre.

INT1: Must be a strange thing to think about which one you would prefer? [Both laugh]

RES: Exactly. I look forward to stressful times sometimes, just so I can sleep... sorry I had trouble falling asleep? No I slept well, I slept well so.

Interviewer(s): ***INT1***
INT2

Respondent(s): RES (P21)

INT1
I mean I'm just gonna ask a very broad question now but could you just tell me about the nature of your brain injury? How it happened, when it happened?

RES
Yeah, okay, so it was literally days before my 15th birthday it was the 16th of January 2012. I was walking along the pavement I don't remember the incident at all. A car was speeding and spun out mounted the pavement and took me out with it. I was fully unconscious when paramedics arrived and I was rushed to Addenbrooke's Hospital in Cambridgeshire and induced into a coma. As I say, it was days before my 15th birthday in 2012. And the injury Sorry I got it from my neuro psych not long ago, actually the injuries showed up as frontal lobe and sub jural hematoma. And effects to the microphonic Sorry,

INT1
Was that the hypothermic hypothalamic pituitary.

RES
That's the one yep that thing, the hormone thing.

INT1
Yep

RES
And so yeah, I was induced into a coma. And I had the metal stent type things pop in the right hand side of my head to balance the pressure, I think is how they explain it to me

INT1
Yep

RES
Yeah. And so yeah, it was all quite, quite traumatic.

INT1
Yeah. it sounds it absolutely. In relation to the coma. And how long were you? Did you lose any? So you may not have known but did you? You must have lost consciousness? I guess.

RES
Yeah I'd lost consciousness. And then I never fully was with it. I was taken to a local hospital in Hertfordshire initially, because the other person who was with me luckily didn't get the full impact of

the car. But because she was up and walking, I think they initially assumed that I wouldn't have been so bad. But the scans at the local hospital on the fact that I never properly came around, and made them then rushed me straight up to Addenbrooke's in Cambridgeshire, to induce into the coma, because the swelling and the bleeding was just too significant at the time.

INT1
How long were you in a coma for?

RES
I believe that was just under one week,

INT1
Right OK .

RES
And when I came around, I wasn't sort of fully round. I was just sort of drifting in and out of sleep for quite a while. And, and obviously the, the talk and speech and language therapy and things like that all had to happen, the physio therapy, etc.

INT1
I was gonna say 15 years old is such a transitional period in life as well. It's such a transitional age.

RES
Well I wasn’t even 15 when it happened, it was just before my 15th birthday for I missed the whole birthday for that. So every year I've made up for an extra birthday.

INT1
Good, so you should. So how you spoke about sort of speech and language and I guess in physical rehabilitation as well, what other things were affected or what other things have been affected as you you've gone through, as you've seen as the years have gone on

RES
Yeah so far, I think it's actually been okay, but I had developed a stutter when I was going through the language therapy. And these days, I must admit, I recovered remarkably well. And the stutter only tends to come on now. When I get a bit stressed or tired, but it used to be a lot more permanent when I was first sort of coming round. Again, it doesn't happen quite as often in the past two years, but I will, I would have been using, I would find it hard to find the correct word. I knew what word I wanted to use, but sometimes I would end up using a word that sounded very similar to that word, but the definition of it was entirely different. And memory obviously was and was still is to be honest, facial recognition nowadays. Like tomorrow if somebody asked me what you guys look like I would not be able to describe you at all.

INT1
Might be for the best REDACTED.

RES

So yeah, like memory and things. I have to write everything down to be honest I've alarms set for all different types of things. And my visual perception, there's still some issues with that I have. So my eyesight is not a problem at all. I've had the scans etc. The eyes are very, very healthy in fact is what the optician say. And, and from a legal perspective, I wouldn't even need glasses for driving because I have been allowed to drive for the past three years now. Automatic only though.

INT1
Yeah

RES
And oh, sorry. The reason for that just reminded me the reason for the automatic. The left hand side of my body has weaknesses, and still not to a severe extent that it will dramatically affect my life anymore. But when I was sort of relearning to walk, after the accident, I would, I would be dragging my leg, but like only one of them, but I didn't, it seemed to them that I didn't realize I was dragging it because they was telling me to lift my leg and I was shouting at them that I was, but I thought I was doing things. And it even nowadays, I have personal training sessions. And it's like, I'm, it should be doing something. But then the personal trainer will say “oh REDACTED” but I didn't know that. That hasn't happened, if that makes sense.

INT1
Yeah, Like proprioception and stuff. So were you that you, you think you're doing it, but actually, your body actually isn't reacting the way

RES
Yeah, it hasn't. It hasn't but it's certainly not as significant as it used to be. It's all like the balance, etc. A lot. I get a bit wobbly still. Like, even nowadays, but it's certainly not as it was in the initial years of recovery, I guess.

INT1
Yeah. I mean, it was such a, it was obviously quite a long time ago now. But you spoke about the rehabilitation you experienced as well. Was there anything that stands out in your memory as something that was really positive, like a positive experience or positive about rehabilitation? Or things that helped your rehabilitation at the time?

RES
No, at the time, to be honest with you, I it's only on reflection, it's only on looking back that I realize actually how bad I was at the time. I was absolutely adamant that I was fine. And that all these people were in my life and they didn't need to be. And it was when they pointed out sort of flaws to me if you like I would turn around I'd say no, that's you like you're stressing me out. And I would actually blame it on the professionals that looking back on reflection, as I said, like, I see things a lot differently now than I was at the time at the time. I just, I just wanted to be me again. I just want it to be teenager running riot, I guess.

INT1
So is there, have you reached a certain level of acceptance? I guess that's been reached or adaptation?

RES
Absolutely. And I do I I wish back then I'm not sure whether perhaps it was my age or maturity level because my brain was so sort of distorted if you like at the time that I didn't realize. But looking back there was like significant things that I didn't help myself either because I wouldn't engage enough with professionals because I would tell them I don't need their help and that I just wanted to get back to school. I just wanted to take my GCSEs and just be me again and do what I did but

INT1
in so just moving on from the brain injury generally, in terms of you've actually spoken about you told us about your dog and cat who absolutely apply to this in terms of your support network, and sort of the people around you. if we just think about the last sort of 15 months or so the pandemic itself during the during the lockdowns who have you seen and who's helped you over that period of time.

RES
Okay, so when so I started shielding myself if you like, before the first lockdown happened. I'd been monitoring the Coronavirus from December onwards December 19. And it made me very very anxious to be honest, the whole situation of it. And I had an ex colleague of mine, when lockdown finally did happen. The like this ex colleague of mine she was contacting me like every single day, bless her heart. Like I wasn't going shopping or anything she would be bringing shopping to me. But other than that, I And like I said, I went nowhere near her like I was absolutely, like so overwhelmed by the whole virus thing. I didn't go near anyone. Even my dog the poor sod like I wouldn't even walk him until what night time when no one was around and kor he must have thought I turned into a vampire. But yes, I kept in contact via like social media and text message with what my friends and things like I do have some good friends around, etc. But yeah, to actually see people that that didn't really happen for months, to be honest with you. And to be honest, I think that actually helped me and to really I think, for you guys, this is gonna sound a lot different to perhaps other people you spoke to, cos you know, a lot of people found lockdown difficult, but for me, especially the first lockdown when it was all very serious and nobody went anywhere I when it was, first sort of announced on the telly, I was really sad. and I suppose I gave up for a few days. And I just thought, what this is it now like everyone's gonna die the world's ending. But then I don’t know something triggered in my head, you know that I can run away from everyone. Now nothing's expected of me because the problem is with me, because I'd look so okay. Since my injury, especially in the past two years, I look, okay, is everything that's sort of there, it's sort of a lot more hidden now like to do my brain injury, it's a lot more hidden.

INT1
Yeah

RES
So in terms of like, the general public, I'm just a normal person, and I should do normal things. So things are expected of me like I should be going out doing this all the time for long hours and working all the time. And that's what people expected me because I'd look okay, I just look like the normal sort of individual, I guess. But then suddenly, lockdown happens, there was no expectations anymore. There was no one expected me to go get another job, because I'd recently come out of work a few months prior. And I didn't have to go anywhere. There weren't people sort of saying, oh, come out, Mel. Come on, you haven't seen us for ages, like, come out. But even if I was feeling really tired, I didn't have to do that anymore. Everything was fine. And I was just at home, and I could just be me. And if I was a bit tired, I could have a nap. And that was fine. But then I also started. Like, I started

focusing on myself a lot during the first lockdown. And like the Nintendo Wii, do you know, the Wii fit from the Nintendo Wii. I started using that. And I was using that for hours every day. And to be honest, like there's all yoga and things like that on there. And when I first started with that, I remembered why years prior i'd stopped doing it because I wasn't, I wasn't active anymore. And I did have balance issues from the brain injury. And that's why I'd stopped like doing it because it used to frustrate me because I used to be very, like athletic at school if you like. But then I, I'm not sure why but I just stuck with it in the first lockdown. And, and as the months went by, and even when lockdown ended, I decided that Id enjoyed myself so much hidden at home, that I stayed in that home for a while, like doing my thing. And I was doing a lot of brain injury research and I was finding other survivors too and speaking to them. And I've never done that in the past, I'd never done any of this. And, like over time, like I realized that it was actually helping my balance. And I didn't know I really did sort of find myself and I did manage to realize a lot about myself that I'd perhaps ignored over the years are really like the brain injury and if even perhaps like emotional matters regarding the accident and the brain injury itself. So it was quite good for me,

INT1
Yeah, sounds it Yeah, it really does sound it. do you feel like it? One thing I want to come back to is I want to, I'll be darting around a little bit based on what you've just said there. And Oh god I just lost my train of thought. Oh no, I'll start with what I was going to say honestly this happens to me all the time. I'll start with what's gonna say before so it's interesting that you spoke about Firstly, you put so eloquently and I think there's something we've heard quite a lot over the course of talking to numerous brain injury survivors. But the way that you worded that the idea of expectation is really interesting. One thing that I wanted to touch on a bit more was you spoke about sort of finding other survivors in this period of time. And talking to other survivors. So one thing I wanted to sort of ask a bit more about that, in a broader sense is sort of how did you how did you do that? So it was it was a case of doing over social media or

RES
Yes

INT1
reach out.

RES
So on Facebook, there is a private group that I'm in. And it has 15.3k members. And it's sort of like, worldwide but English spoken people. And so none of my friends or anything, know that I'm part of this group or anything, because like it is sort of private and hidden to the members only. And to be honest, I can't even remember how I managed to be part of this group, because I had to be invited from someone else. But as I said, but over the years, I've never tried to communicate with any other sort of survivors and hear from them. But yeah, there's, like i said, there's 15,000 people on there. So a lot of things are posted and discussed. And, and it's like that there's a wide range of people, there's people that clearly didn't recover, perhaps as well as I have. But then there's people very similar to like myself also. And, like, they comment that they look okay, to the outer world, they look okay, but they just, they're not functioning like there was beforehand, but other people just can't see that. And they can't just have .Sorry

INT1
no, no, that's fine. Take some time, at any point.

RES
They can't just have like, a full time job. and manage everything else. Along with that. I don't know So it was it's been on this group, it's sort of I began writing my book, if you like, a book might like my story of it. But I've nowhere near complete it yet. I've taken a break from in the past couple of months, to be honest, but I'm writing it in a way that the reader unless they're fully educated in traumatic brain injuries, they would have no idea that this character has a brain injury, because that's what it was like, at the time, you know, during my recovery when I was sort of up on my feet again. And I was back at school part time. And I used to get accused of being drunk or being on drugs. And that was never the case at all. And but because I wasn't like that 100% of the time, people thought that I had been using alcohol and drugs because you know, like, a couple of hours before that I might have seemed a bit more normal.

INT1
Yeah. It's um, it's the case of the hidden disability, isn't it?

RES
Yeah. Yeah.

INT1
Yeah, And the other thing I did remember what I would did want to ask you is what the other thing was based on what you were saying before, it sounded like lockdown, potentially, at first. It was obviously quite a daunting experience and quite an anxiety inducing experience. But it sounded like the more that it went on, it gave you the opportunity and space, I guess to actually explore yourself a bit more or or freedom to go sorry REDACTED

RES
Sorry, sorry, I was just agreeing I was saying yeah definitely. So yeah,

INT1
no, no, no, absolutely no. Just the freedoms I guess to a acclimatize to yourself a bit more.

RES
Yeah, yes.

INT1
Okay. And so in terms of just change topic again slightly in terms of your daily social activity, I guess now compared to during the lockdown did that did that change? So obviously at first you were shielding. But now you were talking about your role in the vaccination center as well. So you are going out a bit more now you are you?

RES
Yeah, yeah, I am. So actually, like, go and do my shopping now. But, and this has nothing to do with a pandemic. This is just to do my brain injury. I don't. Even before the pandemic I would avoid busy times in shops. And I just I could not cope with that. So even. But what one issue I do have regarding the pandemic is that Tesco's used to be 24 hours. And since the pandemic, they stopped that. And so now it's never, I can never go there at a time where it is, you know, entirely quiet, and I am the only

shopper there. But it's not so bad if I head there about 10 or 11 is okay, it's a lot quieter, but it's just like since my brain but I've had severe like sleep issues since my brain injury too. it And yet, it's a bit of a crazy one, it can it fluctuates, sometimes I sleep too much. Sometimes I just cannot sleep at all. It's like a stage of insomnia, I guess.

INT1
Yeah

RES
And but when it was that sort of stage that I would head off, to go shopping at 2 3 in the morning, and then I wouldn't be overwhelmed. And there wasn't all these people around. It was just workers filling the shelves. And that was great. But then because of the pandemic, the shops are no longer 24 seven, they close at 11 or 12. And so that's not quite the same. But yeah, I do. So last weekend was the first time I went back to a pub, like properly. And it was for the England game as well, actually.

INT1
It's a really busy time then.

RES
Yes. But I was I, I looked into it first. And I was very particular on the pub that I chose. And the pub that I chose is managed by two people that are very near retirement age. They're quite elderly people. And before the lockdown first lockdown ever happened, they was very, very strict on everything. Anyway, which I had noted before the lockdown. And so that was probably the best venue for me to go to because I knew that with all the restrictions, etc. They was very, and they're very fussy about who they let in as well. So I knew it wasn't just going to be overrun with a load of rowdy football hooligans. In fact, I was probably the only football hooligan there. But no, no, it was okay. So it wasn't, like I said, because of the walls, etc, that they do have in place. It wasn't overcrowded, etc, as other venues were. But yeah, it's just I used to find busy environments hard because my brain injury before the pandemic. But then I think because I had such a long time away from people and like big groups of people etc. I don't think that helped probably that has made it a bit harder to sort of readjust back to the work at the vaccination center, and the first three months in particular was very overwhelming for me. There was so many times I just ended up walking out and I just couldn't be there. My migraines started during my work with the vaccination center as well. The migraines had sort of eased off for quite a long time, to be honest, but since starting my work at the vaccination Centre in December 2020 and that that will start again and my emotions were effect I was just overwhelmed. I was very overwhelmed and I just I couldn’t I couldn’t handle it to be honest. And that's why that job came to an end anyway.

INT1
Yeah

RES
But

INT1

you're so with your I'm trying to phrase this in the right way with your level of activity and the things that the activities that you do, has that changed pre pandemic. So obviously, I'm talking prior to when December 2019 when you initially started to shield and stuff.

RES
So I since lockdown was the as I said after the first lockdown, and I was still avoiding people as much as possible, but then over time I did eventually start going out by myself but not with not with people at all. I became an annual member for a local wildlife park

INT1
right

RES
near to me and, There was one, there was one week only, it was a members only week and I had gone to Paris wildlife park by every single day in that members only week. And I still like to this day, I go very regularly and I was very upset in December just gone when lockdown to my area, then it was tears then I think, happened again. Because even though I understood about the whole public health thing about the virus and whatever, I just couldn't quite fathom why walking around and outdoor Wildlife Park was such a risk, you know, if the numbers were monitored, etc, as they always did, and that that was taken away from me. So again, I just to be honest from it was probably the end of 2020, I'd had an absolute, like, I'd had enough of the lockdown, etc. Because I did start finding things such as paris wildlife park that whether I was with people by myself, I could go to and I felt, you know, and that made me feel better. And I was still out in public. But even if I was by myself, you know, like I was still in it just it made me feel I dunno , it was just, yeah, that the last lockdown for me was just my, the breaking point for me, to be honest, that was that was that I'd had enough and I couldn't. No matter how hard I tried, I couldn't be as happy as I was in the first lockdown. I think because I was sort of given that sense of freedom again. And like I said, compared to other people, it took me a long time to actually get back into that.

INT1
Yep

RES
But I think that's why I pushed myself to start working for the vaccination Centre in December 2020. Because I knew I needed like something. Why and I know it sounds really silly, but I didn't have the wildlife part to go to any more. I didn't have Woburn Safari to drive around in my car anymore. And I just I needed something and I needed like even at places like paradise went out like Paris wildlife park when I went by myself. Even though I was by myself, there was still sort of staff interaction and especially the lady at a coffee stall place. She recognized me. And like she has a little chat with me every time and so that yeah, it was that the last lockdown for me was just what that was that and I I'm not sure whether I would have made it through if the lockdown last any longer to you. And I'm sorry, I know that sounds a bit dramatic. But that's the point I was getting into I guess last night.

INT1
No not at all, So thinking about lockdown generally then, and I'm using the term lockdown to describe effectively the past 14 15 months so you can interpret that word however you want to. What would you say has helped you? The most through that?

RES
Sorry, what's the question sorry?

INT1
So if we so I'm using lockdown as a term to describe, I guess the pandemic in a sense right, the past sort of 14 15 months of reduced activity or reduce social activity? What would you say has helped you the most through that period of time?
RES
Sorry do you mind if I just take five minutes or something

INT1
Yes, of course. Of course. Yeah. And you wanna join and leave the chat or do you want to come back in when the link should still work and everything. So you can feel free to leave and come back if you'd like me and Sam will still be here or you can just we can just stay here

RES
I'll just switch off my mic. I'm just gonna make a cup of tea or something

INT1
Yeah, no problem at all. No problem.

INT2
Been very thorough so far

INT1
Aye?

INT2
Been very thorough so far like, the longer one your good at getting into advances

INT1
Yeah its good

INT2
I've actually got some notes I can ask as well

INT1
Have you? Good. id say there's definitely probably a lot of stuff that I have missed. Oh, that's okay. Hopefully. Hopefully she'll come back. I can you see REDACTED's face?

INT2
No

INT1
no. Okay, good. Neither can I, I obviously I can hear that she was crying but I couldn't. I didn't sometimes what happens is that my computer, this has happened for a call for me and REDACTED once where the REDACTED could see the person's camera I couldn't. And so like the person was like

showing things to the camera. And I was like, Alright, yeah, and I said to REDACTED at the end, but I didn't know what was going on. And she was like, Hi. See, I just couldn't. So I just wondered whether that happened again. And I was like, I was literally just going off of audio cues, what was happening, but I know it must be really stressful for REDACTED. I'm just trying to be mindful of that as well.

INT2
Have we had any situations like this before? And they either have or haven't come back?

INT1
Yeah. So not a situation where they needed a break. So I've had people obviously crying and people emotional, but I've not had people needing to leave and come back. That's absolutely fine. If that happens, then so be it. As part of the part of the ethics, right? Yeah. REDACTED can choose to withdraw. When if she wants to, hopefully, she'll come back. And we can continue chatting, nearly there. And I'm just going to move on the reason she's talking about lockdown. So I thought I'd just push on with lockdown. So I was just going to talk about what was most difficult, what's been most helpful, and how she feels about getting back into society and then circle back to loneliness instead. And so this is the point probably where I don't know whether you want because I've not got the FOMO questions on my interview thing. So I don't know if you when usually are you asking it directly?

INT2
Usually it's usually just the question about how you feel about lockdown. lockdown ending and opening backup and getting back to. So just comes credit coming. Do you ask it directly? Or is it just that question? It's just asked that question. Basically, we don't specifically mention FOMO.

INT1
that's fine. That's fine,

INT2
just as I'm thinking about my proposal, that I need to get done at the weekend.

INT1
Yeah

INT2
If I'm doing the loneliness measure the resilience measure and the formal measure, in my questionnaire. But then I'm also collecting like, age, gender. What are we collecting? Basically, all the demographics that we've got in the spreadsheet?

INT1
Yeah, pretty much.

INT2
Would that turn into a regression, or would it still potentially be a MANOVA will become a regression like that, wouldn't it?

INT1
From my perspective, I think you've got a regression, I think, I think it makes it easier for regression as well, because what you can do is you're effectively looking at predictors of loneliness. And if

loneliness is your variable, I would just like, I would choose one, one of the loneliness measures for the regression. And then you can affectively have a separate analysis, looking at the validity of the loneliness, the different loneliness measures, which can be like a nice little ANOVA or something on its own. Because you have those loneliness measures, but I would choose one of the loneliness measures and have that as the premier loneliness, measure, then have that as your loneliness score. From that loneliness score, that's your, your variable, you're looking at what predicts that? And then I would use things like age to

INT2
Topic it all

INT1
Yeah, exactly. Yeah.

INT2
Yeah. So FOMO

INT1
FOMO. Exactly. Yeah.

INT2
As I've been listening to this, I was thinking, probably for the best I didn't take the lead with this one.

INT1
Yeah it All right honestly its good, mate. Every time I've tried to do one of these for the past three weeks, no one showed up. So it's nice to get back and do one. And I say that REDACTED might not come back but

INT2
with the last one that I did. I think it still ended up going well, but it was one that he could have gone off the rails because I there was this guy, he basically oh she's back.

INT1
can we talk about what it is? I'll give you a call back after this. After we end this meet? I'll give you a call, we can debrief.

INT2
Cool

INT1
Hi REDACTED are you okay?
RES
yeah Sorry, is bad news for the moment. Now, that was all

INT1
no need to apologize at all. One thing I should have made clear from the start is that you can take as many breaks as you want. I don't want to waste your whole day either, though. So we get I'll try and speed things up as well. But, and obviously, feel free to take as much time as you want, just let us

know. And, yeah, we can obviously schedule any breaks. We can even potentially split the interview if you want to do it another time as well. So just let us know how you go.

RES
Yeah, no no. I'll be fine. Now see, I just needed a minute to think?

INT1
Perfect, no problem at all. So we were talking about lockdown generally. And, and one thing that I was asking was sort of if we think of lockdown as the past 14 15 months of sort of, you know, the social restrictions and diminished activity effectively, not being able to see people what would what would be the thing or the things that you would say has helped you get through that period of time?

RES
Oh, my phone, I guess and my bit, like being able to contact people still wonderful, when we weren't allowed to actually see them. But then like I said, even when we was allowed to meet up with people i still chose not to. But I still spoke to them, I still kept in regular contact with people etc. And I, I just, I took it upon myself to keep myself busy. As I said, with starting to use the, the Wii fit board Nintendo Wii and I hadn't used it for years. And then I started, as I said, making contact with that. And what other brain injury survivors and speaking with them, and then ai was sort of, I kept myself busy with other things, too, but I was doing a lot of reading in that time. And reading for me, since the brain injury, I'd, I know it was back in 2012. But to be honest, I eventually over time, I just, I stopped reading books, etc. Because I couldn't, I couldn't follow it. And it was going on for like a long time, like for a few years, I just I could not follow a book. So I just I gave up all together. Because I didn't want the reminder that I still can't do that. And that I felt like an imbecile because I couldn't read a whole book and know what was going on. And remember the characters from what was what. And so reading books, etc, for me, and only started up again, in 2020, because of the lockdown. And I still, it's still not as easy as it used to be a prior brain injury when I was 14. But I found my own ways of dealing with that now. So when, when I have had enough of reading, I will make notes myself of like things that have happened, and who's who. And I'll put it in the books. And then when I do go back to it, I can refresh my memory. So then when I'm reading, I'm not feeling like I'm reading something brand new that and I have no idea what's gone, what's been going on beforehand.

INT1
Yeah

RES
So I suppose I learned to adapt and use skills to do things such as reading books that for a long time, I had literally just given up on them due to the brain injury effects

INT1
That sounds like you've got some sort of really good coping strategies for actually doing these things that you want to do, but maybe hampered in doing.

RES
Yeah. And it only took nine years to get there, aye

INT1

Yes, it’s a process it’s a process. And I think you've already touched on this. But anyway, I don't want to go over old ground. Because you know, we've already spoken about this. But is there anything aside from what you already told us that you found most difficult over the past sort of 14 15 months?

RES
I am in so I only have to see my neurologist now once a year, because I have recovered quite well. So it's only once a year now. But I was due to see him in the summer, like last summer. And that that that was cancelled due to the pandemic and that he's in central London. And he works Queen square London. And so they just said about the travel etc. Not to go. And for me, I suppose especially because I did discover myself things that are still sort of troubling me. RE, the brain injury I did sort of last summer probably would have been a very good appointment for me. To go to because I did have so much that I had realized myself that I could bring up to him myself that he might have suggestions to help me maybe make referrals to x, y, and Z because like, there are still things such as the, the damage to the hypothalamic, that thing that I believe can sort of not be resolved, but be helped with the right sort of referrals. And that referral hasn't happened yet due to the pandemic, unfortunately. And so that's sort of, I suppose, and caused some issues. So that was probably a negative that's come out of that, I guess.

INT1
Yeah, We're in a position now, where society is starting to get back open. And we, you know, the restrictions are off. To some extent now, anyway, or even though, you know, the Freedom Day has been pushed back slightly. And how do you feel about this sort of life post lockdown about society opening back up?

RES
Oh, well, I'm quite daunted by it. Because as I said, even before the lockdown, like, busy, loud places, was very overwhelming for me. And because everyone has sort of been in prison for so long, I guess. And I know there's gonna it's gonna be like summers, a very busy times anyway. But I think this summer, like later in the summer is going to be absolutely extraordinary. And there's like that, that prospect sort of overwhelms me and I, I perhaps I'm going to be avoiding going out because I know it's going to be even busier than the usual busy summers. And I can never deal with sort of busy summers and places prior pandemic, because of the brain injury stuff. And so I, that's quite, that's quite daunting, I suppose that it's gonna be mayhem for the next couple years.

INT1
Hopefully, it will be controlled. I can understand why you'd be daunted. And the final part of this section before we just move on to some getting you to do some scales, some questionnaire measures. It's just I already kind of explained the project a little bit and the information sheet would have done this. But one thing I want to talk about a little bit is loneliness. So the first question that have in relation to this is how do you define the term loneliness? What does that what does the term loneliness mean to you?

RES
feeling, feeling as though that even if there are people around, they just they do not understand you. Not having people to open up too I guess. It's not just about actually having people in your direct presence. It's just about whether those people make you feel make you feel relevant, I guess. Do you do the people around you makes all relevant? Do you feel? Do you feel wanted?

INT1
Is it a feeling that you've experienced over the past year or so.

RES
to be honest, before we started this meeting, I would have said no, but now I'm actually thinking about it. Yeah, I think I did quite a bit to be honest. Like I said, especially when I was sort of working on myself and realizing that there are still brain injury effects and things that do. I do need to try and work on or perhaps I can't work on that is that now because people in my life are they have for a good few years now. Especially people that I've got to know since I was an adult. So they didn't know me at the time of the brain injury before the brain injury. And they have absolutely no idea about the troubles that I do face. They that they all say "oh, you're so intelligent" like," What? Did the brain injury make you more intelligent? Maybe we should all have a brain injury and be intelligent like you". You know, they just that they can't fathom that actually, I'm not who I was prior the brain injury, and that I can't just live a life like they do. See, yeah, I suppose I did.

INT1
So in relation to because you taught you one thing you said to us is that obviously, you've spoken to some brain injury survivors in lockdown as well. And you have that that group that you have been communicating with? Do you feel that they would understand better than, I guess, you know, the, the other people that you're speaking about in your life who haven't had a brain injury?

RES
Um, yeah. So with this survivor's group thing that I found, I have noticed that I will, but I don't discuss myself to them, and discuss anything about me or myself. And it's just when they write things when they say things, and I will, I sort of respond to them. And I try to suggest ideas to them that they should try or are sort of, I said, I just sort of its, it's, it's nice for me, even though I don't open up to them about myself, it's nice for me to read certain things they write because I do relate to it, if that makes sense. And that that's enough for me, I don't need to talk to them and tell them about my experiences, because it's enough for me that I've read what they're saying. And that actually, it's very similar to things about me now and things about me sort of in the past year in my recovery, etc. So that's sort of been enough for me to, I suppose helped make me feel better, just the fact that, like, there are other people out there who do have the same thoughts that I've had over the years, and perhaps never sort of discussed myself.

INT1
Just kind of like a third party validation, to some extent.

RES
Yeah, yeah.

INT1
And I think that that's all the questions that I have from the interview section. Sam, do you have anything that I've missed, or anything you want to add?

INT2
I don't think you've missed anything. But for something that stood out quite early on in the chat is that when you said about how you kind of started to shield and isolate, before, like, there was

enforced? Did you do you feel like any, like, worries that you have about COVID and wanting to shield and stuff like that has been brought on? by anything to do with your brain injury or

RES
Yeah Yes. So um, with in terms of the brain injury, my concern what because I've been doing a lot of research online for a long time before the first lockdown even happened. And I don't mean research on websites of people that are claiming their doctors, nobody has any idea who they are. I'm very thorough with my research. And, and my concern was the nothing to do with the vaccine. I know, there's all that talk about the vaccine. This was before the vaccine was even brought into use, and the blood clot risk from COVID, etc. And I was concerned that due to the damage I had on my brain, even though it was quite a while ago, perhaps the brain was not as strong as the regular people's brain and that perhaps, if I was to get COVID, and it did affect my oxygen levels significantly, and that perhaps that would have an effect to the brain damage and perhaps bring on a blood clot because I previously had bleeding etc on the brain if that makes sense. That was sort of my way of thinking at the time and the fact that I am quite heavy smoker like cigarettes only sort of concerned me too but the brain injury thing was the big thing for me

INT2
you're not the first person who had a similar worry about that thought I think that's kind of what sprung me like going down that line of questioning basically. Then another thing is kind of just like people understanding your brain injury in general like how you said about how like Sometimes you before the lockdown and stuff you'd have people asking you about it just almost as if I pressuring you to like come out and do stuff if you didn't feel like it. And that was like part of the expectations that disappeared with everyone being locked up. Is that something as well like you well how do you feel about the potential of being asked to do more than you want to do basically by

RES
since the like the last lockdown we've just had I've been a lot more firm I guess. And if I if I'm really not up for something or if I don't fancy doing something I do ensure that I just I say no now and I do either go home or just don't go out in the first place. And like tonight for example im off to be a football hooligan again tonight, England are playing again. And my friend sort of had ideas in that in her head that after the game we was going to go off go out to a party town if you like, that's fairly nearby to us. And we was going to be out until God knows what hour in the morning and I just likeI don't I don't want that for me because I know I know I'll struggle with it and like forget the hangover effect, but I just mean I know that I'm going to be very overwhelmed as a few hours get on into the night and there probably will be a headache. migraine brewing during the night Like I said nothing to the hangover the next day. I just mean in the night because I've been out for a few hours etc and concentrating on the telly watching the game for a while I do know that I'm going to be feeling a bit and with or without alcohol. If I've been out for a few hours I do get very like wobbly on my feet. Obviously alcohol accelerates that but even without alcohol that would happen. So and I've been very firm and I've told her that the end of the game I'm going home and she's walking to go off and do whatever she likes. Whereas prior lockdown I would try to I would go along with that and I would try to be like my friends and just try to have like the whole night out etc and that would never sort of go well so it's not the best thing for me so since the last lockdown I have been very firm I've not allowed people to sort of push me into that anymore

INT2

yeah, I've got say that's something I've also not missed about lockdown as well because my mates would always be “ohh lets go out for a few hours and it would always end up as a night out” and then id be like “no im going home” But um what is it you think it is about lockdown it's actually made you become more firm like there's if it's like had some sort of like empowering effect on you just be able to be blunt with your friends.

RES
I suppose because of the first ever lockdown the first initial lockdown when it all happened. I was I got myself to a point where I was feeling a lot healthier physically and mentally. And because I had focused on myself and I was working on myself. And I suppose I had a lot of a lot of time by myself to consider everything and to properly go over everything about the brain injury and the accident that happened and I suppose finally being able to start accepting that. So I was just in such a better place mentally during the first lockdown. And so I think like now because it did get a little bit squirrely towards the end of last year again. And I wasn't I suppose looking after myself as I should have been. I suppose. This this time I've decided that no like I felt really good in the first look down and even though, I am trying to integrate myself back into life. I'm going to do it on my terms. And I'm going to do what's right for me this

INT2
That’s really good and positive to hear, right. That's all my questions so ill pass you back over to REDACTED.

Interviewer(s) **INT1**

INT2

Respondent RES (P22)

INT1 So can you tell us a little bit about your accident and sort of the brain injury and how it's affected you?

RES The accident was, you're probably not familiar with them, but they were involved Acrows, which are tubular metal supports when in construction, putting the lintel above a window frame say . For example, you put these the tubular with the heavy threaded section on the top, you've seen you put them underneath a particular item, you wind them up, and they support a lintel doorframe, or whatever they weigh eighty five pounds each. On this particular day, a load of them have been piled up in a corner, this shouldn't have been, they should have been laid down on the floor. The job that I was going to look I would look at it being cancelled by an hour. My boss came in. I told him what had happened. I'm not one for standing round, and or kicking my heels. So, I said, well, boss, look, I'll go in the room I'll sort out them Acrows we'll talk about it for a while. He agreed with me. I woke up several hours later in a hospital. That's what I remembered. Well, what had been explained to me was it looked like I moved three of these items, put them onto a piece of flat ground we were standing upright, was a small four foot one. Mangled amongst the others, they'd all been chucked against the wall. As I moved this one out, it clipped three of them, they came over, smashed against the left side of my head, smashed me up against the wall, which in turn I hit the ground to get the damage was done, obviously 3 at 85 erm I had a massive whiplash to my left hand side, where my head with the weight of these smashed against the wall on my right hand side another massive blow. And then obviously ultimately ending up on the ground. In a bit of a mess. Right, we'll leave the damage there. So I end up waking up in the hospital. not normally if I'm Arthur or Martha and a right state. erm And … just a mess. We'll leave it there and I end up back at home after a few few days. In my head, there's nothing wrong with me. Yeah, I've got scars and this happened to that. And a touch of the Elephant Man my head had swollen that. I'm okay. Two weeks I'm back at work. No problem. I'm back at work. After two weeks, I realise now that two weeks of work and I've got all these specialists people telling me you are in a bad state. It's a life changing accident. What if What do Doctors and nurses know eh? Those know nothing? Oh, I wish I'd listened. So another two weeks. Another two weeks. Esta manyana I'm back at work. Come on. I'm actually trying to go out because I'm sports oriented. Play jujitsu, I surf. I know a 60 odd on a skate board. It's a joke, but I was so. Okay. Next month. I'll be back at work. And I'm going to Monkton in the psychologists saying here REDACTED get a grip get a grip. So after six months me, time runs out with a psychologist along Wearmouth. I've got nobody. And then somebody says Look, there's a Headway charity go to the Headway. And I go to this Headway charity. And I'm ashamed of this. Bear with me. I see all these people are really badly damaged. I'm not one of them. I mean, I'm in denial, I didn't know I was in denial but i'm in, denial. I'm not

going there. You know? And then this lovely lady called Charlotte (emotional) said to me, come here, takes me to one side speaks to me like nobody else had spoken to me before about invisible injuries. Just because I can walk and I can talk, I'm fairly agile, It doesn't say I haven't got a brain injury, you know. And she says, just come and sit and watch them. And then when everybody gets up and goes for a coffee, that's me, look, they can walk and talk and they're. yeah That's me. So I decided to go and meet this erm this girl, girl, God forty odd this woman, because everybody is younger than me you know. So here, we get them talking. This is Kim and Charlotte the sneaky clever buggars ya know they put her with me. And after 10 minutes her names, I won't say I'm learning not to mention people's names. She tells me I asked you here and I knew a guy. When when I was younger, I used to do a bit amateur boxing. And I knew this guy, and his name was blah, blah. That's me, dad. I went You know what? that's me, dad. So we'll have summat in common. And then she says what you're doing tomorrow says nothing actually stay in the house. So she comes (emotional).... in the door and she's says do you sing? I say why do you say that. She says i'm looking in your conservatory, you've got a ukulele. You've got sheet music. I said, well I don't know about singing but I make a hell of a noise. She says, I'm i'm. I'm a fairly good singer. And I said, are you? I have met loads that fairly good singers in my life. So I said, look, I've got me Ipad I've got me erm…. bluetooth speaker and set it up. And I'm scrolling through, she says what you singing? I says I just do like REDACTEDny Cash. And Leonard (unclear 07.03), I love REDACTEDny Cash. And says, Can you play it on the ukulele I says I can do a little bit. Which she says go ne son. So it's False in Prison blues. God I've got at a Staffordshire Bull Terrier can sing that. So it's not that hard. So (unclear 07.18). Come on, we'll go. That's quite good. I see what you do. And she looks through she goes Oh, Aretha Franklin, don't play that song for me. And I remember thinking she's not do a good version of that. I thought for you, you must be some singer. You can so I set it all up. And do you know the , do you guys know the song? Right? You know how it starts off doing ding de ding ding. I'm standing next to her and she sings as I'm telling you and the hairs on the back of the neck. So I've met somebody. So she opened the door to brain injury, she had a tumour in her brain cut all the top of her head off. So I've got a buddy. It took us six months for me to get acceptance, to realise I'm never ever, ever….. going to skateboard again. Isn't that pathetic? I'm 65 I'm 60 I was 65 when accepted. I'm never gonna skateboard again. I'm never gonna surf again. I cannot play the ukulele again. And I cannot make a noise shout again because I can't hold a REDACTED. So, acceptances come in, we'll get into the programme Headways great I've two years there. Then we'll come to the issue. lockdown.. it was horrible. I lost everybody and everything. I still speak to them. I still have erm… REDACTED and but I also lost another side. We've got monies from the insurance company. I started rehab. I was loving that. That all stopped. I basically have the little life I built for myself for about 18 months, two years. Gone. That was terrible. I don't like the public. The public have no understanding of brain injury. I have difficulty. I have a little network, I live a quarter of mile away from an Asda superstore. I know people who work in it, the early days I forgot how to use by bankers card ohhoo for your press green, at the end of it totally forgot I knew I had 1000s of pounds in the account until one of the girls so beautifully come up to me and just whispered in my ear press green. I learned how to use my car to get I was so stubborn and pigheaded. I didn't want my wife coming with me all the time. My son come, you took two weeks off work from London. He is head of the department head of Middlesex University. He took two weeks roles had reversed I'd become the child you know and pride. Oh I've always been a very proud person. That doesn't help if you have a brain injury take your pride away put it in a suitcase and put it in the wardrobe for a while because that cramps your style. So my rehab was buggered up

the lockdown came in the April/May. I got very angry with them because let's go in the carpark and do it let's go in the car, they wouldn't have that I did 107 pounds an hour for rehab, that, yes that got right up my nose and when im paying somebody 107 pounds an hour with respect. I expect them to do a little bit of what I'm asking them and why can't, the car park was 23 metres by 40 metres Come on. So, I walk away from them and I found another rehab company neural pathways and those those continued with it with a lockdown so that was good. And but there was only one person allowed the time at the time. And that was fantastic But I'm restricted to no more than a handful of people. So great. The public are going to Asda between quarter seven and seven o'clock in the morning. Cannot cannot do crowds. One of my biggest fears is Joe Bloggs comes up and speaks to me. That's quite em awkward. It's awkward for two reasons. The first reason is that I don't always get the gist of what somebody says. I suffer. My vestibular system are you fam are you aware of my vestibular system in this side is no my little canal in my ear. Yes, it's a boomerang now. So this is and when I walk I veer to the left all the time, which a lot of people think I'm a little bit drunk because three or four steps if I hadn't got a fixed point that I can focus on have to do a little bit of a shuffle to get back on to my, a lot of people see that. And I know they're thinking I'm either drunk or stoned or whatever, you know, and you see the looks and all that. So I have a tendency sunglasses on, focused on a fixed point, and I just go for it. I don't like it. If somebody comes up and then tries to engage in a conversation. I'm learning to tolerate a quip or a little bit of a remark or a passing thing. Well that's okay. That's okay. But don't come up to us and say "eee have you seen the price of that such and such or whatever". And I feel crap about it because before my accident, I was the most sociable gregarious, outgoing gob on a stick could hope to meet my job is a site manager in charge of 250 people, 10 or 12 different trades. I was never a man with a stick, I was a man with a charming attitude that could get the best out of every one of these tradesmen. One of the nicest things that was ever said about me was when I sack somebody. I met him two days later, he says was a pleasure to be sacked by you. And that was You know ,erm some people are good, some people aren't good. Get rid of them as fast as you can, as nice as you can. So I don't end up in a shouting match it spoils my day, and he goes away and get another job without feeling crap. You know, you tried to do things, the days of aghhh rage on buildings sites finished, you know. So I don't want to say I don't like the public anymore. I like to pick and choose who I give my time to people who understand me. Because sometimes when I talk, you're going to find that this is the time goes erm on I have this pathological fatigue. When I get anxious, when I get tired, my left eye goes and, and I studder and I stumbled. Alright. erm I, I the lockdown I love it. REDACTED loves it. I've been able to keep in touch with people for headway. Great. Because it's. My psychologist who I still see once a month, REDACTED. She saying that a lot of a lot of people with brain injuries are quite comfortable with it. Yeah, I am. And I understand why. I and there's also this psychological, physical thing of the mouth, the best part of the face is hidden. I don't know what that has to do with, REDACTED doesn't know what to do with this. But especially the girls, girls are comfortable go out covering the faces. I don't know if yous have an answer answer for it.

INT1 **No. And I don't have not really looked into sort of their mask wearing or or seen any research on that either to be honest with you. And no sorry.**

RES Does anybody talk

INT1 about

RES recovering? in recovery?

INT1 We've interviewed do you mean?

RES Yeah.

INT1 No, I don't think I can't recall that anybody's mentioned it can use that?

INT2 I don't think so. I think a couple people might have like mentioned like, them being like off putting off so know that like for balancing issues or something. But

RES people are like, people don't because the mouth is very important psychologically when people are speaking, you know, your movements are not. And I know, two to two women, especially who are sport who have brain injuries. Erm yeah, they said people have a tendency to back off them. These are loud women, by the way, no inhibitions whatsoever with TBI, because you know, the full Yeah, enjoy. But I I have found that I can honestly say I am more outgoing because there's less people out there since the pandemic than what I was, was before, I'm much more comfortable. And especially, you know, when all the kids and everything what, the schools were closed down, and that I don't know more quieter and more open. Made me feel more comfortable.

INT1 Yeah, I can totally understand that. And you've not been alone in in a lot of the things that you've mentioned today have been things that regularly come up in these interviews and that, you know, we have, you're not alone, you're not going through this. on your own. A lot of people are sort of feeling the same about that sort of new identity that you've having to sort of find in yourself and accepting that life changed and it's not going to be the same as it was so it is hard. It's not an easy process to go through at all. And do you find that I know you said you've changed rehabilitation. Have you found that any of them will helping was the one before locked down Did you find that beneficial?

RES Yeah. Yes, it was. What I should say about that is the erm the actual we call them coaches are brilliant, right, two REDACTED and REDACTED they were in awe of my by determination, my will, etc, etc. They were magnificent people. My (unclear 20.01) were in total agreement with me about the car park, it was the management. Erm and I consequently, there's another story in all of that, but we won't go into it. And, and I was terribly, terribly upset

when I when I left. But as I say at 107 pounds an hour, I feel as if I have a shouting what and those were in total agreement. And when I found out how much more we're getting of that I was even more annoyed but that's by the by. Erm Yeah erm. And when I went to the second place, and I told them saving four pound an hour as well. Yeah. When I went to the second place, that new neural pathways, and em there was a phenomenal lady, and she came to the house and interviewed interviewed me and we talked for an hour. And I said, Can I have a little bit of an input into it? And she said, of course, you know, we need for our clients to have a have an input. And when I went up for one free session erm, it was great. I felt as if I was contributing towards my well being. I also found out she was an ex psychologist so that she knew the game erm I was happy, I was groovy and I'll just digressive a bit. The very first medical person who came to assess, assess me was a doctor, Julian Harrison came up from Kent and he came to my house to knock on the front door, stepped in he put his hand out to shake hands with me. And as I shook hands with him, he immediately wrapped his other hand round my hand. He says your hands are freezing. REDACTED, have you been out the house? I said I haven't been out the house at all. He says has your endocrine system been checked? He says because your hands are, I says you've been two steps in me house and you're diagnosed he says I know because I'm good. I met these increase absolutely right. Went in his initial report, he said unfortunately in the near future, because of REDACTED's vestibular system is balanced, I'm aware of it and this and that. He will have a fall and it will be a bad fall (unclear 22.22) in he came to my house and he assessed me for three hours. Three hours giving me all sorts of physical tests. Christmas just gone halve past seven in the morning on Christmas Day. As I'm looking forward to my family spending the Christmas together I stepped out the shower. We have a fairly large bathroom. I take two steps I turn in the radiator to grab the the towel and my vestibular system went and as I'm on my way down we know the worst place in the house to fall all I can think of is my 30 years of playing jujitsu get your elbows in getting your chin in the chest, long short of it I broke my foot in five places and fractured the base of my (unclear 23.20) on my leg and all I remember thinking is is I'm lying there my wife will have heard it she's gonna come flying in, the panic, so I just lay there and as she opened the door all I could think of saying was Merry Christmas Pet I'll be in back again. She's what else is just wait look, also we had two friends. My wife's got two gay friends Bet and Jane and those were in the bedroom. What was it I told my wife to go in there and I'm bollock naked and I thought ahh so the lesbians what the hell and I remember crawling on my hands and knees across the landing thinking one of them one and sure enough not one of them come to the landing but both of them Merry Christmas girls I'll be with you in a minute, I got through to the bedroom and me foot was… I'm old enough and ugly enough to have to realise it you do not go to A&E on Christmas Day. So Tramadol.... and vodka got us through the day I was in bed for eight o'clock on Christmas Day. The grandkids had to kick the foot as well didn't they, during the course of the day. And the son picked me up at six o'clock on Boxing Day. I was in I was tret I was plastered and I was back home for eight o'clock. But painkillers and everything. But that goes to show how good Julian was leading back to the story it couldn't do physio. The treatment I was receiving was called a sympathetic conservative treatment, they put half a cast on it and I promised to do nothing for seven weeks or so apart from lie on the couch. So I ended up with blisters on me backside with lying on the couch that in turn stopped my rehab it neuro rehab it neural pathways which in turn kept me in the house for seven weeks. When it comes to going out again, the left the lockdown had been lifted a little bit, I've got such anxiety I couldn't go in to Asda again, I was back to square one and again, again this brought in and I'm gonna get emotional because I thought I'd got over it I was terrified to I was, me wife had to take us

into ASDA and start all over again. Why? Because I've got a brain injury that's why the brain injury you know, we get muscle memory well the brain injury you don't get a brain memory. I had to, it's your cosmic cognitive and executive had to start all over again. Where does that come from? Well, I'm your brain injury. That's where that's where it comes from...sorry about this. Because this is you get a full on full on emotional lability.

INT1 **You're fine REDACTED absolutely fine You don't need to apologise to us at all. Would you like to take a little moment.**

RES No

INT1 **All right.**

RES No, Cos I'll run away, I've done it before

INT1 **Are you sure you can feel free to run you know, you don't have to stay**

RES Running away It doesn't help anybody because one day this is the thing Nichola's told us what to do. Don't run away. If I run away I've lost and if there's one thing I'm not, not gonna use the word coward because it's not a fight. But I'll face me demons because they've got to be faced, got to be. I've watched too many people over the last three years since I came out of denial and I accepted and I watched other people, you cannot keep on running away. It's, it's you've got to get a grip of it. You know, and me lovely psychologists REDACTED what a woman she is, she you know she said she says look, I go I know now REDACTED because you've got to come back someday. She's helped me fantastically. You know.

INT1 **Has there been anything particular with the rehabilitation that you think has stood out that has helped you?**

RES With Yeah, emm really able to... one good? Well, when I have my accident I was a big lad, 13 and a half stone, height of five foot eight. So really I was nearly as broad as what I was tall. I was the site manager. I was on a lot of money a year. I used to go to a very quiet I used to wear me whistle and me tie depending upon what aspect of if there was meetings or this, but I always carried on I work your old proper me uniform you know we work boots and that. And If we're getting a delivery of 10,000 bricks or blocks, I come out me porter cabin with me work boots on the work gear, and everybody would think what's he doing, he's the site manager. And I work with all the labourers till 10, 15 20,000 bricks were off the van in the car park or taken to wherever we need it on the site. Because on the nighttime I was that wacked? I stopped playing jujitsu I was sick or getting thrown all over the of the coach.

And I was sick of getting through all over the dojo of young 20 year old lads and that, I just used to you get through by hand signals. I was sick of waking up in the morning (noise 29.38). So get fit, work fit. You know, and that's a 13 and a half store. Now I find over the years I'm down to eleven stone, two pounds yesterday. That's a hell of a, hell of a difference. I don't have, Sam you pro? How heavy how heavy are you Sam?

INT2 ***I don't know, I haven't weighed myself since the start of lockdown So I'm like, I don't want to find out how heavy I am. I think I think last time I got weighed I was about 11, 12 and a half. But I'm about six foot. I'm probably close to 13 now***

RES 13 alright so yeah your (unclear 30.30) tall, thin. And I knew it's very difficult if you've been a substantial weight. But what I'm trying to work was a psychological thing for you to understand, go from a.... very confident, solid lump of a guy to where me wife's got to link in with us to stop the windblowning us over. Dealing with that psychological thing in your head is well it's the whole transformation of me life is what I'm trying to portray to you and I'm not very articulate. My whole thing was my verbal ability my physical presence, I had a nice and people used to like me, I've lost all me erm… persona my who I was, which I've been assured is what happens.... you know the erm.... talking about the... it's coming the woman who comes to see me… god I've mentioned her

INT1 **Is it REDACTED**

RES that's it that ee how long we all been talking? 30 minutes. And she's trying to get that back for me. But you see, that's one thing I know has happened since... before the lockdown. I was I felt as if I was on track to get an a bit back of who I was, I was doing a rehab, I put on about five or six pounds, weight. I know that sounds nothing but to me that was a massive deal. Massive deal. With is interacting with people in the gym I have something in common. I knew I was good at physical activities, that lifted us up and that and erm those pushed me they knew I like being pushed, so I had a challenge. And me handful of friends was those were good. And then all that, all that went away. And I couldn't, with the leg that was you know, I'm still having a problem with me with the foot when I'm walking.... erm... And I sort of, I've went backwards. If you know what I mean? I went backwards. I'm glad there's no people out and about everything. So I've started what I've walked three miles this morning. You know, I went out at seven o'clock. On the way I live I live in paradise at Bolden and I walk 62 steps and go through a cut. And I've got a half a mile of burn and the right of me in a half mile of burn to the left of me and I do a complete loop and it's 1.1 miles. And I've done three loops of that this morning. You know, nobody nobody at all. So I'm trying again for the to get back what the pandemic too away. If that makes does that make sense to you? You know

INT1 **Yeah, total sense. definitely get that erm**

RES What I've done. I'm sorry for butting in I've went from down there to up here. And I'm back down there again where I was it the beginning. I'm trying for the to go there again. Because there's nobody, there's nobody out there. But it's it's it's a thing. It's all about that. But with a brain injury REDACTED and Sam, you cannot plan. You can't, you really cannot me wife plans for me. And I go with with the flow. And this is this is an interesting experience. Because I said to myself, my wife went away on Thursday and what I was going to do I was going to do with this and I was going to do that and I've done jack all, motivation I need to have somebody kicking me arse saying do this do that and it's been an interesting few days. If Anne is here and she saying go and do this I'll do it. But I don't seem to do the plan in my head. But I'm aware it, I know it is. She deliberately didn't write me a list little bugger, she deliberately. I know what's, with the this psychologist kid that comes around the two of them work together against me they do, just trying to make light of it. The erm.... but I know what they're doing. what they're doing (laughs) and I'll be living up to their expectation expectations

INT1 (laughing) good, good, keep going. Erm. So throughout lockdown obviously if you've had your wife with you at home I'm guessing for the most of the time were you still in contact with Headway via some sort of technology like this or had all of them and things stopped as well?

RES I can't, I can't do that. You know I was telling you I'm not one of them people, if you've got eight of them on this, and they're all shouting at the same time I'm sorry. And that's no reflection on them. It's me. Those can cope with it great. I cannot, I cannot I tried it

INT1 it's hard it's no

RES It's very hard because all shout at the same time.

INT1 Yeah, I don't like it i that much prefer to be in a room with people face to face.

RES Yeah.

INT1 So Have you erm been in contact with anybody else sort of have you had anybody visiting you through lockdown or did you bubble up with anybody or..

RES No we just came the culmination of my claim that's took two and a half nearly three years. I didn't, that was another thing that they've done it Headway if it hadn't been for Headway I would never have made my claim. They knew I knew who REDACTED Brown was. He's the chairman of Headway is the chairman of a half a dozen other brain injury or, he's brilliant

doesn't just do it for financial gain. He does it with a passion The man is, you know every so often you meet certain people that you know are just really nice, caring he's one of them, he's kept on putting, you know, solicitors kind of approach it kept on putting this guy next to his when we had do's and fundraisers you know, and erm massive you know, speaking to him and that he would never discuss me case, oh what did you used to do I'd say I was in the building game. And then after two or three obviously Anne realised I was bad and I have to make I didn't have to make a claim. But the way the ex boss's went on was getting up me wifes nose. So she spoke to the solicitor, solicitor company, came to me house done half a dozen test's, because solicitors there's a lot of phoney's make claims and and me solicitor knew because I've never approached him I wasn't a phoney, they know the things to look for, come to me house ripped out the, (unclear 39.12) all cognitive test, there was a giraffe elephant and a rhino and he says what's that? And I told him very eloquently that there was a rhinoceros pig. He burst out laughing. And me and my wife went next door for half an hour and then we started the claim. So two and a bit years of being taken care of that. Culminating in the the just settled in the April wasn't happy about so all my time and my wife's time have being preoccupied, no not mine you see, I talk rubbish. My wife, not me at all, my wife's times been all occupied with that. Up until April, we had a court date on the 22nd of April, two weeks before that pulled out money to settle outside of court which made me very, very angry because the lies that were told to psychologists lied through his back teeth. Questioned, (sigh) I have four years City and Guilds, I have an ONC, an HMD and an IOSH part one, part two, a NEBOSH. I could, I've got I've got something like 10 trade qualifications that have taken over approximately forty years. He said I doubt if Mr. Egan has one qualification. That hurts you know, things like that hurt. By law, I cannot be a site manager, unless I've got I've got NVQs, two, three and four was as well. That really, I ended up with tears over that and I wanted him in court, I wanted him in court. Anyhow two weeks before we due to call, to go to court REDACTED phones up, I could hear him speaking to an Anne, and Anne's going that will really upset REDACTED that really. Anyhow, they paid the price fight, the judge seen through them, that cost them, that cost them another 130,000 pounds. Thank you judge. Erm, But still, I want, I wanted my day. So all my time, everything. I've been on ice and now got a fantastic settlement. Right, over the moon had to give up 25% liability, which hurt me that was because I had a NEBOSH qualification. And they ended up blaming me for all the stuff being put there. Because I was in charge of health and safety. I wasn't there when they put it there, as soon as I seen it was there I wanted it move (ahhh), anyhow, you know, we can completely with you. So yeah, so um, I'm now at a stage where a lot of money. Ask us if I'm happy.

INT1 Are you happy REDACTED

RES Not one little bit. Not one little bit. No, my dear. psychologist, she said I said five years in the wilderness. And erm you know that saying money doesn't make you happy? I never Ever. Ever thought that was true? It's true. Yeah. So we'll, we'll see what happens.

INT1 So yeah, definitely. Money definitely isn't the be all and end all.

RES No, no, no Far from it. We, I've worked all my life. My wife was it was a senior civil servants, senior civil servant 35 years in the ministry. My last 10 years wages. Yeah. I averaged the net of 48,000 pounds a year. You know, we're not poor people, I worked hard. I worked all over, sometimes. I would go away and not come home for six weeks. And, you know erm, we built bars. The company Scrafton and started in 1886 we the built the first pub for Vox breweries, you know, that went bust, well I wasn't well, went in the voluntary liquidation because I was the driving force there, the managing director was a waste of space but thats (unclear 43.53) again time for another week.

INT1 If we can just talk about sort of, of lockdown if that's all right em you mentioned sort of about how much you enjoy exercise and fitness and things and you're walking in now. Did you try and keep up some fitness through throughout the lockdowns? Or is that just something that you've started to do again recently?

RES I just got a phone call yesterday from my local community centre, which is only about 150 metres away that erm that they've opened the gym so I'm quite but I don't know how my foot's going to, It's it's getting looser and looser. I can't understand it. But then again, as I when I see Nigel Walker, the physiotherapist who's looking after me with my foot, he says howay REDACTED, your 68 man get a grip. See? I'm in denial about me age.

INT1 Aren't we all (laugh)

RES I'm going to see him next Wednesday. And then I'm hoping he's going to give me the thumbs up for Go, go. Go to the gym I'd be happy.

INT1 Yeah, brilliant. So what do you think you mentioned that you quite liked lockdown in some respects in the fact that there wasn't a lot of people about. You prefer that. But at the same time, you'd lost what you were building up and what you were starting to gain back for yourself. What do you think's been the hardest thing about lockdown for you?

RES .... I was in a set pattern. I was in a regime and it all went out the window overnight. I think that was the, I've got myself in to a place into a programme and I was dependent upon that programme three days a week. It was a set pattern, kind of suddenly literally whoosh gone in that happened at Christmas. Although I don't know how to how to express what you're seeing. It was only three it was Monday, Wednesday and Friday. And then, but then again, I'm talking at the tail end of the of the lockdown because that was a Christmas and I'd went the pandemic was how longs the pandemic at Christmas that would have been a year and a bit wouldn't it?

INT1 Err At Christmas it would have just been about 10 months we went into lockdown in the march so it would have been nine months at Christmas

INT2 and probably been in the country a year by then though

RES it would had, yeah, six months that neural pathways and then it was stopped. And I'd had like a It started in March didn't it of last year? Yeah, that would be right. Yeah. And I had a three months gap until I hooked up with neural pathways that came from Optimise at Crawcrook Yeah, yeah. Erm that we're not had nothing erm but I was just I was at the side of my house and I was just working out myself doing isometrics and sit-ups press ups, things like that. emm I just... if none of this pandemic or anything had ever happened I'd found a way of everybody has a way of coping with something, or two people can have the same problems two people will probably cope with it in completely different ways. You know. I had found a way of evolved avoiding avoiding the public. I didn't. My wife and our friend went to America for a month and I had, I had one of our good friends Julie will come round for me at nine o'clock no half past nine in the morning when the schools are all finished. Now I'm going back to pure, pure after my accident when I started getting out and about which was about six months and my wife was comfortable and her and Bet went to where did the blues come from in America and Georgia the dell you know the make on Georgia and all that. Her and Bet went over, I went over they'd talked about it for years, I'd already been at work in America I've done all that. Seen and erm Anne and Bet away for five weeks. See how it's starting to come together and Julie would come for me at half past nine with and I had my dog was still alive spike and her dog and we would walk for an hour and a half remember then she'd go and do her stuff I would do my stuff lie on the couch. And then she'd come around again at lunchtime and I would cook because Julie was me. My wife's got a very conservative taste. now when I worked for myself in the 80s 90s I done nearly every Asian restaurant in South Shields refurbished them, yeah. And Julie, Anne wouldn't come but Julie would come with me on the opening nights and you know get all the food and that and but whenever any forget If I had worked on them or not if a new restaurant had opened or anything like that, Julie, me and Julie would go because we're both foodies, so I'd cook nice little dishes and stuff like that. And me and Julie would eat great so Anne comes back and still me and Julie would go and do whatever or if it was English, this restaurant Carter and Miller when those first start opening up here three years, four years ago, me, Anne and Julie would go. So I've got erm I'm doing okay, Anne's back, me and Julie still going out and everything. Then me friend Danny, who's head of computing at Gateshead University when he found out he come round said do you fancy going surfing. Now I said Danny I cannot really, when he says I know what he said I've looked it all up, he says, but we can. You've still got your big (unclear 50.56) which are bigger surfboards. He says come on, he says we can belly board and we can this. So he started taking us out surfing. So but i'm keeping it again to no more than five people. There's REDACTED, this is REDACTED, this REDACTED, there's REDACTED and there is REDACTED friends that go back and I actually started feeling comfortable until when I was with them other people started coming, and I went right back into my shell again. Yeah, it's ... small is comfortable. That's when I was laying in bed last night thinking I must remember to tell yous this. I know you should. I know. You might be thinking why didn't you write down notes because I write down notes. And I only put abbreviations and I can't remember what the abbreviations are I get really upset and

chewed about that. Yeah, I've stopped doing, REDACTED my psychologist says if you can't remember them don’t wright, because I'm really bad. And I can't remember what I what I wrote. Yeah. I cannot do people REDACTED and I cannot do people Sam I cannot it..... Yeah,

INT1 **Yes, I think I think that's fine. I think that's totally understandable. You know what you can do and that's the important thing. You know what your limits are. And you just work within those. And from talking to you today, you've been, I mean, I'm in awe almost, of how much you've come from and how much you've done and how you mentioned about not being articulate. You really are. It's been an absolute pleasure to talk to you and to listen. Fantastic.**

RES Please don't praise me up.

INT1 **Oh, you need it sometimes though**

RES REDACTED seriously. Please don't praise me up, I'll go to pieces and I'll walk away

INT1 **Ah don't**

RES I'm just a bloke who's had a bang on his head and he's doing okay.

INT1 **You're doing fantastic. Erm I just wanted have you still been able to keep in touch with sort of Julie, for instance, and some of the other people that you've mentioned through lockdown. Have you been getting out and walking with them still?**

RES Yes. Yeah. I can't walk with dog walkers. Because I've lost me dog. Spike. little bugger. And then the other guy. There's too many people. Now it's ended up this six or seven you walk 100 yards we stop and talk about EastEnders for 10 minutes, then walk another 100 yards and stop and talk about Emmerdale I've got no time for anything like that. I now go out in the mornings by myself head down, bum up. And I'll do a lap. I'll do another lap, I walk two miles inside of what half an hour. I've got a little bit of a err me hearts going a little bit. nice, comfortable. I'll come back and have a glass of water. And we're now looking at about half seven. And I'm way off to ASDA I get my Danish pastries just coming out the oven. I come back home, make a cup of coffee. And then my wife will just get up round about then I'll sit and read the skin of my Guardian newspaper. She comes down she straight into the err, I make her a cup of tea, luke warm coloured tea, and then she read the paper. And if we're gonna do anything or go anywhere, I'll do it, inevitably not. Then I've got a bit of a growth, lump on my neck thing here. busy doing that. To have a scan last week I'm going for MRi. They're doing a go for an MRI tomorrow. Don't like the hospital. too big. Too many people.

INT1 Yeah. I get that

RES So she's back tomorrow as well.

INT1 Okay did you find your walk over your walks on a morning to find that the help you find beneficial?

RES I go to a different place.

INT1 Yeah,

RES Yeah that came to me from erm from REDACTED. I go out a this this is no exaggeration, right? We've got kingfishers on it. We've got Swift's out in the morning all the swift and swallows getting you know just above the river getting all the the grubs and everything that the flies. I go into a nice place it tries to balance my day. And, you know, get into a nice little place for my walk. Plus, it's a good little bit of cardiovascular.

INT1 Yeah,

RES Because I can't quite run yet. My foot still a bit of a problem but I can do a bit of a power walk and make sure that I'm it's it's working my lungs. You know, getting up a bit of steam. But I like it.

INT1 Yeah, it sounds wonderful. What are your thoughts and feelings towards lockdown ending and society opening back up again?

RES They're a little bit erm... open ended. It's erm we're bringing politics in. If It had been done properly right from the get go, we wouldn't be here, still under the conditions, right? If it had been done properly, with this delta variation. If If Boris Johnson hadn't been trying to be the big man in front of Modi, the Indian Prime Minister, you know, we wouldn't have this. It's because we're, I'm sorry. I don't know what your politics are. But we have an incompetent baboon he's a, well done Sam, you're alright. Both are actually smiling. And, you know, we've got an appso' we've got a man who's been promoted into a position of Prime Minister that powers to be in the background. Because there's nobody else is as ignorant, as thick skinned? Is that the fool? Who has no pride or anything? which is you know where I'm coming from and don't need to see anymore?

INT1 All right, well, we'll quickly move on before you're heart rate goes up (laughter)

INT2 someone else says it for once, It's therapeutic for me not being the one to say it for once.

INT1 So in terms of lockdown, and lifted then in sort of all those restrictions going to? And (frozen) looking fantastic for us. How? How are you feeling about that? Is that sort of bringing up anxiety again, for you? Or are you feeling quite like positive about getting your routine back?

RES It's a double edged sword? Yes, I do. I think I think about it erm thinking about this, the, the school holidays, and kids running around everywhere, and this and that, and everything, and so they should, and they have every right to do it. And I've got a terrible stinking attitude about it, et cetera, et cetera, and who I, how dare I, dare I even think they've got no right to do it. I'm ashamed of myself on that. That's Yeah. I told you earlier I was the most gregarious social person you could, you know, and I'm getting anxious about it. I am but I'm having a great bit massive wrap around on me house so I'll be kept away of it. And I know ASDA going to be busy, it's not going to be Bedlam at seven o'clock in the morning. And it's a funny one. totally is.

INT1 Yeah, I can totally understand that. It's, you're not alone there. I think a lot of people feel the same, you know, definitely.

RES Before, before my accident, I would never worry or get chewed about anything. It was always it's my attitude in life is being if you're worried or get chewed about something, you're already on a path to defeat. Because you're making things up inside of your head that will probably never arise. It's also a bit of a double edged sword because you're basically covering all angles as well. You know, overthink it but I prefer not to as somebody who's just, my saying on the sides were no desperate situations, there are only desperate men and that was one thing I always pride myself on. on having a cool head when everybody else is never going to just while we are waiting. But of course we're going to do it with we're going to fix it. It's going to come in, it's going to come in on time it's going to come in on budget. What's the problem? Come on there’s six of were here, we can sort this out. That's always been my not. Okay, I'm in charge of it. I'm the one who would get the sack or really get it in the neck, all of yous wouldn't but six heads are always better than one. It's not a able thing. It's, I will be the one who gets the pat on the back and get the extra five grand bonus at the end of the job. Right. But come on us, everybody you can do it. you can do it. So I've got to stop running away with myself. I'm aware I'm starting to run away with in anticipation, in anticipation of what, of life going ahead is normal. This is me brain injury. It's hard to keep it in. In, in, it's box. Yeah. It really is. Yes sorry go on

INT1 **No, I was just I've just noticed the time we've sort of been talking just over the hour, if you will, right to continue for a little longer. Would you like to take a break? Or?**

RES I'm starting to shake now.

INT1 **Okay.**

RES And we will go on for as long as this this is, I'm getting anxious now.

INT1 **That's alright**

RES Not not anxious with yous. Because you're making us think

INT1 **Sorry,**

RES no, no, no. Don’t because it happens whether it's you or anybody?

INT1 **Okay. Would you like to take a break? Because we can do this and finish it off sort of later on today or tomorrow or later on in the week?**

RES Then I have the preconceived anxiety of the pause and the wait

INT1 **Okay sorry. We haven't got too much longer there's probably a couple of questions, and then we’ll go onto the scales**

RES Can I just put me jacket on.

INT1 **Yeah, course you can. Yes.**

RES That's me. me endocrine system. me thermostat

INT1 **Yeah getting a bit cold**

INT2 ***It's been a really good listen, though.***

INT1 **It has hasn't it**

INT2 ***Extra questions to add on.***

INT1 **You do or you don't?**

INT2 ***I don't, I think it's been very thorough.***

INT1 **Yeah. I think he's covered everything without us asking.**

RES I've got my body walmer. Although it's really warm outside where the sun now is over there. It doesn't come around and come to the back of the house until about 12 o'clock.

INT1 **Okay.**

RES And then it's ridiculously hot.

INT1 **Get stifled then**

RES Yeah, I've got a big conservatory on the back of the house and when the heat hits that and it's like ahhh

INT1 **Right. Do you want to get yourself a coffee or anything**

RES Do you want a chewing gum?

INT1 **I'd love one (laughter). Changing, sort of moving away a little bit from sort of locked down. And could you define the term loneliness? So how would you define the term loneliness?**

RES Very happy with loneliness. It's a little bit deeper. This is a thing that we'll touch on with my psychologist. I come from an absolute total dysfunctional family. Right. And when I was 10 years old, in the morning, and it's Sunday morning, me him downstairs, I come downstairs and my father's trying to kill my mother. Knife in one hand hammer in the other. You can imagine the scenario. He was taken away given a 20 odd year prison sentence. Erm the whole family and everything went to pieces.... err I was an absolute lunatic. I got locked up when I was 13, best thing that ever happened to me. I was sent to Wellesley Nautical School at Blyth Northumberland. It was a naval based institution where the inmates ruled the place. On a Saturday morning, you are given half ounce of tobacco a Mars bar and a packet of Polo mints. On my first Saturday there two lads Ken Walker and Dave France's were the bullies of the dorm. I was in a dorm called Nelson. I was given a punch in the mouth in my mars bar and tobacco and Polo mints taken off me. I tried to fight back I was 13 year old, those two lads were 17 imagine how far I got. So then everybody told me Oh, that's what happens. After a few weeks, you become one of the lads and the only take the tobacco off you. i thought aye I'm not having that. So second day came and erm... I had a rock in my hand. So as much as I could I got both of them in the face with the rock. And then I got a kicking and they still got me stuff. So the third week they didn't even bother asked I just got a kicking and they took it off us. Well, I was so I'm not having that. I'm not gonna have that on a Saturday night or two fire extinguishers and I stowed both their heads in with them. I was whisked off to Newton Aycliffe remand Centre for people who are classed as crazy. And I was about to be charged for attempted murder until a phenomenal child psychologist called Dr. Agogi he came to meet me. What's this all about? Then he knew what had happened with me father and everything. But Furthermore, when I was born, erm I was err doing okay, and I went, my mother died who I thought was my mother. And we're at the funeral. And I'm introduced to my mam and my dad and a brother. It was me Auntie Lilian and Uncle George who brought us I was born out of wedlock. And in those days, I was dealt with, you can imagine blah, blah, blah. So that's where the damage was initially done. So going back to Newton Aycliffe this Dr Agogi he did all these tests and everything, and I was I was quite a clever kid. And he actually thought hay he stood up for yourself here. But you went a little bit over the top. And erm then so he took us under his wing. And basically, I was there I was in solid I'd just turned fourteen and they could it to us then I was kept it at Newton Aycliff, remand Centre for crazy kids, for lack of a better word. and I've got to take this is. And err... three months in solitary confinement….. And I learned to love it, then this is where you see, when you're a child. You can em, you can go wherever you want in your head. And you can learn to love and learn to hate. I learned to love where I was, because I wasn't going to hate it. And I learned to hate the system. Putting us there. Why what why? Why will okay I bashed their heads in. Right? Why did the system allow them to get away with it for so long, because it was easy control. That's what it was. very, very easy control. And furthermore, it was intimidation. It was bullying. And this is they tried to do instead of going what was this kid's problem? What was this kid's problem? You know, I got locked up. I got locked up for two offences of burglary 76 and consideration because me Mam had no money and she had five sons. And I used to break into shops and steal food and steal clothes and dud dud der and give dit her. I used to give them to my mother and she used to sell them and buy booze and cigarettes. So So this doctor Agogo takes us under his wing he , and he's quite happy with us there. And I meet all these other psychiatrists and psychologists and all these other specialists people and they're fascinated by me. Because I'm clever. And

I'm a guinea pig for three months in there. Nobody else. Ten rooms on a wing, and I'm just in it. So loneliness. Loneliness is a state of mind and you can make it whatever you want to be. And I quite, I quite enjoyed it. I really did. So after a while when I'm, all I'm saying is what you going to do about then people who bullied me? They couldn't believe somebody like me. So this doctor Agogi he started realising and when I was when I was there (unclear 1.10.07 Newton Aycliffe there'd been a massive riot at Blythe. Yeah, really? it kicked off about the bullying, you see people don't realise in those days, the powers to be, ex military ex naval, when there was trouble in the military in the armed institution in the in the 40s in the 50s and the 60s. Have you ever heard of the riot squad? Whenever the Army or the Navy or whenever they rebelled the riot squad was sent in with pick axe handles and they battered and they killed they killed their own people, right? to suppress it all, you might know what I'm talking about Sam if you've ever read anything about stuff like that. So they done The same at Blythe only these young lads from 13 to 16, who are being bullied on a daily basis, you know, people get people get fed up with it. You know, it happens in all young institutions, but they don't talk about it. I've got a friend REDACTED Totten from South Shields who was at Medomsly. And he was a nutter, and he wouldn't get the young kids for the nonces that were running the place to be raped. Right? So the smashed his shoulder blades, to pieces, he's still fighting them in court. Because he wouldn't be pick young kids for the nonces that were in. You must have heard about it still going on. So anyhow. So yeah, I thought they told us after three months, I was going back to Wellesley, I went back to Wellesley they kept this for another month in an isolation cell of loneliness. So I like loneliness. Take us away from my family. And it kills me though. I'm happy being lonely with me family.

INT1 **Okay. And is that something that you've experienced later on in life? Or, particularly recently through the lockdown? Would you have felt lonely in either a positive way or a good way?**

RES I felt lonely, but it's it's not how you would feel with loneliness. It's, you understand what mindfulness is? Obviously you do, right? It's the same. I explained to REDACTED, my psychologist, and I can go wherever I want in my head, REDACTED, anywhere. You're only restrained by your thoughts. That's the only barrier. But you have to be on. Have a certain awareness of yourself before you can get to that stage.

INT1 **Yeah, definitely. And do you find that erm.... you have felt that especially recently, could you purposefully bring on two minds or do some mindfulness? Or is it just something that you've learned to just do naturally without really thinking or realising you're doing it?**

RES It's inherent. It's It's my auto go to. But that's got nothing to do with my accident. That's my, that's been my failsafe since my childhood like I say its inherent.

INT1 I can totally see why that would be the case. Yeah, definitely. And, Sam, do you have any other questions? I think I've covered everything off the interview.

INT2 I don't particularly I think, just for our demographics stuff. Do you know when you had your, I don't know might have actually covered it if I've missed it. When you had your first. Well, when you had your accident that caused your brain injury, did you have any loss of consciousness?

RES Oh, yeah.

INT2 How long was that for? If you don't mind

RES Now. I think it was, I was totally gone for, for, I'll tell you.

INT2 I somehow found a bit strange when we ask that as like, well the only way you could know really is if someone's told you.

RES Ah I've got that here because my son took a photograph.... erm So I can tell you exactly (mumbling 1.15.12) Yeah here we go.... ten, ten to seven in the accident happened at three, four hours I don't know if you can see? Can you see that?

INT1 Now you haven't got your camera on REDACTED. We can't see, can't see anything we can just hear yeah,

RES We, I haven't got the camera on?

INT1 No, (laugh)

RES how..

INT1 Err you can put it on if you click on your, are you on computer or a phone or

RES I'm on the iPad to access the camera Okay, yep. Can you see me now?

INT2 Oh yeah

INT1 yeah Hello? Yes

RES Can you see that...

INT1 I can just see your face so I'm not sure where the camera is might be a bit higher....

INT2 It's up now I think

INT1 your screen off....oh yes oh goodness me. Wow right yeah That must have been scary for your wife.

RES That was I was the big lad

INT1 Yeah,

RES Now just let them pathetic skinny yeugh. it was didn't know what or why or where Iwas or anything Can't believe I've been speaking to you guys with that. With that off. You should have said something

INT1 Lots of people do and we're happy either way. You don't have to have your camera on so you're absolutely fine. Some people choose not to

INT2 That's why we didn't say anything else because it's been a couple people who felt like they had to even if didn't really want

INT1 Yeah.

RES Where'd you, Sam where'd you come from?

INT2 Sunderland.

INT1 **Good to see you definitely got Sunderland accent. I could go the top of the hill. You know where the water reservoir is between Baldwin Sunderland.**

Interviewer(s): **INT1**

INT2

Respondent(s): RES (P23)

INT1: Alright, yeah, so... could we start just by telling us a little bit about your brain injury?

RES: I've had a few. My first one- erm... my first two is after a seizure. So I had one seizure, how had a [inaudible], hit my head on a desk, swivelled in a chair and hit my head on the floor. Then two weeks later, I had another seizure, hit- kept banging my head off the floor. A year later I hit my head with a blender. A few months later, my little boy knocked into during hide and seek and just- hit me on the head, ended up in hospital again. I walked into a door. Erm, yes, I know [laughs] I'm very clumsy.

INT1: We all have our moments.

RES: And then my latest one in February, erm I- was picked up my little boy's scooter. And I hit myself- as I picked it up, hit my head with that and then flew back into the garage. The garage caught my fall, so I hit my head there and then hit my head on the back.

INT2: How long ago was the first one roughly, do you know?

RES: Four year ago.

INT1: That's a lot to have happened in four years... wow.

RES: I hit my head pretty much every year [laughs].

INT1: Do you have them scheduled in now?

RES: And I'm under [inaudible], which is the brain injury specialists. And they... so after I hit my head last year, I think that was with the door. They said that I never really got back to where I should have been. And then I hit my head again, and I'm just on the bottom. Like I've got no concentration levels, I'm struggling to read, I'm really tired, I've got chronic fatigue. And like when I read I'm- it's really blurry, and especially at night time so even reading text messages it's really really blurry. Erm, it's fine in the morning, so I tend to do everything in the morning. But by night time that's why this said it's chronic fatigue because by time as you go through the day, struggling walking. So if I pick up my little boy, I have to do it on the afternoon because I can't physically- I'm struggling in the morning getting up. So I can't do the mornings, I have to do the afternoons and I have to take it really slow. And it's just

building everything up. So they've said, I have to read a chapter of a book that I've read over and over again. So it's Harry Potter at the minute [laughs]. And I read a chapter a day. And I slowly build that up to two chapters, my walking I've got a build up. So I do like 10 minutes a day, on an afternoon, I and I, like, on a lunchtime and then build it up to 20 minutes. The chronic fatigue, they have no idea how they're gonna try and do the chronic fatigue. It's just so bad. The- the- I'm a complicated case [laughs], as I said, because I've got epilepsy as well. And it's also causing absences as well. They said they don't know how, like, erm... because usually the two aren't connected. But they think it's because of the chronic fatigue, I'm getting tired, so the absences are coming on. Erm, so it's affecting both.

INT1: When you talk about the epilepsy, is that something that you had before the initial brain injury?

RES: Yeah, I've had that since I was 13.

INT1: Alright. So when you talk about like the different things that affect your, like, memory, and reading and fatigue and stuff, how much has that affected, like, your day-to-day life? Like those kinds of things that you would have done, like, on an average day?

RES: Oh, hugely, hugely. I mean, I didn't realise... I always thought I was just one of them forgetful clumsy people until I had a brain injury, and now I know what it is to be forgetful and clumsy [laughs]. I just can't- I find it difficult to retain information... I leave, and I can't remember if I've locked the door or not, and I have to go back and lock the door again. So I'll get halfway down the street, and I'm like, "Oh my God, have I locked the door?" And I have to go back. I'll forget to cook, so I'll cook and I'll let the chicken nuggets- like my little son's chicken nuggets, I cook them four times because I've burnt them four times [laughs]. That kind of thing, that's how bad the memory is, just retaining and little things, it's day-to-day things. That- that's- so I can't leave the kitchen if I cook like literally can't leave, set timers. I sometimes even forget to set the timer. So it's- it's just it's day-to-day stuff, just things that you would take for granted to do, boil the kettle, erm... it's just- it's just a struggle.

INT1: So would you say that the memory is like the biggest effect that it's had on you, it seems like that's the thing that you spoke about there?

RES: I think the chronic fatigue. I'm just tired all the time, like because of the brain injury and the concentration, I can't concentrate. Like just watching TV tires me out. It's like a watch TV, and I'm like, "why am I tired?" [Laugh] And the brain injury specialist they said because you're taking in so much information, just watching TV. And I never even thought of that. I thought that- that is relaxing time like switching off, and they're like "no because you're taking in sounds, taking in visuals, and then taking in the story." So I can't watch anything that really requires concentration. You know, like a really serious storyline because I just have a massive headache afterwards because I've had to concentrate for so long. So at the minute, I just watch programmes that I've watched a million times like Friends, Big Bang

Theory. Like if I miss something and I'm- I drift off, it doesn't matter, because I've watched it so many times and it doesn't hurt to watch. But if I have to watch or concentrate on anything, the headache is just har- it's so- so that's why he's trying to build me up.

INT1: When you so- when you say as well about how like, the erm, fatigue affects your concentration, do you think that it might actually, erm, might also have an effect on memory as well?

RES: Yeah, that's what he said he thinks it's all connected.

INT1: So is it a sk- kind of thing where like, if you could actually get around the chronic fatigue, it might actually make everything else a little bit better?

RES: Yeah, yeah, that's what he said. But he- he thinks that the chronic fatigue is gonna last for years. And he has no idea how it got on top of the chronic fatigue. Just, the only thing that he can help me with- help with other things like reading books, because I'm concerned about going back to uni, like I have no idea how I've passed so far when I realised and he told me that I didn't even hit the level where I should have been before and I was studying at uni. And I had no idea that I should- I shouldn't even have been able to pass because it was that bad. I just couldn't understand why I was struggling at uni and I was putting pressure on myself. Going "why am I- at- my brain injury was a year ago, why am I not able to concentrate on these books? Why am I struggling to critical think? Why am I"- how I passed I don't know [laughs]-

INT1: It's an incredible effort like.

RES: injury specialist said, like, a psychology Master's is not something he would have chose for me, like, it's something I never should have went into [laughs]. So how I passe-

INT1: I'm not sure anyone should with how this year's gone [both laugh].

RES: And I'm really- he sai- I'm really dreading going back because I had to leave and put it over three years. And even before I hit my head again, we're doing neuro psychology and I couldn't even watch the three minute videos, I was falling asleep. I was putting to a minute, falling asleep, and that was before I hit my head again. And I physically couldn't concentrate on the videos, it was it was too much learning about the neurons and it was so in depth. And I said, if we go back next year, and I have to sit through a two hour lecture, I'll not get through it, like there's no way I'll be able to take it in, like the headaches and everything. And that was before I hit my head again. And he's like, the brain injury specialist is not confident that I can go back in January, he said at maximum, I'll be able to be at the level I was before I hit my head again. So to- to go back- and I'm not even sure I'll be ever... go back

to where I was before the first brain injury. It's I'm constantly in brain fog. And I was like "is that my tablets- my epilepsy tablets?" as well. So he's looking at reducing maybe my epilepsy tablets and maybe risking more seizures to try and see if they can compensate and lessen brain fog. Just to try and get my day-to-day life back. Really.

INT1: You know, oh I've forgot what I was going to say about erm- oh yeah, you know how you've had like these multiple injuries as well, do you feel that, like each next injury is like, kind of making uni and stuff like that harder to get on with?

RES: Yeah, definitely. I feel like after each one... I had two others and I was neglected afterwards, it was just kind of like, "oh, you're fine. You know, get on with it." And I didn't feel right after the one last year. Like the chronic fatigue was quite bad, but it was just kind of like, "Oh, you've hit your head again", you know, what do they do? And I was like, "I really don't feel right, I'm not able to read, I'm not able to concentrate." It wasn't until I'd hit my head again that they realised I wasn't actually right after the last one that I wasn't actually sorted and they should have checked up on me. And that- that, erm, the chronic fatigue hadn't went, I was having problems concentrating, and now I've went [makes noise and downward gesture] way down. And maybe if the had looked at me last year, I could have maybe improved, but I do feel- like my clumsiness has gotten worse, like after each time. My husband says it's not normal to constantly keep hitting your head [laughs]. I said "I've never hit my head before, my head was always fine. And then after the brain injury- I just got the first brain injury... Four year ago, I was just really clumsy." I'm just constantly clumsy. I was always a little bit clumsy, you know, but now I'm really clumsy [laughs].

INT1: It must be frustrating as well to be, like, thinking... for if it might feel like things are becoming easier to get worse. If in that sense if things are getting worse each time you hit your head or something like that. When you- when you talk about, like, can't remember if you said like a therapist or something, for getting helped. What has your rehab basically been like throughout this process and has it changed?

RES: Erm, I see any neuropsychologist, I see an occupational therapist, I see a brain injury specialist nurse. Erm... I see a psychologist, erm, just a normal, you know, psychologist, therapist that they talk to and assess- they've got a really big department actually. Erm, it's [names hospital and location], the occupational therapist comes out once every couple of weeks to see me... erm, just to- just to give me a plan really and check up how I'm doing and I have to write a daily record of everything I've done that day and to see how my concentration levels are and to say if I've done too much, so he says if I've done something one day and I've- I've done too much that I have to do nothing the next day. I have to pace myself, which I'm not used to doing because four years ago I was really active. Like [laughs], I didn't pace myself at all I filled up my days and I was working, can't work anymore. I'm on disability. I went from not being on disability to working, to not working and being on disability. So that's a big change. The team are really good erm, what, like the neuropsychology do- deal with the medical side, and occupational therapist deals with my everyday day-to-day side and managing.

INT1: What- what do you think has helped you the most or been the most difficult about the process?

RES: I think the occupational therapist helps the most because he helps the day-to-day side, then the initial- after I've hurt my head the neuropsychologists are the best because they say "right what part have I hurt?" [Laughs]. They go "right what- what has being hurt?" And the last time they saw me they were just like "you've done everything" [laughs]. They were like "your cognitive just everything, you know?" [Laughs] They were just like "your cognitive everything you know" they said "you've done your front, you've done your back", I forgot what they said to be honest, so, just like mainly I've hurt- I've done- the neuropsychologist said the cognitive and said something else I've done as well, that I've knocked and said it'll take a while to to get back to... to improvement.

INT1: I suppose if they're saying it could take a while that's not ruling out it happening at all though, so-

RES: They don't like to put time limits on it.

INT1: Yeah.

RES: And they like to- they don't like to say... because they don't like to use the word 'normal' or anything like that because they say nobody's normal and they say there's no definition of normal. They just say whatever happens happens and whatever improvement happens and... but they said they were the ones who said you can't do uni, like, put it into three years. Erm... an occupational therapist is the one who's saying "we'll be able to get you reading by January, you know, your psychology books. But we can't put a time limit on anything else and we can't put a time limit on what you'll be if you'll ever be back to completely the way you were beforehand, or if we're just going to get you the level you were after your last brain injury or the brain injury beforehand". They just says "we don't know", we just can't put any- when it comes to the brain they just said "we don't know we can't put anything on it."

INT1: Because then I suppose another thing for them is like if they put a date on it and like, you don't have another injury in that time, and then hit that date and you're not where they said you'd be that probably puts them in trouble as well and might make you feel bad and stuff. When you talk as well about the, like, clips as well for uni that get uploaded and you talk about lectures and things like that. I'm assuming that- well I don't know why I'm saying assuming I know that they've came in because of lockdown. How do you feel like lockdown has been for you and what would you say has helped you?

RES: Struggled, I struggled all the way through lockdown because my brain injury- everything. I go to Headway, so, Headway was great, I got out like once, they to do it once every two weeks. And one week was physical one week was craft. It was starting up cooking as well so I was going to do a cooking class. I also went to [location] Mind as well and I went to their lunch club and that got me out, so anytime I felt really down because I get really bad anxiety with it, I have mood swings, like how my husband deals with I don't know because each day is a different mood [laughs]. And obviously because my chronic fatigue, like when you feel tired you're not in the nicest mood. Like when you feel constantly tired, it's like having a bad night's sleep. So when- like groggy when you go up in the morning and you're groggy, so you're like you're not in the nicest mood, like, so going to the lunch clubs going to Headway, it really improved my mood and I came out feeling a lot better having other people to talk to in their similar situation. Lockdown I was- I was locked in with my chronic fatigue, moody with a five-year-old, he was five at the time, and I was having to teach him, doing uni I was not in the best place [laughs]. And doing the lectures at uni I couldn't do it online I was really- when I was in uni I passed, I did really well because I was able to talk to the lectures I was able to take more of the lectures in. I was able to concentrate more, but it as soon as it went online, my concentration levels- that it was tiring, I was having really focus on a- on a screen. I wasn't really on- able to understand it as much, I was forgetting it. And I know when I spoke- the teacher said, "well, you can replay it" and I was like "but it's a lot of concentration, sit and watch it, and then replay it", especially, I'm not going to name names on teachers [laughs]-

INT1: It'll get anonymised anyway.

RES: -but if its a droning, especially if it's a droning voice. And you're like, sleeping if it's constantly droning, and it's monotone voice. And it's not an exciting lecture as it is if it's like on SPSS [laughs], and you're having to really concentrate. I was just falling asleep during the lectures on the phone and I was having to rewind and go back and it just wasn't- it wasn't going in. And that was before I hit my head again.

INT1: Did you feel like, if it was in person but longer like because you said about going back to having them like an hour or so? Did you find them easier to concentrate in person... was that before the last injury has that made it worse, do you think for concentrating going back or was it...?

RES: I still think- I don't know, because I've hit my head again, I'll not know until I go back. But before I hit my head again, this time, it was definitely better in lectures. I mean, by the time the two hours came around, I was really tired, I was needing a break I was needing a rest... but it was just an hour when we first came out of lockdown and it was just an hour in class and that hour was really good I felt like I took it in, it wasn't too long, it wasn't two hours to the point where after the hour I was trying to get drained two hours was too long for me, I was starting to get really drained and I didn't take it in But the hour, where it was an hour a week I was really taking it in, I was able to concentrate we're on the computers at the same time so I was learning at the same time and it was perfect for me. The two hours was slightly

too long I was starting to drift off in the class, I was getting really really tired... I was using coffee to try and keep me up and [laughs] they say to brain injury don't have coffee because it's a stimulant, it makes your brain injury worse, if you do don't have coffee. I was needing coffee in the break time to try and get me through the lecture... but the hour after lockdown when it was an hour a week in class that was perfect for me. I was spot on it wasn't too long to get tired but was enough where it was communication to take it in.

INT1: **Something I've actually just thought of which... I think that the actual plan for uni going into next year might be to ha- I think it's to have, [INT2], if I'm wron- if you know that I'm wrong correct me but I think the plan is actually from next year to keep lectures online but to have seminars on campus.**

RES: Right, that'd work for me.

INT1: **Would it?**

RES: Yeah.

INT1: **Oh well that- that- did you say would or wouldn't?**

RES: Probably would, yeah.

INT1: **Oh well that- that's good then. How do you feel about that- obviously the prospect of that for next year and then also just like getting out of lockdown because obviously we were actually supposed to be out of it by now weren't we? But it's been- even though it's loose, it's been extended. How do you feel about the prospect of normality? If we can ever have that again.**

RES: I'm fine cause headway are constantly in touch they've started up their groups again, [location] Mind have said in July their groups their lunch clubs are starting up again, so it's giving me something to look forward to, I'm not homeschooling. That was the worst. And cause his stuff is actually- some it was complicated for a five-year-old [all three laugh].

INT2: ***Yeah, I definitely spent more time googling what I should be teaching than actually teaching anything I don't know whether you were the same because I didn't get it either [laughs].***

RES: I had to message the teachers feeling really stupid, asking what- what were they talking about because I don't understand. And that required concentration, I had no time to look

after myself, so I spent all my time trying to concentrate and using all my concentration levels on my child. I had no time to do anything else or trying to do uni and having concentration left over for myself, I didn't have any. And I just- I did one of my assignments in one day. I'm surprised I got a 48[%] because I literally did it in one day. Because I just had no concentration looking after my son. And my husband took him for one day on a weekend I just said right I'm just gonna do my assignment in one day. So I'll just do it one day while I've got my concentration I'd slept well the night before. Do it in one day, and just handed in.

INT1: Did it feel like an accomplishment to be able to get that done in a day though?

RES: No, I felt terrible because I did it in one day I went "there's no way I'm going to pass" [laughs].

INT1: At least you did. You know the thing about school as well, like, it might vary from school to school, but I found that like- th- especially early on, the stuff that the schools were giving to the kids just wasn't helping at all, like, my girlfriend's niece had some physics homework to do- well it was all homework, wasn't it. And there was something to do with waves, and she'd- apparently she'd been looking at it for about two hours and was just crying because he didn't know what it was. And so- because I did well in physics- like in it and like GCSE, she asked me about it and showed me the actual like, materials that they'd got given and I was like, for a year nine to be like, looking at like the terminology that they use on this thing is ridiculous. And literally all I did was I drew a little diagram, and used the actual basic terms and send a picture back and she was like, "Oh, yeah, I get it now" [RES laughs]. The- I don't think the schools help the kid- obviously it was hard for the teachers and they've tried their best I don't think they helped at all some points... that was a real irrelevant ramble from me though [laughs]. If you could forgive it, when you- I've kind of jumped to the end of lockdown, but there is more talk about lockdown who- who have you apart from like your husband and your son, who have you- have you seen anyone else throughout the course of the pandemic and anyone else to speak to help you through?

RES: My in-laws came over to the door to sort of see my little boy and give him presents, but no one else. No one else in lockdown. Towards the end of lockdown, you know, I did social distancing with my friends, which really helped. But other than that, no, I was in a room with my son, and that was- that was it.

INT2: Did you utilise any sort of technology like- like we're doing now to talk- to keep in contact with your friends or anything like that or were you using social media any more? Or again was that a no-no just because of the fatigue and concentration?

RES: I did have a group chat on Messenger and a group chat on WhatsApp. Erm... but that's it, that's- that's it really. But they were stressed to bits so I didn't really want to keep- they were in the same situation as me. All of them were to be honest. And it- it wasn't really the

same... I tried ringing my nana but she was just stressed all the time, she actually made me worse. So erm, I just tried not to ring her, I still try not to, cause she's really stressful. My husband was working from home so I didn't really have anyone to talk to, I didn't have my groups to go to or anything. So it was hard, it was hard. I do feel a lot more relaxed now, I'm not homeschooling, things are opening up again my groups are opening up again, I'm not at uni so I've got a time to relax and get back to... to resting and what I need to do.

INT1: When you say, erm, as well about how you- people from like Headway and things like that, obviously you'll have been seen people from uni as well. How much did you- when lockdown began did your typical day and the people you would see change beyond just the uni and was anything- there might- it might not be anything beyond that but... was there?

RES: I well- before lockdown I'd see my friends that weren't from uni but I have friends that live local and we'd hang out all the time with the kids. And I'd went from that to- to being quite sociable to nothing. And then not went from my son being quite sociable and seeing all his friends to him not seeing anyone and saying "why aren't I seeing my friends? when can I see my friends again?" So because he felt it I felt it as well it made that worse, my husband is not a social person, so he was fine [laughs]. He's an introvert so he was completely fine.

INT1: Well, at least- at least you benefited then, it must be hard like to be seeing your kid going through that as well like, and the effect that this could have on kids is like- I saw something about kids having like- it's like not gone from a stage of kids wanting to see their friends to, like, there being reports of loads of kids having panic attacks at the thought of seeing the friends. Which is... horrible like. What would you say that, erm, how do you feel about your daily activities at the minute? And how different do you feel about them now to how you felt before lockdown?

RES: Once I can get back out and do things again, erm, I'm fine. COVID doesn't bother me to be honest, I've been vaccine- I've got both now vaccines, I've booked like, three holidays [laughs]. I've booked [US location] for next year [laughs], I'm going to [UK location] in a few weeks like to me doesn't bother me, I'm not one of them people anxious about it. I was when it first happened, when it first came out was really, really anxious, like I didn't want my husband going to the shops, like if he went to the shops I'd have a panic attack, but that was like when it very first came out like last March. Like not March just gone [2021], March before that [2020], then I got over it. Once- once it's not as [inaudible], it's just like the new norm, it's something that you're going to have to have to deal with. So, to me I'm not bothered about COVID.

INT1; When you talk about, as well, the fear that you had initially, did your brain injury factor into that at all? Or was it just something that you were to feared regardless?

RES: I was having a lot of mood swings at the time, so I can't judge, due to a lot of mood swings, I do suffer from anxiety from it. So anything that I feel is heightened. So because of the brain injury, it affects my emotions, I don't have a logical side, so completely effects my logical thing, and so I only feel- so what I feel to me is true. So if I feel I'm in danger, then that is true, I can't look at the logical side of it. I can- I go by my feelings and my emotion. Which gets me in a lot of trouble because it's not until afterwards I see the logical side.

INT1: It's understandable like we all have moments like that as well anyway, even if it's varies how often from person to person. Getting a little bit away from lockdown, how would you define the term loneliness?

RES: Oh, that's a difficult one... Feeling isolated?

INT1: We're not looking like to be telling you the correct answer or not.

RES: I'm trying to think would I feel lonely? I don't know- feeling like, I've got nobody, I suppose that's when I feel lonely when I feel like I'm alone. Feeling like I've got nobody there.

INT1: When you talk about when you feel that, is that something you do feel?

RES: Yeah, like, I- I feel lonely. I felt lonely a few weeks ago, erm, when my friends were able- when my friends went out, and I wasn't able to and because I've had an operation as well on top of that. And my friends went out and I wasn't able to and I felt really lonely. I also felt really lonely when the peop- I was meant to be finishing my course right now... and I'm not in I pictured everyone graduating and I'm not, and I felt really lonely on that one. So they're the times I feel lonely I suppose feeling left out. Yeah, that's my loneliness being left out.

INT1: Do you feel like how your your thoughts and feelings and maybes experiences of loneliness have changed since the start of the pandemic? Or do you think it's not really been affected that much by it?

RES: I think the only difference is I'm more aware of when I'm in and when I'm out. If I never went out I didn't even really notice. But now because everything's been shut and now everything's suddenly been opened I'm aware of when I'm in the house and when I'm not going out and not- when I'm not socialising

INT1: Do you think about the awareness helps you all or doesn't really affect anything or maybes makes it worse?

RES: Only when I feel left out, I guess.

INT1: What- would you say it helps or-

RES: I'm like "oh well I'm stuck in the house" like I feel like I'm not more aware that I'm stuck in the house now I didn't before. But then on the other hand I've now learned how to keep myself occupied in the house.

INT1: So there's positives and negatives to it then. Would you- we've spoke a little bit about ho- your social life's changed over the last year, I think, do you- how do you feel about where your social life's at and then also maybes where it could go after lockdown?

RES: Erm... I don't really have a social life at the minute, it's non existent. And I feel positive-

INT1: You're not the only one [both laugh].

RES: -that after, I feel positive after lockdown things will go back to the way it was before lockdown, and I'll just go back to my groups and go back to seeing my friends and go back out for meals and go on my holidays and it will be as if lockdown never happened. Except when we have to wear masks and obey social distancing, but, other than that...

INT1: That's good then. I for one welcome the use of masks going forward. Do you- when you say about, erm, a little bit before about not feeling like you had anyone to talk to, do you think that that also, like, applies to loneliness as well? If you're feeling lonely, you would struggle to have someone to talk to? I think earlier you might've said that was more just during lockdown? Erm... yeah.

RES: I wanted to reach out to people, but I didn't feel like there was anyone there. I think that's contributed to because I'm- I like to reach out to people. But, then if I feel that I want to reach out and nobody's there that contributes to it.

INT1: Could things like Headway, and not being able to go there do you think that that might have been made harder? Like, would you talk to people about it there?

RES: I wouldn't talk to them about it there they were just more of a distraction. I just- I knew they were going to the same things, we don't really talk about it. We just sort of erm, we just distract each other with sports, and- but you don't have to talk about it because you already know they've going through it. So it's kind of like an unspoken thing where we're just- we're going through it, but we'll meet up and have fun. It's distracting.

INT1: And your use of the word distraction as well, like do you feel like it- like it's something that's always there, and actually it is literally a distraction and just makes you- it helps you not think about it, or do you think it actually does remove some of the negative- you nodded there?

RES: It's more of a distraction. It does help remove the negatives sometimes because if we have a meeting on Zoom she'll- the leader'll find things to help sor- sort of, we'll talk about disability bus passes or, erm... swimming like just things that it can help your day-to-day life, things that'll come up that someone's struggling with and she'll say, "right, if you go on this and apply for this, this'll help you." That- that'll that will help. But most of the time when you meet up, it's more of a distraction.

INT1: Alright, well, suppose the distraction is helpful and away. The- when you give us your definition of loneliness as well, do you think that people around you would understand what loneliness means to you?

RES: No, they've probably going through their own loneliness, I suppose.

INT1: Just more focused on like, their- what's their own things are and how they would process things like that?

RES: Yeah.

INT1: I think that that's everything from me through the schedule. Any anything just to like, follow up on, is anything you've got, [INT2]? Or anything you feel I might have missed?

INT2: Just a couple of things. First of all, can I just check, you've not mentioned it, but do you have a carer at all?

RES: My husband is.

INT2: Your husband, and is it just your husband and your son that you've lived with over the pandemic? You haven't been living with anybody else at all?

RES: No.

INT2: ***Just them, brilliant. And then I just wondered we talked sort of about the positives of your rehabilitation that you've gone through; was there anything particularly difficult that you've found with it?***

RES: I think it's been the chronic fatigue because it's really hard to help the rehabilitation. And it's hard to see when they say to pace yourself when you've got a five-year-old and they want to go out on a weekend, they want to go out on a Saturday and Sunday and they say don't, only pick one day or go out. That's really hard, or if something comes up on a weekday you can't help it, you've got to go out. And that's what he said, you know, you've still got live your life it is really hard. But then if a couple of things happen in a row, then I'm tired for the rest of the week, like I physically can't do anything. And it's- it's put me back a week [laughs]. And- and that- that's the, the thing about pacing myself is that actually, erm, is it something I'm actually able to do? That that's the bit that's hard. It's alright if I have a week where there's not much on, or I'm able to- when we come out of lockdown, and especially in the six week holidays, I can't pace myself in the six weeks holidays. I've basically said that to him. I'm physically unable to pace myself. The chronic fatigue is going to be terrible, erm, I've got a five-year-old who demands attention, he's an only child. So he wants "play with me, play with me, play with me, play with me." And then to- I find taking him out is better because he can keep himself entertained. But then just getting on a bus tires me out, it's that bad that walking to a bus and getting on a bus. So it's just the six weeks over holidays, anything like that the pacing is just not- he's going to have to find an alternative to help me with the chronic fatigue and the concentration and memory because it's just not- it's not going to work the pacing. So I don't know, that's going to help with my, erm, rehabilitation and things like that.

INT2: ***Okay. Do you find that... do you feel a little bit stuck sort of between like a- a rock and a hard place because you really want to do all those things with- with your son and you don't him want to miss out, but at the same time it's perhaps slightly detrimental to yourself-***

RES: Yeah.

INT2: ***-because you want to do those things with him.***

RES: Yeah, it is. It's really hard. It is really hard.

INT2: ***And sort of when we talked about, erm, while we were in lockdown, erm, and you were sort of stuck in- I feel your pain with the homeschooling I have two it was horrendous. Was there anything that helped you through that sort of kept you going or anything that you used or...?***

RES: I ate food, like I lived at the fridge, takeaways [laughs], like I stopped making tea on a night time and I ordered Dominos... it helped me through [still laughing]

INT2: Yeah anything for an easy life, definitely [laughs].

RES: If it hadn't been for takeaways I don't think I would've survived. Making teas on a night time as well, like honestly, takeaways saved my life I think I gained a bit weight but [laughs].

INT2: You also mentioned sort of about keeping yourself occupay- occupied in the house and finding ways to keep yourself occupied. Can you tell us a little bit about what- what you have been doing?

RES: Gardening, gardening has really helped. Focussing on projects on the house I redid- the third bedroom it's now an office, and gardening just completely redone the garden and planting, erm, it's really really worked like I spend hours out there and going to Aldi or going on Amazon and buying seeds [laughs] that's what I did I used to order from Amazon in lockdown ordering seeds and just planting an ordered soil and stuff and just spent time in the garden and I've got my little boy to help it's amazing how much time it took up. We cleaned a lot just reorganise the furniture [both laugh]. It's amazing how much you can actually do in the house, playing board games with my son like I bought loads of board games from eBay just- we've got a Pokémon board game because he loves Pokémon, dinosaur board games. Anything really, anything like that to keep us occupied- crafts did lots and lots of crafts.

INT2: With the gardening was that something that you'd done before lockdown?

RES: No, no, I- I neglected the garden, the garden never got touched and in lockdown I thought "that garden's a mess, I'll paint the fence, fence needs painting" and painted the fence. Erm, I cut the grass, I did flowers, did plants, bough- got garden ornaments oh it's a lovelier garden now we've took the garden swing set down we've got a fancy swing, I took like the canopy that I hated it that's all gone. Like it's lovely now [both laugh].

INT2: Brilliant. Is that something you think you'll you'll keep going? Do you think-

RES: Yeah

INT2: -that's like a new hobby for you now? That you're gonna keep on with.

RES: That is the positive of lockdown, I have done the garden.

INT2: ***Brilliant. And then sort of you mentioned when we're talking about loneliness and, erm, not having people around you to talk about or to share how you're feeling with, erm, when you mentioned sort of your friends starting to go back out again and obviously people graduating that you should have also been doing. Do you feel like- or would would you feel comfortable sharing with [audio cuts out] that? How you've been feeling?***

RES: Sorry? It froze a bit.

INT2: ***Oh did I breakup? I just thinking- when you were saying about loneliness and sort of feeling left out, erm, with your friends that have started going out again socialising in the university friends, do you feel that you could express how you feeling with them?***

RES: Yeah, I mean, I don't begrudge them or anything but [laugh] a little bit, I feel a bit envious, like-

INT2: ***Yeah, I can understand that.***

RES: Like, I'm going to be picturing them stand up on the podium getting their certificate, and I'll be in the house [laughs].

INT2: ***Yeah.***

RES: And then they'll be going afterwards and celebrating, and I'll be like, "that should have been me", and I've got another six months, I'll still have got my stuff to do, I'll still- they will have done their dissertation, it'll be over and done with, and they'll be looking for a job and career and be all excited and I'll be sitting in the house [laughs]. And I'll still have all that to do. I'll still have that dissertation to do. So I have the- the two assignments from neuropsychology to go through and all that class to go through and because I've already started and looked at it I know how hard it is, I know what I've got to do. So I'm dreading that already now.

INT2: ***Yeah.***

RES: And they'll have already done that so it's envious more on the fact they've already done it [laughs].

INT2: ***Yeah.***

RES: And I've got to go through it. So that's what I envy like, though- they would've already done it. It's over and done with they've gotten the certificate, and I've still got to go through it.

INT2: Yeah. And do y- do you feel like you could talk about how that makes you feel with the occupational therapist, or any of the psychologists that you see?

RES: Erm, it makes me appreciate them. Because if it wasn't for them, then I probably wouldn't even get to go back in January. So it makes me want to cling on to them, and see them all the time and soak up the knowledge they've got. Because if it's not for them, I'm- I'm not even going to go back in January, so then it's the other part of it. That should be grateful that I go- get to go back in January and finish it off and hope that I do get to go back. So on the other hand, I'm going to be envious, and then on the other hand, I'm going to be like, "Oh my god, I hope I get to go back. I hope I get to graduate."

INT2: Yeah, everything's all just up in the air, and you're just trying to keep juggling through it if you like. Yeah, I can understand that. Erm, the last question I've got, I just want to double check what your surname is?

RES: [Surname].

INT2: That's fine. Because on your- on your Teams, you've got [different surname]?

RES: Yeah [laughs].

INT2: That's fine. So just so I knew I've got the correct details. That's it for me, [INT1], thanks.

INT1: Well, while I was listening, I've actually come up with two more questions myself. So erm the first one is you know how you said about when you've been doing the house, how you've made an office? Do you find it that's helped with the concentration, being able to get things done, and there?

RES: Stressed me out so much, but it also kept me occupied. So it also gives me something- yeah, like you said, it helped with my concentration a little bit. Because I had something to focus on and like pick carpets, a bit paint. But I physically couldn't do it myself. I tried stripping the wallpaper off. Because I got sick of just waiting for my husband, because he was working. And I was just like, "right, I'm gonna go upstairs, I'm gonna strip the wallpaper off", I managed to do it for 10 minutes and then I passed out on the floor, because it was too much for me. And he was- I was like, "well, we can't do the full room. I've started on one wall. So we'll just stick with one wall, and we'll just paint it" [laughs]. And we've never touched the rest. We've just done one wall. Because that's all I could physically do, I think I would done

it, 10 minutes, broken up, went back, and it was too much my husband had to do the rest. Just couldn't do it it was too much for me. But it was enough where it kept me occupied in the house in lockdown. Enough where it gave me a hobby to do. But I physically couldn't do it myself, it was too much, I was passing out and just sweating. Too much concentrating, I was ill, I was having to go downstairs, take a break and eat and just let th- my husband do the rest.

INT1: This last question... kind of might have one or two little questions just to make sure that it actually- the main question actually works. The first one is, when- is your son your only child?

RES: Yeah.

INT1: Alright. Just because basically what I was thinking was that, erm... I might if- might- this if I've missed remembered the years that you've given this might not work at all, but, was there not much ta- time between your first brain injury and having your son?

RES: No, that wasn't I think he was like two.

INT1: So obviously becoming a parent and then a brain injury like this they're both two big quite changes in you- your life. Do you- how did you feel that, like, do you think that your experience of both of them being so close together might have been made easier or harder based on your brain injury and the demands of being a parent, erm, and like maybes differ from other brain injury survivors, it's just I'm, there's just something that sprung up I don't think we've had any of the participants who've had a brain injury and a first child so close together.

RES: Erm I don't I think [son]'s ever known me without a brain injury to be honest because I went back to work when he was two, and I lasted about three months before I went down and had the brain injury. So I was working with a child, erm... so not only was I had a two year old, I was working and then I had a brain injury and I had to leave everything. And, erm, so as well as- it was really difficult because I was having look after myself while going through a brain injury and my husband working, and I went through this strange phase where, during the brain injury I wouldn't let anyone else have him. Like I was used to my in-laws taking him once a week and I wouldn't even let them have him then. It was a really weird phase that I went through with the brain injury. I was used to hanging out with my in-laws all the time, I- I went through a phase where I wouldn't even be around them, like they stressed me out, I would get into arguments... I wouldn't let go of [son] at all, like he was two, and the thought of him going to nursery- he was due to go to nursery as well. And I just remember having panic attacks, oh I was in a mess with [son], like just looking after him. And my memory's gone to be honest, my memory is shot of what it was like, I just remember not leaving him, like, I would not let him go. It was like a bond issue that I had. And I- I kept feeling like if he left the house he would die? Like after the brain injury that's what I felt. So I wouldn't let

him go to anybody else. Even though I know logically, most accidents happen in the household. But I still have that now to a degree, like after the brain injury, I still have that to a degree I think if he goes out the house all the nightmare things that can happen even when he was with me, and he has to hold my hand all the time, like he's naturally used to doing that now because he's had that since of brain injury where I'm just controlling [laughs]. So he's just naturally used to holding my hand now, erm, he's naturally used to me groggy in the morning he's just used to the way I am now. He doesn't remember life any other way. Erm... yeah, he's used to me the more emotional one... don't remember a time with him where I have not been the way I am. I can't remember life with him before the brain injury, I just remember life before the brain injury because I went from being baby blues from having him, to still being emotional, getting over that, and then having the brain injury and going back to that. So to me never really had a break [laughs].

INT1: When- when you talk as well about how like it's not as bad now have you- is that something that you've actually worked on, and have focussed on?-

RES: Yeah, I had to work on it with psychologists, counsellors, erm, think I worked on it with a midwife er- not on midwife a health visitor because I was- he was still seeing a health visitor then. Had to work on it with her, erm, coming out and you know, trying to speak logically with me, you know, like, let him go [laughs]. It's fine [laughs]. Erm... yeah, it was quite bad. And eventually I think I just took the step and just let my in-laws have him, and that was my first step. Once he got out once a week I started going out again, I didn't go out at all, I didn't go out for about four months, I didn't leave the house after the brain injury I just stayed in the house didn't go anywhere, I didn't do anything. Because my brain fog was that bad I didn't make any sense, like when I was [inaudible] nothing made sense, like, in my head. God knows what [son] thought [laughs] even though he was two, like he was used to going out and he just didn't go anywhere. Yeah I just don't really remember it, that four months is just a blur. I don't remember any of it.

INT1: When you erm- you might not really have any feelings on this and we can move on to the scales if so but when he says well about how he doesn't really [inaudible] doesn't r- didn't really know you pre-brain injury, do you particularly have any feelings about that?

RES: I've just learnt to accept it, this is the way I am. He's used to- what I feel really bad on is he's used to me sort of letting them down in a way and lockdown letting him down, like I'll say "oh we're going here" and he's like, "yeah, if it happens." And I feel really bad, and that's a mixture of lockdown, and me not being well. So if I've not been, we've had to say "right mammy's not well, too tired we can't go" or- and lockdown's happened and everything's gotten cancelled. So now, if I plan anything, he's just like, "yeah, if it happens" and he's six, and he's went from being this optimistic child, who's dead excited for stuff to just saying, "yeah, if it happens" [laughs]. And I'm thinking "he's six he shouldn't be like that."

INT1: I think all kids kind of have phases like that, though. Don't know if you agree, [INT2] but I definitely did. Well, if- unless you've got anything else that has sprung up, [INT2], we can

go on to scales, no? Alright, I'll start sharing my screen, and this should not take too long. So if by any chance, you're sick of us by now [laughs] we- we haven't got long left.

Interviewer(s)	**INT1**
	INT2
Respondent	RES (P24)

INT1 **So if I could start and just ask if you wouldn't mind telling us a little bit about your brain injury.**

RES Hmm. I am. Well, so I err went to Cambridge University did a PhD at bath University. And then in my last year there had a bicycle accident in do you know Dolby forest down in err

INT1 **Dolby? Yeah, that rings a bell,**

RES yeah, I do a jump. And apparently, apparently my front wheel hit a rock and my face hit the floor. I was helicopter to hospital and in a coma. And that was about 12 years ago. I think. So erm... yeah, since then, I've lost erm I've got brain injury. I've lost half of my field of vision, my right half. So I really struggle with reading, which I've tried lots of jobs and even jobs I could do before but people just sacked me, because I've become useless. You know, I've done easy jobs. I mean, you know what hemianopia is? Have you heard of the term hemianopia?

INT1 **I do yes**

RES Right hemianopia. So we write we write from left to right. So reading anything normal is so hard. And I don't know, I don't feel disabled I feel fully functional, but i've had my driving licence. I mean, I drove for 11 years and my licence got revoked to say that and that's the that's the biggest thing. And I've been all over the country working and stuff. But I was get sacked. And I always end up coming back to this, which is a house owned by my mum for a long time. She used to let it out and stuff just gives it to me. So yeah,

INT1 **How erm... you've mentioned sort of about working things and obviously that your vision causing quite a lot of problems in terms of (frozen 01.49) how has the brain injury affected anything else in terms of your daily your daily activities prevented you from doing a lot of things? Would you say?**

RES Erm well, yeah, to a lot, to any level, like for yourself. If you want to relax I used to do all the time you read a book, you read something, but now it's now can't read even watching TV. it

shatters me. I mean, fortunately, we've got this three and a half year old now. So she takes my life you know, my daughter?

INT1 Yeah.

RES But It's just everything's changed. I don't notice I just noticed that people react to me differently because apparently a bit more outspoken and things and they used to be

INT1 okay,

RES so.

INT1 And so with the brain injury, was there anything else that was damaged? Other than sort of your, your vision, you mentioned about erm being more out spoken Has there been some sort of personality changes or anything like that?

RES not noticeable, but it's just a bit more extreme if you like, yes, that's what I picked up because people I was friends with before with before I kind of drifted away. I mean, okay, it's life, but there could have been a bit more supportive and even I went fishing with a real my best friend. He's go fishing with him all the time, but he just kind of drifted away. He lives very close, but just because I can't drive now. I'm useless to everybody basically, if people want a cup of tea, I can't go there. And it's just I do cycle everywhere. I shouldn't do because it's very dangerous, but I need to have a life you know, I mean, my my main hobby, I love fishing. fishing without a car busses don't go to fisheries or can't afford a taxi. It's just,

INT1 yeah,

RES I don't know, It's life though I just accept what I can do now basically, rather than hoping.

INT1 And have you have you had any rehabilitation at all?

RES Do you remember in Newcastle there was Momentum, they've gone now?

INT1 Yes.

RES Did you work there.

INT1 I didn't our researchers REDACTED did. He used to work there for quite some time.

RES Yes. I recognise his name. Yeah. Yeah. So I I was there twice and both times I left with a job and got sacked so they weren't much help, they gave me confidence you know. Met, again, with Headway. I see Headway Middlesboro when I can it's not on at the minute I know but I go there once a month as well. Normally, I was in headway Newcastle When I lived there, I did my Momentum things. It's just meeting like minded people. You know, people have the same frustrations you do. But again, it's not the same because every brain different a brain injury is different. It's very frustrating. For me, obviously with my education stuff is the reading and anything like that. So I came back to Guisborough because I was working in sixth form college here that my mum worked out so I had an easy way in, to help with maths. But then they got rid of me which is it's horrible really because A level maths isn't hard. But just I don't know what it is. They don't say to me, you can't do it, this. Make an excuse up saying Oh, the money's not we're not funding that anymore. Which for the amount of money they paid me is nothing for the help I gave. All the students like me, because I'm much more in tune with the students if you like, and maybe the staff don't like and not maybe I should be a bit of an upper level. I don't know. It's frustrating. I'd love a job I worked for triage in Middlesbrough where they help people getting back to work, but they came up with an excuse to get rid of me. So it's just frustrating, you know, I wanted to do something that helps people but what now without a car can't do anything, so

INT1 it's about, you mentioned earlier about what sort of focusing on what you can do instead of adapting things and changing things to ensure that that that that's your future plan sort of these picking out those things isn't it. And and somebody's given me a chance as well. You work with headway? Or you're sort of contact with them? Have you found that that's helped

RES Not Headways as such, meeting other like minded people has. Err in Headway it we just go there one a month. They try to arrange things with it's always just to go there to speak to people, you know, have a cup of tea and talk you know, so..

INT1 Yeah, see it more as a social sort of outlet then. Yeah,

RES I was, I lived in Hexham I didn't have a job there they sacked me. I was in Headway that I was in headway Newcastle. She's really really good in I lived in West Jesmond and that Headway over in it. is it what's it called up North somewhere and north of the Tyne. I love that house was really good to loads of people about 30 people every week. And then the headway here is once a month, it's not very common, but

INT1 what was it about the one in Newcastle that you liked so much?

RES The amount of people yeah, I'd say about 30 people met, maybe that's exaggerating, but the right 30 people you paid a pound, I think it was one pound a week or something just for cup of tea and biscuits. But you could talk to anybody and that's what I needed one he meet the same people every time which is good. Don't get me wrong. And there's a guy he has got the other hemianopia to me. So I can't see the right and he can't see the left. And it's just when you meet these people you realise why am I trying to work because no one, I mean I guess it's the wrong kind of people. They're not trying to work either because they no they can't and they're all the same frustrations, they get jobs. They get sacked no they get let go, not sacked because we don't say horrible thing. Just I don't know what it is, we're just not as useful.

INT1 And with your hemianopia have you had any rehabilitation to help with that at all?

RES Well, yeah, Well again, I'll say i've done research with you people who follows a girl in the University of Durham, Lina, I don't know if remember she's got a she's gone down south now but she was doing research into this kind of thing and she had this thing scrolling across the screen and doesn't really help much (laughs). Again, there was research behind it but you can't really rehabilitating it just have to learn to deal with it. So ...

INT1 yeah, that's it's just sort of re educating your your movements and and you're learning to adapt because of what you can see

RES Yeah for example coming to cross the road you don't just run across now and especially with my daughter she's the focus of everything don't take any risks with her you now, and the thing that's scary actually with them now on cars becoming electric that's quite scary because around at Guisborough its' quiet which is good, but somewhere like Newcastle's where there is cars everywhere, I wouldn't know that were there and when they break the law and go through a red light or something that's when maybe, I nearly got hit in Hexham, no in Jesmond in Jesmond someone went through a red light and I went across the road when with a Green Man and I was scared but again, he learned to live with it you check check, again. You do check two times, at least two times just to be certain. If you get hit by a car even without breaking the law, you're dead. It's a matter of law you're dead. So

INT1 it's not worth it is it? And and yeah, you're right with the electric cars. There's so quiet. It's a concern. And I suppose as well, I'm a similar age to you too. We were taught that stop Look, listen. that listen, it's irrelevant.

RES Exactly.

INT1 So If we talk about erm lockdown, can you tell me a little bit about sort of the people that you were in touch with during the lockdown sort of who you were you were seeing on a regular basis.

RES Erm I'm being honest in lockdown hasn't really affected my life because no one wants to socialise with me. So I have seen people on and off, various things have stopped. That I might like, I do try playing football once a week when people, so I go in goal where they can semi circle where only I can be, so no one else can come in my semi circle. Unfortunately, the goal behind me is a bit of a thing and jumps out at me. So if I go to the right but erm, that's coming back in now and everyone's a bit happier but I've never been a big pub goer or anything you know. So the fact they were closing didn't really upset me much well it makes everyone happier I don't see many people and I still go to Morrison's everyday to get food for the family. It's interesting cuz my girlfriend she's normal and she really erm she really struggled with it and she bought a car and thinks it's a new car thinking that would cure her, but she didn't use it, and she got very frustrated I think why are you frustrated? You've got a car,, you can do everything you want to do, if I the car here I used to love fishing on the coast like towards Widnes there so much fishing down, I used to love it over the winter, summer I'd go fishing anywhere but without the car can't do any of it and she can do anything she wants, which is still frustrated because she had no I don't know. She can't entertain herself. A lot of people are like that they can't entertain themselves or as I'm quite good I love again fishing quite happy with fishing by myself and just, it's a but miserable I know but (laughs). It's been good. So yeah,

INT1 that's it's whatever you enjoy. And so would you say wherever she's found it particularly frustrating. Do you think that's because she's had a big change from how her life was before the pandemic? And then everything pretty much stopped for her, whereas do you would you say that life is quite similar before the pandemic and during

RES I'd say Yeah, I would say exactly that I would say, she was just frustrated she was never very like a busybody she likes to do as much work as she needed to do to do to get by, but to have that taken away from and even when she had you know stable income because with my ESA goes up if she doesn't work, it's not much money but it's enough to live on and it's just as soon as she got the car again she's really happy she got a life back you know she can go anywhere and do anything and I don't know I'm just, like I say with my daughter she's been the focus of my lockdown and nurseries have been open, I don't know if you've been aware they've been open throughout the lockdown so she's been gone to nursery her life hasn't changed our life hasn't changed go to pick her up and cook for her and stuff. So it's been good and one interesting thing actually do you know what PIP is?

INT1 Erm... yes some sort of payment PIP

RES my Personal Independence Payment it's for disabled people

INT1 yeah,

RES I've self (unclear 12.22) because I before this all happened before lockdown I had a case out that I wasn't getting anything from them. And I'm like, the classic kind of person should it's just kind of educated people that don't like to give me it and thankfully, they finally looked at my case properly. And now I'm getting like what I should be getting in it's some kind of sort of my life I can live the life I want to live and don't struggle for money now. So it's what I needed and that against a lockdown. First thing with a lockdown look at is has this person got a brain injury? And it is yes. is a kind of a given. You know, there's a lot of people crazy, especially a lot of people will claim for you. And it's nothing wrong with them. Just because I wanted a bit more money. And that's that was that was one of the things I enjoyed as well about the lockdown, just like brain injury kind of. And the other thing is people without brain injury realise what it's like for us you know, they've realised you don't need to earn 50,000 or whatever, they can just live on pittance. And you know when erm Wales started doing this month where everyone gets what's it called Universal income? You heard about that? That's what it was like for us basically, wasn't it? So what an obligation to work and everyone else in the same you get his universal income. And they can work if they want to, people got very frustrated about it, didn't they? That's how we live you know, we disabled, that's how we live and I don't know

INT1 So like you said in the beginning that sort of lockdown hadn't really affected you. Do you think it's all of those sorts of things where you were already living this life? And it's just everybody else has joined you?

RES Yeah and mean so it Guisborough we haven't got a train stations so can't really get any where there is a bus but it's an hour and a half to the station. And even that's the Middleborough station which isn't very useful. It's very frustrating. I love trains. Buses are annoy me, because they're so slow. They always get stuck in the goddamn traffic. And so what if there's no buses we never I never got on the bus anyway, it's I don't know, my daughter again with a daughter. She didn't understand how life could be if I had a car. I would be taking her everywhere I know but, she's happy with what I give her you know we've got the woods justs behind us, like 10 minutes walk in the woods and she's happy to go to the park and happy you know and it does make you realise you don't need to spend a fortune. You don't need to do things when you're happy just seeing wildlife.

INT1 Yeah, definitely. put things in perspective a little bit, I think hasn't it in terms of what we do need

RES needs and needs and wants. Yeah,

INT1 **exactly. Erm sort of like you've mentioned, your daughter was still going to nursery And sort of going shopping and things that you were doing on a regular basis anyway? Do you think your daily activities erm have changed much because of the lockdown? Or were you pretty much doing exactly the same before and during?**

RES Well, there was a period when fishing was banned. It wasn't for very long which was a bit awkward, fortunately, I think it was early in the years kind of April time, which is never great time. I still went fishing because it's never that busy where I go. And the one thing that did well, I did say that I used to cycle a bit go with friends to kind of tea rooms and things and the tea rooms were closed. But in all honesty, I've stopped doing that. Because now my daughter's got a bit older she's much more hands on, I need to be there all the time for so I wasn't doing that anyway. I'm not doing it for a long time anyway. So my life hasn't changed much no.

INT1 **And how do you feel about sort of the amount of activities that you're doing in your sort of daily routine?**

RES I'm happy with it. Now. I'm just accepting it again. I always think life could be better. But again, since the injury I worked with the University of Cambridge as a teaching assistant there and being in the city. I don't know. I was going mad. Because you want to I love fishing want to be out in the country fishing when I'm a breaks but then in the City you can't really pop to the country it's not so easy whereas here. At least I've got the North North Yorkshire Moors just behind me and there's lakes everywhere. Yeah. So

INT1 **did you find sorry go on...**

RES No Carry on, carry on

INT1 **I was just going to ask about sort of the fishing and the nature. Do you find that that helps you find it improves your mood? And you said you really appreciate it.**

RES Because fish don't appreciate my hemianopia they don't care the fish you know, It's like y daughter she didn't understand my hemianopia, I don't, don't explain it to her. She just, she is my daughter kind err it's a level above just what you need you know. For example, fishing and fishing. I might not be able to see on the right and I might not see fish there. But so what it makes no difference in my live, I'll still catch fish and enjoy it. Yeah.

INT1 **Do you think it's the case for yourself that you're almost transported back to a time before the brain injury because the you nothing else is affected, You're just, just you and you're not reminded of of what's happened.**

RES That's exactly it. Yeah, that's exactly what upi say is completely true. That's completely true. I still people still come up and stop me doing it. I do break a lot of laws. I shouldn't be breaking but it's not a big thing. You know, if I'm using for example about fishing using a barbed hook, is there a barbless hook little thing, Tiny little things like that it's not a serious law? People get quite uptight about it (laughs) so I don'y know. Yeah, generally its fishing fishing.

INT1 **Yeah, yeah. Enjoy it?. erm Would you? Where you've mentioned sort of breaking the little rules with Have you always been sort of a bit of a risk taker like that? Or is that something that's come about from the brain injury, do you think**

RES that's brain injury has brain injuries because I do break a lot of rules. For example, if I'm going from A to B, and have to cross some person's land, I do because it's a straight line, I don't want to do pointless detours to get to the shop whatever. And people again people spot you doing it get quite quite uptight about it. These's rich landowners and things which I don't know. I never used to do anything like that, because but now I don't really care. It's my life. I want to make my life easier to go to the shops and stuff they've got I've got done car. But yeah, so I'll tell you now my frustration with cars is beyond anything I get so angry at them all the time, whenever they're breaking the law, Park on a pavement or whatever. If I stay on the road, it doesn't bother me when they're in my face It's.... frustration because I had a car for 11 years. And I know what it's like,

INT1 **I get that, I think car drivers can sometimes take them for granted a little bit. can't they?**

RES Excited

INT1 **Freedom that they give.**

RES Yes.

INT1 **So changing tact a little bit. And I'm quite interested, especially with you doing your fishing and yet I imagine you're quiet by yourself for quite a long time whilst you're fishing. And how would you define the term loneliness?**

RES Erm having no one to vent to I think, but fortunately, my mum is in Guisborough and I talk to her, have cups of tea with her every day. I mean, she's nearly 70 now but she's fully functional and and also with my daughter as well she's always there and always thing so you kind of don't worry about that. Let's say with loneliness I've can see my family and stuff because I think there was bubble the bubble thing wasn't there my mum's always been in a bubble so always been have a cup of tea with her. And like I said not really let it upset me too much again because again i don't have a car so if I want to have a cup of tea I cycled someone's house and have a cup of tea with them. Whereas with cars it's kind of obvious if you go to someone's house, you shouldn't be, whereas the bike it's hiding round the back or something? I think everyone else has followed the law a bit more than I have. But yeah, loneliness I don't know, hadn't been such a problem for me.

INT1 And would you say it's something that you have experienced in your life sort of that inability to vent to people and, and share your feelings?

RES No again, because my mum has always been there, she's always the backbone and she's the one who's driven through my PIP she knows to, because myself I don't realise whats wrong and myself, you know, she's the one I put down everything that is wrong. And there wasn't exaggeration and I don't know she's just helped me with everything she's like, I guess she's done my support worker you know or carer of you want so..

INT1 Yeah sounds like you've got a really good support system there with with your with your mum. Sounds like a good one

RES Yeah, with my daughter, with my with my, with her Mum, but like my girlfriend she's a bit less pushy than my mum You know,

INT1 we're all different though. That's it. That's what makes us wonderful. And do you think and your thoughts of what loneliness is have changed at all because of lockdown or potentially because of your brain injury?

RES Maybe because of the brain injury? Yeah. But has lockdown changed it? I don't know. Not really people say they've been lonely. But If you're really lonely you can go and see people. Oh, you know, it's not let's be honest, I don't respect this disease at all. Or this lurgy I probably should, I've not had me jabs but it's always been her you know, it's always been here, it's just now it's got above a certain level where it kills more people. I mean, every year the flu kills lots of people. Do they do anything about that? they don't really, just because it's on the news all the time think it's a lot worse than it really is. It doesn't kill young people my neighbours have had it. I know lots people had it and it's just there's lots of it it's not it's not bad you know. So..... I don't know

INT1 would you say your your stances that we've just got to get on with it now when you're quite positive about life or the lockdown or lifting and all the restrictions? How are you? How are you feeling about those sort of easing or going?

RES No difference really like, I mean I'm looking forward, I love train I love trains I can catch a train. It's just again with my daughter, she's stopped doing anything I can't (unclear 22.25) catching a trains an effort. I dropped off at nursery I pick her up for nursery, we only have 15 hours a week so I'm with here all the time. She's just kind of neatly potty trained. Now so it's awkward to go anywhere with her. Because I guess part of problem is you need to to where the public toilets are in the first place, which is awkward as well. But.. Yeah, I mean, yeah, it's only if if we're going somewhere that you realise this is this problem exists. I went to Edinburgh for my girlfriend to get her passport renewed. Because she's Lithuanian, and you realise nothing's open and the trains, there's not many trains running. And that's the only thing that we've done really? Just that one train ride and you realise. And buses again, normally just kind of halve - 20 minute buses to Middlesboro from Guisborough. But apparently they've been every hour. But again, I've not done that very often so .

INT1 And how have you felt about sort of the erm.. the amount of people that have been on the train? I presume it would have been a lot quieter than normal? That or is it not made any difference?

RES it's made no difference really, because the trains around here aren't very busy and know over in Newcastle though and Sunderland can be a bit busier, but when I go down to Middlesboro they're empty anyway. And yeah I know they've although they've been doing some cross country, they have trains twice as long. But and in fact, Northern rail, there's a few trains round here twice as long but you couldn't really rely on the trains because, I mean, for example, Google's had different times than the actual train lines you turn up in the train wasn't there and you'd be puzzled.

INT1 Yeah,

RES same with the buses. You couldn't trust Google to give the right answer when the bus was it's frustrating

INT1 I could imagine. What would you say if anything has helped you through the lockdown?

RES What has? My daughter definitely, definitely my daughter. Definitely having someone to care about something to do? Because I look at myself mostly since my accident, I tried a lot of jobs and I've been sacked from them all. I'd love to do more work but I know lot of thems makes me realise I can't, I mean, Guisborough but there was only this one kind of came out

of Guisborough because there was a college it had sixth form college that would employ me. They got rid of me now. And I was lost. I need to be in a bigger place like York or, Newcastle was good. Somewhere where there is work more like, I might be able to try, even part time work. I want to try as well. But this is a pause in my life while I'll get my daughter to school and stuff after I'm sure I'll start doing things but, I can't see what I can do within reach a Guisborough I need to live in a city if I want to work

INT1 **yeah it sounds like work it's quite important to you**

RES You understand this come on, With an education you don't want to rot away there do you? ,

INT1 **Definitely. What was your sorry just gonna ask about your your degree what was what was your degrees in in your PhD...**

RES That was in, my degree was Aero thermal engineering. So I was designing planes and stuff and then my PhD was more plane design for the US military which was it was fascinating but

INT1 **I bet**

RES Again, nowadays all that kinds of things well guess it's things you couldn't really model on the computer but a lot of engineering is now on a computer which again, I went back to the job was employed by the steel works over in Middlesbrough. But they got rid of me because all it could make me do is work on a computer and again computers cripple me, they tireme out reading all the time and

INT1 **yeah**

RES yeah fatigue fatigue fatigue everyday for example, I sleep I still sleep an hour a day. Without fail

INT1 **and hasn't been anything that you found particularly difficult that lockdown**

RES No I've enjoyed it because they've been a lot fewer cars on the road, which for me has been wonderful. Yeah, I guess (laughs) I say the obvious things like buying pasta was quite awkward. And I guess buying flour in other parts the country was harder. But erm no, and honestly, like less and less trains less buses, but that's only once in a while. We'll do that

anyway. So for example, for me once a month, we'd go to Middlesbrough go to Whitby on a bus. But no

INT1 **But that's about it Yes, not much. And can I just check as well, when you mentioned about your your accident? And I think, did you lose consciousness for some time? Were you in a coma?**

RES Yeah, it was apparent I was six days in a coma. And then induced me for a further four days.

INT1 **Okay, wow**

RES because I my brain had a brain swelling and stuff.

INT1 **Yeah.**

RES Then I had basically a month after my accident, I was talking about, and I can't remember any of it. So I was in hospital and they got jaundice and things that gave me some drugs they shouldn't have given me and yeah, hospital for four months. But you don't really appreciate it. It's just I just woke up in hospital. I didn't think why or anything. I was in a coma over Christmas, as well as the 23rd of December had an accident. So a cheap Christmas (laughter)

INT1 **I bet your mom didn't appreciate Christmas dinner at the hospital mind.**

RES Exactly I was doing my PhD at the time I'd done all the work. I was just a stage of just about to write it up. And I came came home for Christmas. And that's me mate my mate Mike for he wants to go to Dolby forest some mountain biking on the 23rd of December. So we did. I had my accident and I was out of my PhD for maybe 10 months and I went back down to Bath for a year just to write it up basically with a lot of help. Well, a lot of help, probably I do write everything was written down into sort of very staccato within the I couldn't really to get the flow that you need for that writing I mean, I had five journal articles and stuff i have i journal articles and stuff as well. But obviously a lot of help find a supervisor to word them correctly. I've done all the work for them, but the wording piles up. But with a PhD with the Viva stuff. I wasn't given any help. It was genuine. I did it properly myself, which was satisfying, you know, that they didn't know had an injury or anything so..

INT1 **fantastic. I mean, that is so much achievement, especially if you you mentioned sort of the tiredness I imagine back then as well. Fresh. The fresh brain injury would have been even harder for you. So that is so yeah, I'm in awe so (laughter) well done. Very impressed**

RES It means nothing at the end of the day, though does it. I've got a certification but so you know, it's funny because all my friends have moved all over the world. They're living the life I could have been living you know, getting paid a fortune. Even my brother you're getting paid a lot of money. Yeah. And he was always my thick younger brother. You know.

INT1 Like I said, money, moneys not everything, and you've got that that wonderful little girl.

RES That's exactly the key. Yeah.

INT1 Keeping you going there. They're a joy, aren't they? Especially at that age.

RES Yeah. Yeah.

INT1 Probably not answering back too much yet is she

RES Are you joking? answering that constantly. Because she's so happy all the time you know so?

INT1 Great, isn't it? Yeah, I've got a five year old and a nine year old. So. erm yes, they bring you joy and frustrations all in, In bags of err Yeah, there was their challenge will say especially homeschool. I'm pleased that done with

RES That must have been hard for you that Yeah.

INT1 And Sam do you have any questions before we move on to the scales?

RES No

INT2 For me, it was just one thing that stood out was how you said like when you were talking about wanting a job and wanting to be able to help people. And then when we're talking about lockdown you said about how having your daughter has helped you through a lockdown? Do you feel that the, The responsibility that comes with being a parent, especially having a young child is something that helps fulfil that need for helping others or do you think it's a different type of helping people that you would want through a job?

RES No, it's very similar because again, I know people in Guisborough but one person who's is probably function and stuff, but he's got looks after his children during the day. Because I guess you understand it being a mother. If you've got children, I'm sure you had a break in your life while you brough your children up for first few years and you always need someone at home to look after your child. So say, I say there's a break in my life basically. I'm sure my life will move on after this but yeah, that's that's my view.

INT2 Yeah, was everything for me.

Interviewer(s): **INT1**

INT2

Respondent(s): RES (P25)

CARER

INT1: Lovely stuff then. With all out the way then we can actually get going now.

RES: Right we're off.

INT1: So if we could just get a few demographic things to start with? Would you mind telling us your gender identity?

RES: I think I'm male.

INT1: Alright. Lovely.

RES: Only just!

INT1: Your age?

RES: Erm... sixty... four.

INT1: I struggle to remember mine as well, sometimes and I'm only 23 [laughs]. What area of the country are you based in?

RES: Well, where are we? Tyne and Wear. North East.

INT1: Alright, cool, North East. Your employment status?

RES: Not if I can help it.

INT1: Unemployed, retired?

RES: Sick.

INT1: Alright. And what was the other things I've forgotten what they were there's a couple of others-

INT2: Who do you currently live with?

INT1: Yes, that's the one.

RES: My sister.

INT2: Your sister, just the two of you, yeah?

RES: Yep

INT2: Yeah. Is she your carer- do you have a primary care at all?

RES: Erm... yeah I suppose you can put her down as that.

INT2: Yeah.

INT1: Alright. Is that everything?

INT2: And would you cast yourself as single or are you in a relationship?

RES: Single.

CARER: What they asking you?

RES: Just who's my carer.

INT2: That's it from me [INT1]

INT1: Alright cool, so if you could just start by telling us a little bit about-

RES: How do you get rid of this?

INT1: You what, what's that?

RES: We've got a black stripe across the screen, the centre of the screen.

INT1: We've had some people complain about that before haven't we? I think it's just a Teams issue. I'm not sure if you can do anything about that.

INT2: Is there- if you click on the screen is there a little button at the far right like an "X" or a "dismiss" that you can click on to get rid of it?

CARER: No

INT2: Nothing in there? Does it say anything on it?

CARER: No it's the record screen with the video and microphone things on it.

INT2: And there's nothing on that click- to clear it off?

CARER: No.

INT2: It's not going? Can you pick it up and drag it... out the way or...?

CARER: [Inaudible] see what that says what that says. Turn off a video, no, we don't want to do that. So we'll just have to leave it.

INT1: Alright

RES: It's alright

INT1: As long as you can hear us.

INT2: Sorry.

CARER: Just we can't see [INT1]'s eyes.

INT1: Alright-

CARER: We can see you, [INT2], but-

INT1: -you're not missing much [INT1 and CARER laugh]

INT2: They're beautiful eyes as well [laughs]

INT1: They don't work very well though.

RES: Yeah. No problem, carry on.

INT1: Alright, cool. So if you could just start by telling us a little bit about your brain injury?

RES: Erm... what about it it's erm... I fell off my bike, I got run over, and then I went to sleep for a few months.

INT1: Was that erm- so did you- so you got ran over while on a bike and that was in your- so you were unconscious for three months was that?

RES: Yeah.

INT1: Yeah. How long ago did that happen?

RES: [Inaudible, connection issues]

[Break to try and fix connectivity]

INT1: Erm... is- can you hear us?

INT2: No, you getting anything, [INT1]?

INT1: I'm not no.

INT2: Oh, are you back?

INT1: Oh are you there?

RES: Pardon?

INT1: It kinda froze just as you were talking there we might need to get you to switch your camera off just so we can- because for some reason the- use- the connection seems to be better when you don't have the camera on.

CARER: Right, so we put that off?

INT1: Yeah.

CARER: See what that's like?

INT1: Yeah.

RES: Can you see it now?

INT1: We can hear you yeah. So what was that you were saying about your brain injury? how long ago it happened?

RES: Happened in- 1st of October 1978.

INT1: Oh, some time ago now.

RES: I fell asleep... I woke-

CARER: Came off your bike.

RES: -I started to wake up... I've been told the fir... 30th of December

INT1: So that's nearly... nearly three months that isn't it, yeah. And how did it affect your life after you had the brain injury? And also thinking of how long ago it was how old were you?

RES: [inaudible, connection issues]

INT1: So where we were at was, I think you said that you had your accident in 1978?

RES: Yeah.

INT1: So how old would you have been then?

RES: Pardon?

INT1: How old would you have been when that happened?

RES: 21.

INT1: 21. So just to get a bit of an idea of like, where you- what sort of like phase of life you were in at that point. How much did you feel like your life was affected by the accident? And the- any, like, just ways in which it it's affected the way that you behave or can do things?

RES: Erm... how it's effected me? Erm... I don't know-

CARER: Your arm.

RES: I've only got the one arm now.

INT1: Did you lose that in the accident or...?

RES: Yeah.

CARER: You haven't lost it.

RES: No I-

CARER: No, he hasn't lost it.

RES: I haven't lost it I've still got the arm, it's still there-

CARER: It's paralyzed.

RES: -it's just paralyzed.

INT1: Alright. And so-

RES: I had- the wagon went over my head, and that put me to sleep for few- for a while. And then I woke up... would have been mid-January... Mid-to-late January when I woke up- myself. And they kept me in hospital for a few weeks and kicked me out. And that was it [laughs].

INT1: And so when you've lost the movement of your arm, is there any other part of- parts of your body that you've lost the use of or is it just the arm?

RES: Just the arm.

INT1: And did you- after the- so is there anything else that might have been affected? Or is it like just the arm movement that you've lost? Like not not just like-

RES: I-

CARER: Yeah he got a traumatic brain injury. They can't see.

RES: Dislocated- aye they can't. My... left wrist, I dislocated my left- left wrist.

CARER: Yeah, he has the brain- he has the brain injury, so he has a great deal of trouble with concentration, memory, and stuff like he can't follow- he gets easily confused if you tried to give him instructions. You can't give them a list of instructions- you know two or three instructions, you've got to go through things one, do it and then give him the next one.

RES: Yeah one at a time.

INT1: And so after you had like one- once these like effects came in your life from the injury have they- any of them gotten better or worse over time or have you had any sort of rehab to try and help deal with them?

RES: No-

CARER: Yes you have.

RES: What, erm-

CARER: Leatherhead.

RES: Oh, yeah, I tried to do- tried to go back to work well [laughs]... Went into hospital down to St. Mary's in Paddington-

INT1: Yeah.

RES: -for nerve- nerve tran- muscle transfer. And but that was no- that didn't work and when they- I came out the- came out of St. Mary's I wen- took- sent me to Stanmore... Stanmore Royal National Orthopaedic Hospital... and up there the... occupational therapist or whatever put me in touch... no it wasn't an occupational therapist. Somebody from the employment they got me onto a course at Leatherhead on welding which I tried and... realised I couldn't do it. And then I lived... I got a job down in- down in a place called Chertsey which is just a few miles away from Leatherhead. I tried to go to work down there and I had a few accidents in the workshops... in the workshop so. They reckoned I was too much of a liability to be employed- employed there, so they said goodbye. I came back up north and... I... started doing- I went to a... employment rehabilitation centre at Gateshead... and they got me in... Gateshead.

CARER: Was that when you started doing the O levels?

RES: No, that was erm... They got me... They told me I- well I couldn't do any work. I wasn't unemployable. And I finished there and I started just tending to seeing a psychologist, at Durham Univ- at erm...

INT1: Durham University?

RES: Durham- no Durham County- Durham County Hospital.

INT1: Alright.

RES: And at the same time as I was doing that, I also started attending a course for handi- for the handicapped at Washington Multi-purpose Centre... and I did that for a couple of years then I tried to go back to college to get some O levels or whatever and- so I- this would be '86... yeah '86 I went back to college and tried to do a couple of O levels-

CARER: Which you got-

RES: I didn't I got two big U's... English and psychology- not psychology... sociology, that's erm... yeah. I got two big U's for that and then I tried... tried thinking about going back to work in '87 and it's in... I think there's- about the May, I think in '87 when I was... offered a job-

CARER: Was that in London?

RES: Yeah, I was offered a- I applied for a job with Lambeth...

CARER: Council.

RES: Lambeth County Council in London. And I just on as support worker for the long stay hospitals that they were closing down down sou- down in Kent. And I went down to London and tried to work there, but my memory did as it always does- just cut me off that and I was forgetting people's- people's names and other things that I shouldn't have. And so they said goodbye and so I came away from there. Went back to college. I tried to do maths O level and... sociology again. Yeah, that's right, yeah. And the maths sort of clicked with me I got a- well, I just I just did an intermediate course so the highest side get would- was grade C. And I managed to get a grade C in maths then. I got another U for sociology. Went... when... I thought I'd, I needed some sort of help. So the next year... I went back. No, this is, erm, sorry... this was eighty-erm... this was '88, I'd been down to London... ah yes this was ninety- ninety...

CARER: It doesn't really matter.

RES: Must've been ninety...

CARER: It doesn't really matter, what did you do?

RES: I tried to erm... I tried to do another course and... I tried- I tried, tried doing it A-level maths and, but I- I just couldn't- couldn't understand it couldn't take it all in so I got through and I parted company with them... that would be '91

INT2: [RES] Just- just wondering had you been diagnosed with a brain injury? [Audio cuts out]

RES: Pardon?

INT2: -caused? Did they know what damaged- [audio cuts out] with you brain injury?

RES: What damage with the brain injury? Well, no I don't think. I- they cut a few holes in my head-

CARER: I'll go and hunt that doctor's report out from the '80s.

RES: I think it's- my sister's gone to get the doctor's report from... from the '80s from when I- exactly what hap- what happened. But they cut... five- five burr holes in my head. Couple, couple here, and couple here, and then there was another one round here, where that's where the wagon went over my- went over my head. Must've been- must've been about there. I was looking away, so I didn't see it coming [laughs], you sneak up behind me and ran over me. Nevermind, but... then that was what were you asking about there?

INT2: I was just wondering as soon as you had your accident that they realised sort of the long term damage that had been caused? Or was that later on in your life before they realised?

RES: I don't know. I don't think they- they just wanted me out of hospital. It was, erm- was, because at the time when I... when I had my accident the hospitals were always- industrial unrest, the strikes and everything were all around the- coal miners strike started soon after I had my smash, or soon after I came out the hospital. That was eighty... '83 or '84... the coal miners strike. Yeah.

CARER: I don't know where it is.

RES: I'm sorry, I'm getting mixed up again, now. What were you...?

INT1: So when it comes- so if you didn't necessarily get any sort of like diagnosis of any, like, brain- like, specifically brain injury or like- like what's up with what's going on in here?

Have you had anything like sense and more recently, like as- maybes the understanding of the brain has got better?

RES: I... have, erm... I've done all sorts... tried to do all sorts... I'm sorry, do you mind- would you mind repeating?

INT1: Have you had a so- since you had the accident, it doesn't seem like you're necessarily were given like a diagnosis of what was wrong. As time's gone on has any like and you've seen more, like, specialists or anything like that, have they given you a diagnosis?

RES: The City Hospital, Sunderland. Dr. [name], consultants neuro- neuro rehabilitation at City Hospital Sunderland. Can you read, here's my sister.

CARER: Yes, he saw Dr. [name]- erm Dr. [name]. Just Headway had said it'd probably be advisable to have a another neuro consult because it'd been so long since he had one. And there was really nothing he could do. The only thing that could try was he gets phantom pain in his paralysed arm, and try him on prega- pregabalin to see if that would help with the pain. And he also has double vision in both eyes. And so- and sent him to the eye hospital to see if there was anything new that they could do. At that time, there wasn't and the pregabalin didn't agree with him. It seemed to make him more confused. So everything was stopped again. He did have a letter but I can't find it, from the hospital originally. You know what happened to him in the accident. But I can't find it at the minute. So I don't know.

INT1: So when it come- when it comes to rehab and things like that, is that like more of a recent thing as you've found things like Headway?

CARER: Yes. Yeah.

INT1: How How do you, for [RES], how do you feel that Headway has helped and if there's anything that's been particularly hard without and maybes even with Headway?

RES: There's nothing- nothing- nothing been hard with Headway, there's erm...

CARER: Headway's helped with meeting people and-

RES: Yeah, they've helped me to get various things and helped me to do do things- a lot of things I would never have- I don't suppose I would've done. There's people wouldn't have met.

INT1: When it comes to meeting people as well as we've come in lockdown the last year or so is the many people that you've been able to see or if there's people that you used to see before lockdown that you haven't been able to since?

CARER: You see me now, [names other sister].

RES: I only see my sisters.

INT1: And is there anyone that you used to see before lockdown that you know don't because of it?

RES: No I don't- because of it?

CARER: Because of the lockdown, well Headway people.

RES: Oh, because the lockdown... Yeah, I don't- I don't see the- don't have meetings with everyone from Headway. That was... then they were just about the only people I saw anyway. I've been in the house, that much. And I, well, I used to go out for rides on my bike and I haven't done... I haven't done any of that... Since the lockdown.

INT1: When you say as well about how you haven't been able to say the people from Headway, do you think that seeing less peop- how do you feel about having seen less people like as has it bothered you at all?

RES: I don't think so. No, I don't think it's bothered me too much.

CARER: I think headway has been good that they've been doing mainly weekly or two weekly Zoom sessions. And it's not with the majority of people but at least there's somebody there to talk to and [name] been a great help because she's the focus of it all.

INT1: Would you say that like the online technology is all it's, like, given you a sense of continuity from the change of lockdown to- from pre-lockdown life?... It's fine if not.

CARER: I don't think he really understands what you're asking him, but yes it has. I mean, having the technology there has helped.

INT1: Yeah, so yeah- basically while he's gotten that like as- since you've been able to kind of keep in contact with them- which is basically has that helped lessen the impact that could have had?

RES: Erm, I don't know... it's, erm...

INT1: Yeah that's fine.

RES: I don't know if it has or it hasn't. Haven't had that muchh contact with them.

INT1: Support might be hard to say when you don't know what the alternative could have been. When you say about your bike as well and not going out on your bike as much since lockdown started. How- what would you say you do in an average week now because of lockdown? Now there's Zoom calls with Headway.

RES: And sit here, listen to the radio.

INT1: Do you- enjoy the radio as anything-

RES: [Inaudible]

INT1: What was that? Is there anything particularly that you like to listen to on the radio, like you'll find engaging, or do you just listen to music?

RES: I don't. I don't listen to music.

INT1: So is it like chat show things?

RES: Just- no- Radio 4.

INT1: Alright. And do you- how- do you think that- that- has that changed at all since the beginning the lockdown or is it- do you like listen to them more because you don't go out as much?

RES: No.

CARER: Well you do listen to it more.

RES: Well, I listen to it. There's nothing else to do. Yeah.

CARER: You do word searches.

RES: Yeah, I do word searches, listen to the radio.

CARER: Been doing jigsaws.

RES: Jigsaws and... anything to kill time.

INT1: I think a lot of people have been like that during lockdown just wanting to do something to keep their mind occupied.

RES: Yeah.

INT1: How do you feel about what you're doing on a day-to-day basis at the minute?

RES: How do I?

INT1: Feel about what you're doing on the average day?

RES: It's just got to be done, with having lockdown. It's either this or... what? [Laughs]

INT1: So would you just say that you're kind of indifferent or that you don't really have any sort of feelings towards it?

RES: Yeah just accept it and...

INT1: Okay.

RES: Sorry.

INT1: What would you say has helped you through lockdown and have you found it any more difficult then life before lockdown?

RES: No, not really. I just can't getting... well... I just get out on my bike.

INT1: Is that when you're talking about being on your bike as well is the anything specifically about lockdown that made you decide not to do that? Because, obviously when we went first into lockdown, you were allowed like an hour of exercise outdoors I think it was?

RES: Yeah.

INT1: Had you just decided not to leave the house because of the threat of the virus?

RES: That plus my sister's in high depen-

CARER: Shielding.

RES: She's in the shielding... she's... she's a much higher priority than me. So I'm shielding- I'm shielding for her.

INT1: Yeah.

RES: So she can't go out, or she's not supposed to go out. So there's no sense in me going out and bringing things- bringing things back to give to her.

INT1: Yeah.

RES: Which defeats your object of her shielding.

INT1: Yeah that makes a lot of sense, yeah,

RES: So I don't- so I don't go out either.

INT1: How do you feel about life after lockdown and, like, obviously, now we were supposed to be out of it and has been put back by four weeks. How do you feel about what things are gonna be like, once we're 'back to normal', as they say?

RES: I don't... don't see there being very much difference.

INT1: Are you looking forward to potentially going back out on your bike?

RES: Yeah, yeah, I'll get out on the bike again, yeah.

INT1: Is it basically just for you that you feel like- that you'll be able to go out on your bike and then get back into Headway, is that the only real difference for you? Or is there anything else?

RES: Yeah. That's... that's about it. Get out- get the bike out and see people I used to see through at Headway. And... I don't know... I'm sorry.

INT1: Are you alright?

RES: Yeah, I'm okay, just-

INT1: Alright, cool.

RES: -I just can't think of anything to say.

INT1: Yeah, that's- that- that's fine, yeah, that's totally okay. Moving on from lockdown. When it gets to things like loneliness, how would you define loneliness? Like what does it mean to you?

RES: Loneliness... I don't know, I've never been lonely.

INT1: Well that's always good.

RES: Well-

CARER: He doesn't have sort of normal emotions. So he doesn't normally feel bored or lonely or happy or... like anything, really? Just is.

INT1: When- is- is that something that you've always been like? Or did that come from the brain injury?

CARER: From the brain injury, definitely.

INT1: Do you think that it was because of, like, direct effect on the brain, or just because of adapting to changes in life and like, kind of not really being fussed about things?

CARER: A direct effect. When he was in- unconscious in the coma in hospital, and the doctors there said, like to be- because he was always very laid back and easygoing. Erm, and they said, "be prepared", because if he did wake up and when he did wake up, he could be totally the opposite way. He could be very aggressive.

INT1: Yeah.

CARER: But luckily, that never happened. But there's like no real emotions. Don't you think?

INT1: Yeah, I'm just- I'm thinking of some things that we've learned about in psychology about like, how it can affect personality in like, things in the brain. So you would not- you'd say you'd never been a particularly lonely person? Do you think that that's just because of the brain injury or do you think it's, like, if you, like when you say you don't feel things normally. You think you might have been if-

RES: It's a coping mechanism, I... my memory's no good. I can't... can't remember things. So, if I don't have to- if I don't have any... contacts and that I don't- I can't upset people by not remembering them, not remembering them. Not remembering meeting people.

INT1: Yeah.

RES: Because I can't I can't remember meeting people anyway. So misplacing where I've seen someone before, so I say a good morning, to, to the same person three times- three times on a morning and... haven't realised that I've done it until someone's pointed it out to me. Just try and be friendly and carry on. There's nothing not much I can do about it.

INT1: Yeah... Do you erm- do you think that if you ever were to actually feel lonely, do you think you'd have people around you to talk about it?

RES: Yeah I would do if erm... I've got my family here. And as you say there's Headway, people through there. Yeah.

INT1: Okay. That's... cool- that's everything from me. Anything you'd like to ask, [INT2]?

INT2: Nope, I think we've covered everything.

CARER: I have found the letter sorry, the letter that said what was wrong with him at hospital. Right it was saying he was admitted to Pontefract Hospital with a fracture of the- unconscious, a fracture of the jaw and left wrist. Due to the gross head injury, he was transferred to Leeds. Two days later, his condition deteriorated and a burr hole operation was performed. And this reveled is a collection of blood under the skull, over the surface of the right side of the brain. Also noticed was diffuse swelling of the brain on the right side and also had a fractured jaw, that was both sides. Treated by interdental wiring, left palm surgically decompressed due to extensive swelling, and it was decided not to treat the fracture of the wrist because of his overall medical condition. Remained unconscious for several weeks. And post-operative recovery was jeopardised by a chest infection, which required performance of a tracheotomy and assistance with ventilation, but he had that all the time actually he was unconscious, had hyperventilation. Tracheotomy tube was removed on the 24th of November. Shortly after that he was transferred to Newcastle Hospital and then to Dryburn at Durham. On admission he was found to be- also had a paralysis of his right arm, I think that was only found at Dryburn, like in December, or the beginning of January, because until then he was comatose... and wasn't responding to anything. Received intensive rehabilitation in the form of physiotherapy and occupational therapy, to improve coordination, and gait. Speech theraty- speech therapy to improve speech, and then the Coptic clinic for treatment of double vision. On the 8th of February, he was discharged at [inaudible] he was discharged. So...

INT1: That's quite a list.

CARER: Yeah, that's what it says here. Seen regularly in outpatients up to the present time, which was... 1980, July 1980. And although originally had showed some recovery of sensation in the upper right arm, slight function in the biceps muscle, there's been failure of any further improvement. He has therefore been prescribed to polythene splint to wear. He was put on epilepsy drugs, initially, but then taken off them because they know they didn't think he would have any fits and he hasn't. Yeah. So just says here neurologically, since the accident, he is more passive, has very poor memory and concentration, a lot slower in responding to verbal questions and commands, very clumsy and needs to be supervised in his daily routine. And while he can undress himself he needs, oh, well then he needed help in getting dressed. Arm totally paralysed, and therefore food has to be cut up. He can feed himself, still has to double vision. Learning to write with his left ar- with his left hand, which he does fantastically now. But then it was saying it was like very difficult to master-

RES: 8th of February, was it?

INT1: I'm left handed and not even there with good handwriting [laughs].

CARER: No, well, I can't write with my left hand. Complains of askew jaw bite, and feel that his appearance has been altered because of the accident. Well he had all his teeth then removed and had false teeth. Because they were just crumbling where all the wires had been.

INT1: Yeah.

CARER: He answers questions put to him logically, if somewhat slowly. And then just about the burr holes in the skull... there is a right bonus syndrome? Never heard of that, small right pupil. Some, I don't know how you pronounce it, p-t-o-s-i-s. I don't know what that is. And his jaw bite has been-

INT1: I've never heard of that.

CARER: No, well, erm-

INT1: [Lead researcher] will probably be able to make some sort of use out of this when he gets to listen to it [laughs].

CARER: Yeah, yeah. And then it was just saying about the, erm, like, well healed tracheostomy scarring. Right arm gross muscle wasting in the whole arm, the arm is virtually paralysed with only movement present being due to muscles controlling his shoulder blade, and very weak. Complete loss of sensation... So yes, that's... that's about it, really.

INT1: That's incredibly in depth. Thanks for sharing it. Some of it I don't understand what it means, but I'm sure [lead researcher] will will have a better grasp of it when he gets to listen back to it. But since that's all of our questions, we'll move on to the scales. So I'm going to share my screen now, if you can just let me know, that you can see my Word document here.

RES: Alright I'll just nip out to the loo.

INT1: Can you see that?

CARER: He's just nipping to the loo for a minute.

INT1: Alright, okay no bother.

CARER: Oh, well, that's hidden behind this bar across the middle.

INT1: Alright, well, I'll read them out anyway, so it should be okay.

CARER: It's very difficult, I mean, I hope you realise it is very difficult for him to answer questions.

INT1: Yeah.

CARER: Because sometimes while he's thinking he forgets what he's supposed to be thinking about. And then trying to put into words and things, you know?

INT1: Yeah there's been a few times I've been saying things and saying, and thinking "I need to simplify this", because I'm very long winded with some of the things that I say.

CARER: Yes, and he just remembers the beginning bit of what you say or, or takes one bit of it, and forgets the other- the rest of it. It's sort of hard work at times, you know, well, most of the time, actually. But I mean, he's brilliant.

INT1: It's really good for us as well, that, like, he's been able to persist because people who've got struggles and stuff that's gonna be useful for us going forward. Like, if we just had loads of people who talk like just for days without thinking about it and haven't got any problems then we wouldn't really be able to make any use out of it.

CARER: Yeah. He tries to get like, when you're asking him like a timescale of rehabilitation and stuff, and he tries to sort of pin down

INT1: The entire timeline.

CARER: Exact times, you know. Because it is in there, he knows it's in there and it's amazing because he- having this Zoom meeting probably two hours time he'll barely remember it and I'll say something about near say "what? what?" But the longer back it is, the more he can remember. He amazed us once- we found some old- an envelope of old photos and we were looking through them and they're ones taken before his accident.

INT1: Yeah.

CARER: And as we're going through them, we said "Oh where are these?" and he said it was some motorbike rally and he said "oh well the next one will be so and so walking across the field" and that was the next photo. You know he knew the order that were in-

INT1: Yeah.

CARER: -in this envelope and as I say that was before his accident but, totally different since.

INT1: So is that a case of where like... if something happens, he'll forget about it and then the further away from he'll remember I was I just thought the further back in time things are he has a better memory of them because it was before the accident?

CARER: Oh well before his accident his memory's fantastic, but he has quite a good memory up to about 1986/87 he has a- he has to think hard about it but his memory is better for that period of time than for anything since then. So I don't know whether that's, you know, gone into the long term memory and he can access it easier, I'm not a psychologist so I don't know... But he has got a good memory for things way back.

INT1: I think that- I might be misremembering things from earlier on in the course but I think that long- and short-term memory, like, I think are pretty much all memory is actually long-term memory. I think short term memory is like only actually bracketed it into, like, seconds or minutes, rather than like, days or weeks or like anything after about like an hour or two I think that's actually down to long term. Am I right, [INT2]?

INT2: Yeah, well it's a few- short-term's very short.

INT1: It's a lot shorter than we all would think before you learn about it.

CARER: It's not that then, erm, I mean his, well, his memory of things in the recent past, very recent past would practically be non-existent.

INT1: Yeah,

CARER: You know, but he's back here so you can ask him your other questions.

INT1: Okey dokey, we've only got... probably about 40 just little things of how much you agree with them, basically.

RES: She brought into the shop [audio cuts out]

INT1: You what, sorry?

RES: When I used to go to the shop for my- for my mother she had to write things down write a list down.

INT1: Alright.

RES: Everything that she needed. I often still forgot it.

CARER: He'd get to the shop and forget he had a list so just buy anything.

INT1: Yeah.

RES: Yeah, my- my memory is not very good.

INT1: Understandable, given what you've been through.

RES: Forget... forget things practically immediately. I hear them and try and think of- try to repeat them in my mind and I forget them.

INT1: Yeah

RES: So not very good, never mind, but... carry on, sorry.

INT1: For- no bother. For our scales here. This first one we've got it's just six statements and basically whether you- it's about you in general. So if you strongly disagree; disagree; neutral; agree; or strongly agree you've got I tend to bounce back quickly after hard times?

RES: Yeah, yeah. Carry on.

INT1: Would you say agree for that one?

RES: What?

INT1: I tend to bounce back quickly after hard times. How would you rate yourself on that one?

RES: After hard times, I have never had any. I imagine be pretty much [inaudible]-

CARER: No, that's where the emotional side comes in. But it isn't there that he- each day is just the same to him. He was refused ESA about four years ago, five years ago. And so they took his money off him and said he had to get a job.

INT1: Yeah.

CARER: And then we fought it with a tribunal. But he just carried on and "well I'll go to the job centre and I'll get a job", and, you know? We had- we were there fighting for it for him, you know, and he go it overturned. But it hardly affected him at all.

INT1: Yeah.

CARER: But it did to us, we were stressed to death for six months, but it didn't affect him.

INT1: So would you say that you do bounce back quite well, from hard times, because you don't see them as hard times?

CARER: Yeah.

RES: Yeah-

INT1: Agree or strongly agree.

RES: -doesn't effect me that much.

INT1: Would you say you agree or strongly agree?

CARER: Just say something, [RES].

RES: I don't know, I agree.

[Progresses through scales]

INT1: Okay. You've got it does not take me long to recover from a stressful event.

RES: No because I forget about it.

INT1: So disagree?

RES: That's where a bad memory comes in handy.

INT1: A blessing and a curse. W

RES: Yeah [laughs].

INT1: Would you strongly disagree or disagree?

RES: Disagree.

[Progresses through scales]

INT1: There are plenty of people I can rely on when I have problems?

RES: Yeah I pile them all off onto them.

INT1: Lovely

RES: Onto my sisters [RES and Carer laugh].

CARER: Yeah stresses us out, and he goes along quite happily.

[Progresses through scales]

INT1: Okay, how often do you feel left out?

RES: Can't feel left out if you- you're not trying to get something?

INT1: Would you say hardly ever to that one then?

RES: Aye.
