# Supplementary material for: Uncovering the social determinants of brain injury rehabilitation
Source: J Health Psychol. 2023 Apr 7;28(10):956–69. doi: 10.1177/13591053231166263 (PMC10466963; doi:10.1177/13591053231166263)
Supplement: sj-pdf-3-hpq-10.1177_13591053231166263 – Supplemental material for Uncovering the social determinants of brain injury rehabilitation [file sj-pdf-3-hpq-10.1177_13591053231166263.pdf]

Dunne, S., Williams, G. P., Bradbury, C., Keyes, T., Lane, A. R., Yang, K., & Ellison, A. (September, 2022). Uncovering the social determinants of brain injury rehabilitation.

This repository contains the data, code, and analysis product necessary to reproduce the analyses described in Dunne et al. (September, 2022). *Uncovering the social determinants of brain injury rehabilitation.* .

This repository, along with materials and interview transcripts, is hosted on the OSF.

Please see the metadata file for a description of the files and folders in this repository.

This work is licensed under CC-By Attribution 4.0 International.

Reproducing the qualitative analyses

Full participant transcripts are provided in pdf format located in /data/compiled_transcripts.pdf.

No software was used for this analysis. Authors preferred pen, paper and sticky notes to visualise data. After data was transcribed, authors 1 and 1 familiarised themselves with the data by watching the recordings, conducting multiple readings of the transcripts, and made notes of preliminary ideas. Author 1 independently conducted general coding, highlighting, and labelling any relevant passages of text with descriptive code throughout all transcripts. To explore patterns of meaning, similarly coded extracts, along with a description of each code, were placed together in a table. After all transcripts had been coded, authors 1 and 2 met to discuss the interpretations of the data, agreeing codes, and discarding any not related to the research enquiry. All codes were sorted into categories and subsequently refined into themes. Data and coding within themes were analysed and inter-relationships between themes visualised. The themes were named and defined, and a written summary of the analysis grounded in participant data was constructed.

To ensure rigour in our qualitative analysis we adhered to the 15-point check-list criteria provided by Braun and Clarke (2006, 2013) which provides guidance on the processes of transcription, coding, analysis, and the creation of a written report. Examples of quality control within this study include ensuring transcribed data have the appropriate level of detail, checking recordings for accuracy, checking all data have been considered equally, confirming coded items have been collated and themes checked against the original data set, reviewing whether all data have been interpreted rather than summarised and that all analysis matches the data set and stating all assumptions and approaches to thematic analysis. Additionally, we employed researcher triangulation, with authors 1 and 2 having conducted the interviews, not only discussing, and evaluating their impressions of the data but also discussing their experiences of conducting the interview. Finally, throughout the data processing and analysis phases, author 1 kept a reflexive journal to log their thoughts, motivations, values, and assumptions in the research process.

References

Braun, V., & Clarke, V. (2006). Using thematic analysis in psychology. *Qualitative Research in Psychology*, 3(2), 77–101. https://doi.org/10.1191/1478088706qp063oa Braun, V., & Clarke, V. (2013). *Successful qualitative research: A practical guide for beginners*. Sage. https://uwe-repository.worktribe.com/output/934201

Reproducing the quantitative analyses in Python

The cleanest way to reproduce these analyses is to create a virtual environment in Python and then install the required packages using the requirements.txt file. VS Code is recommended to complete the following.

1. Install VS Code from https://code.visualstudio.com/download.
2. Install Anaconda https://docs.anaconda.com/anaconda/install/ or Miniconda from https://docs.conda.io/en/latest/miniconda.html
3. Set up Python in VS Code by following the instructions here https://code.visualstudio.com/docs/languages/python.

Using VS Code ope nthe folder for this repository (File...Open Folder...).

Next, create a virtual environment from which to run the analyses. For details of working with virtual environments in VS Code, see https://code.visualstudio.com/docs/python/environments or instead follow the quick instructions below.

Quick start instructions for using virtual environments

Using conda virtual environments

- To create a virtual environment: `conda create -n bi_loneliness python=3.9.5` .
- Activate the virtual environment: `conda activate bi_loneliness`.

Using venv

Open the terminal and run the following:

- To create a virtual environment: `python -m venv venv`.
- Activate the virtual environment: `source venv/bin/activate`.

Installing Packages in the conda or venv environment

Install the necessary packages from the requirements.txt file: `pip install -r requirements.txt` .

Running analyses in VS Code

Once you have activated the virtual environment and installed the relevant packages, in VS Code you can select your Python interpreter (from the virtual environment) using Cmd + Shift + P. Choose your recently created environment (if using Conda, this should be bi_loneliness Python version 3.9.5). Open the file /python/analysis.ipynb and click the Run All button at the top of the analysis file in VS Code. The whole analyses will rerun from the top of the file to the bottom.

Deactivating and removing the environment

If you want to deactivate and/or remove the environment after reproducing these analyses, follow these instructions depending on whether you created the virtual environment in conda or venv.

Using conda virtual environments

To deactivate the environment: `conda deactivate`.

To delete the virtual environment: `conda remove -n bi_loneliness --all` .

Using venv

To deactivate the environment: `deactivate`.

To delete the virtual environment: simply delete the folder containing the environment.
